# Supplementary figures and images for: Structural basis of human full-length kindlin-3 homotrimer in an auto-inhibited state
Source: PLoS Biol. 2020 Jul 9;18(7):e3000755. doi: 10.1371/journal.pbio.3000755 (PMC7373317; doi:10.1371/journal.pbio.3000755)

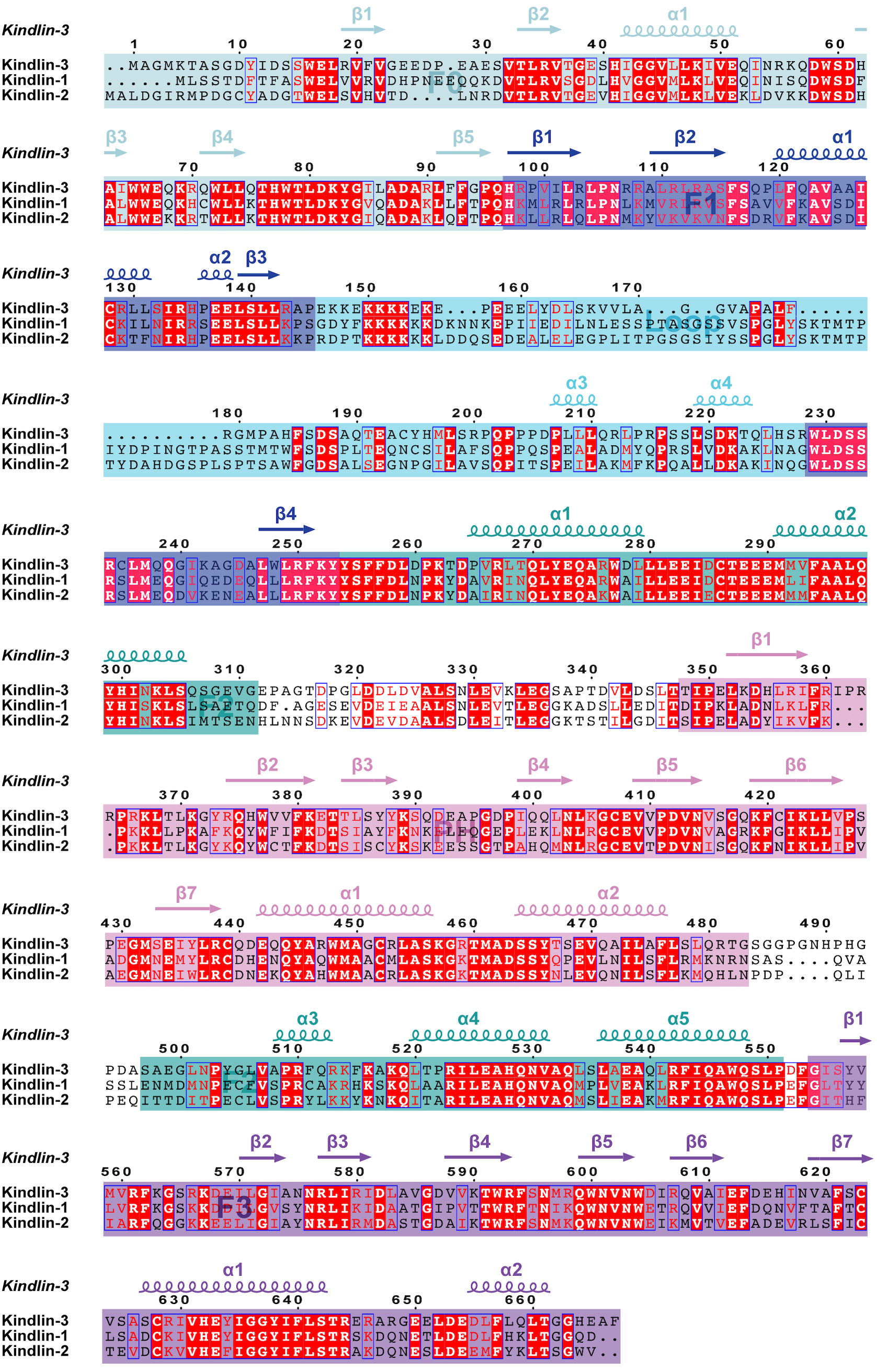

Supplement: S1 Fig — The domains and the F1 loop are highlighted in the same manner as in Fig 1A. Secondary structure motifs and numbering are based on kindlin-3. (TIF) [file pbio.3000755.s001.tif]

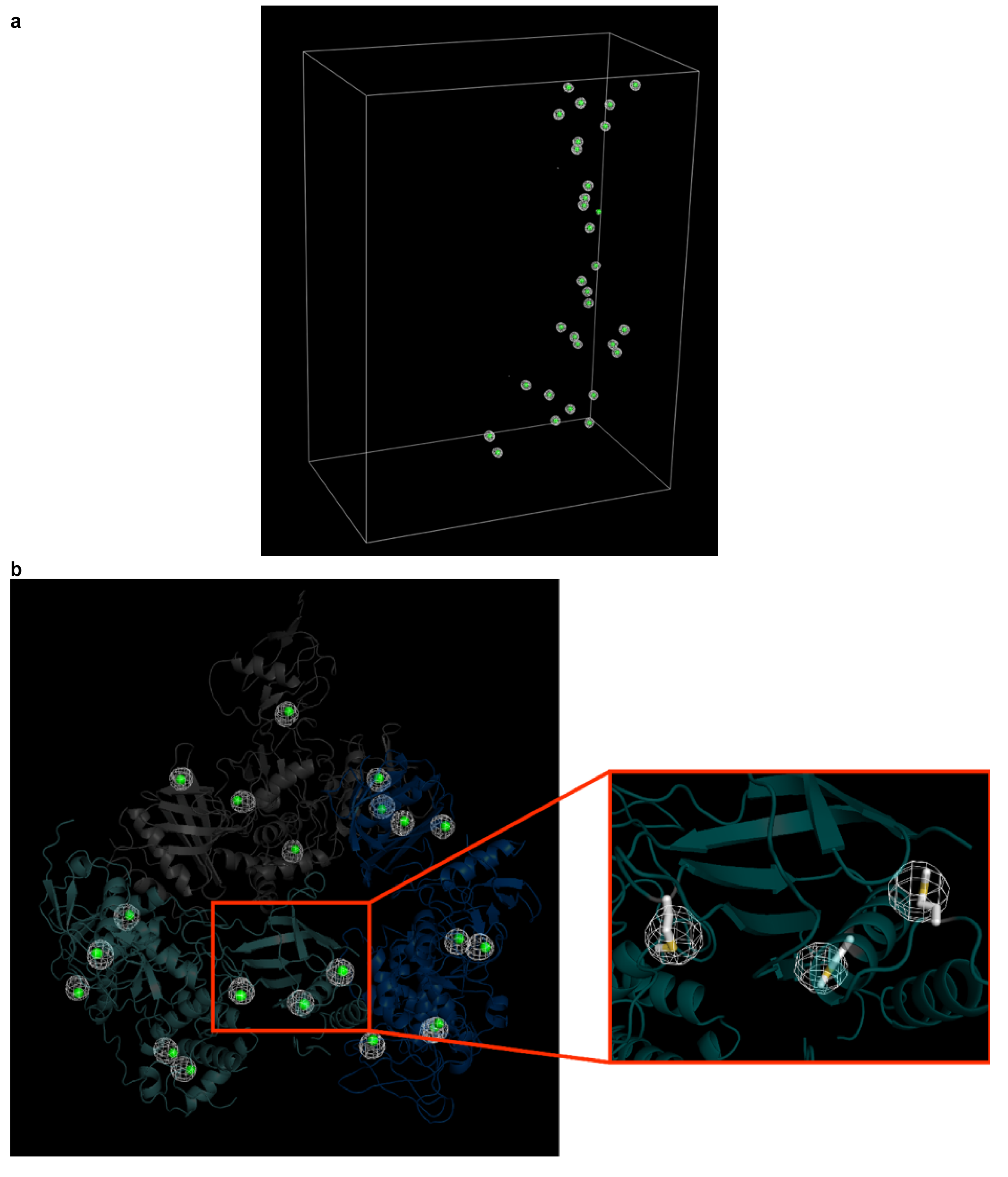

Supplement: S2 Fig — (a) The anomalous difference Fourier map is generated using Global Phasing Buster. The map is displayed with Se sites as green spheres and contoured at 3 sigma. Total 31 Se sites were shown in the unit cell. (b) The anomalous difference Fourier map (white mesh) is displayed with the final refined model (semitransparent cartoon rendering). The sulfur atoms on methionines of the final model are shown as green spheres. A zoom-in view of the methionine sites is also presented with 3 methionine side chains shown in stick model. Se, selenine. (TIF) [file pbio.3000755.s002.tif]

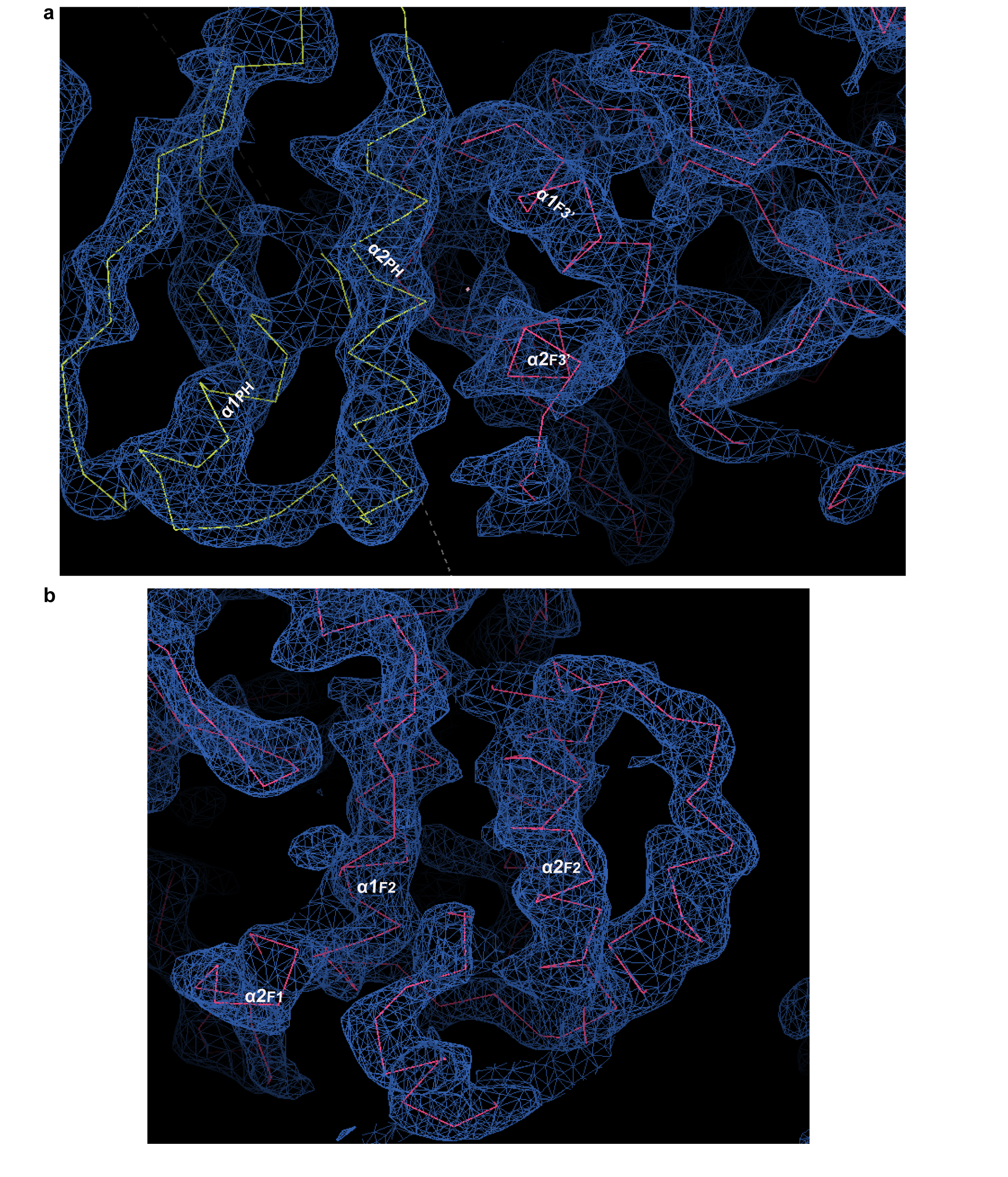

Supplement: S3 Fig — The representative 2Fo-Fc electron density maps are shown in blue meshes with the ribbon model of the protein. Secondary structure elements are labeled. (a) 2Fo-Fc electron density map of protomer–protomer interface. (b) 2Fo-Fc electron density map of F2 subdomain of one protomer. Fc, calculated structure factor; Fo, observed structure factor. (TIF) [file pbio.3000755.s003.tif]

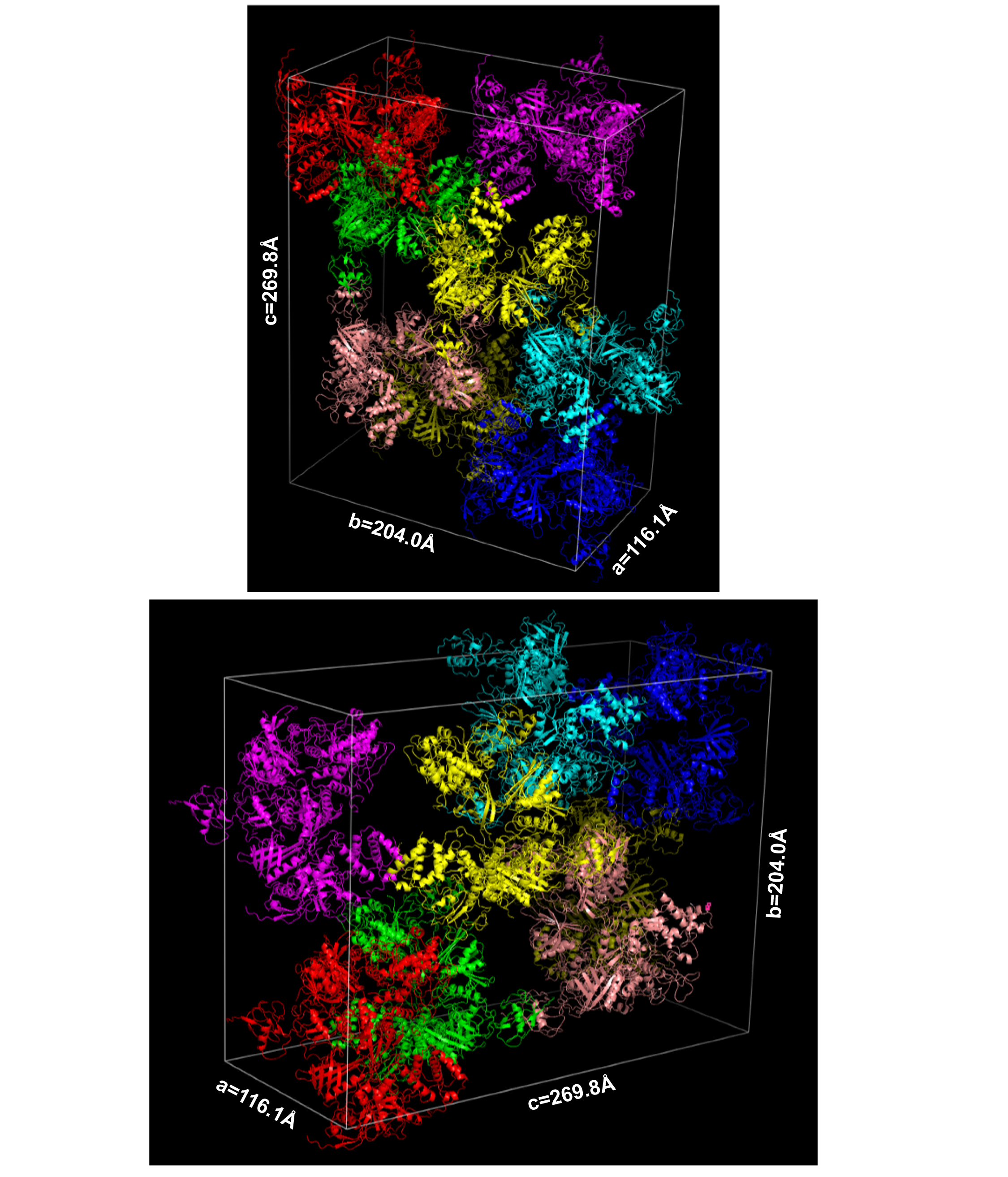

Supplement: S4 Fig — Each trimer is colored identically. (TIF) [file pbio.3000755.s004.tif]

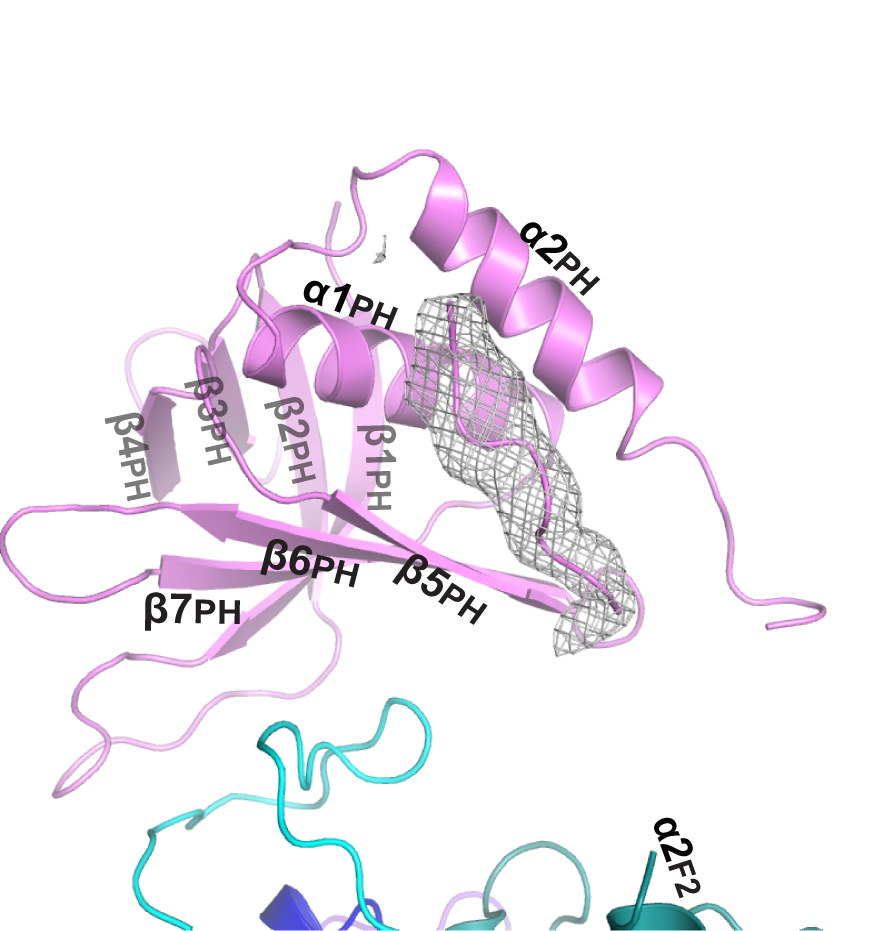

Supplement: S5 Fig — The modeled loop between α2F2 and β1PH appears to be a helix interacting with α2PH to stabilize the entire domain. Fc, calculated structure factor; Fo, observed structure factor; PH, pleckstrin homology. (TIF) [file pbio.3000755.s005.tif]

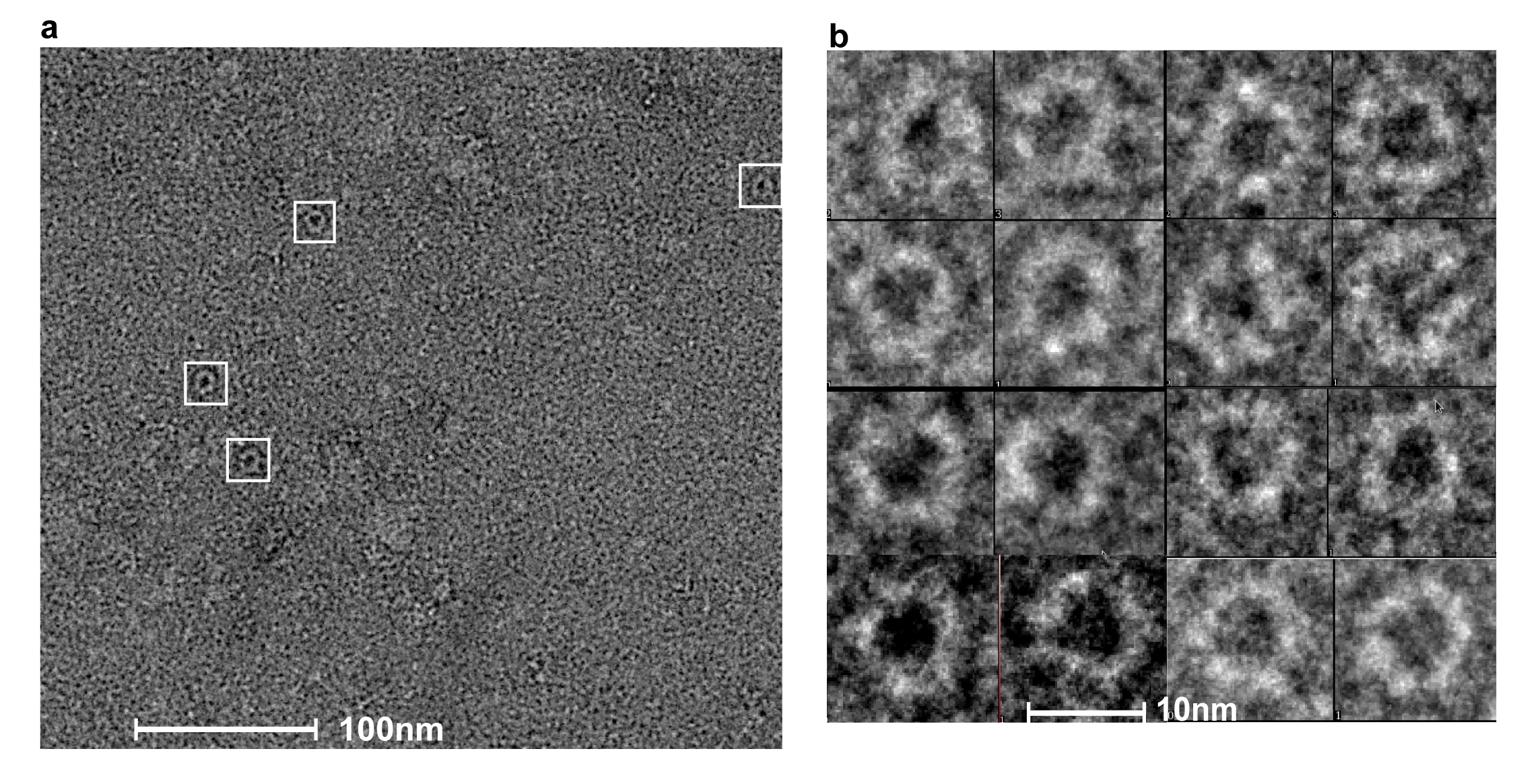

Supplement: S6 Fig — (a) Typical negative stain electron microscopy micrograph of kindlin-3 trimer purified from Sf9 cells. Kindlin-3 particles are highlighted by white squares. (b) Close-up view of kindlin-3 particles. Sf9, Spodoptera frugiperda 9. (TIF) [file pbio.3000755.s006.tif]

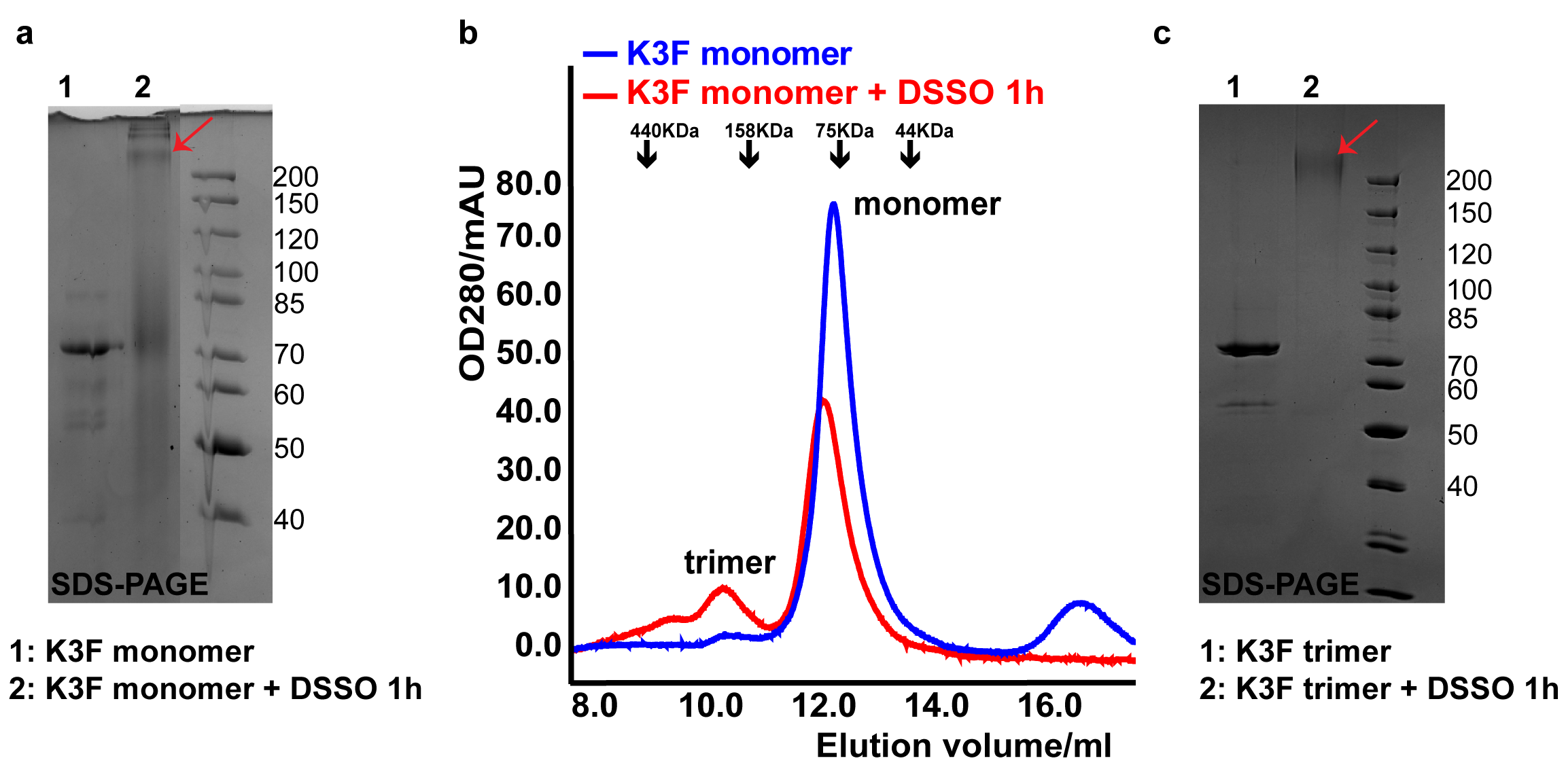

Supplement: S7 Fig — (a) SDS-PAGE of kindlin-3 monomer with or without DSSO treatment. Lane 1 indicates the native kindlin-3 monomer purified from insect cells. Monomeric kindlin-3 in solution gave a band above 70k Da. Lane 2 indicates the kindlin-3 monomer crosslinked by DSSO. Crosslinked trimeric kindlin-3 in solution exhibited a band above 200k Da (labeled by red arrow). (b) Analytical gel filtration chromatography profiles of kindlin-3 monomer with or without DSSO treatment. K3F monomer without DSSO treatment (blue) and K3F monomer with DSSO treatment (red): K3F monomer without DSSO treatment only exhibits monomeric state, whereas K3F monomer with DSSO treatment exhibits both monomeric and trimeric states. Note that molecular weight markers for analytical gel filtration chromatography are indicated by black arrows. (c) SDS-PAGE of kindlin-3 trimers with or without DSSO treatment. Lane 1 indicates the native kindlin-3 trimer purified from insect cells. Trimeric kindlin-3 in solution was denatured into monomeric state to give a band above 70k Da. Lane 2 indicates the kindlin-3 trimer crosslinked by DSSO. Crosslinked trimeric kindlin-3 in solution exhibited a band above 200k Da (labeled by red arrow). DSSO, disuccinimidyl sulfoxide. (TIF) [file pbio.3000755.s007.tif]

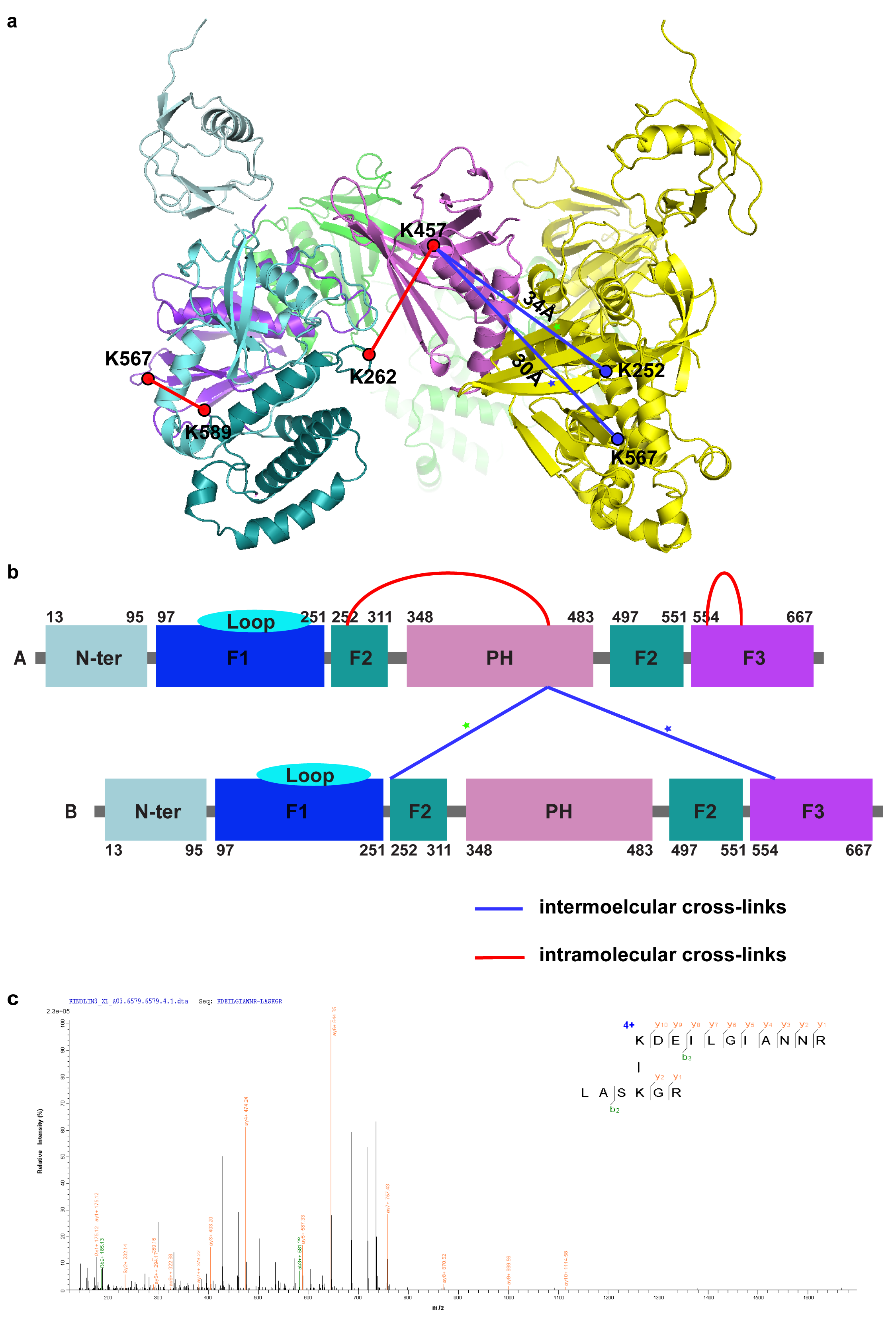

Supplement: S8 Fig — (a) Lysine–lysine intra- (red) and inter- (blue) molecules crosslinks were mapped onto the kindlin-3 crystal structure. The intermolecular crosslink marked with a blue asterisk is approximately 30 Å. It was identified with very high confidence. The intermolecular crosslink marked with a green asterisk is approximately 34 Å. It was identified with a relatively low confidence but also appears reasonable upon inspection of the structure. Besides, both 2 intramolecular crosslinks were identified with very high confidence. (b) Two domain organization of kindlins showing the identified lysine–lysine crosslinks. K567-K589 and K262-K457 are intramolecular crosslinks. K457-K567 and K252-K457 are intermolecular crosslinks. (c) Annotated MS/MS spectrum showing the b and y fragment ions of intermolecular crosslinked peptides K(252)DEILGIANNR-LASK(457)GR. DSSO, disuccinimidyl sulfoxide; MS, mass spectrometry. (TIF) [file pbio.3000755.s008.tif]

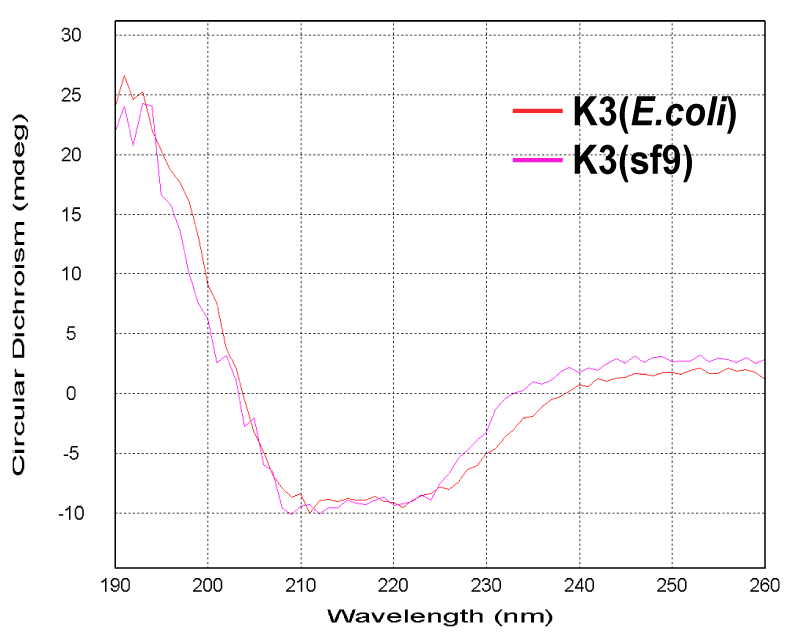

Supplement: S9 Fig — The far UV spectrum shows that E. coli–expressed kindlin-3 and Sf9-expressed kindlin-3 have very similar secondary and tertiary structures. E. coli, Escherichia coli; Sf9, Spodoptera frugiperda 9. (TIF) [file pbio.3000755.s009.tif]

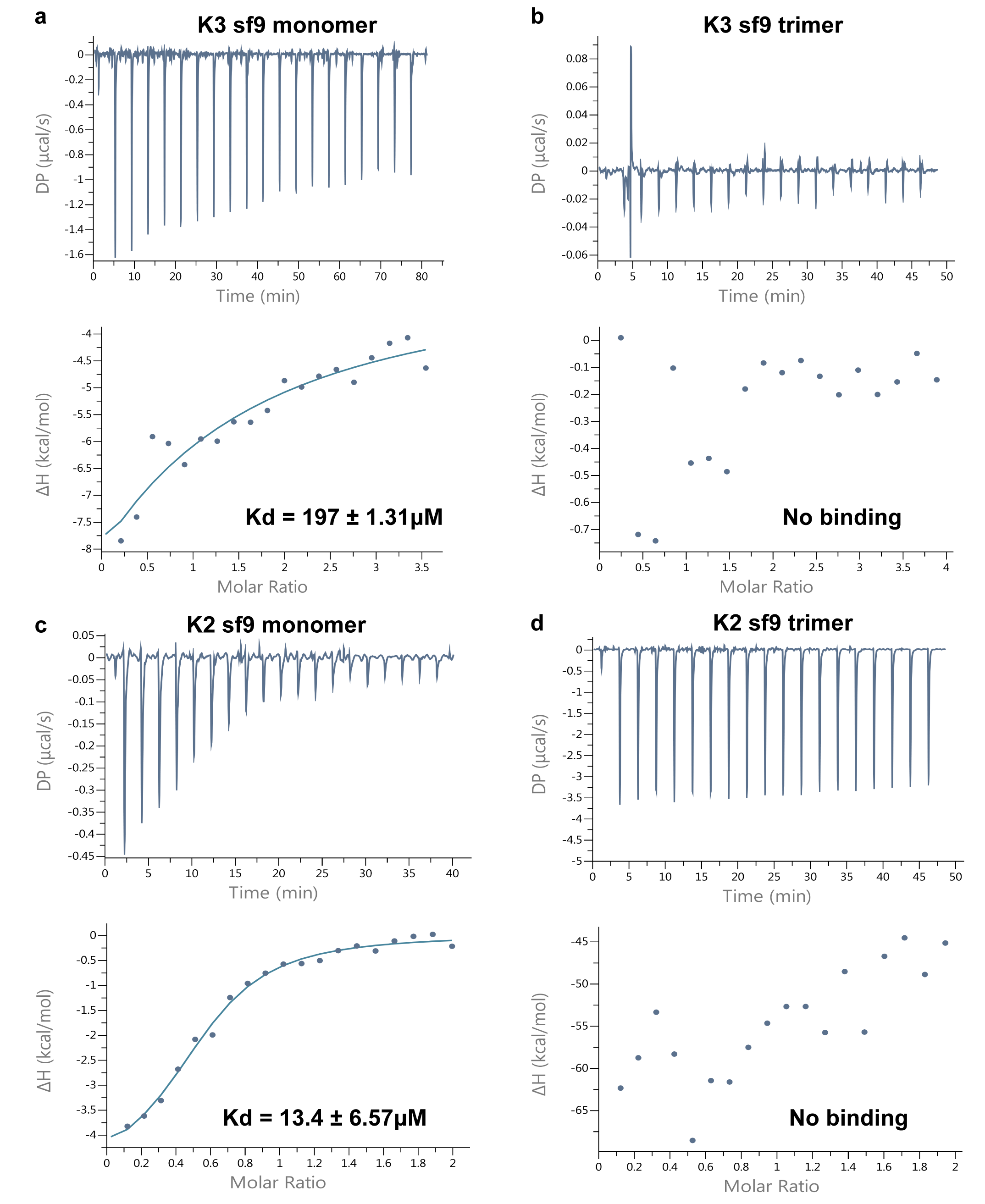

Supplement: S10 Fig — Note that in the individual figure, the upper panel shows binding isotherm, and the lower panel shows data-fitting curve. (a) Binding assay for kindlin-3 Sf9 monomer. The protein tested is the monomer form of native kindlin-3, which is expressed in Sf9 insect cells. ITC measurement demonstrated a moderate binding between integrin β1 tail and monomeric kindlin-3. (b) Binding assay for kindlin-3 Sf9 trimer. The protein used is native kindlin-3 trimer, which is expressed in Sf9 insect cells. In agreement with our structural data (Fig 3C), kindlin-3 trimer shows no binding to integrin β1 tail. (c) Binding assay for kindlin-2 Sf9 monomer. The protein used is native kindlin-2 monomer expressed in Sf9. Compared with monomeric kindlin-3, ITC measurement indicated a much stronger binding between integrin β1 tail and monomeric kindlin-2. (d) Binding assay for kindlin-2 Sf9 trimer. The protein used is native kindlin-2 trimer expressed in Sf9. In agreement with kindlin-3 trimer, kindlin-2 trimer shows no binding to integrin β1 tail. ITC, isothermal titration calorimetry; Sf9, Spodoptera frugiperda 9. (TIF) [file pbio.3000755.s010.tif]

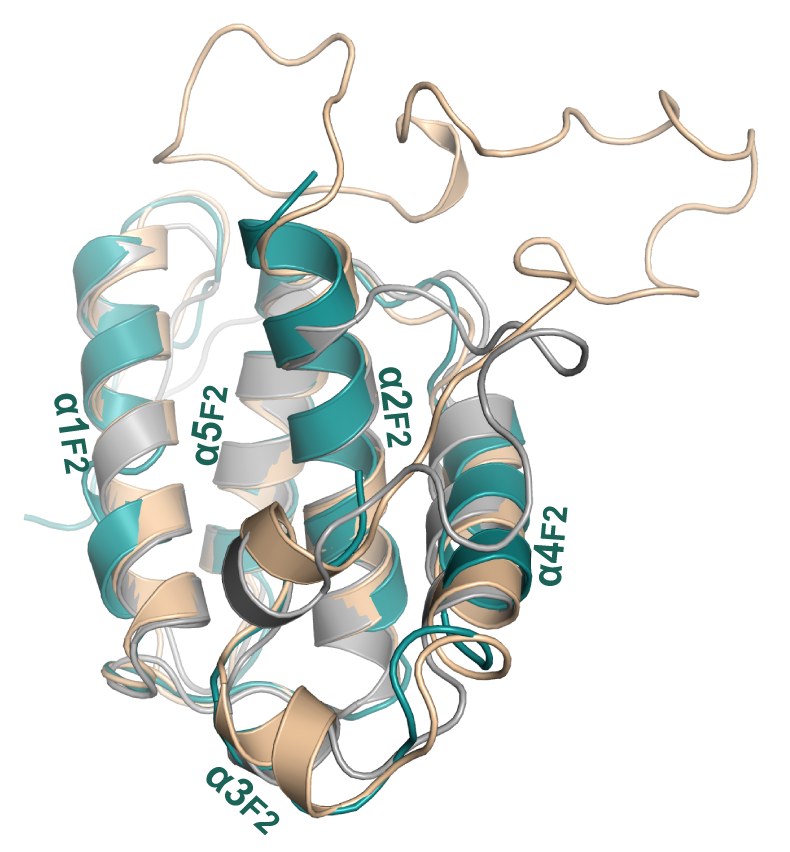

Supplement: S11 Fig — The F2 domains are from kindlin-3, kindlin-2, and talin, colored deep teal, yellow, gray, respectively. (TIF) [file pbio.3000755.s011.tif]
