# Supplementary material for: Structural basis of human full-length kindlin-3 homotrimer in an auto-inhibited state
Source: PLoS Biol. 2020 Jul 9;18(7):e3000755. doi: 10.1371/journal.pbio.3000755 (PMC7373317; doi:10.1371/journal.pbio.3000755)
Supplement: S1 Data — DSSO, disuccinimidyl sulfoxide; MS, mass spectrometry. (ZIP) [file pbio.3000755.s012.zip › S1_Data.pdf]

KINDLIN3_XL_A01.6318.6318.4.1.dta Seq: KDEILGIANNR-LASKGR

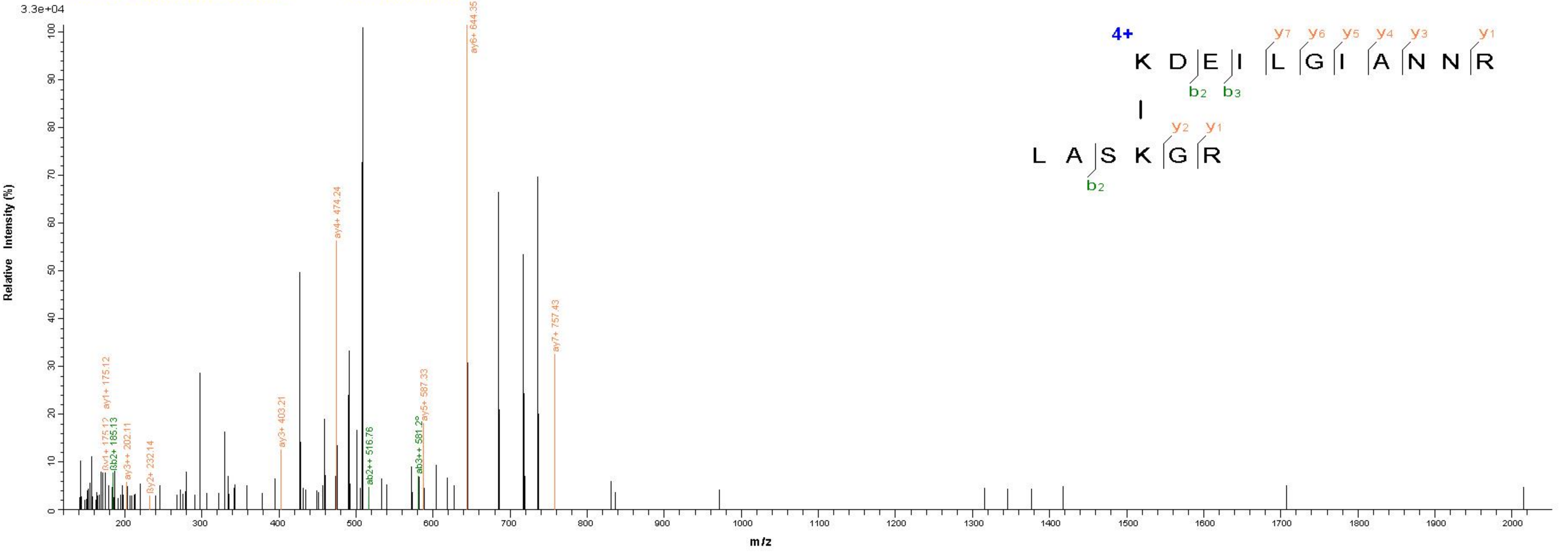

KINDLIN3_XL_A02.6523.6523.4.0.dta Seq: KDEILGIANNR-LASKGR

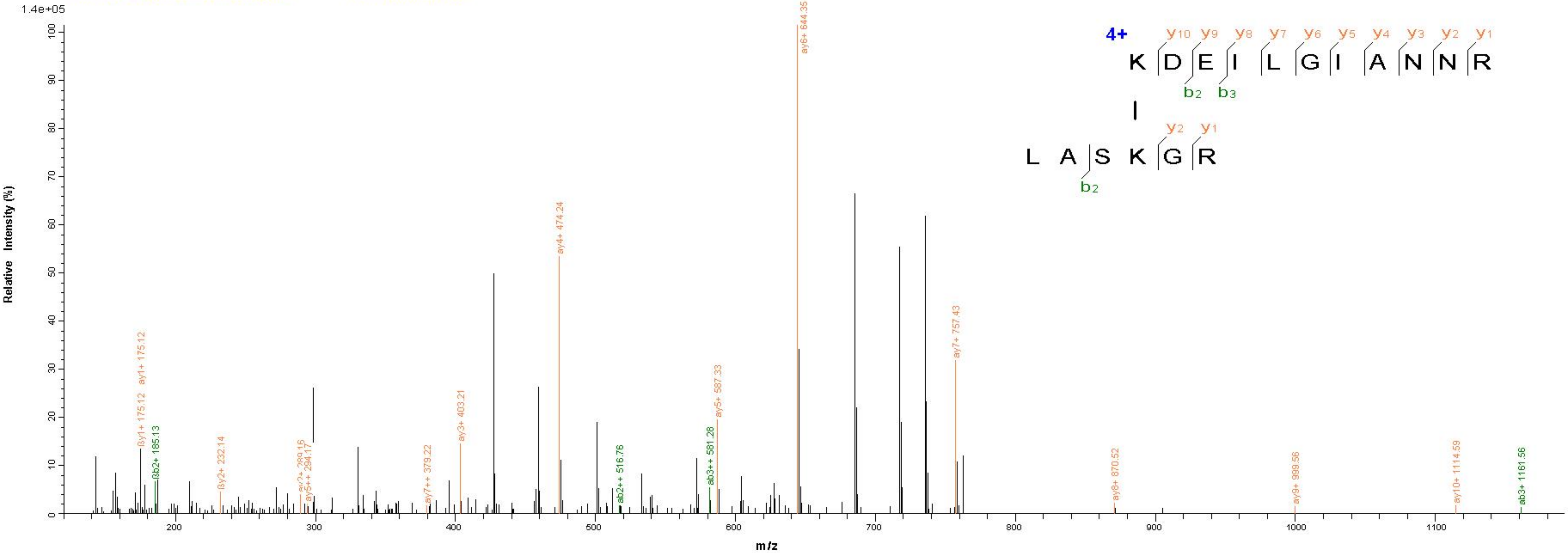

KINDLIN3_XL_A03.6536.6536.4.1.dta Seq: KDEILGIANNR-LASKGR

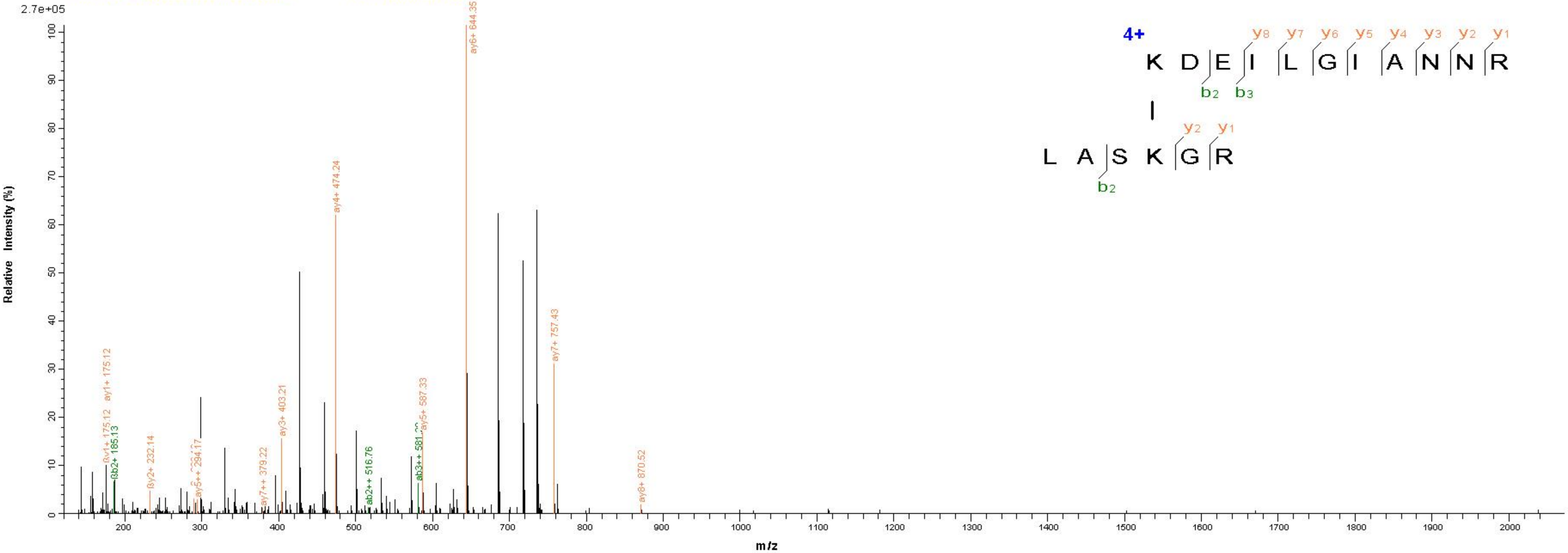

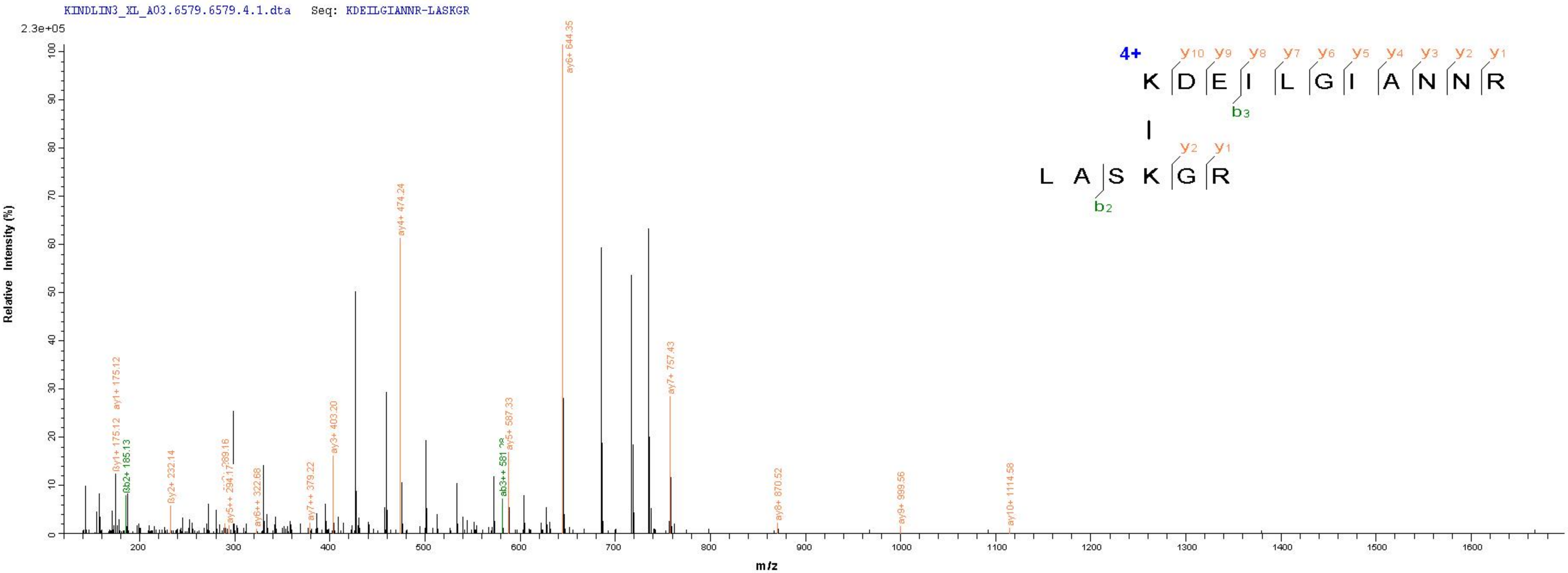
KINDLIN3_XL_A03.6579.6579.4.1.dta Seq: KDEILGIANNR-LASKGR
2.3e+05
Relative Intensity (%)
m/z
4+
K D E I L G I A N N R
y10 y9 y8 y7 y6 y5 y4 y3 y2 y1
b3
L A S K G R
y2 y1
b2
ay6+ 644.35
ay4+ 474.24
ay7+ 757.43
ay1+ 175.12
ßy1+ 175.12
ßb2+ 185.13
ßy2+ 232.14
ay3+ 403.20
ay5+ 587.33
ab3++ 581
ay5++ 294.17
ay6++ 322.68
ay7++ 379.22
ay8+ 870.52
ay9+ 999.56
ay10+ 1114.58

KINDLIN3_XL_B01.6522.6522.4.0.dta Seq: KDEILGIANNR-LASKGR

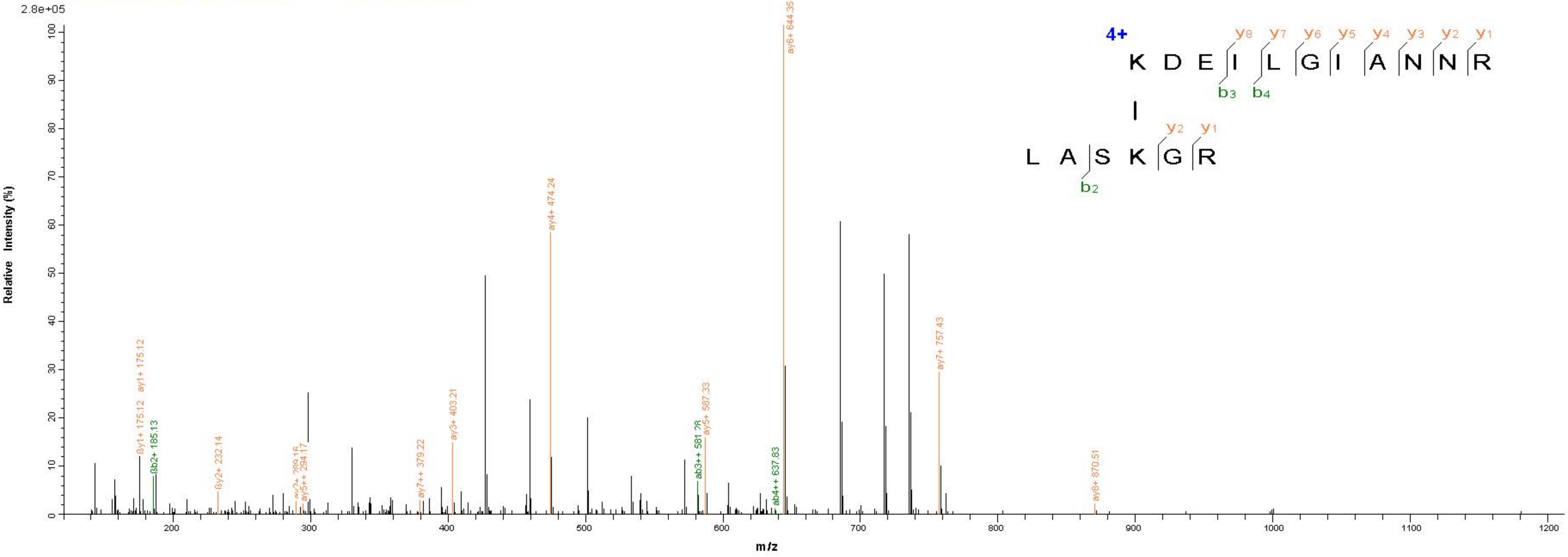

KINDLIN3_XL_B01.6559.6559.4.0.dta   Seq: KDEILGIANNR-LASKGR

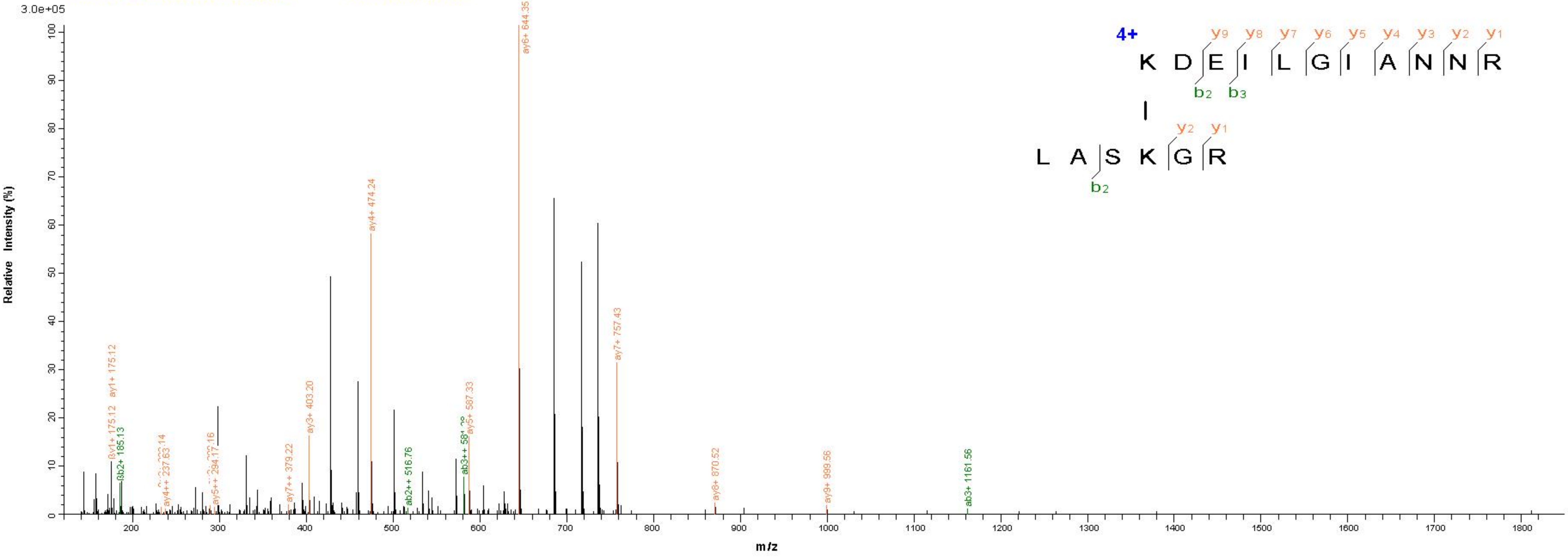

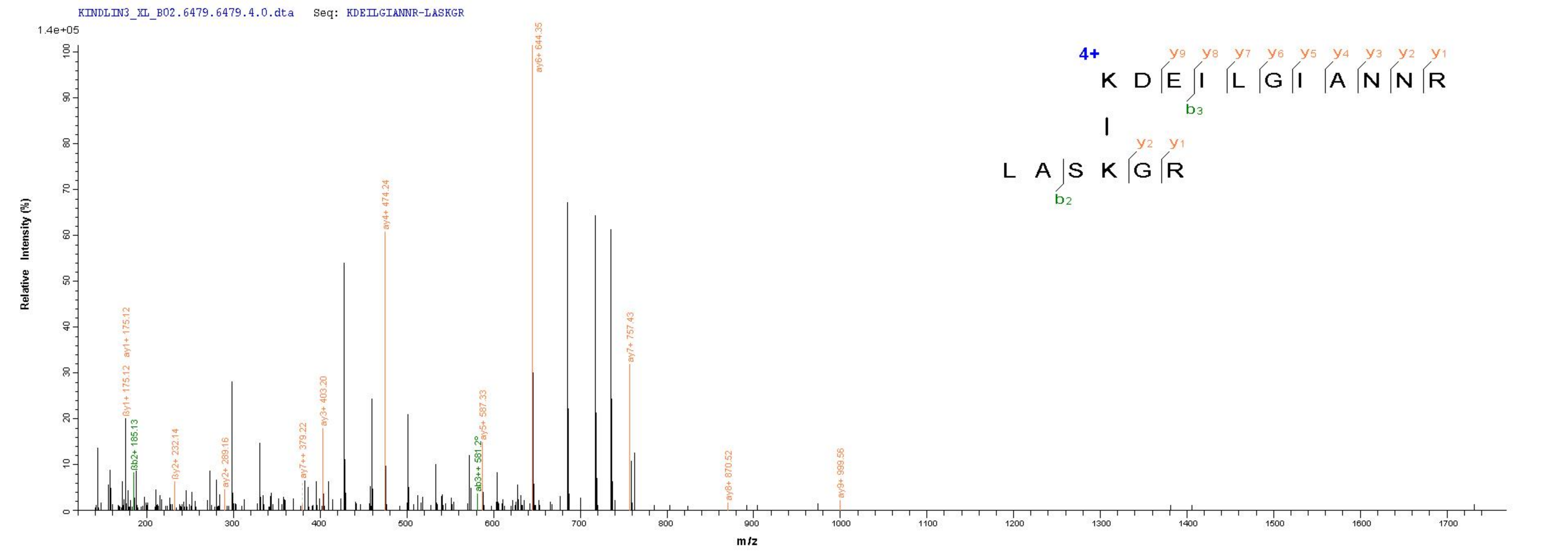
KINDLIN3_XL_B02.6479.6479.4.0.dta Seq: KDEILGIANNR-LASKGR
1.4e+05
Relative Intensity (%)
m/z
4+
K D E I L G I A N N R
L A S K G R
y9 y8 y7 y6 y5 y4 y3 y2 y1
b3
y2 y1
b2
ay6+ 644.35
ay4+ 474.24
ay7+ 757.43
ay1+ 175.12
ßy1+ 175.12
ßb2+ 185.13
ßy2+ 232.14
ay2+ 289.16
ay7++ 379.22
ay3+ 403.20
ab3++ 581.2
ay5+ 587.33
ay8+ 870.52
ay9+ 999.56

KINDLIN3_XL_B02.6522.6522.4.0.dta Seq: KDEILGIANNR-LASKGR

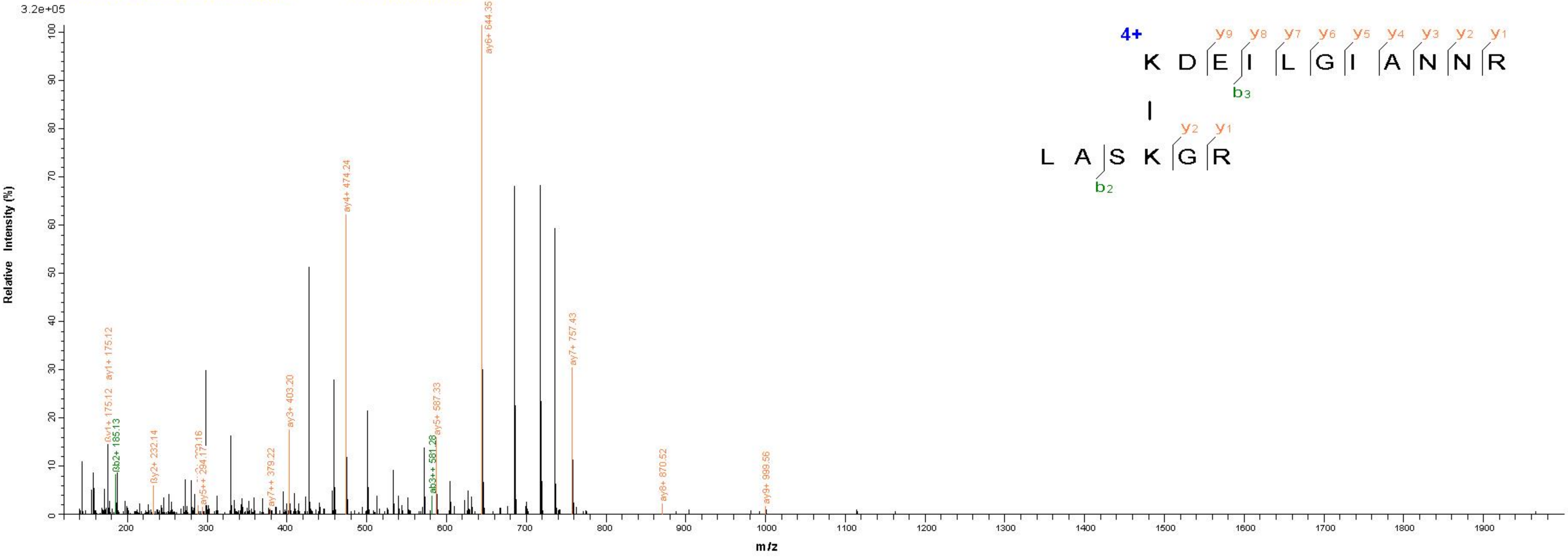

KINDLIN3_XL_B03.6494.6494.4.0.dta Seq: KDEILGIANNR-LASKGR

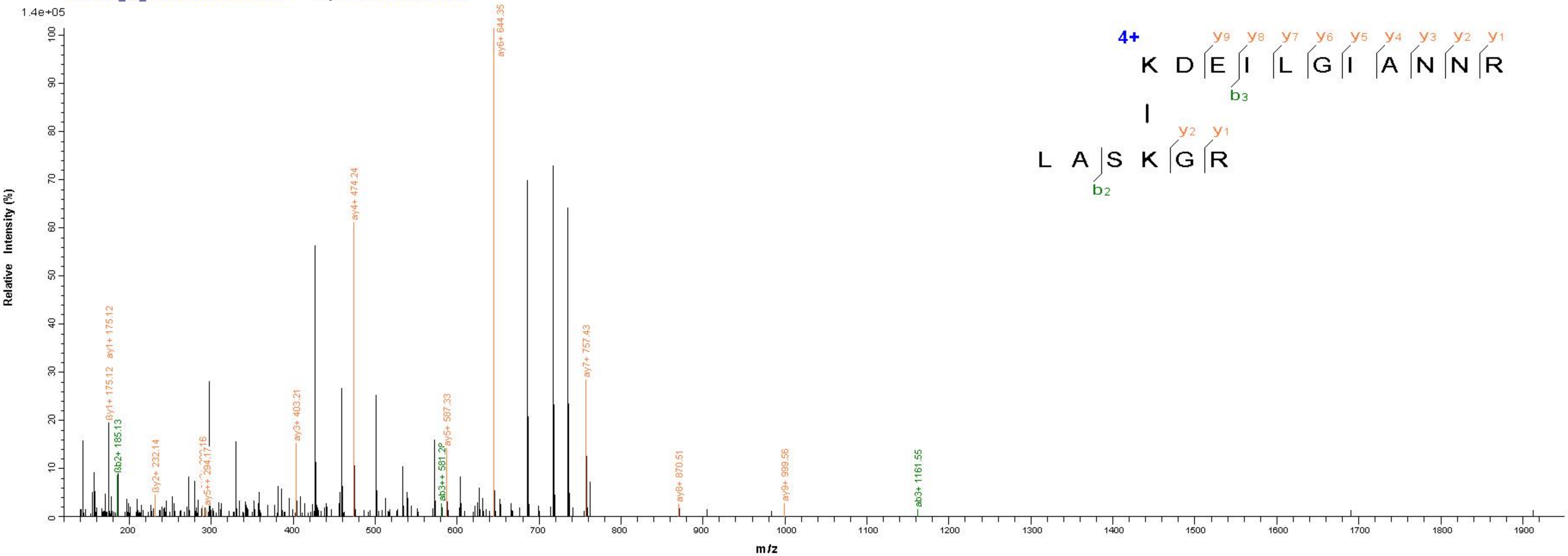

KINDLIN3_XL_B03.6537.6537.4.0.dta Seq: KDEILGIANNR-LASKGR

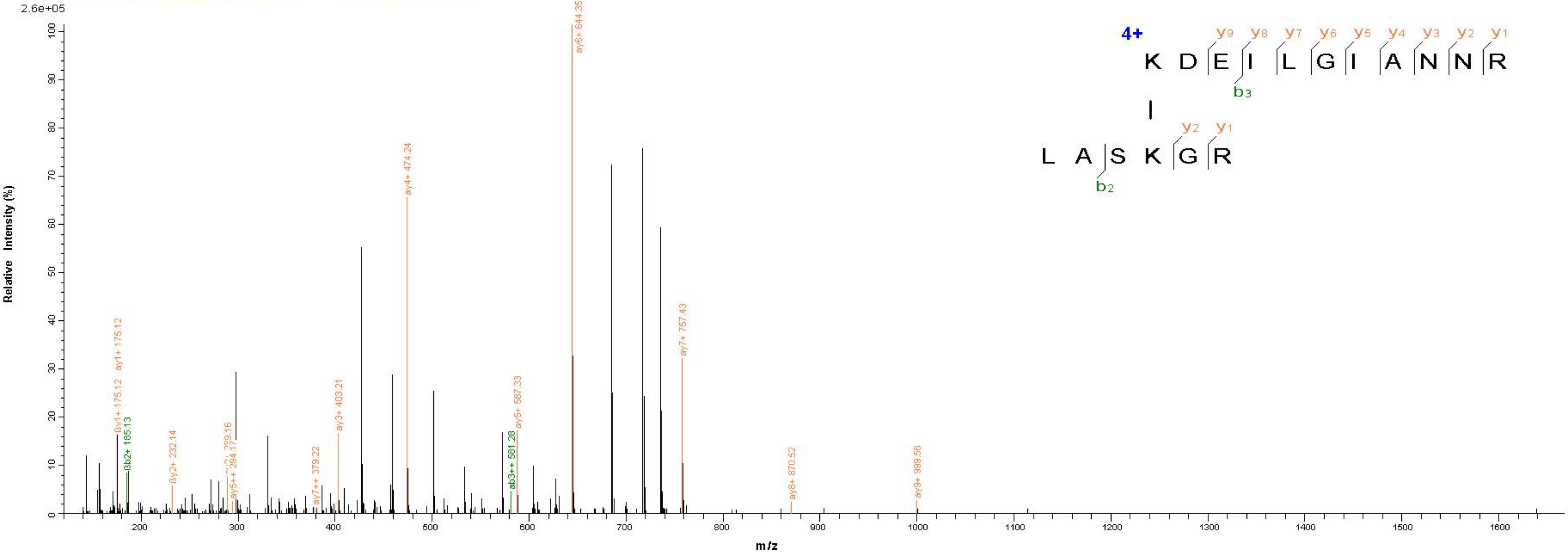

KINDLIN3_XL_A02.13418.13418.4.0.dta Seq: IDLAVGDVVKTWR-KDEILGIANNR

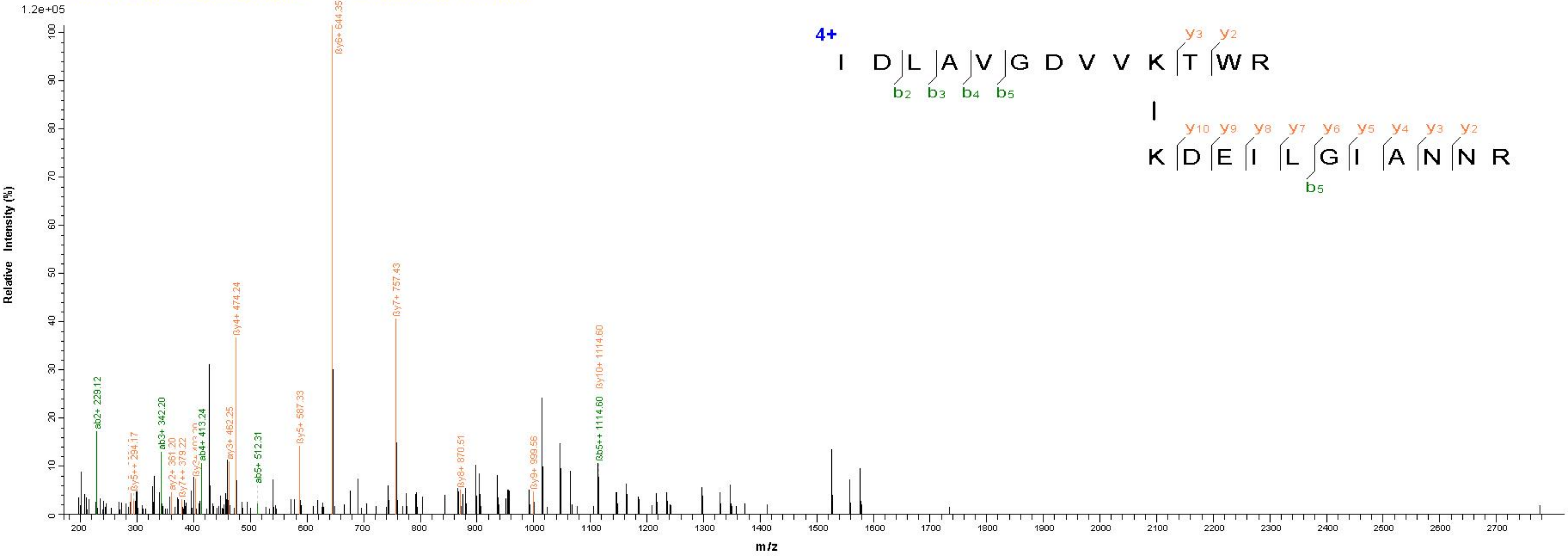

KINDLIN3_XL_A02.13441.13441.3.0.dta Seq: IDLAVGDVVKTWR-KDEILGIANNR

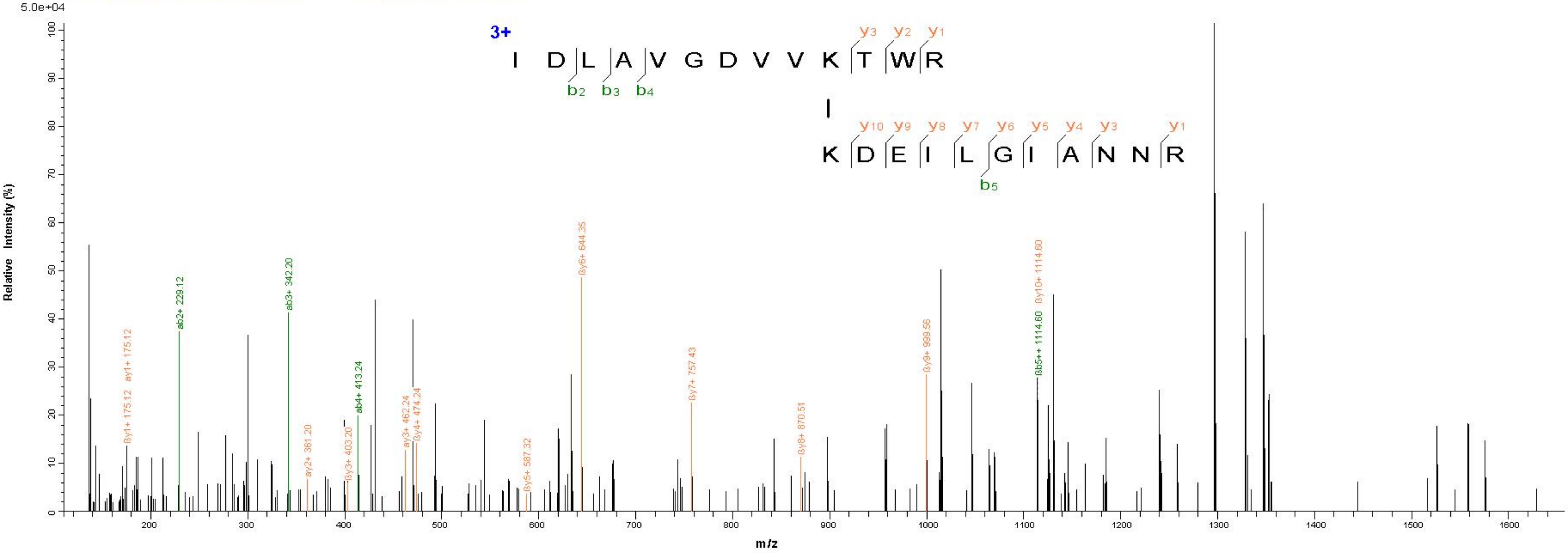

KINDLIN3_XL_A02.13442.13442.3.0.dta Seq: IDLAVGDVVKTWR-KDEILGIANNR

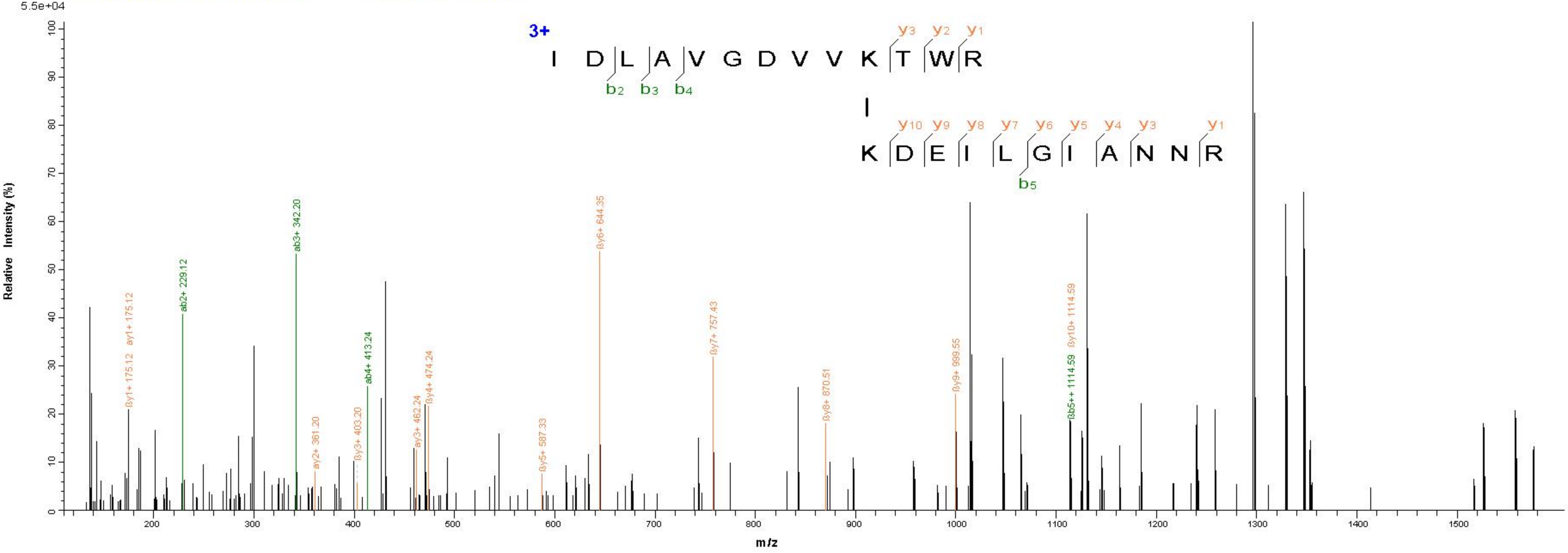

KINDLIN3_XL_A02.13443.13443.3.0.dta   Seq: IDLAVGDVVKTWR-KDEILGIANNR

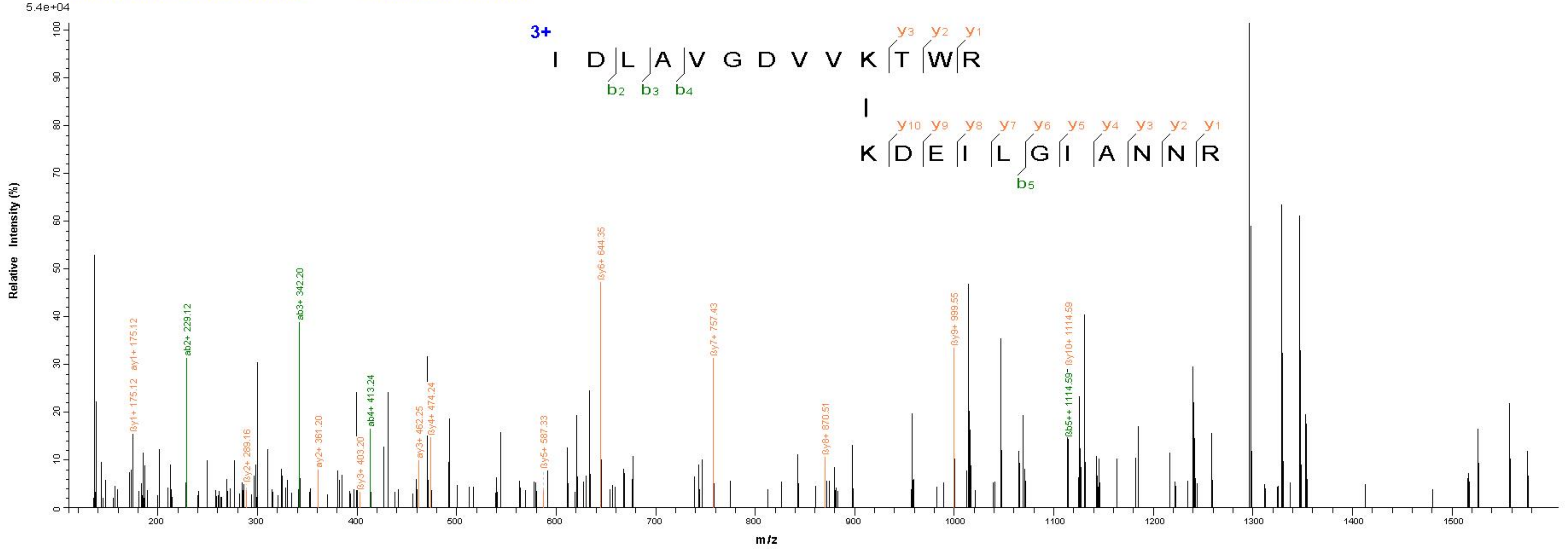

KINDLIN3_XL_A02.13445.13445.3.0.dta Seq: IDLAVGDVVKTWR-KDEILGIANNR

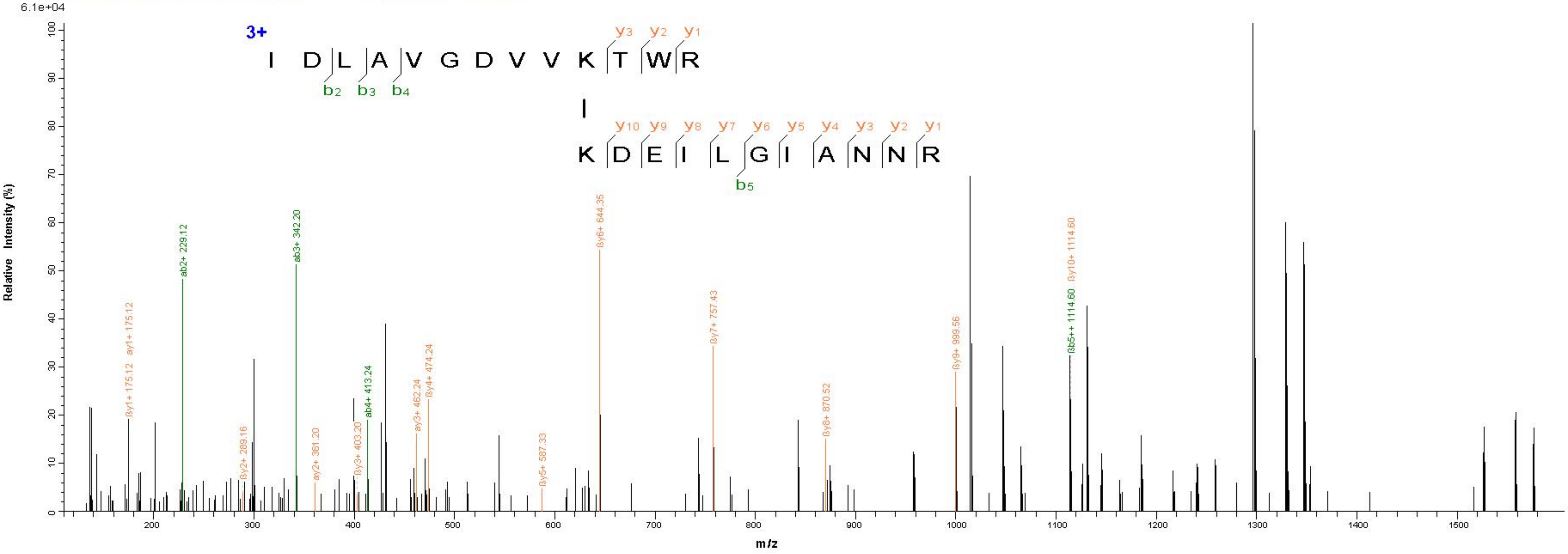

KINDLIN3_XL_A02.13451.13451.3.0.dta Seq: IDLAVGDVVKTWR-KDEILGIANNR

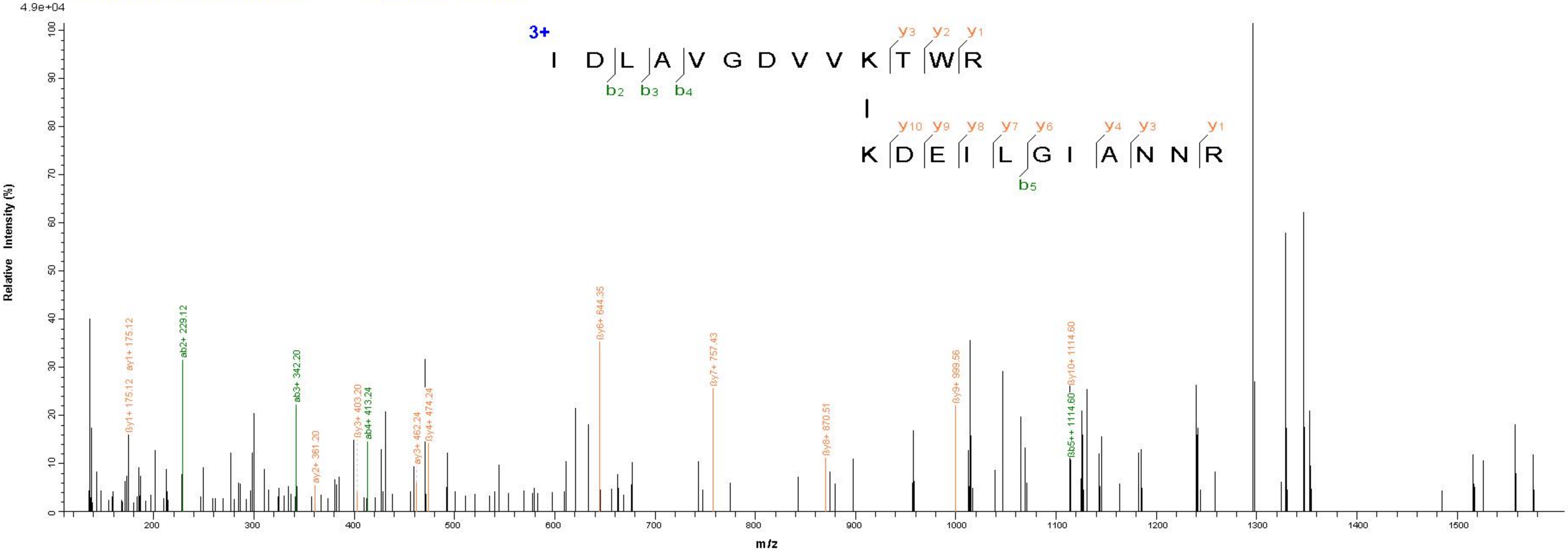

KINDLIN3_XL_A02.13452.13452.3.0.dta Seq: IDLAVGDVVKTWR-KDEILGIANNR

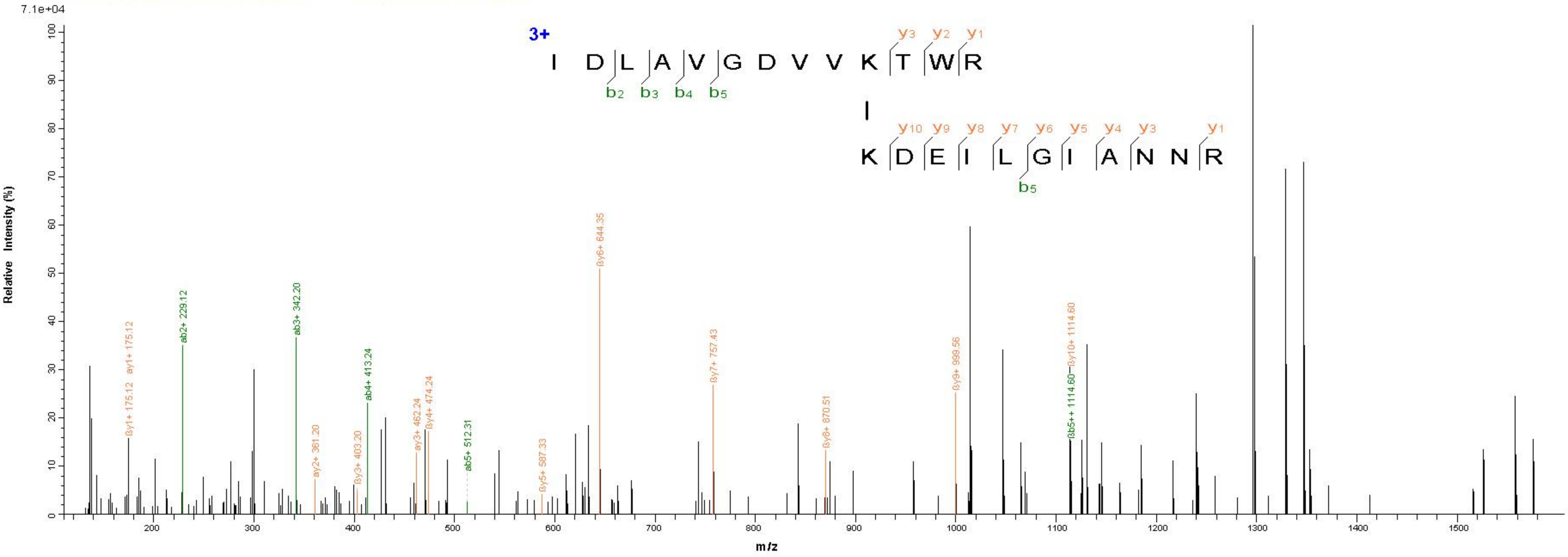

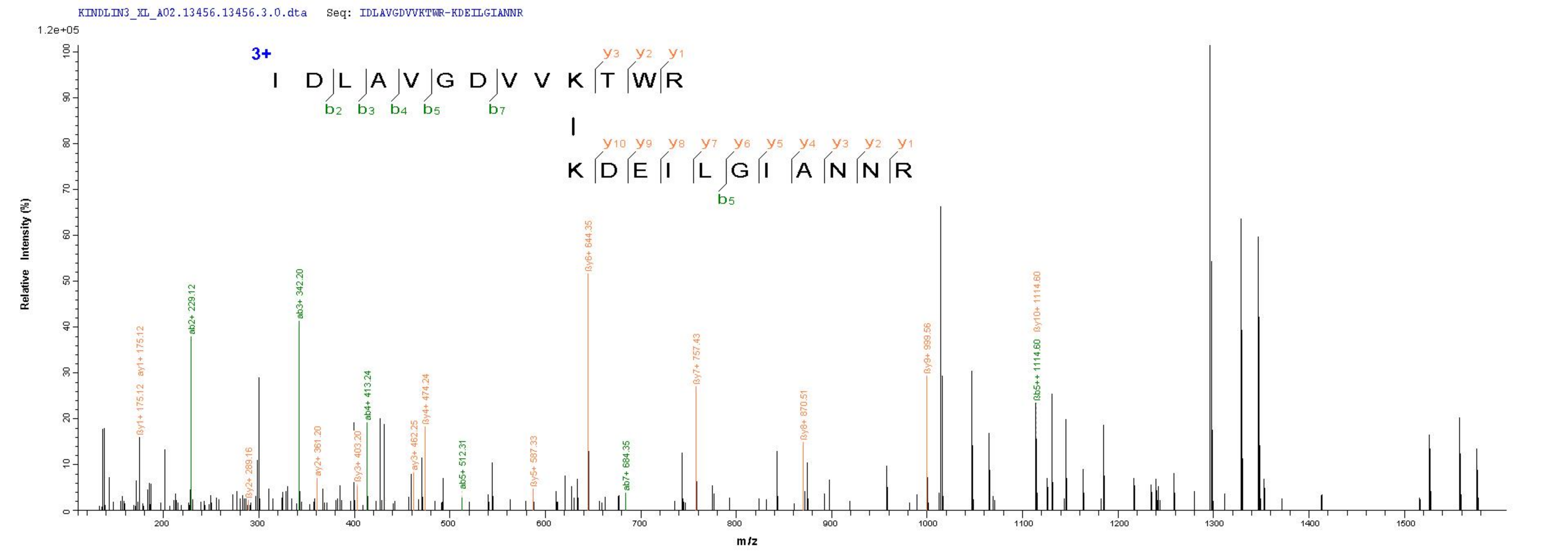
KINDLIN3_XL_A02.13456.13456.3.0.dta Seq: IDLAVGDVVKTWR-KDEILGIANNR
1.2e+05
3+
I D L A V G D V V K T W R
y3 y2 y1
b2 b3 b4 b5 b7
K D E I L G I A N N R
y10 y9 y8 y7 y6 y5 y4 y3 y2 y1
b5
Relative Intensity (%)
m/z
ßy1+ 175.12
ay1+ 175.12
ab2+ 229.12
ßy2+ 289.16
ab3+ 342.20
ay2+ 361.20
ßy3+ 403.20
ab4+ 413.24
ay3+ 462.25
ßy4+ 474.24
ab5+ 512.31
ßy5+ 587.33
ßy6+ 644.35
ab7+ 684.35
ßy7+ 757.43
ßy8+ 870.51
ßy9+ 999.56
ßb5++ 1114.60
ßy10+ 1114.60

KINDLIN3_XL_A02.13482.13482.4.0.dta Seq: IDLAVGDVVKTWR-KDEILGIANNR

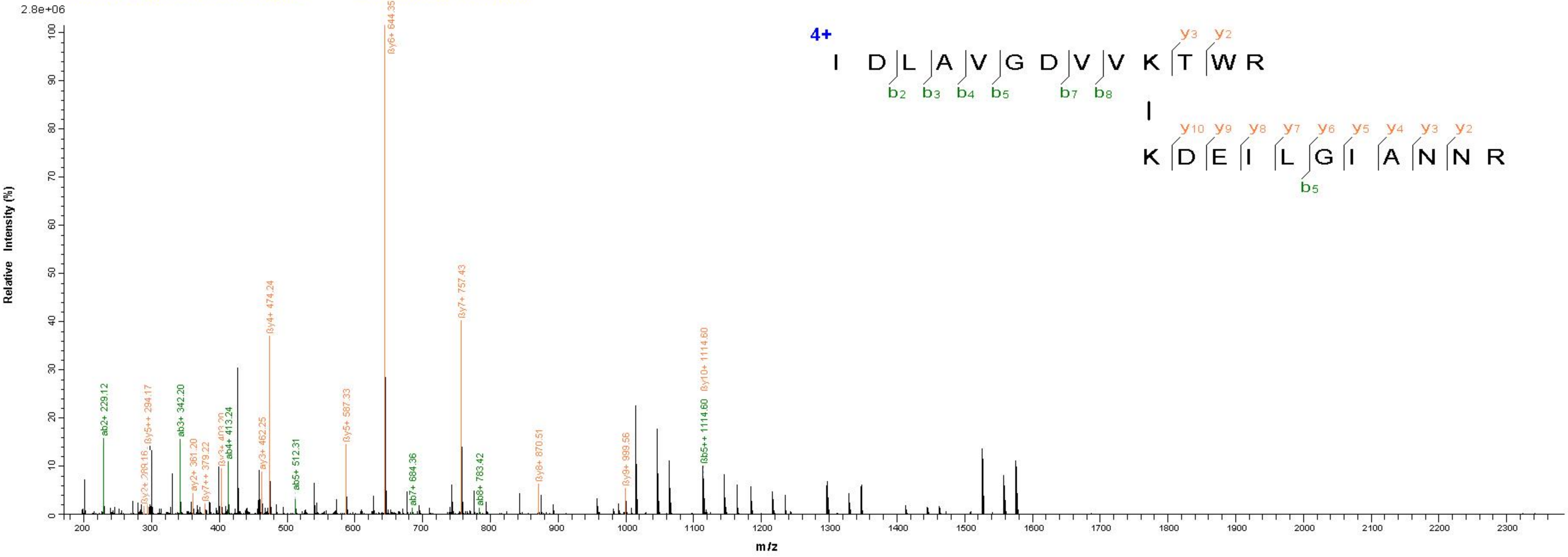

KINDLIN3_XL_A02.13549.13549.4.0.dta   Seq: IDLAVGDVVKTWR-KDEILGIANNR

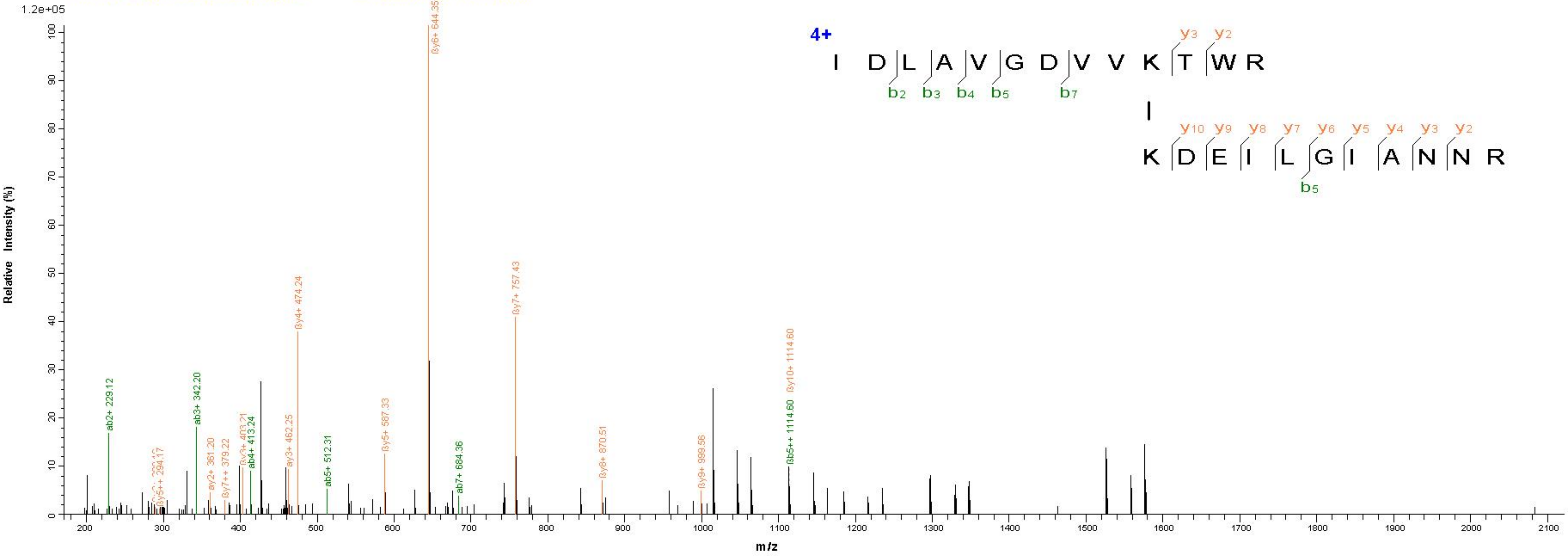

KINDLIN3_XL_A02.13614.13614.4.0.dta Seq: IDLAVGDVVKTWR-KDEILGIANNR

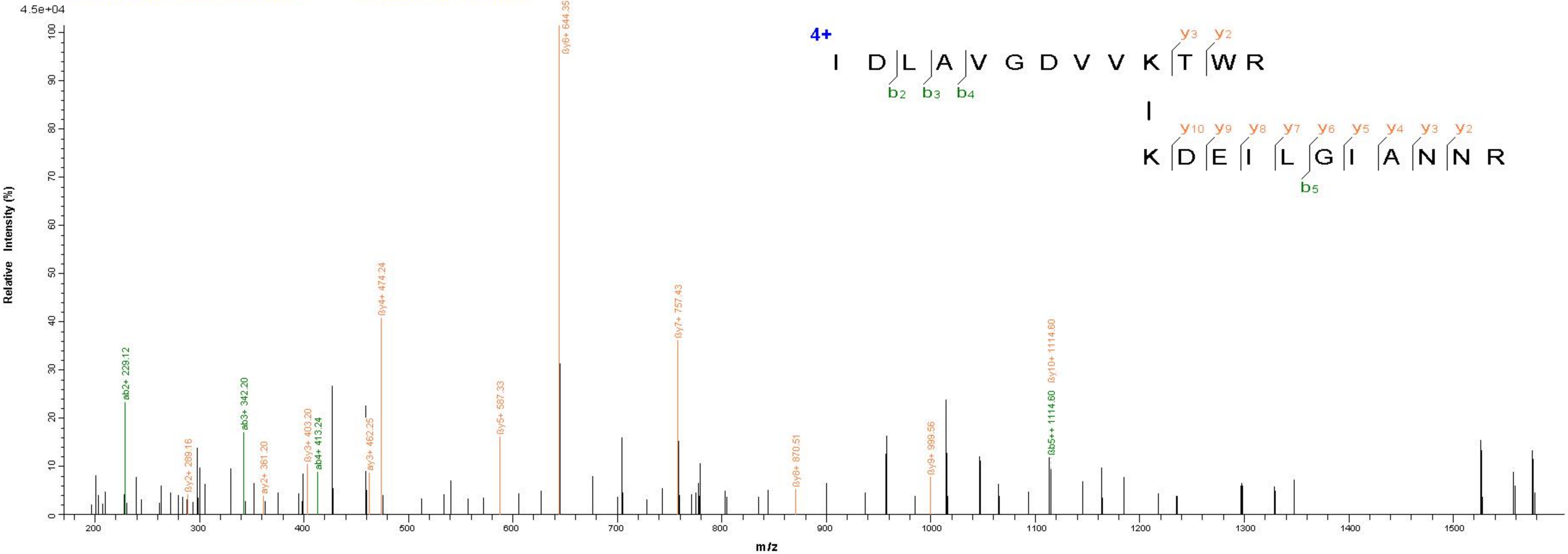

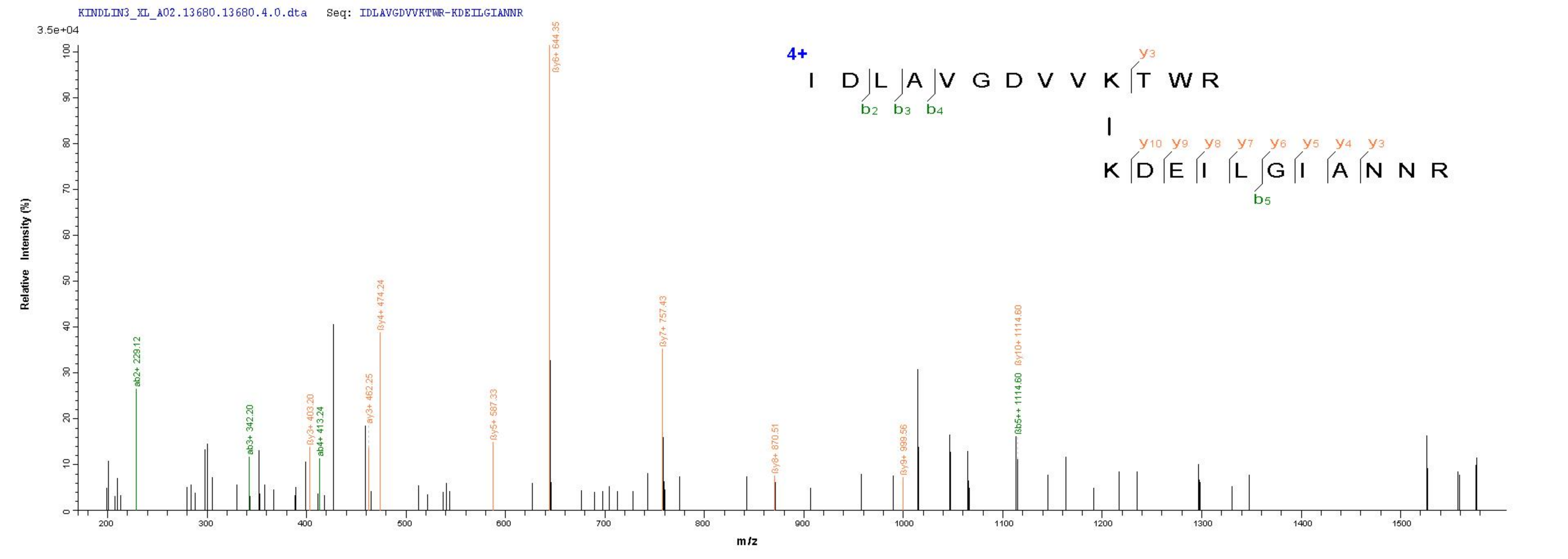

KINDLIN3_XL_A02.13680.13680.4.0.dta Seq: IDLAVGDVVKTWR-KDEILGIANNR
3.5e+04
Relative Intensity (%)
m/z
4+
I D L A V G D V V K T W R
K D E I L G I A N N R
ab2+ 229.12
ab3+ 342.20
ßy3+ 403.20
ab4+ 413.24
ay3+ 462.25
ßy4+ 474.24
ßy5+ 587.33
ßy6+ 644.35
ßy7+ 757.43
ßy8+ 870.51
ßy9+ 999.56
ßb5++ 1114.60
ßy10+ 1114.60

KINDLIN3_XL_A02.13792.13792.4.0.dta Seq: IDLAVGDVVKTWR-KDEILGIANNR Mod: 25,_deamidated[NQ]

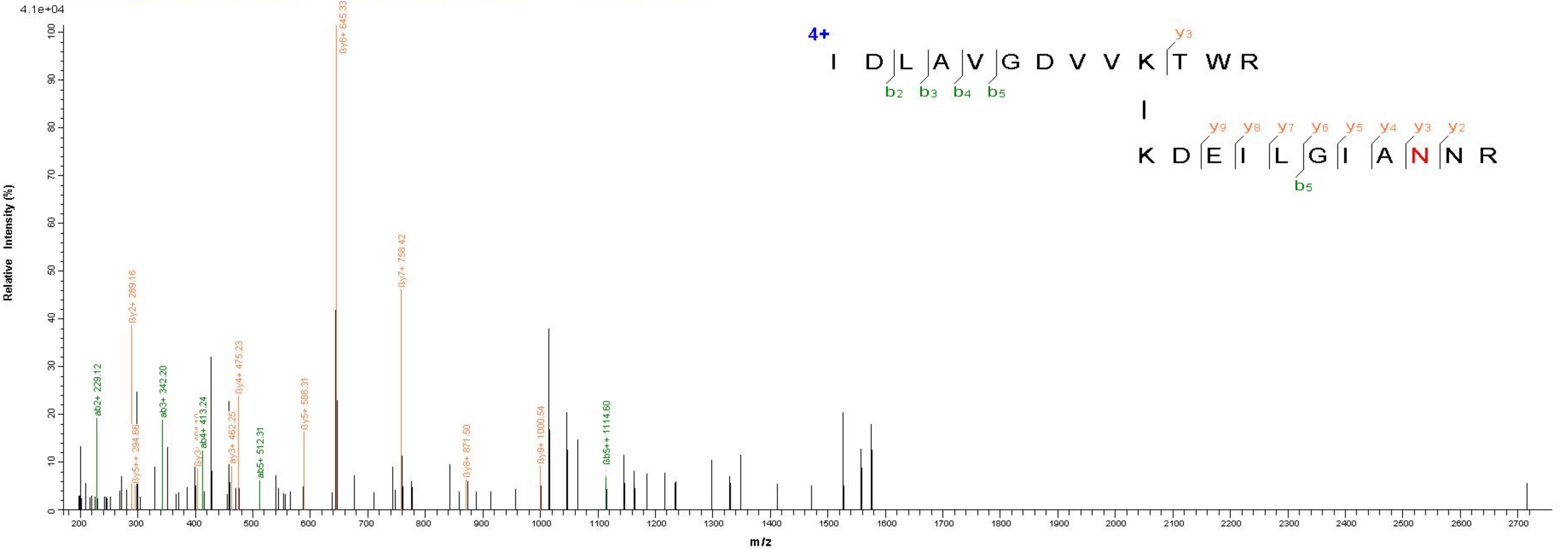

KINDLIN3_XL_A02.13872.13872.4.0.dta Seq: IDLAVGDVVKTWR-KDEILGIANNR Mod: 25,_deamidated[NQ]

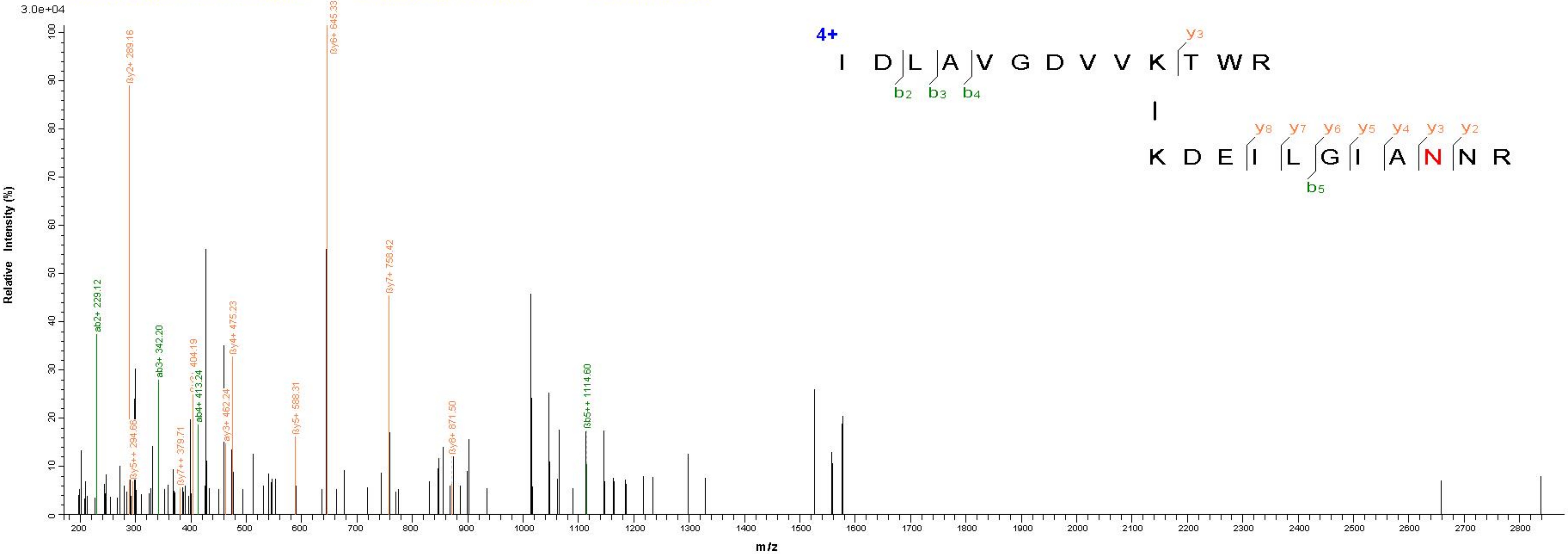

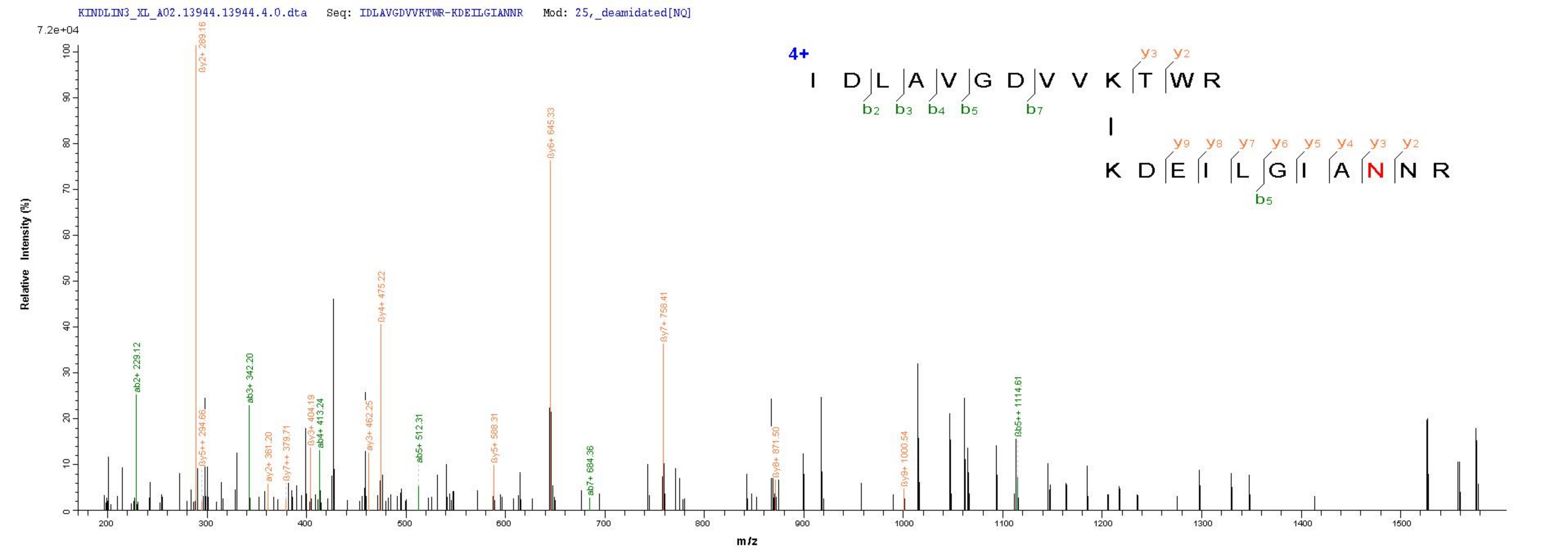

KINDLIN3_XL_A02.13944.13944.4.0.dta Seq: IDLAVGDVVKTWR-KDEILGIANNR Mod: 25,_deamidated[NQ]
7.2e+04
Relative Intensity (%)
m/z
4+
I D L A V G D V V K T W R
K D E I L G I A N N R
ab2+ 229.12
ßy2+ 289.16
ßy5++ 294.66
ab3+ 342.20
ay2+ 361.20
ßy7++ 379.71
ßy3+ 404.19
ab4+ 413.24
ay3+ 462.25
ßy4+ 475.22
ab5+ 512.31
ßy5+ 588.31
ßy6+ 645.33
ab7+ 684.36
ßy7+ 758.41
ßy8+ 871.50
ßy9+ 1000.54
ßb5++ 1114.61

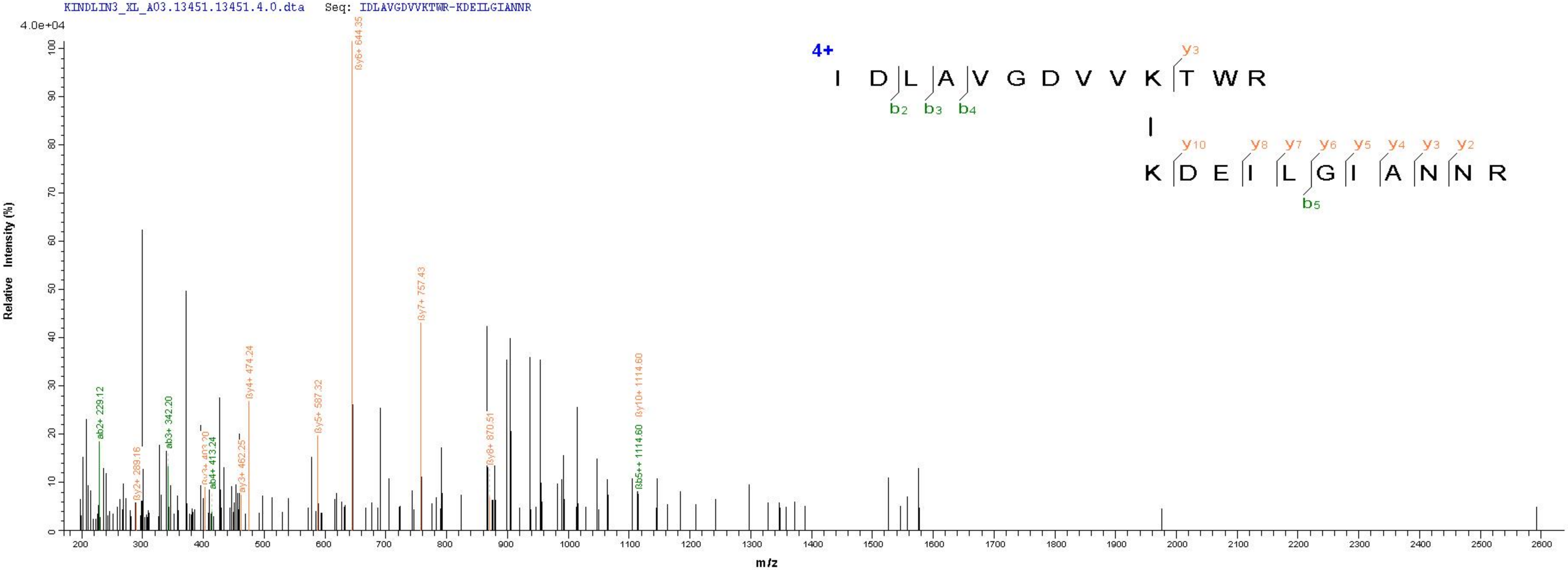

KINDLIN3_XL_A03.13451.13451.4.0.dta Seq: IDLAVGDVVKTWR-KDEILGIANNR
4.0e+04
Relative Intensity (%)
m/z
4+
I D L A V G D V V K T W R
K D E I L G I A N N R
ßy6+ 644.35
ßy7+ 757.43
ßy4+ 474.24
ßy5+ 587.32
ßy10+ 1114.60
ßb5++ 1114.60
ßy8+ 870.51
ab2+ 229.12
ab3+ 342.20
ab4+ 413.24
ay3+ 462.25
ßy2+ 289.16

KINDLIN3_XL_A03.13460.13460.4.0.dta Seq: IDLAVGDVVKTWR-KDEILGIANNR

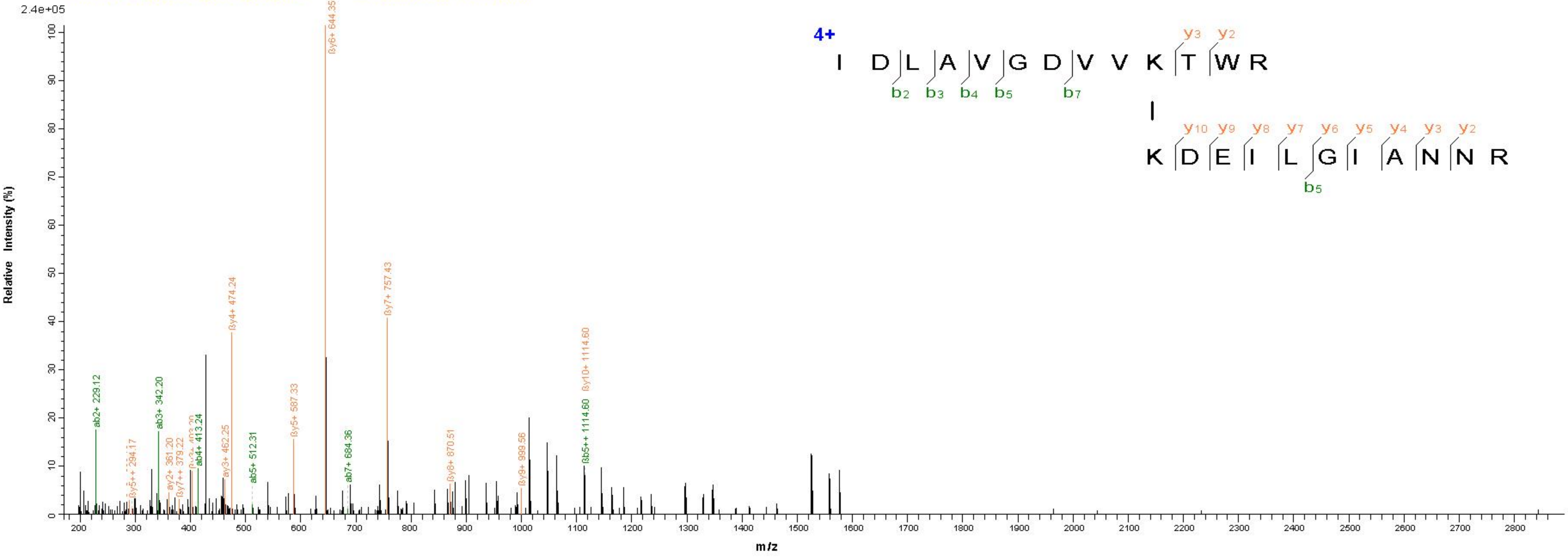

KINDLIN3_XL_A03.13479.13479.3.0.dta Seq: IDLAVGDVVKTWR-KDEILGIANNR

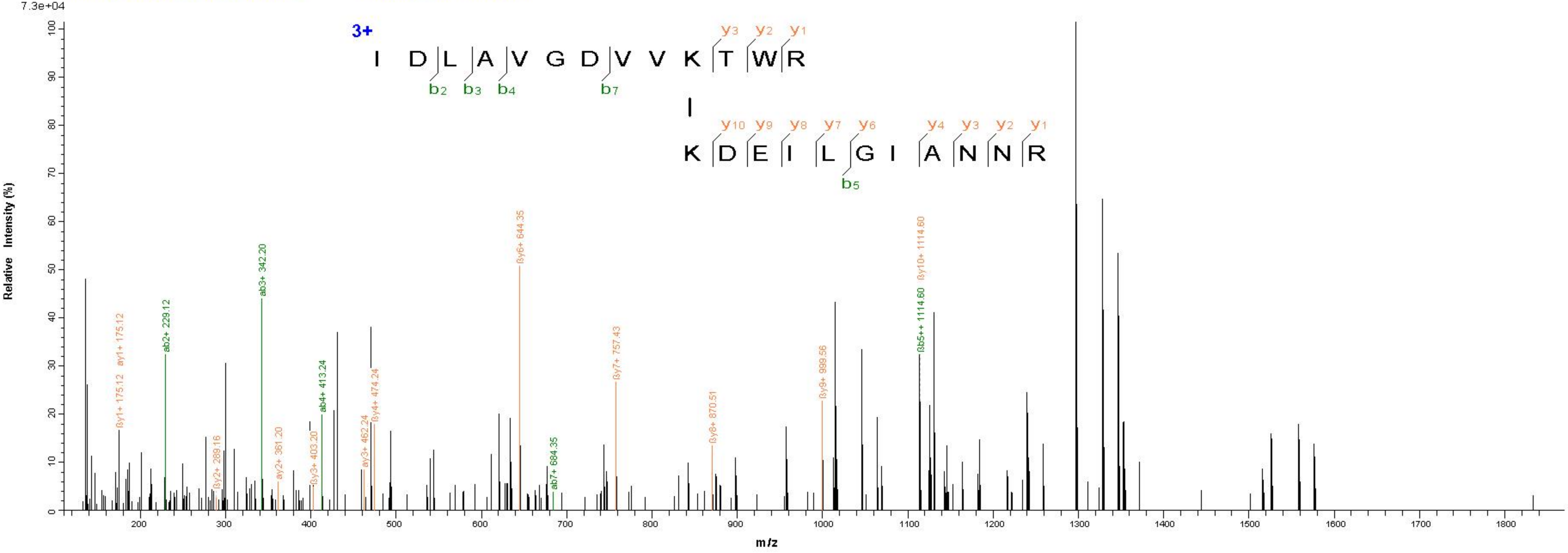

KINDLIN3_XL_A03.13480.13480.3.0.dta Seq: IDLAVGDVVKTWR-KDEILGIANNR

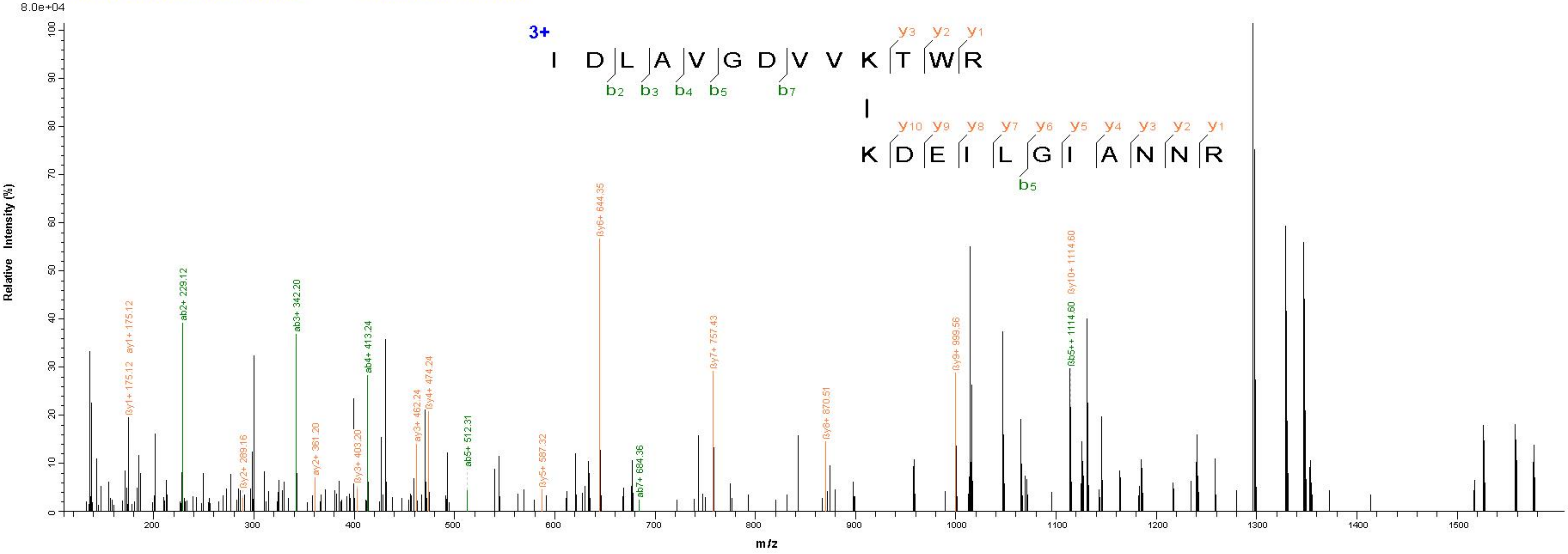

KINDLIN3_XL_A03.13491.13491.3.0.dta Seq: IDLAVGDVVKTWR-KDEILGIANNR

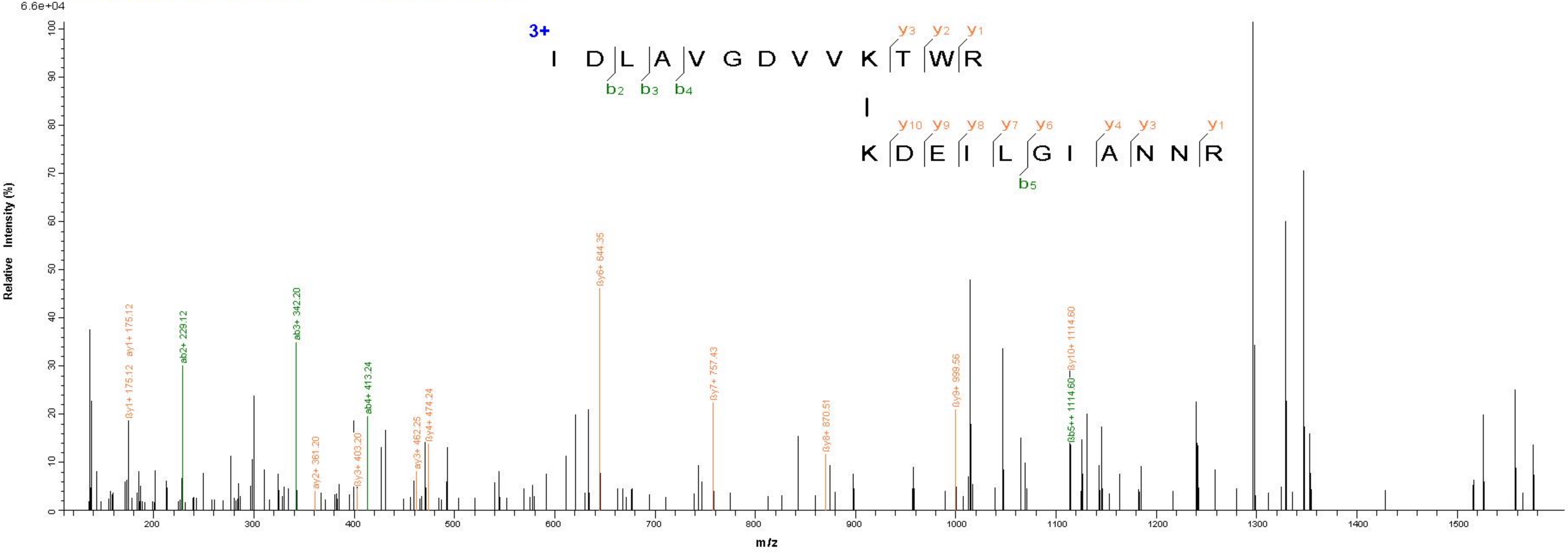

KINDLIN3_XL_A03.13492.13492.3.0.dta Seq: IDLAVGDVVKTWR-KDEILGIANNR

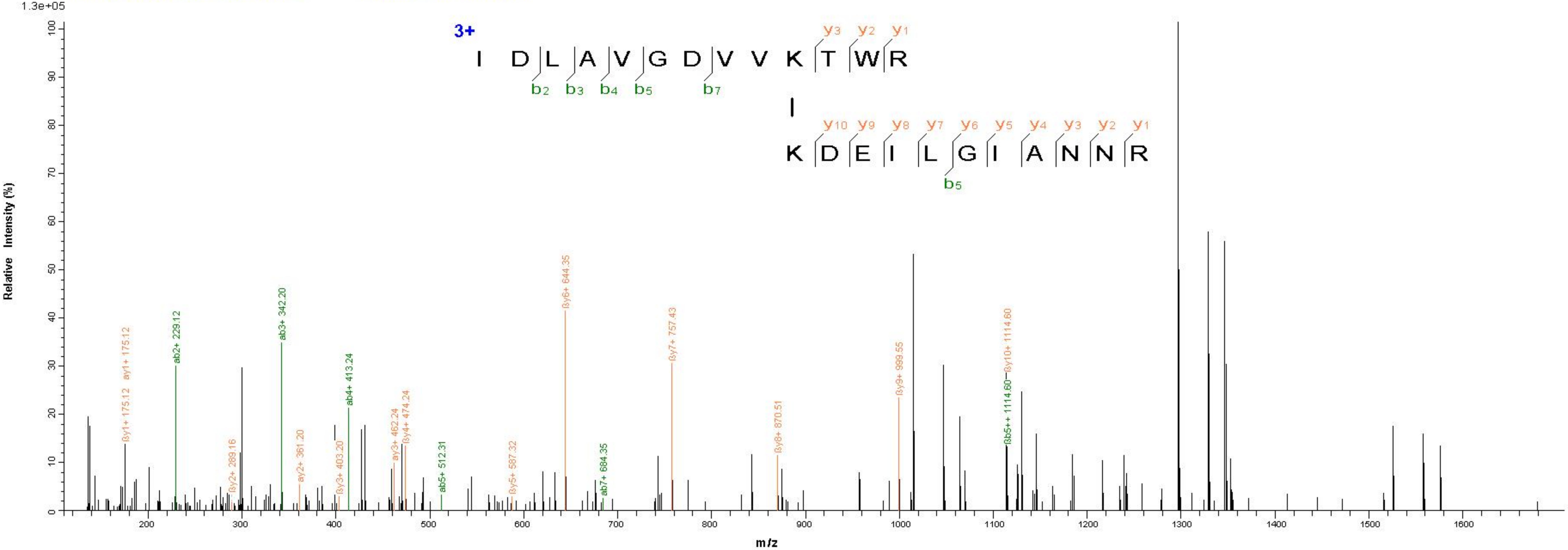

KINDLIN3_XL_A03.13497.13497.4.0.dta Seq: IDLAVGDVVKTWR-KDEILGIANNR

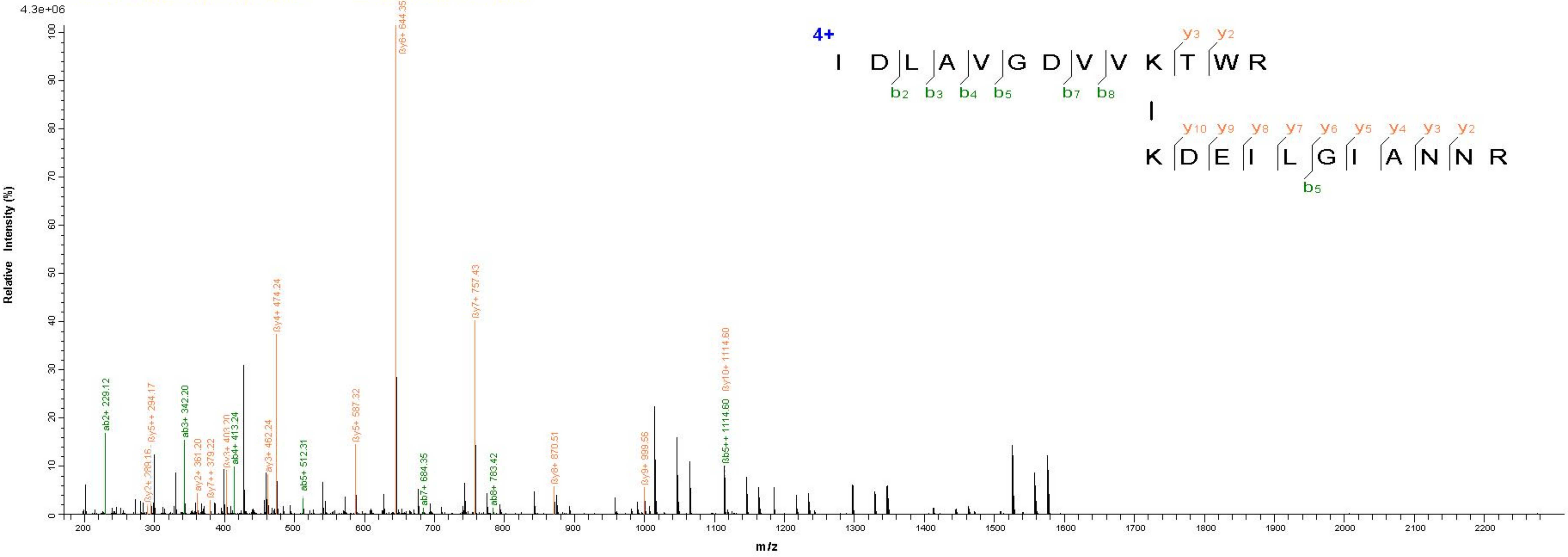

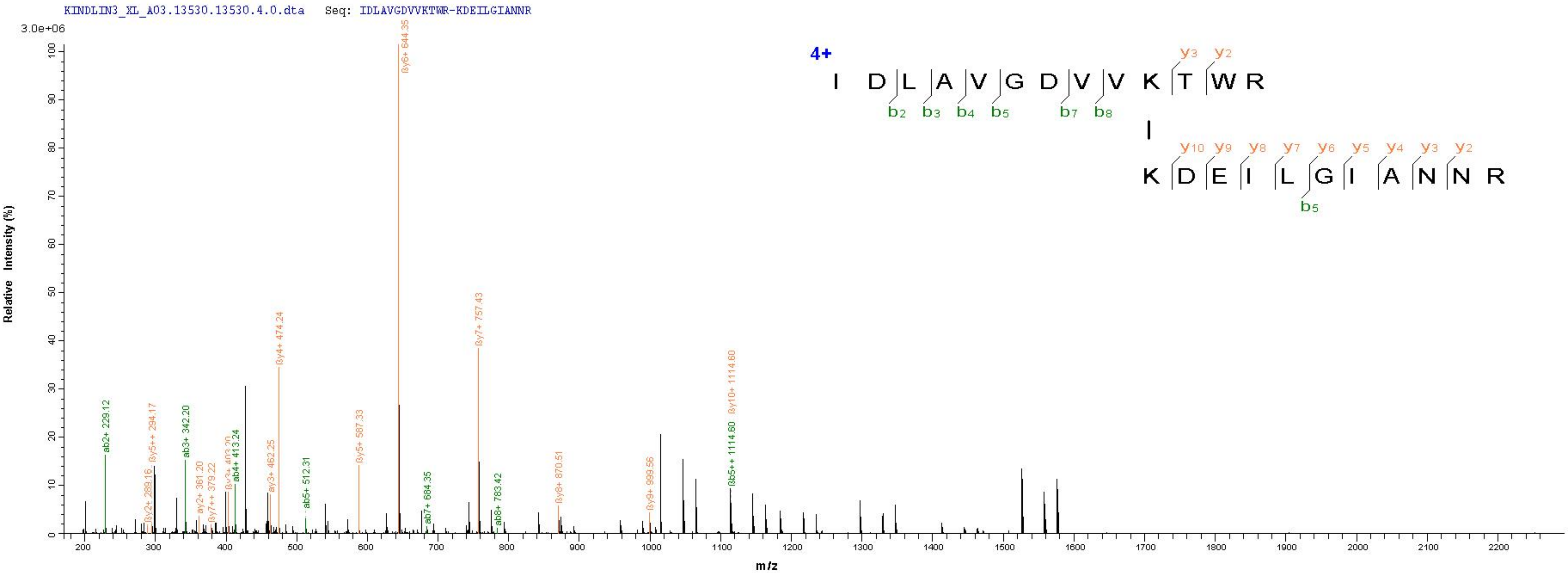
KINDLIN3_XL_A03.13530.13530.4.0.dta Seq: IDLAVGDVVKTWR-KDEILGIANNR
3.0e+06
Relative Intensity (%)
m/z
4+
I D L A V G D V V K T W R
K D E I L G I A N N R
ab2+ 229.12
ßy2+ 289.16
ßy5++ 294.17
ab3+ 342.20
ay2+ 361.20
ßy7++ 379.22
ab4+ 413.24
ay3+ 462.25
ßy4+ 474.24
ab5+ 512.31
ßy5+ 587.33
ßy6+ 644.35
ab7+ 684.35
ßy7+ 757.43
ab8+ 783.42
ßy8+ 870.51
ßy9+ 999.56
ßb5++ 1114.60
ßy10+ 1114.60

KINDLIN3_XL_A03.13563.13563.4.0.dta   Seq: IDLAVGDVVKTWR-KDEILGIANNR

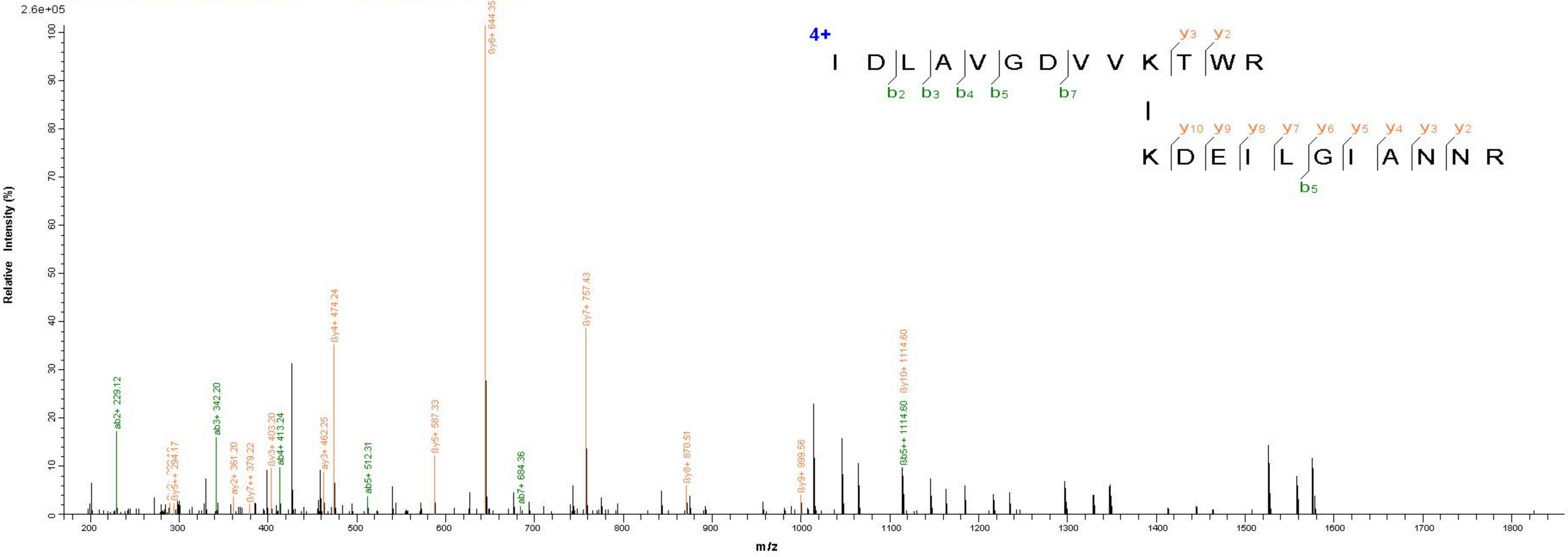

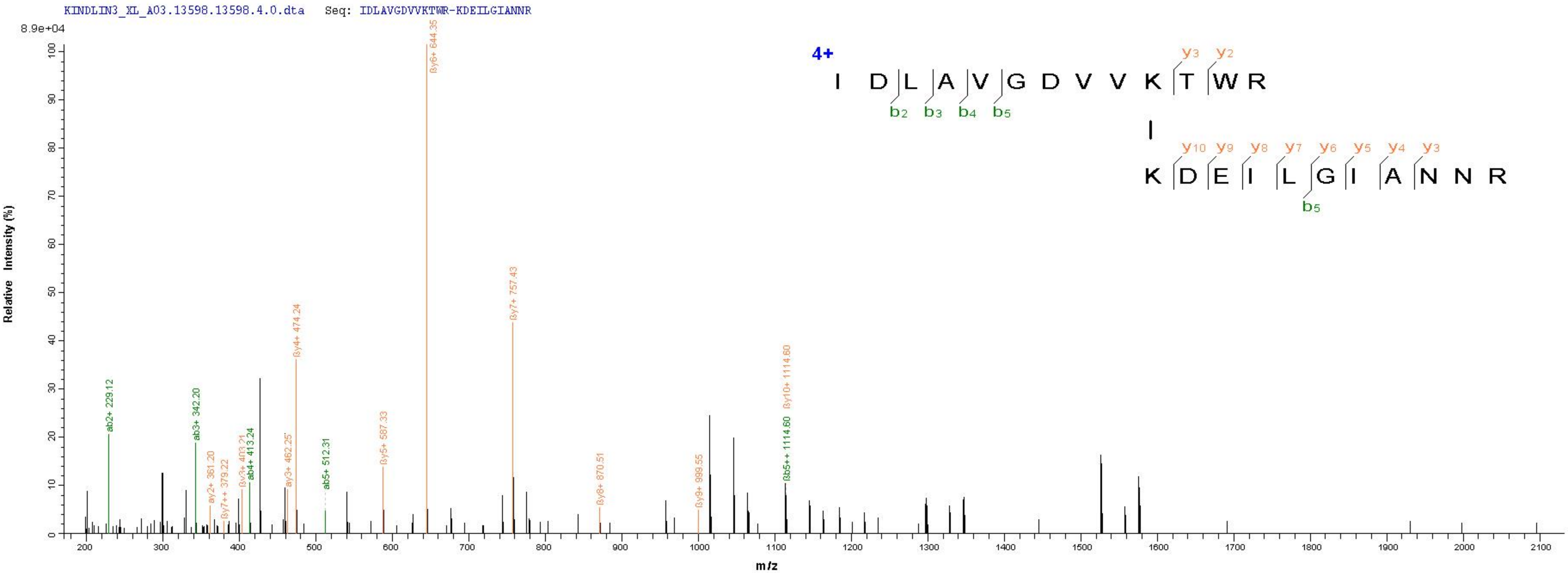

KINDLIN3_XL_A03.13598.13598.4.0.dta Seq: IDLAVGDVVKTWR-KDEILGIANNR
8.9e+04
4+
I D L A V G D V V K T W R
K D E I L G I A N N R
ab2+ 229.12
ab3+ 342.20
ay2+ 361.20
βy7++ 379.22
βy3+ 403.21
ab4+ 413.24
ay3+ 462.25
βy4+ 474.24
ab5+ 512.31
βy5+ 587.33
βy6+ 644.35
βy7+ 757.43
βy8+ 870.51
βy9+ 999.55
βb5++ 1114.60
βy10+ 1114.60
Relative Intensity (%)
m/z

KINDLIN3_XL_A03.13647.13647.4.1.dta   Seq: IDLAVGDVVKTWR-KDEILGIANNR

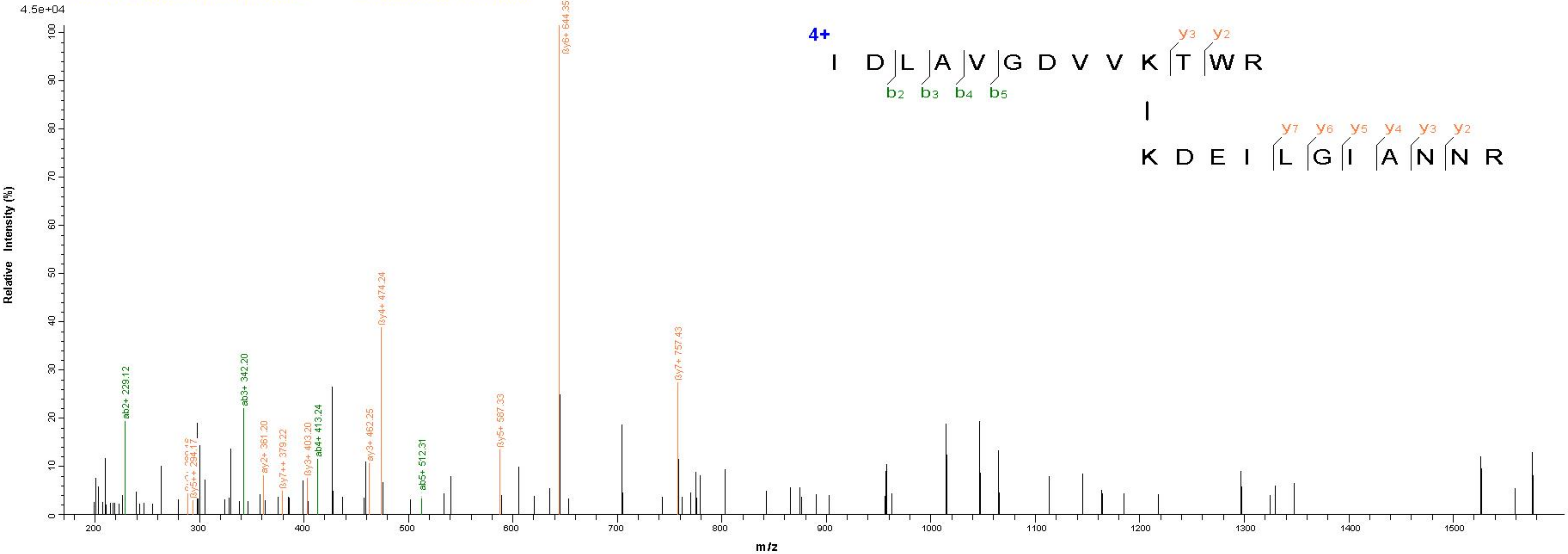

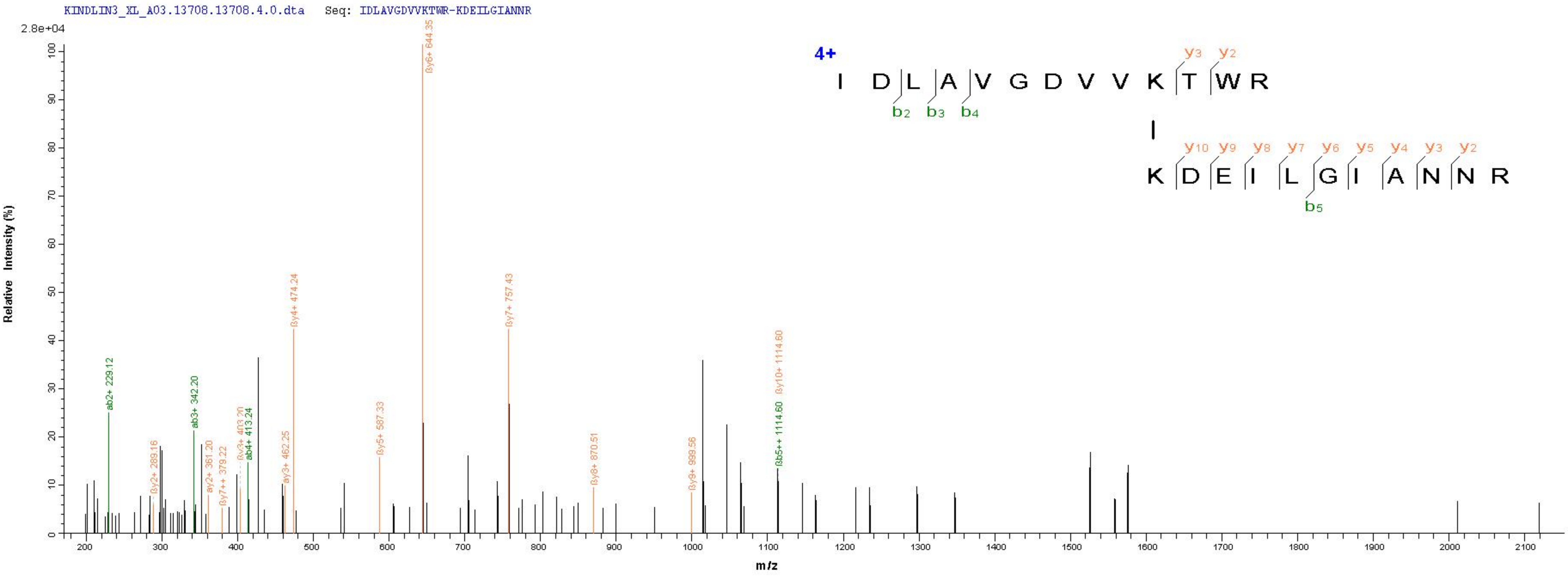
KINDLIN3_XL_A03.13708.13708.4.0.dta Seq: IDLAVGDVVKTWR-KDEILGIANNR
2.8e+04
Relative Intensity (%)
m/z
4+
I D L A V G D V V K T W R
y3 y2
b2 b3 b4
K D E I L G I A N N R
y10 y9 y8 y7 y6 y5 y4 y3 y2
b5
ab2+ 229.12
ßy2+ 289.16
ab3+ 342.20
ay2+ 361.20
ßy7++ 379.22
ßy3+ 403.20
ab4+ 413.24
ay3+ 462.25
ßy4+ 474.24
ßy5+ 587.33
ßy6+ 644.35
ßy7+ 757.43
ßy8+ 870.51
ßy9+ 999.56
ßb5++ 1114.60
ßy10+ 1114.60

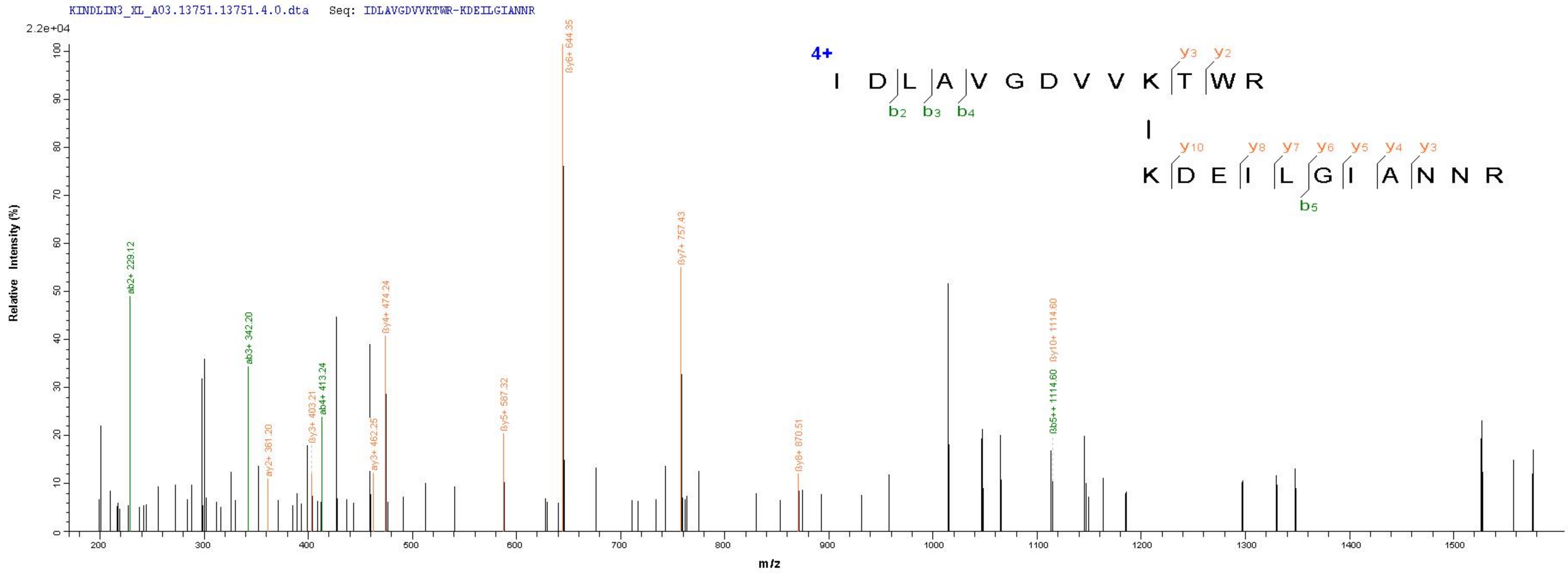
KINDLIN3_XL_A03.13751.13751.4.0.dta Seq: IDLAVGDVVKTWR-KDEILGIANNR
2.2e+04
Relative Intensity (%)
m/z
4+
I D L A V G D V V K T W R
K D E I L G I A N N R
ab2+ 229.12
ab3+ 342.20
ay2+ 361.20
ßy3+ 403.21
ab4+ 413.24
ay3+ 462.25
ßy4+ 474.24
ßy5+ 587.32
ßy6+ 644.35
ßy7+ 757.43
ßy8+ 870.51
ßb5++ 1114.60
ßy10+ 1114.60

KINDLIN3_XL_A03.13819.13819.4.0.dta Seq: IDLAVGDVVKTWR-KDEILGIANNR Mod: 25,_deamidated[NQ]

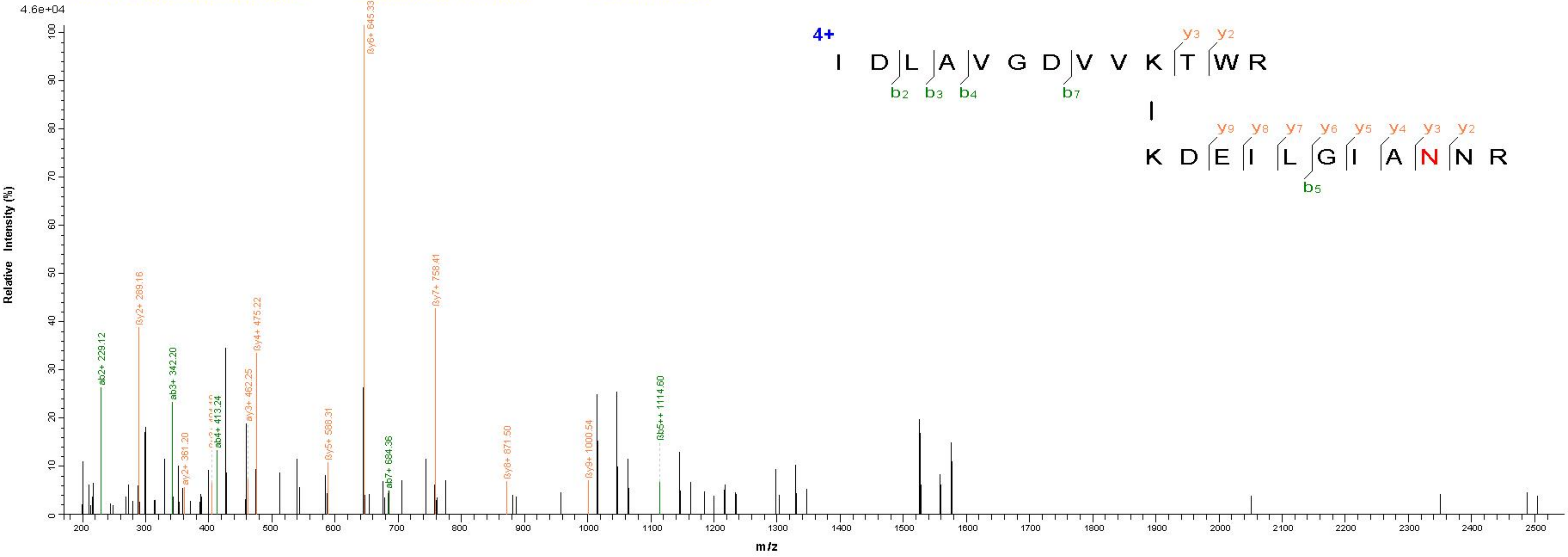

KINDLIN3_XL_A03.13860.13860.4.0.dta   Seq: IDLAVGDVVKTWR-KDEILGIANNR   Mod: 25,_deamidated[NQ]

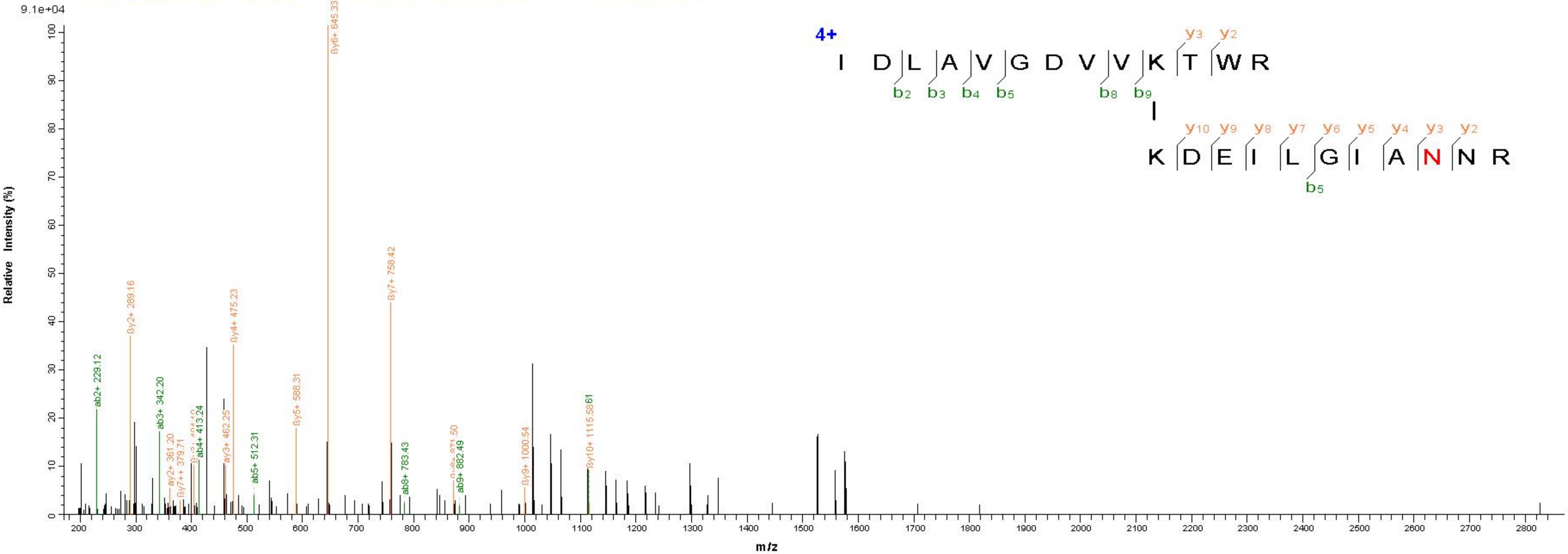

KINDLIN3_XL_A03.13911.13911.4.0.dta Seq: IDLAVGDVVKTWR-KDEILGIANNR Mod: 25,_deamidated[NQ]

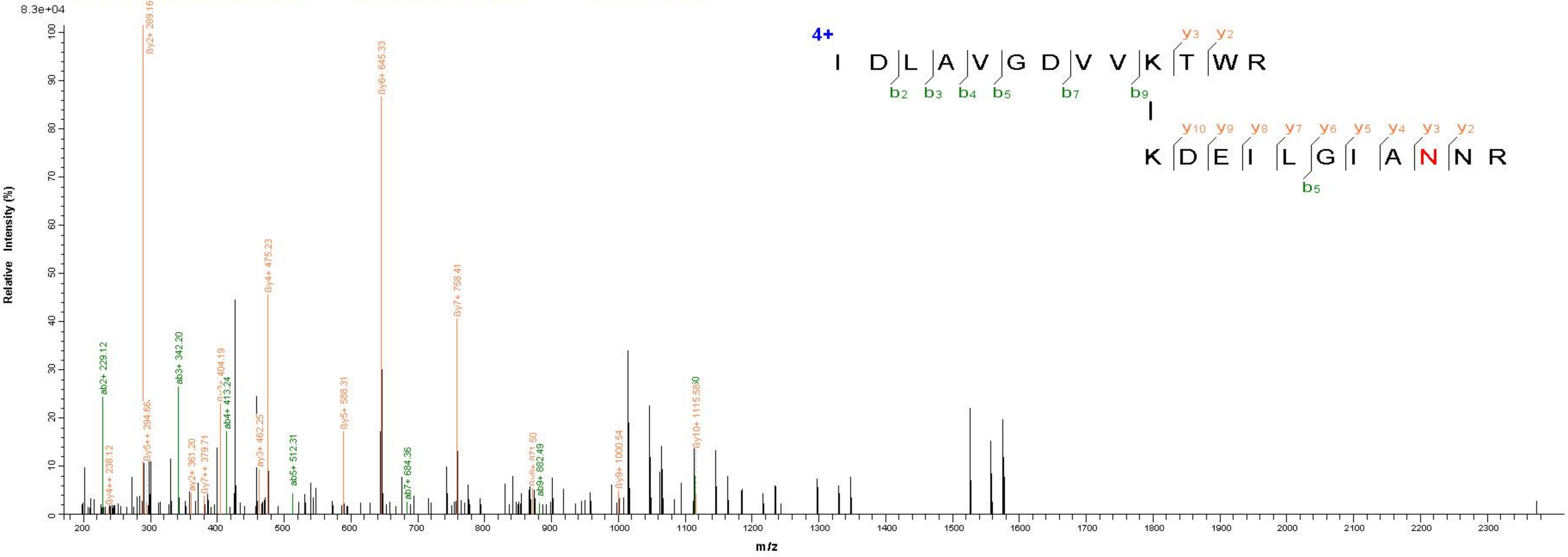

KINDLIN3_XL_A03.13948.13948.4.0.dta Seq: IDLAVGDVVKTWR-KDEILGIANNR Mod: 25,_deamidated[NQ]

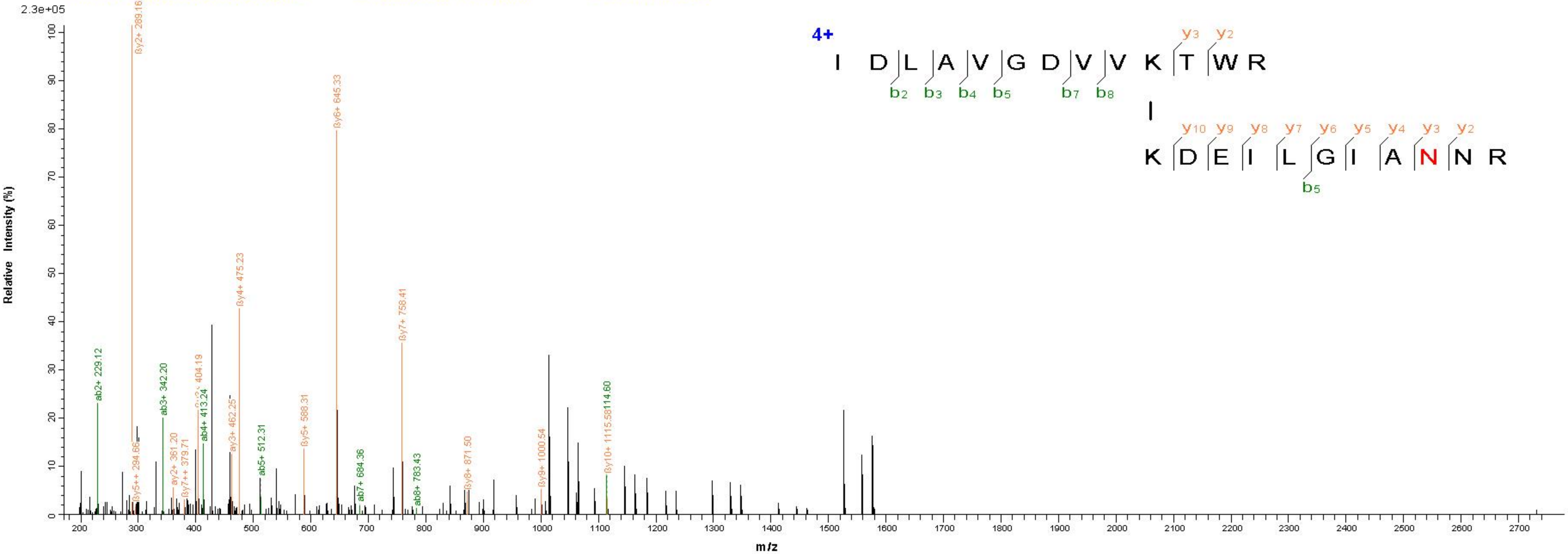

KINDLIN3_XL_A03.13981.13981.4.0.dta Seq: IDLAVGDVVKTWR-KDEILGIANNR Mod: 25,_deamidated[NQ]

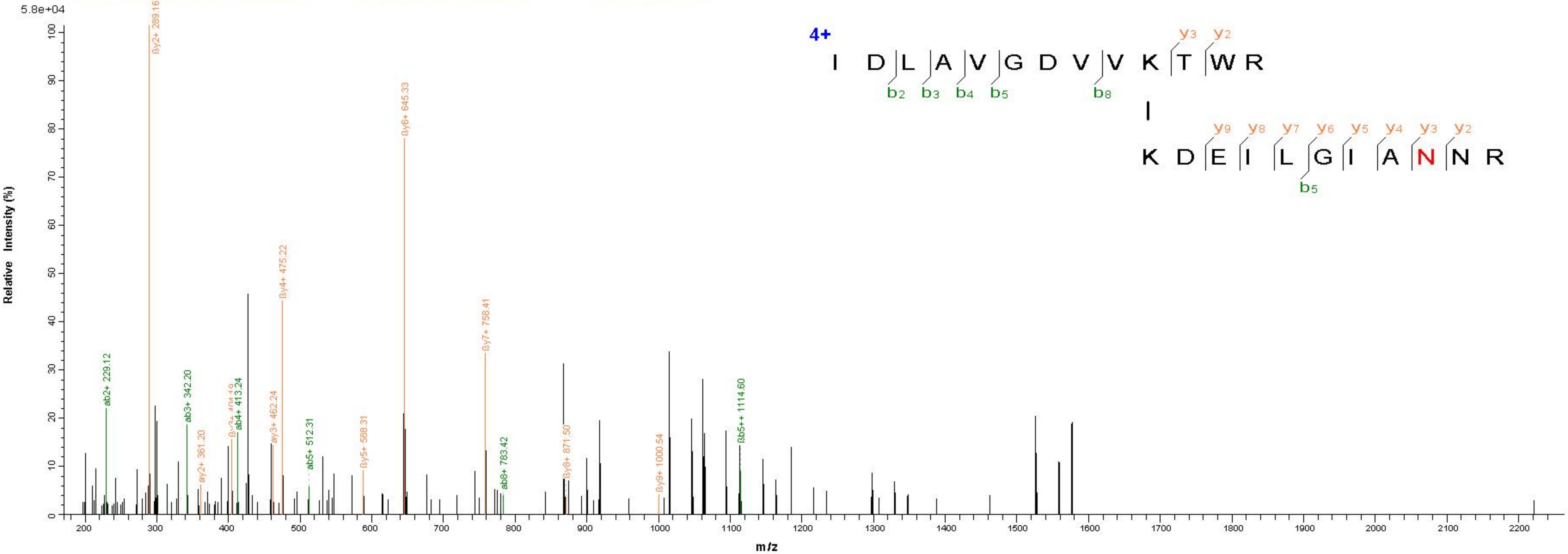

KINDLIN3_XL_B01.12988.12988.4.0.dta   Seq: IDLAVGDVVKTWR-KDEILGIANNR

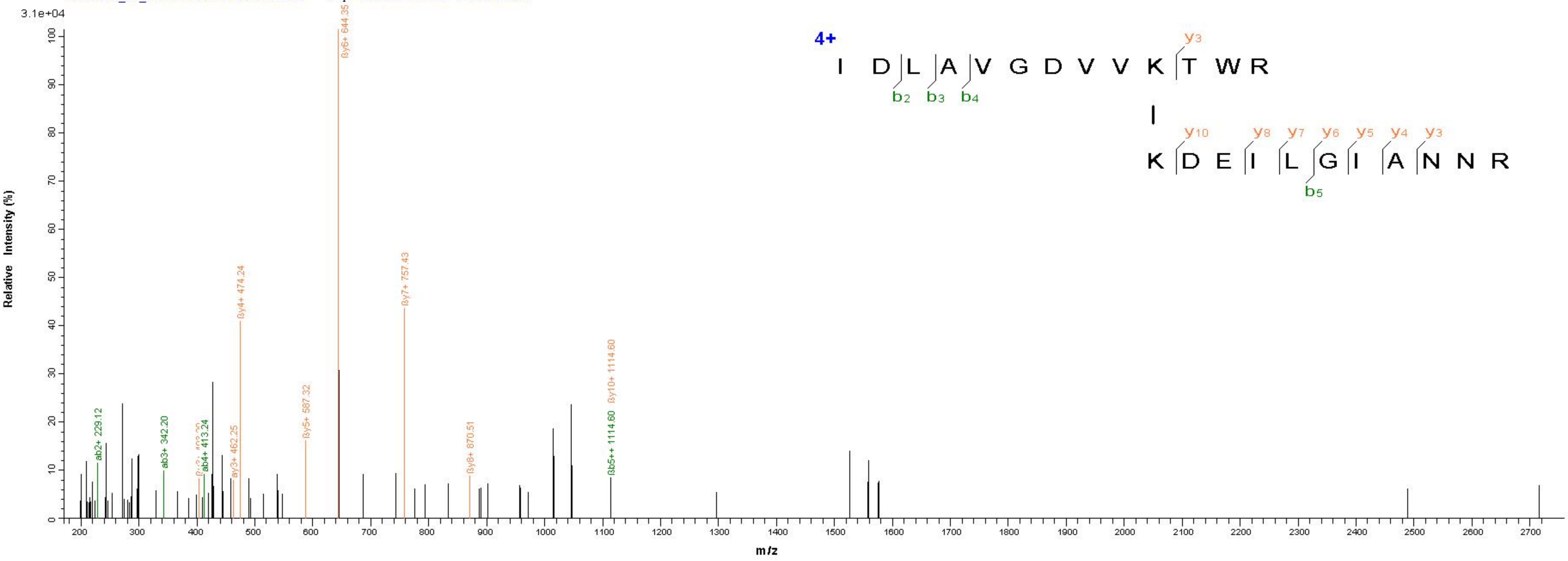

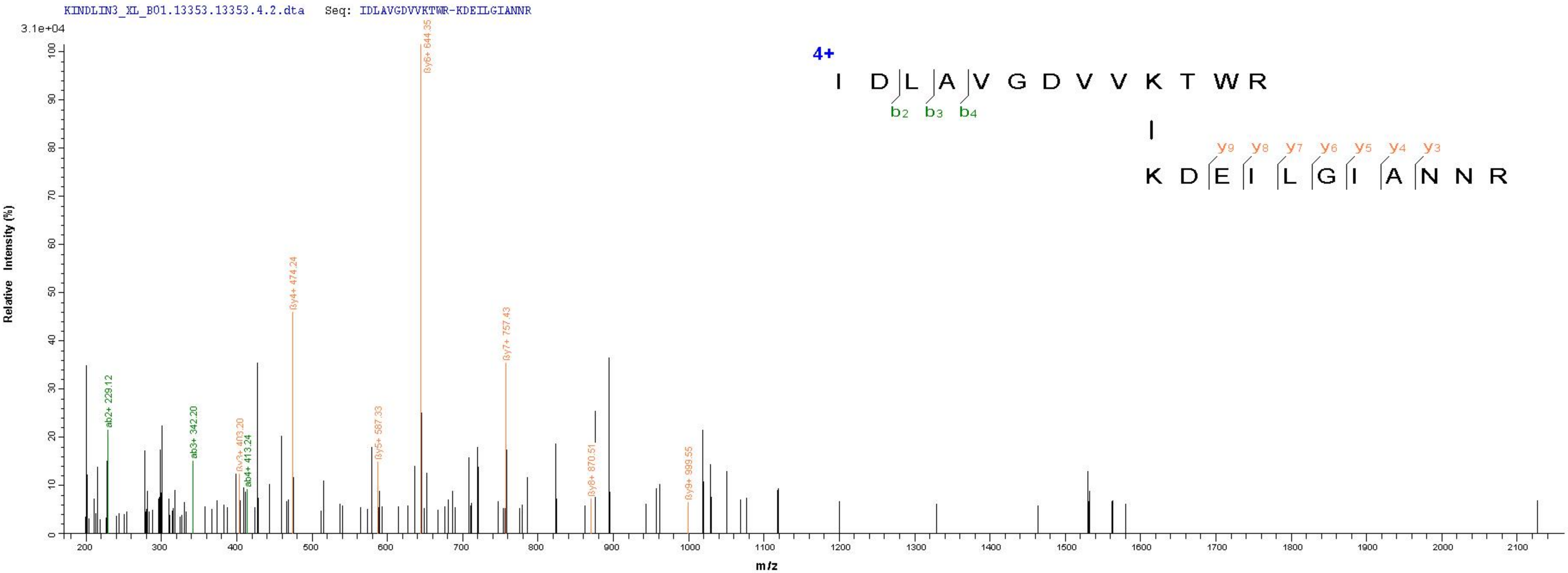
KINDLIN3_XL_B01.13353.13353.4.2.dta Seq: IDLAVGDVVKTWR-KDEILGIANNR
3.1e+04
Relative Intensity (%)
m/z
4+
I D L A V G D V V K T W R
b2 b3 b4
y9 y8 y7 y6 y5 y4 y3
K D E I L G I A N N R
ßy6+ 644.35
ßy4+ 474.24
ßy7+ 757.43
ab2+ 229.12
ab3+ 342.20
ßy3+ 403.20
ab4+ 413.24
ßy5+ 587.33
ßy8+ 870.51
ßy9+ 999.55

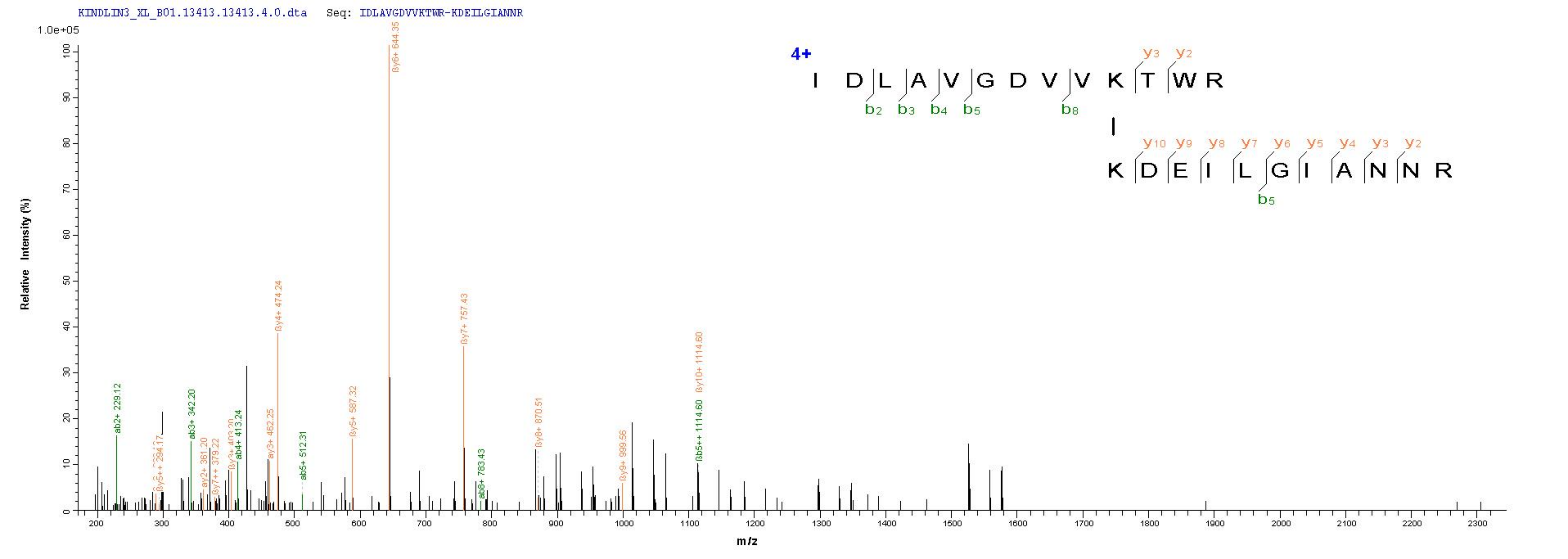
KINDLIN3_XL_B01.13413.13413.4.0.dta Seq: IDLAVGDVVKTWR-KDEILGIANNR
1.0e+05
Relative Intensity (%)
m/z
4+
I D L A V G D V V K T W R
K D E I L G I A N N R
ab2+ 229.12
ßy5++ 294.17
ab3+ 342.20
ay2+ 361.20
ßy7++ 379.22
ab4+ 413.24
ay3+ 462.25
ßy4+ 474.24
ab5+ 512.31
ßy5+ 587.32
ßy6+ 644.35
ßy7+ 757.43
ab8+ 783.43
ßy8+ 870.51
ßy9+ 999.56
ßb5++ 1114.60
ßy10+ 1114.60

KINDLIN3_XL_B01.13435.13435.3.0.dta   Seq: IDLAVGDVVKTWR-KDEILGIANNR

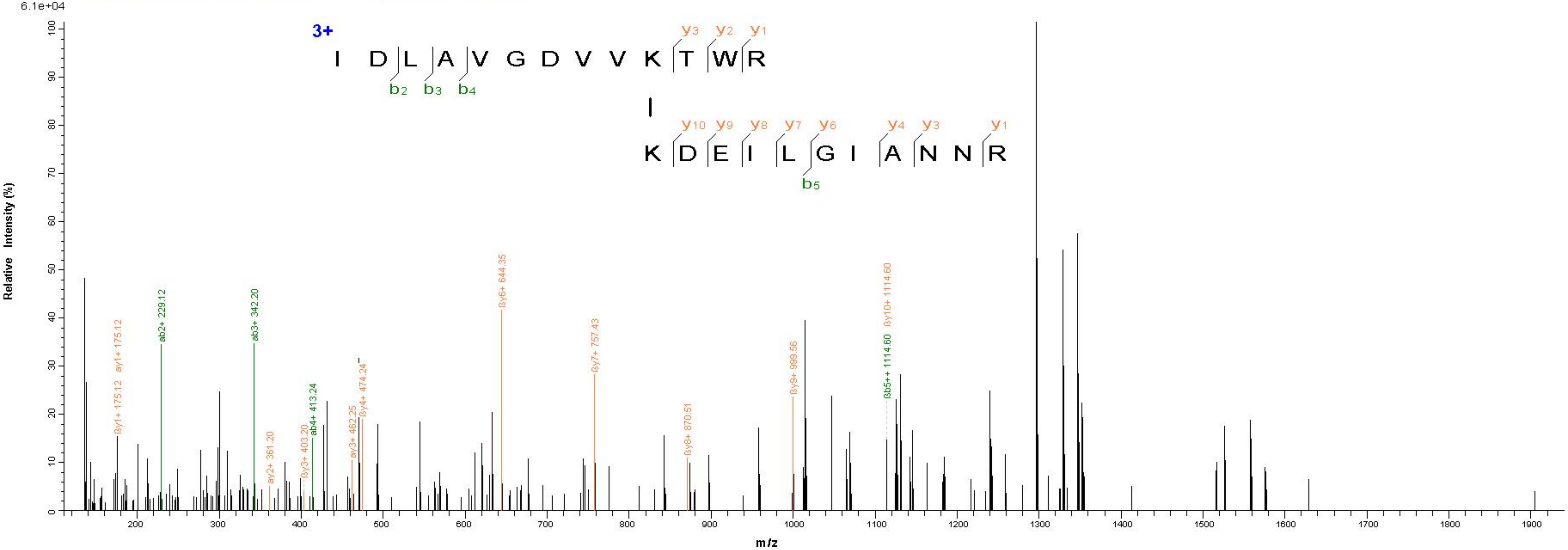

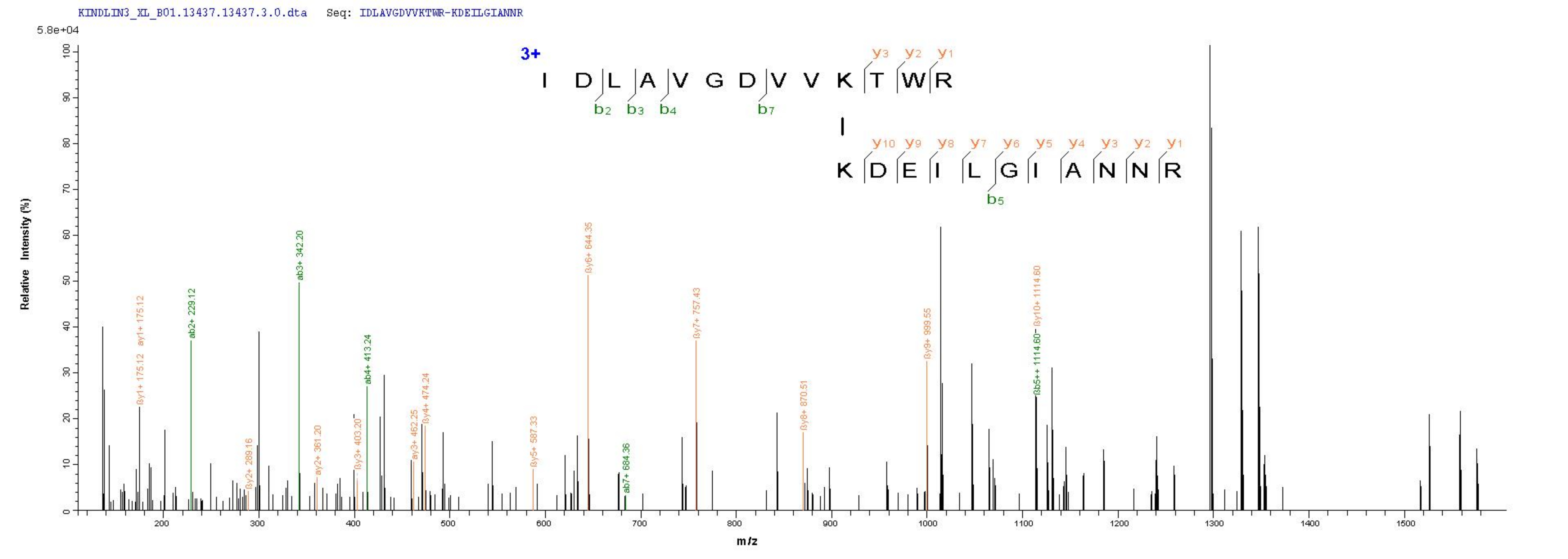

KINDLIN3_XL_B01.13437.13437.3.0.dta Seq: IDLAVGDVVKTWR-KDEILGIANNR
5.8e+04
3+
I D L A V G D V V K T W R
y3 y2 y1
b2 b3 b4 b7
K D E I L G I A N N R
y10 y9 y8 y7 y6 y5 y4 y3 y2 y1
b5
Relative Intensity (%)
m/z
ßy1+ 175.12
αy1+ 175.12
αb2+ 229.12
ßy2+ 289.16
αb3+ 342.20
αy2+ 361.20
ßy3+ 403.20
αb4+ 413.24
αy3+ 462.25
ßy4+ 474.24
ßy5+ 587.33
ßy6+ 644.35
αb7+ 684.36
ßy7+ 757.43
ßy8+ 870.51
ßy9+ 999.55
ßb5++ 1114.60
ßy10+ 1114.60

KINDLIN3_XL_B01.13441.13441.3.0.dta Seq: IDLAVGDVVKTWR-KDEILGIANNR

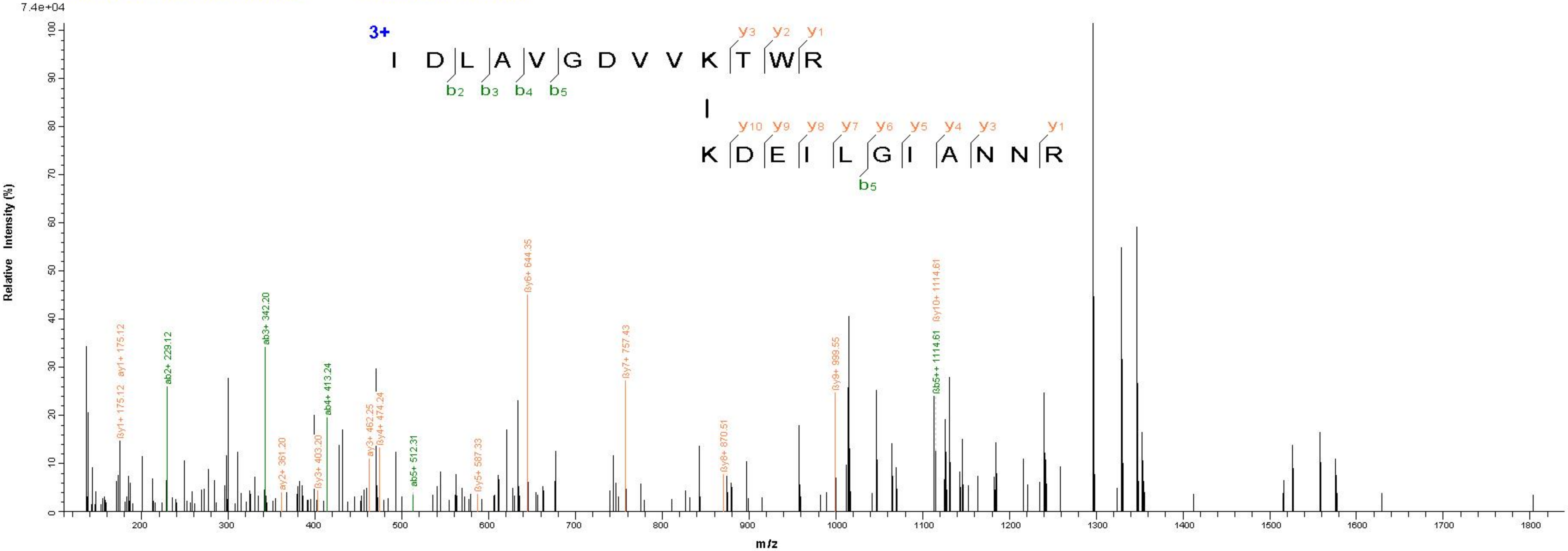

KINDLIN3_XL_B01.13447.13447.3.0.dta Seq: IDLAVGDVVKTWR-KDEILGIANNR

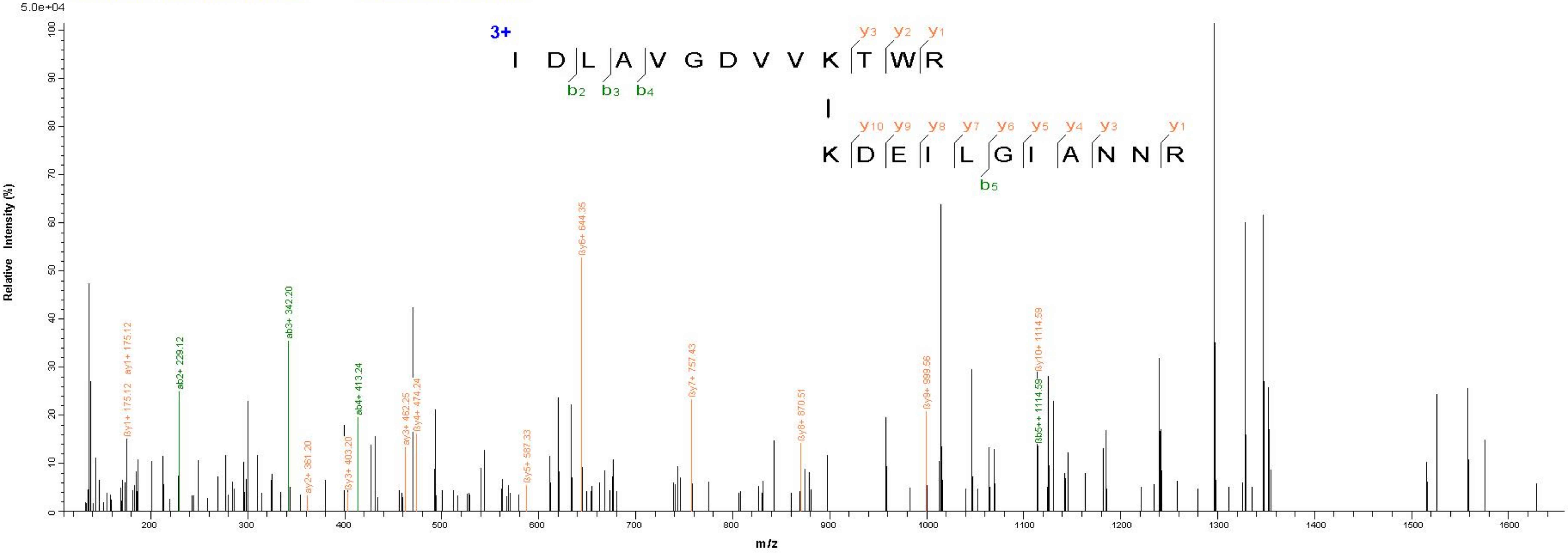

KINDLIN3_XL_B01.13449.13449.3.0.dta Seq: IDLAVGDVVKTWR-KDEILGIANNR

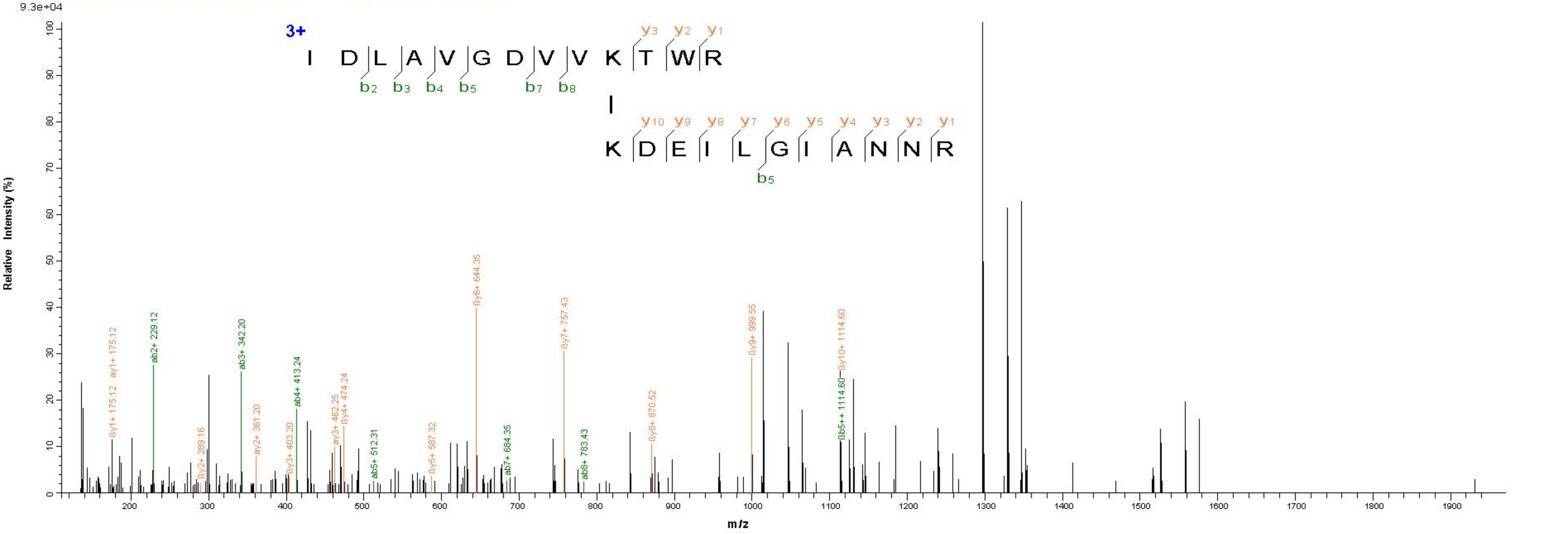

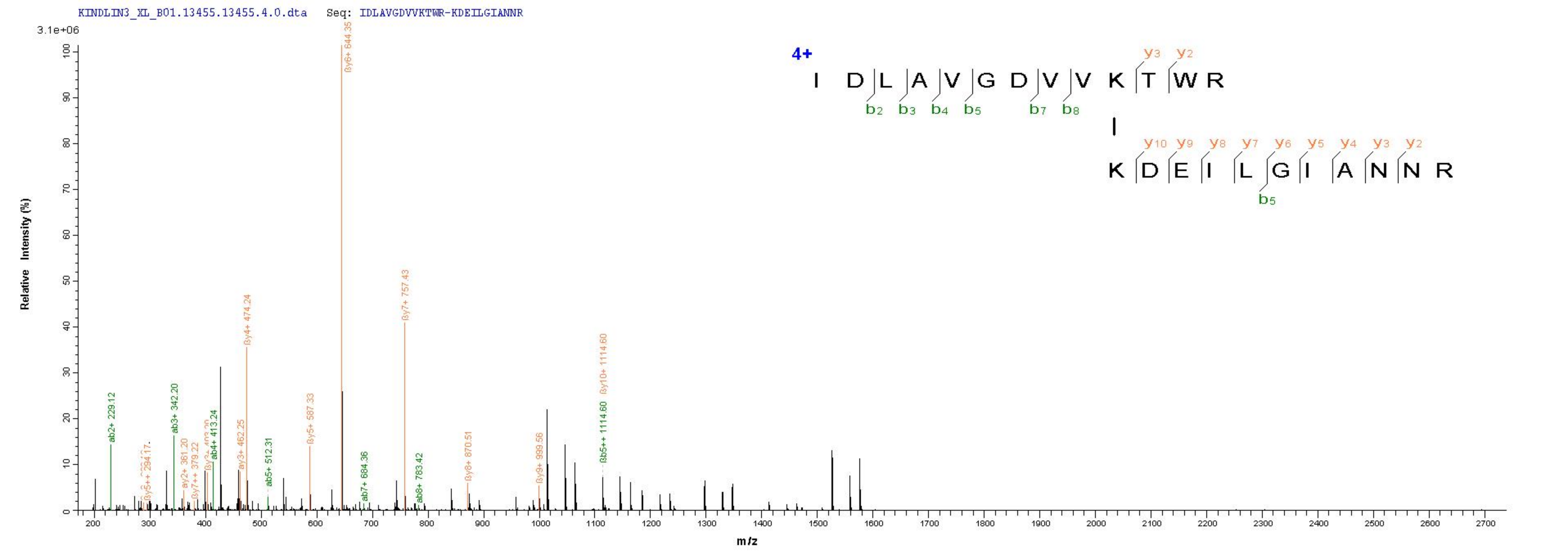

KINDLIN3_XL_B01.13455.13455.4.0.dta Seq: IDLAVGDVVKTWR-KDEILGIANNR
3.1e+06
Relative Intensity (%)
m/z
4+
I D L A V G D V V K T W R
K D E I L G I A N N R
ßy6+ 644.35
ßy7+ 757.43
ßy4+ 474.24
ßy10+ 1114.60
ab2+ 229.12
ab3+ 342.20
ab4+ 413.24
ay3+ 462.25
ßy5+ 587.33
ab5+ 512.31
ab7+ 684.36
ab8+ 783.42
ßy8+ 870.51
ßy9+ 999.56
ßb5++ 1114.60
ay2+ 361.20
ßy7++ 379.22
ßy5++ 294.17

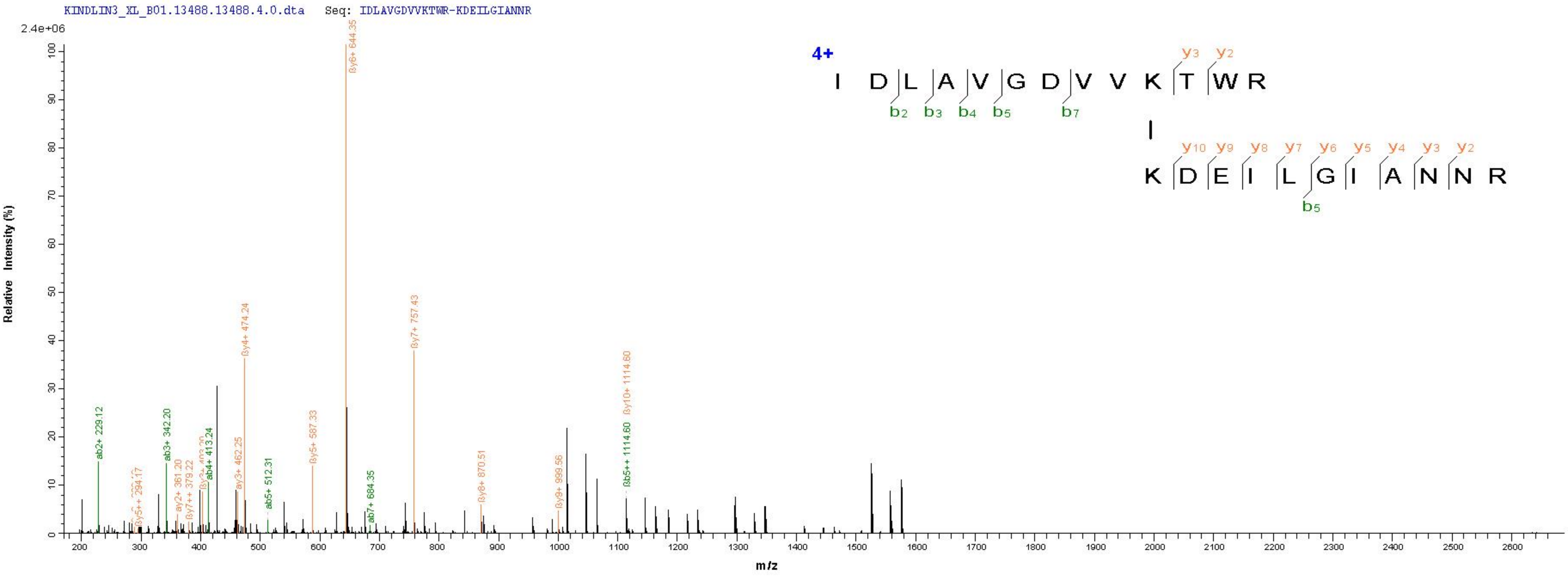
KINDLIN3_XL_B01.13488.13488.4.0.dta Seq: IDLAVGDVVKTWR-KDEILGIANNR
2.4e+06
Relative Intensity (%)
m/z
4+
I D L A V G D V V K T W R
y3 y2
b2 b3 b4 b5 b7
K D E I L G I A N N R
y10 y9 y8 y7 y6 y5 y4 y3 y2
b5
ßy6+ 644.35
ßy7+ 757.43
ßy4+ 474.24
ßy10+ 1114.60
ab2+ 229.12
ab3+ 342.20
ab4+ 413.24
ay3+ 462.25
ab5+ 512.31
ßy5+ 587.33
ab7+ 684.35
ßy8+ 870.51
ßy9+ 999.56
ßb5++ 1114.60
ay2+ 361.20
ßy7++ 379.22
ßy5++ 294.17

KINDLIN3_XL_B01.13521.13521.4.0.dta Seq: IDLAVGDVVKTWR-KDEILGIANNR

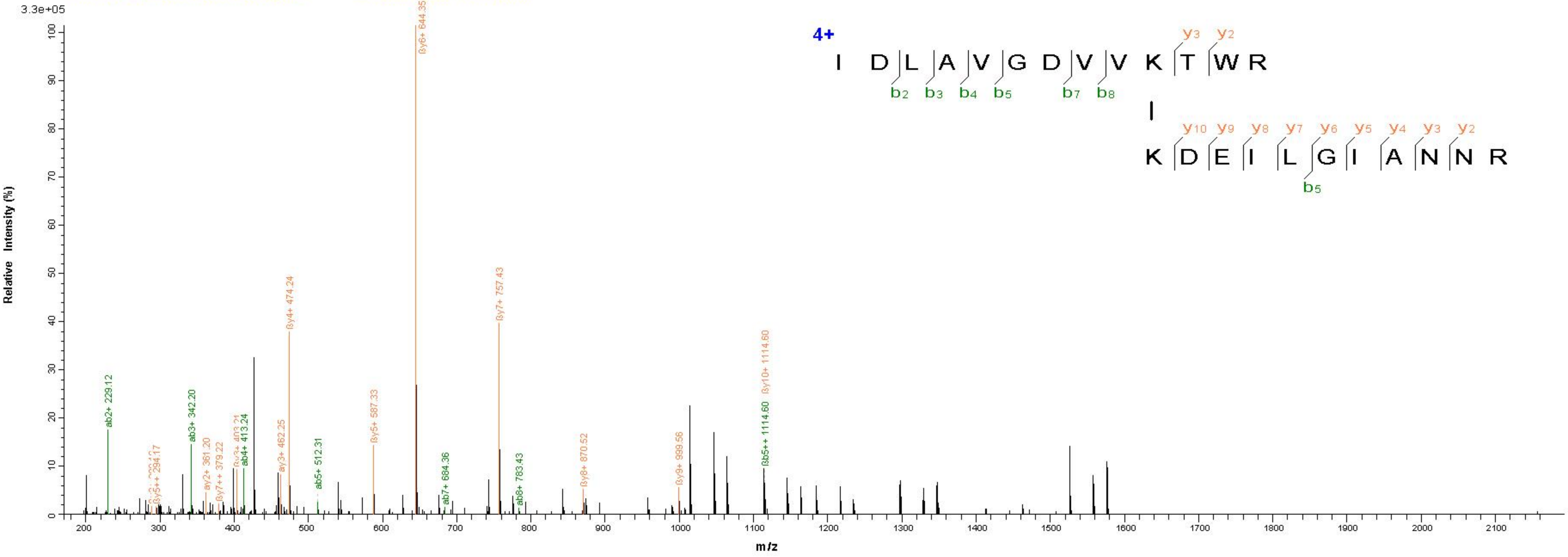

KINDLIN3_XL_B01.13554.13554.4.0.dta   Seq: IDLAVGDVVKTWR-KDEILGIANNR

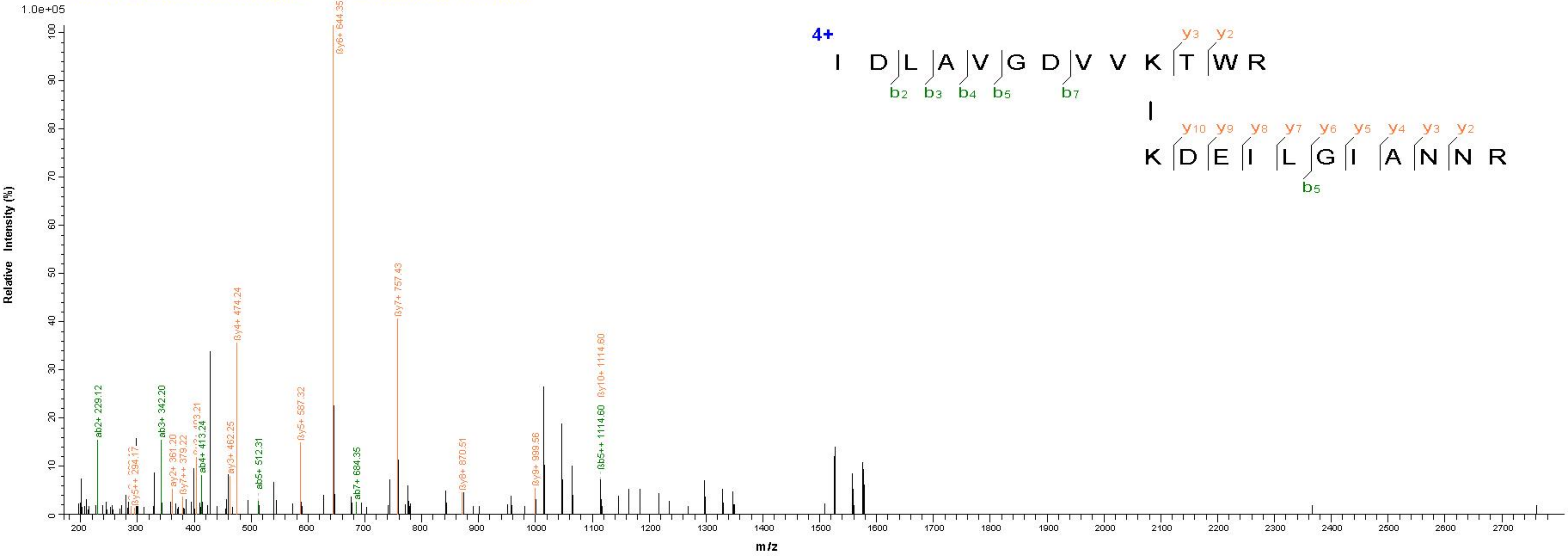

KINDLIN3_XL_B01.13589.13589.4.0.dta Seq: IDLAVGDVVKTWR-KDEILGIANNR

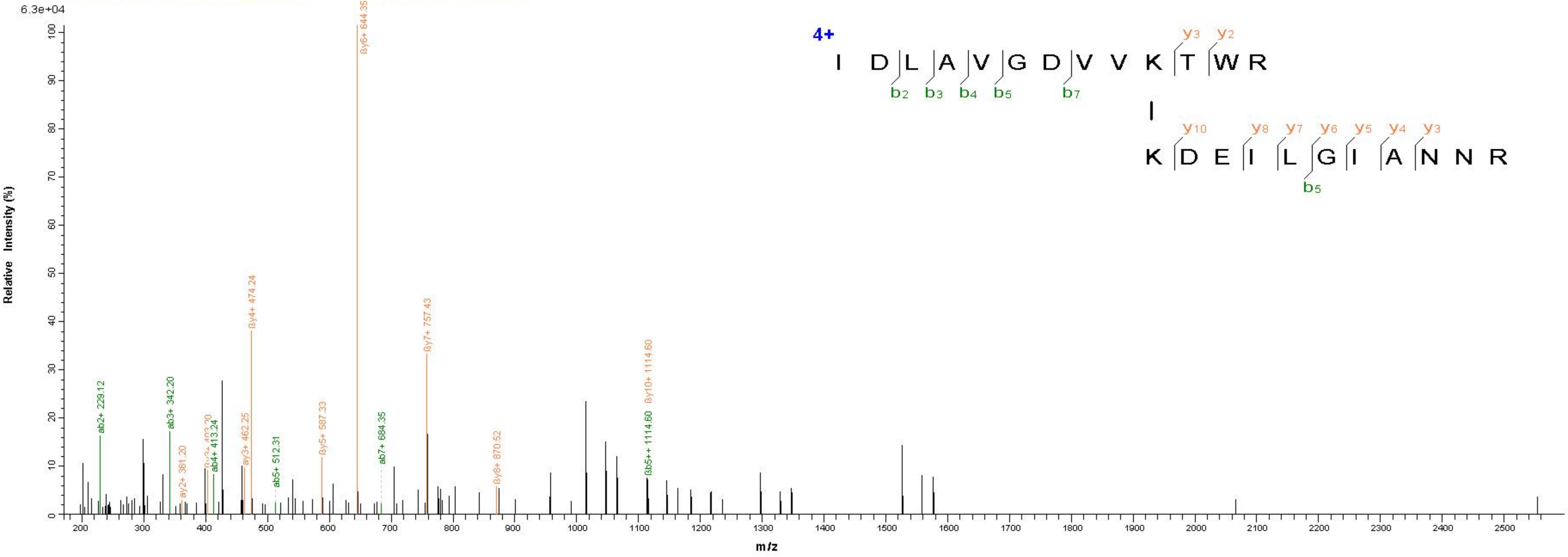

KINDLIN3_XL_B01.13634.13634.4.0.dta Seq: IDLAVGDVVKTWR-KDEILGIANNR

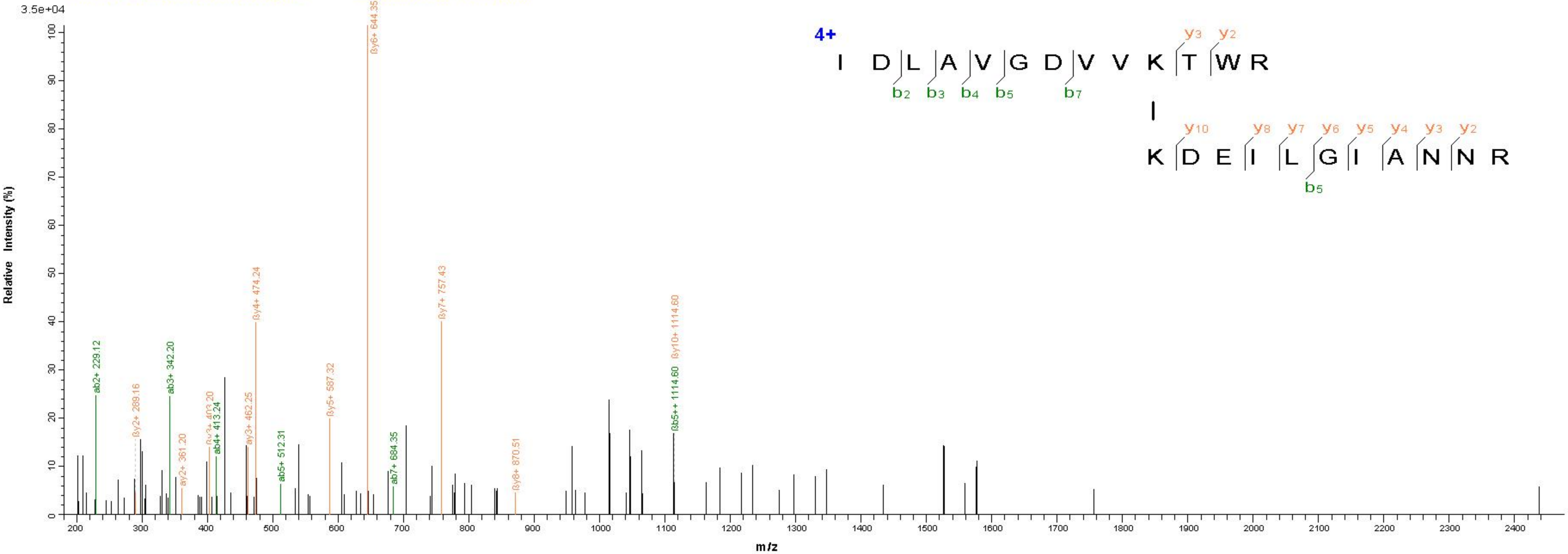

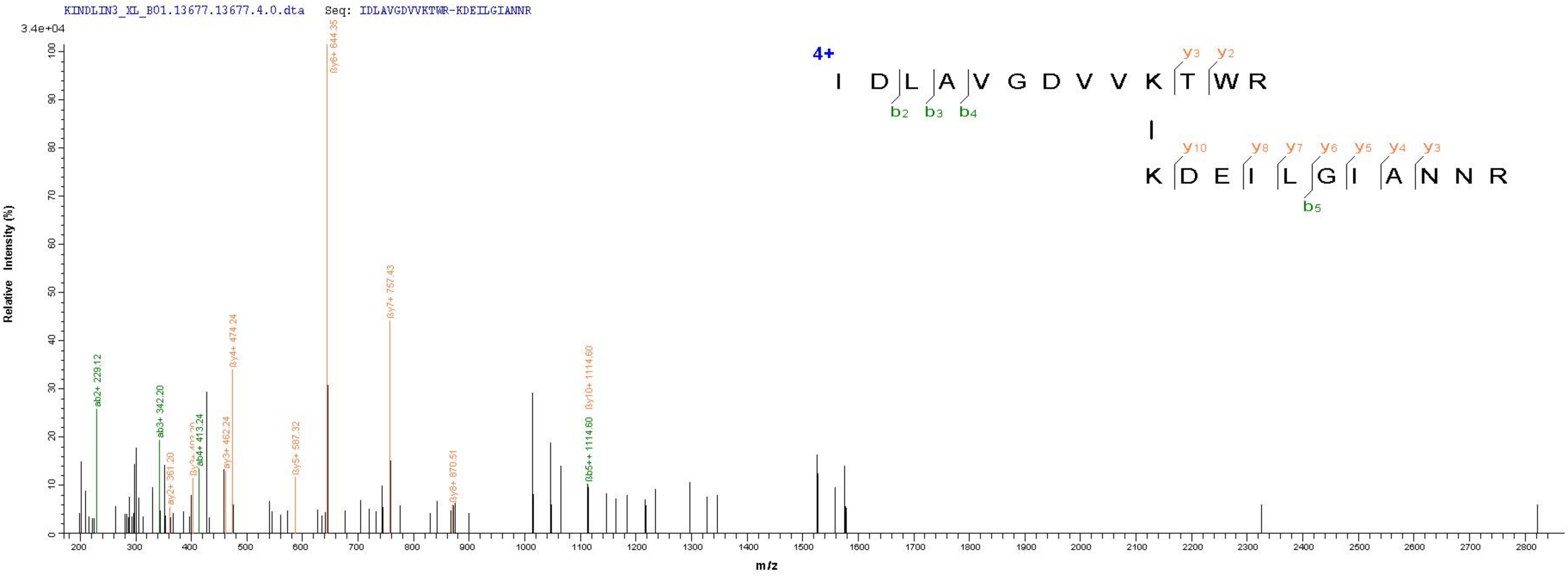

KINDLIN3_XL_B01.13677.13677.4.0.dta
Seq: IDLAVGDVVKTWR-KDEILGIANNR
3.4e+04
Relative Intensity (%)
m/z
4+
I D L A V G D V V K T W R
K D E I L G I A N N R
b2 b3 b4 b5
y3 y2
y10 y8 y7 y6 y5 y4 y3
ab2+ 229.12
ab3+ 342.20
ay2+ 361.20
ab4+ 413.24
ay3+ 462.24
ßy4+ 474.24
ßy5+ 587.32
ßy6+ 644.35
ßy7+ 757.43
ßy8+ 870.51
ßb5++ 1114.60
ßy10+ 1114.60

KINDLIN3_XL_B01.13798.13798.4.0.dta   Seq: IDLAVGDVVKTWR-KDEILGIANNR   Mod: 25,_deamidated[NQ]

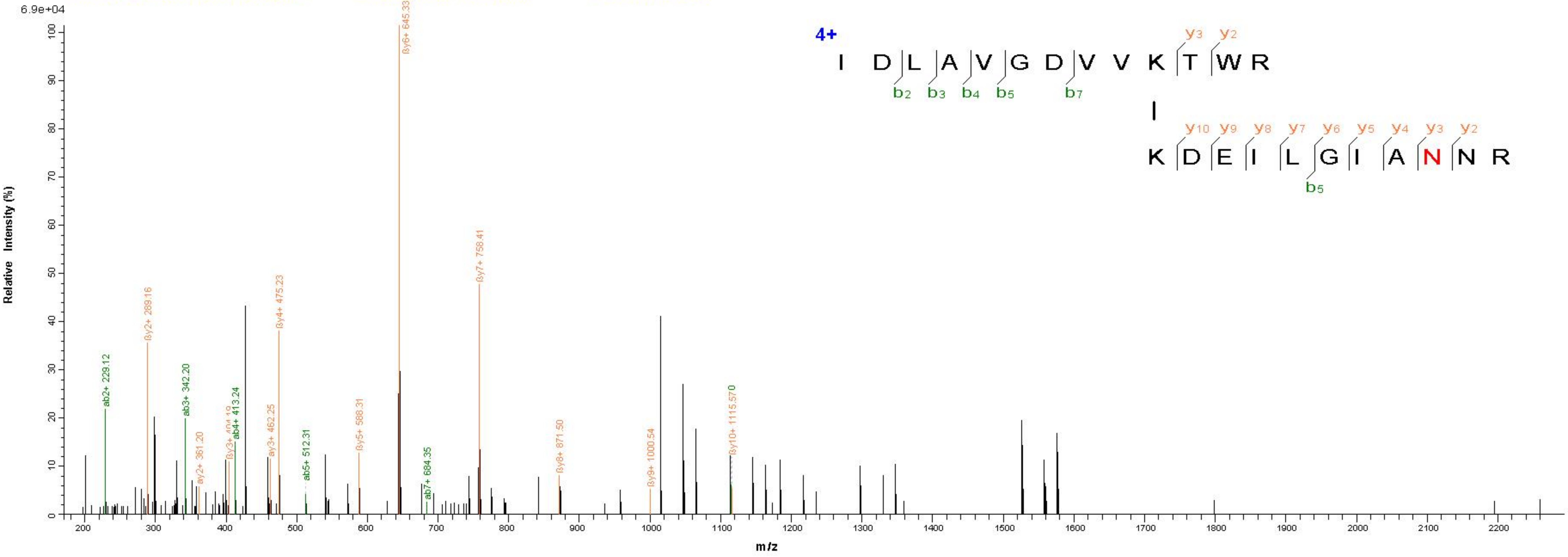

KINDLIN3_XL_B01.13849.13849.4.0.dta Seq: IDLAVGDVVKTWR-KDEILGIANNR Mod: 25,_deamidated[NQ]

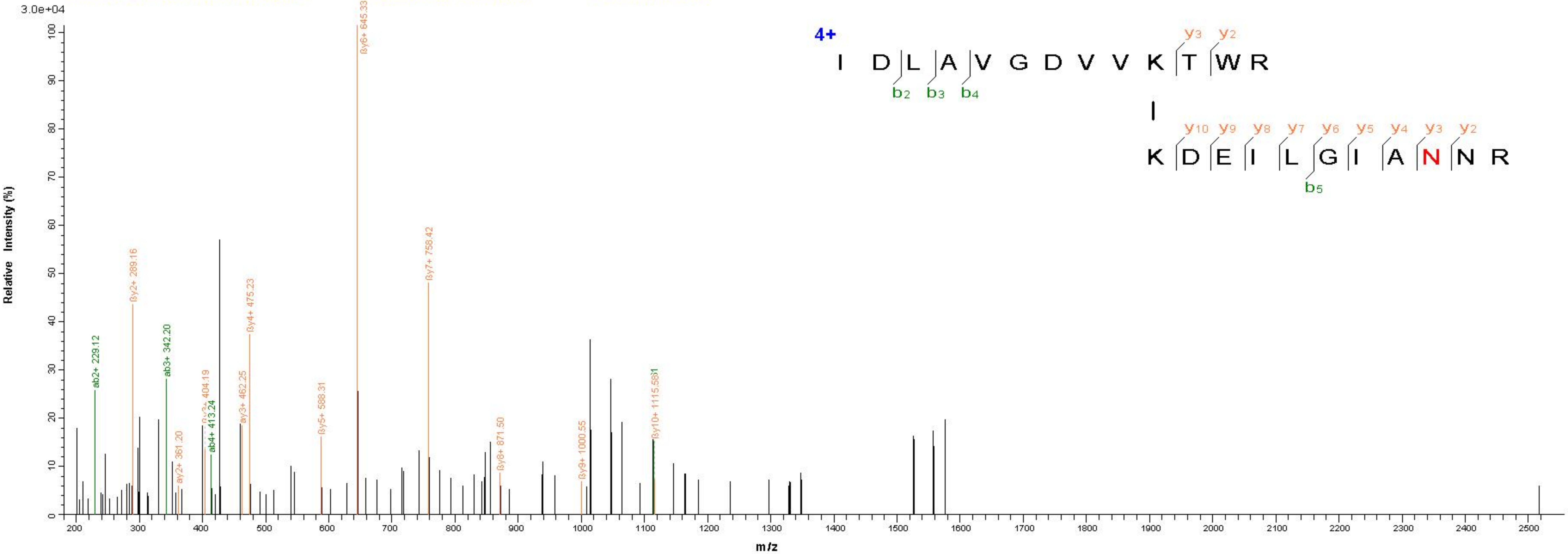

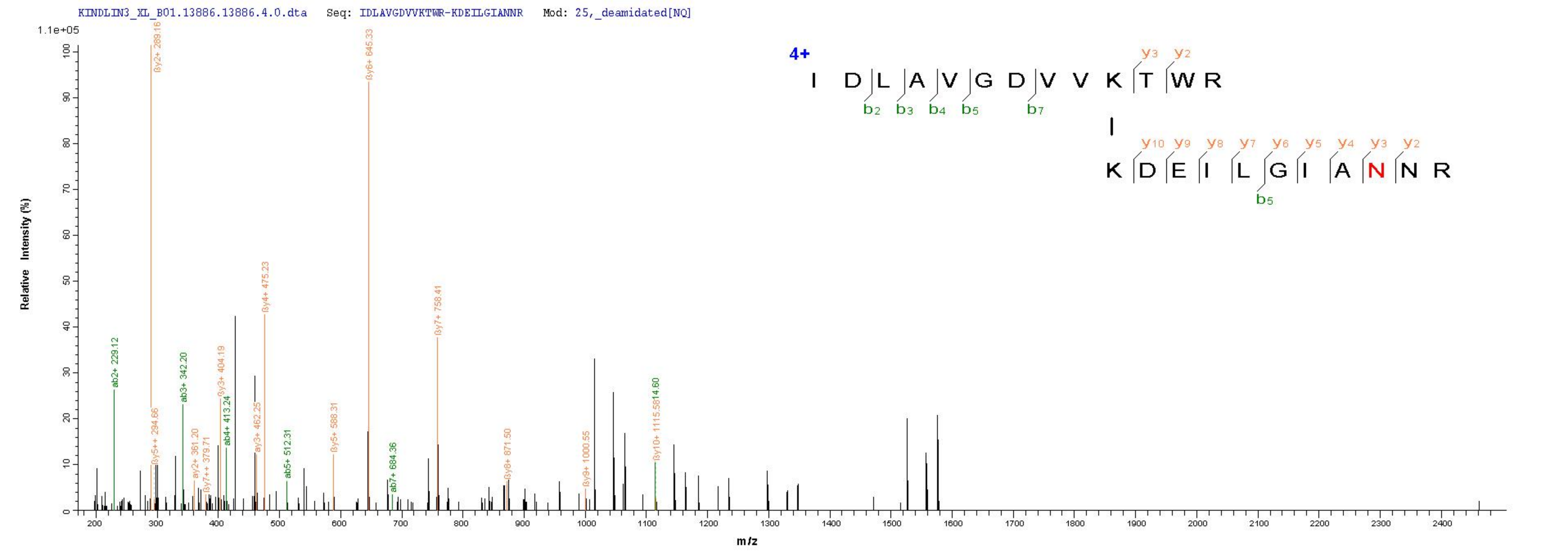
KINDLIN3_XL_B01.13886.13886.4.0.dta Seq: IDLAVGDVVKTWR-KDEILGIANNR Mod: 25,_deamidated[NQ]
1.1e+05
Relative Intensity (%)
m/z
4+
I D L A V G D V V K T W R
K D E I L G I A N N R
y3 y2
b2 b3 b4 b5 b7
y10 y9 y8 y7 y6 y5 y4 y3 y2
b5
ßy2+ 289.16
ßy6+ 645.33
ßy4+ 475.23
ßy7+ 758.41
ab2+ 229.12
ab3+ 342.20
ßy3+ 404.19
ab4+ 413.24
ay3+ 462.25
ßy5++ 294.66
ay2+ 361.20
ßy7++ 379.71
ab5+ 512.31
ßy5+ 588.31
ab7+ 684.36
ßy8+ 871.50
ßy9+ 1000.55
ßy10+ 1115.5814.60

KINDLIN3_XL_B01.13928.13928.4.0.dta   Seq: IDLAVGDVVKTWR-KDEILGIANNR   Mod: 25,_deamidated[NQ]

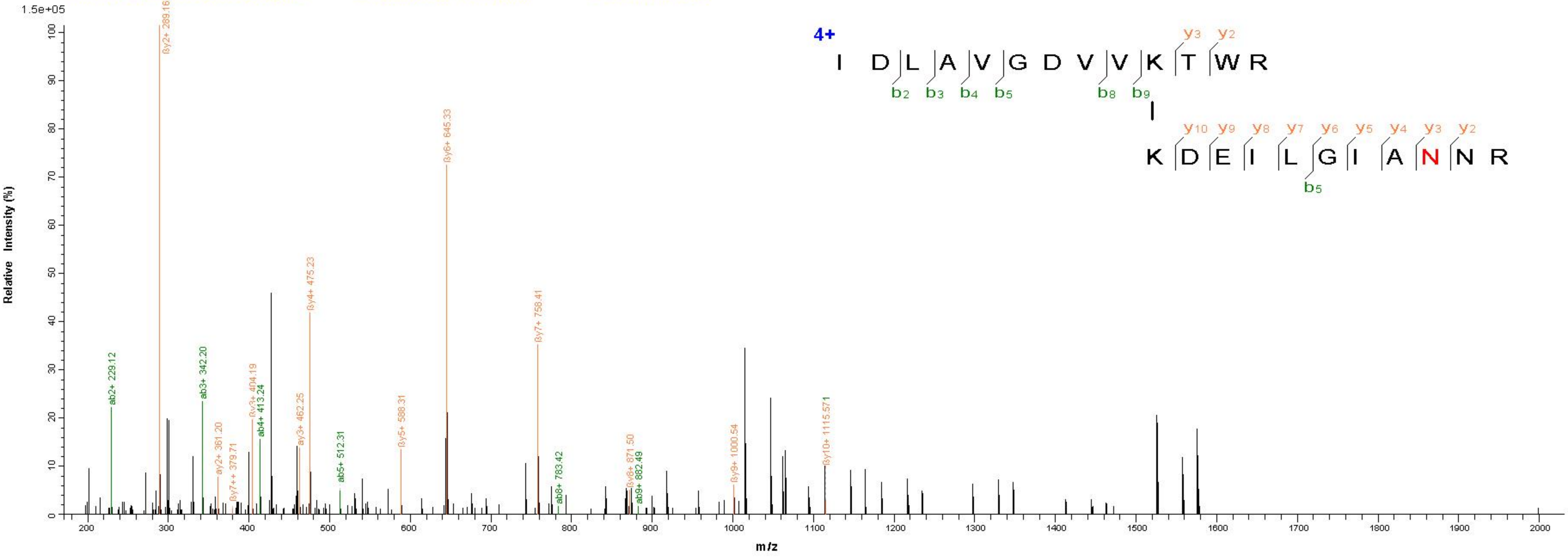

KINDLIN3_XL_B01.13934.13934.4.0.dta   Seq: IDLAVGDVVKTWR-KDEILGIANNR   Mod: 25,_deamidated[NQ]

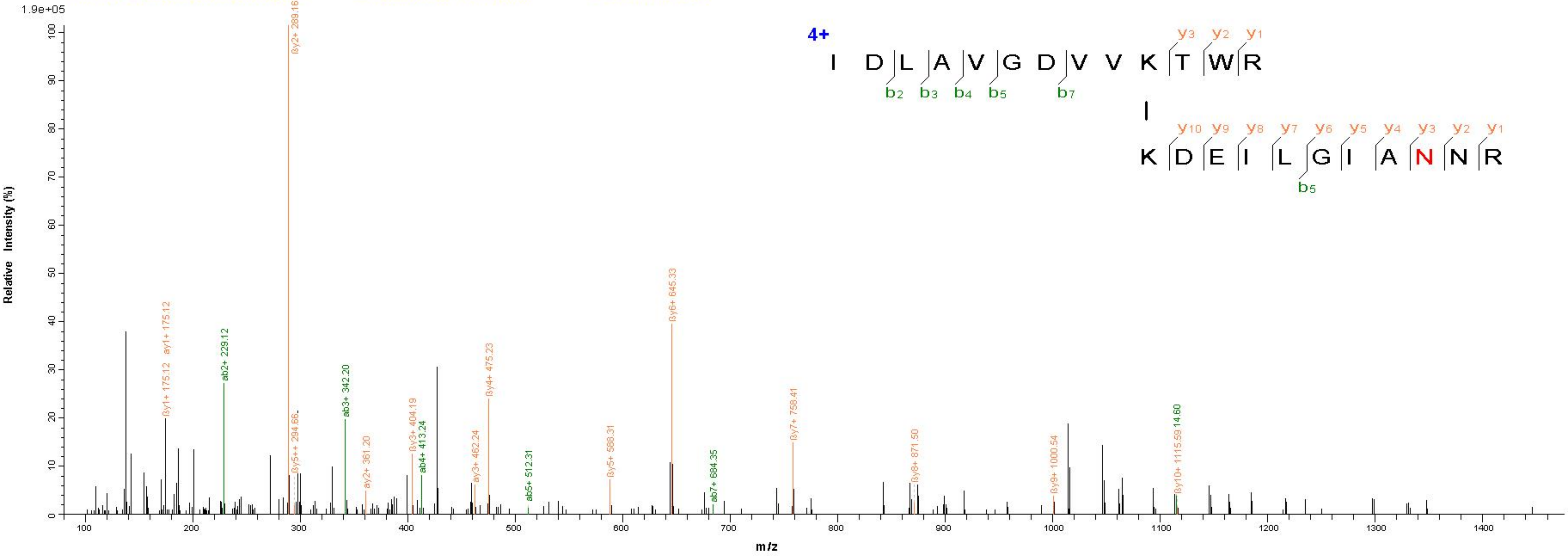

KINDLIN3_XL_B01.13945.13945.3.0.dta Seq: IDLAVGDVVKTWR-KDEILGIANNR Mod: 25,_deamidated[NQ]

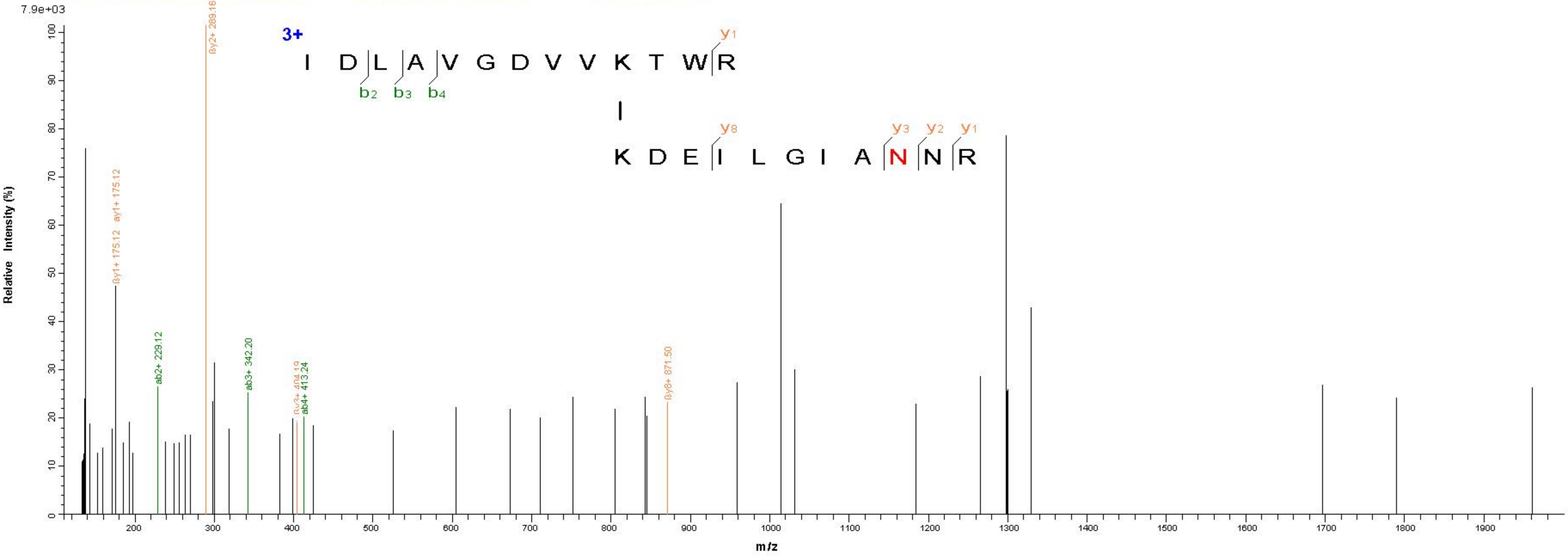

KINDLIN3_XL_B01.13962.13962.4.0.dta Seq: IDLAVGDVVKTWR-KDEILGIANNR Mod: 25,_deamidated[NQ]

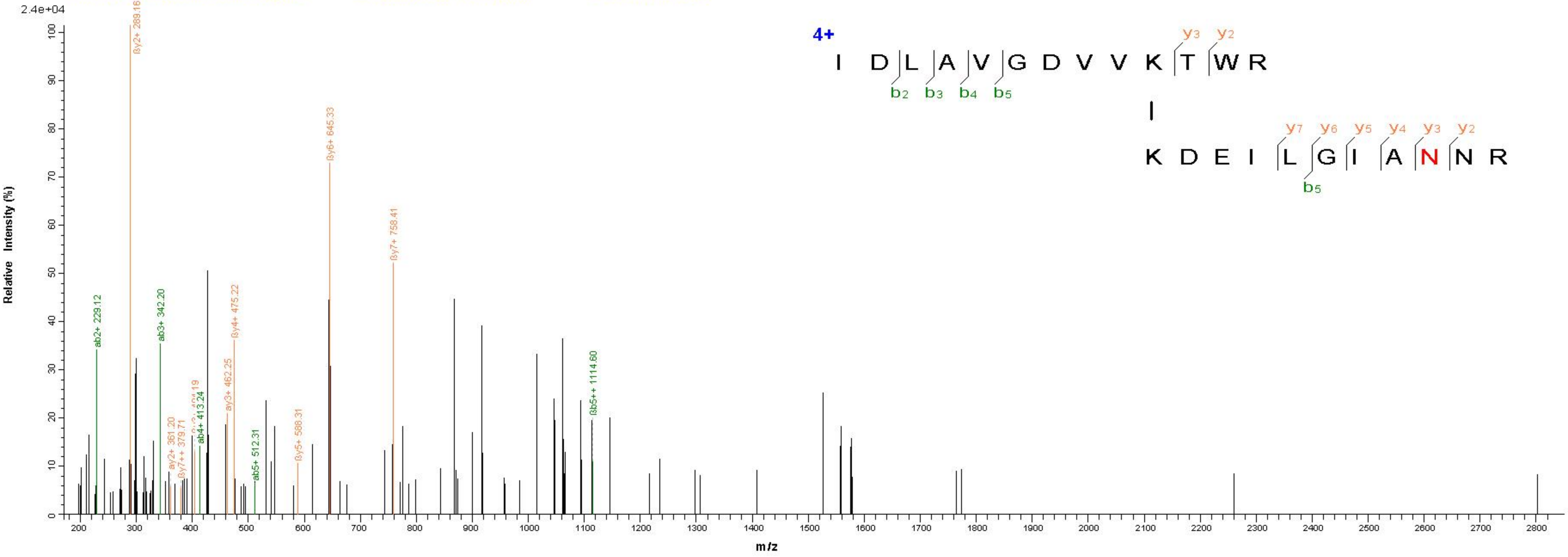

KINDLIN3_XL_B02.13016.13016.4.0.dta Seq: IDLAVGDVVKTWR-KDEILGIANNR

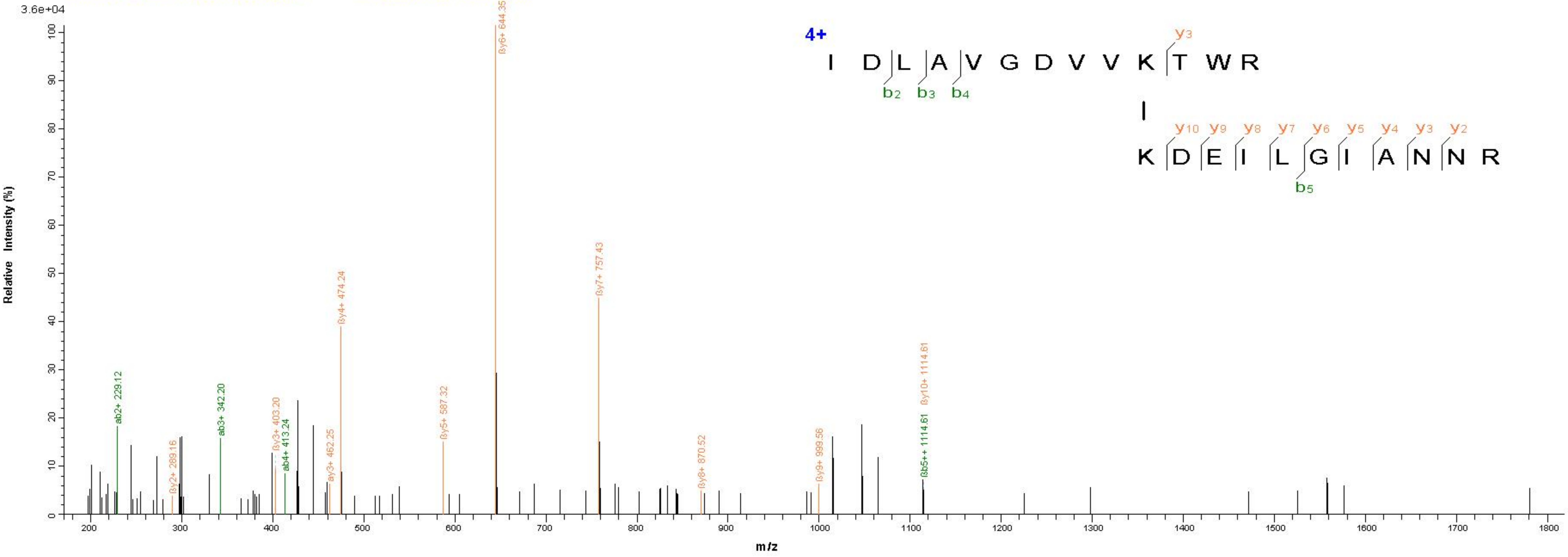

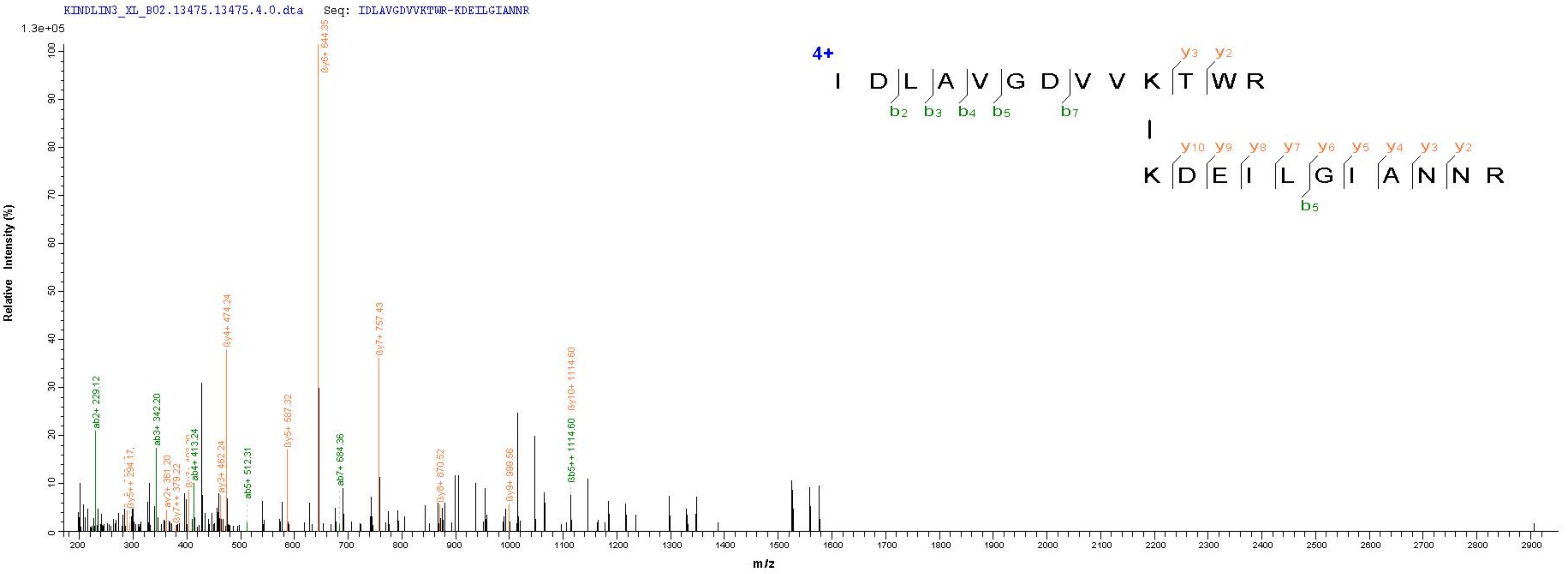

KINDLIN3_XL_B02.13475.13475.4.0.dta Seq: IDLAVGDVVKTWR-KDEILGIANNR
1.3e+05
Relative Intensity (%)
m/z
4+
I D L A V G D V V K T W R
K D E I L G I A N N R
ab2+ 229.12
ab3+ 342.20
ab4+ 413.24
ab5+ 512.31
ab7+ 684.36
ßy6+ 644.35
ßy7+ 757.43
ßy4+ 474.24
ßy5+ 587.32
ay3+ 462.24
ay2+ 361.20
ßy7++ 379.22
ßy8+ 870.52
ßy9+ 999.56
ßy10+ 1114.60
ßb5++ 1114.60

KINDLIN3_XL_B02.13498.13498.3.0.dta Seq: IDLAVGDVVKTWR-KDEILGIANNR

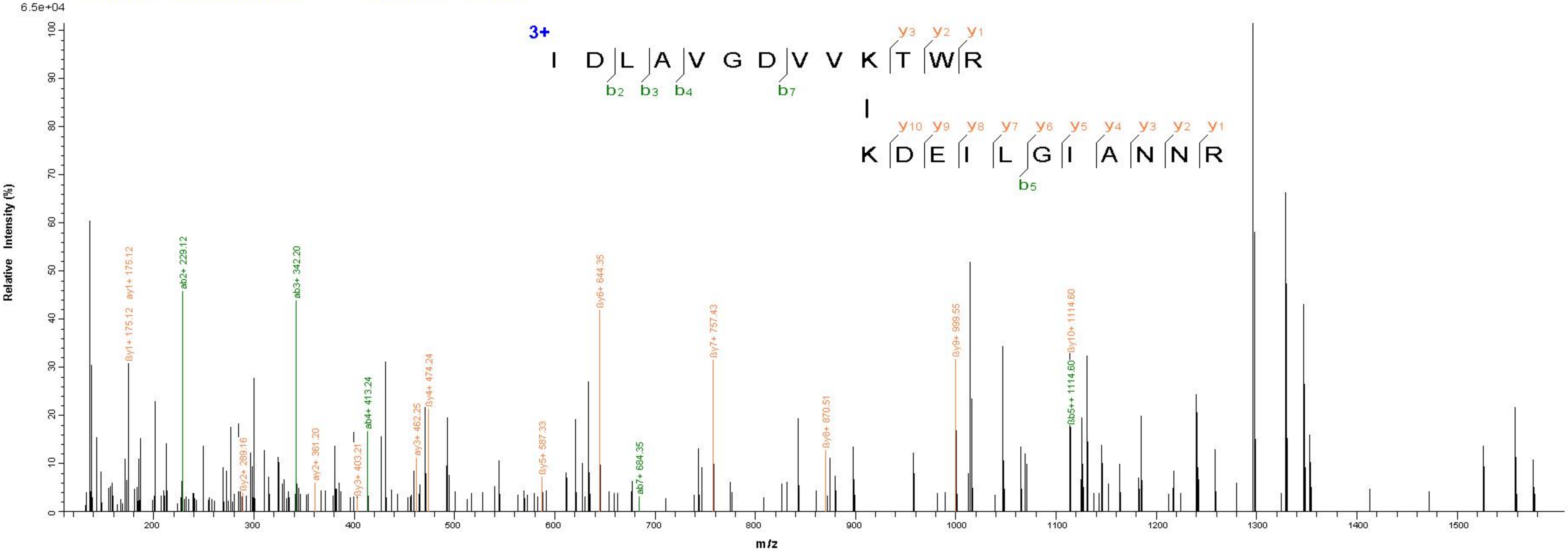

KINDLIN3_XL_B02.13499.13499.3.0.dta Seq: IDLAVGDVVKTWR-KDEILGIANNR

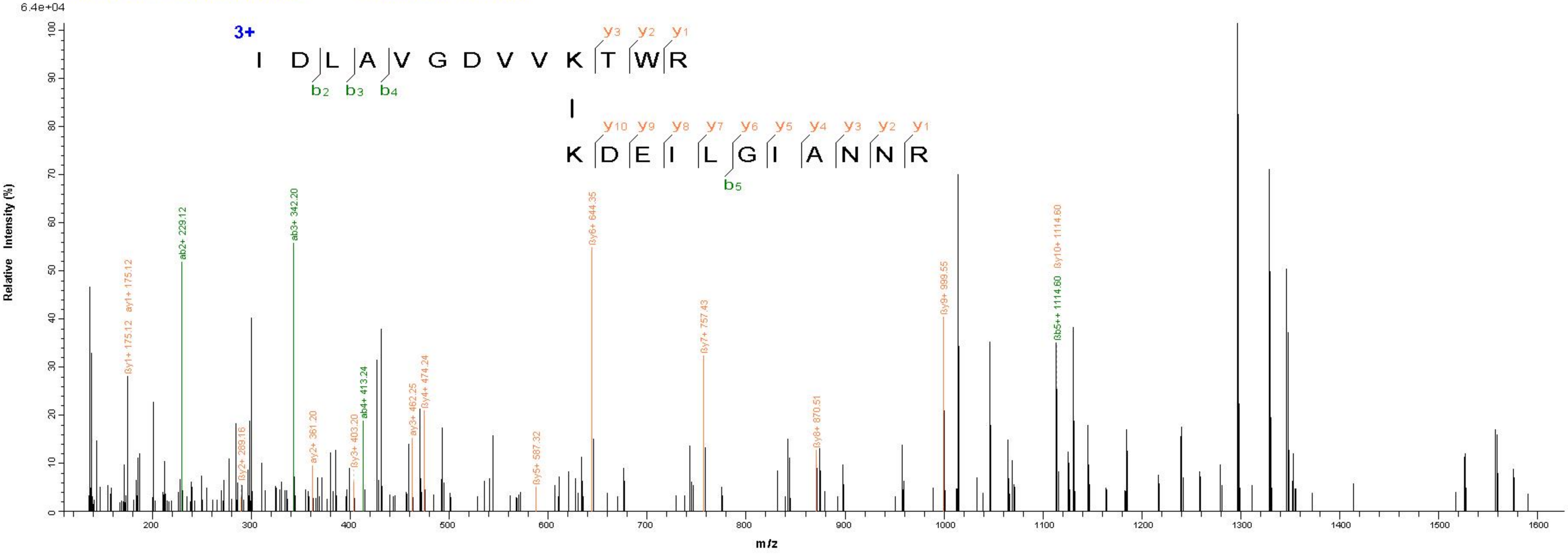

KINDLIN3_XL_B02.13503.13503.3.1.dta Seq: IDLAVGDVVKTWR-KDEILGIANNR

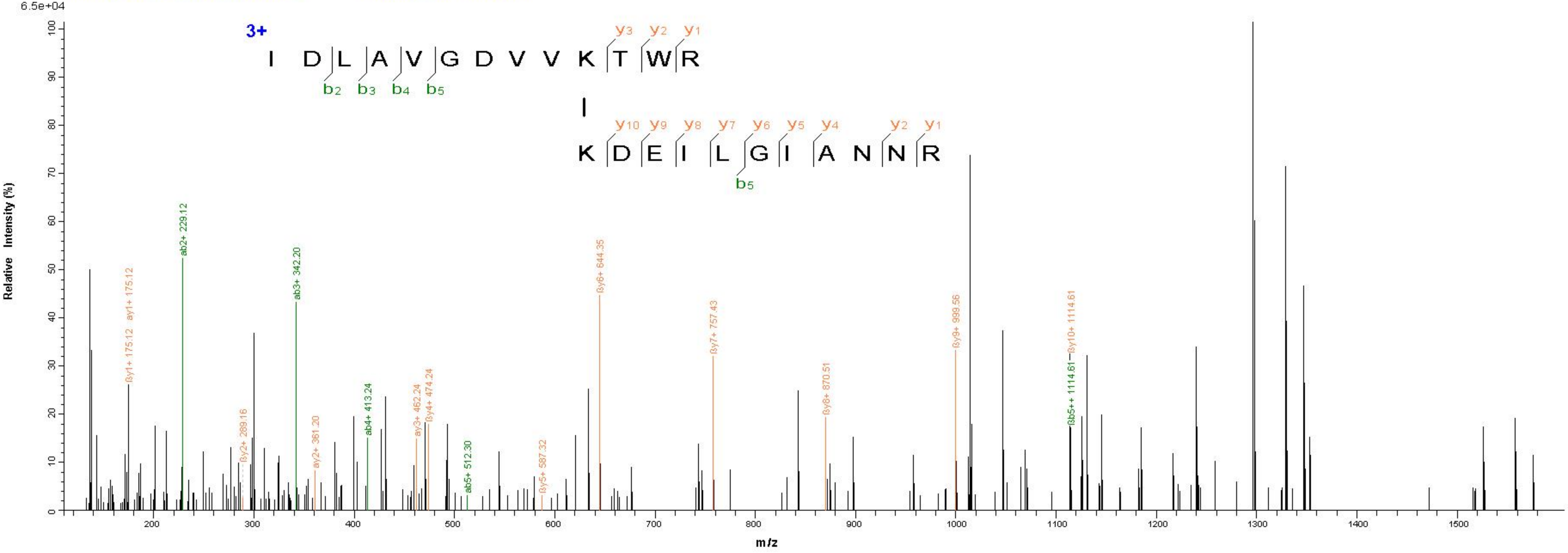

KINDLIN3_XL_B02.13513.13513.3.0.dta Seq: IDLAVGDVVKTWR-KDEILGIANNR

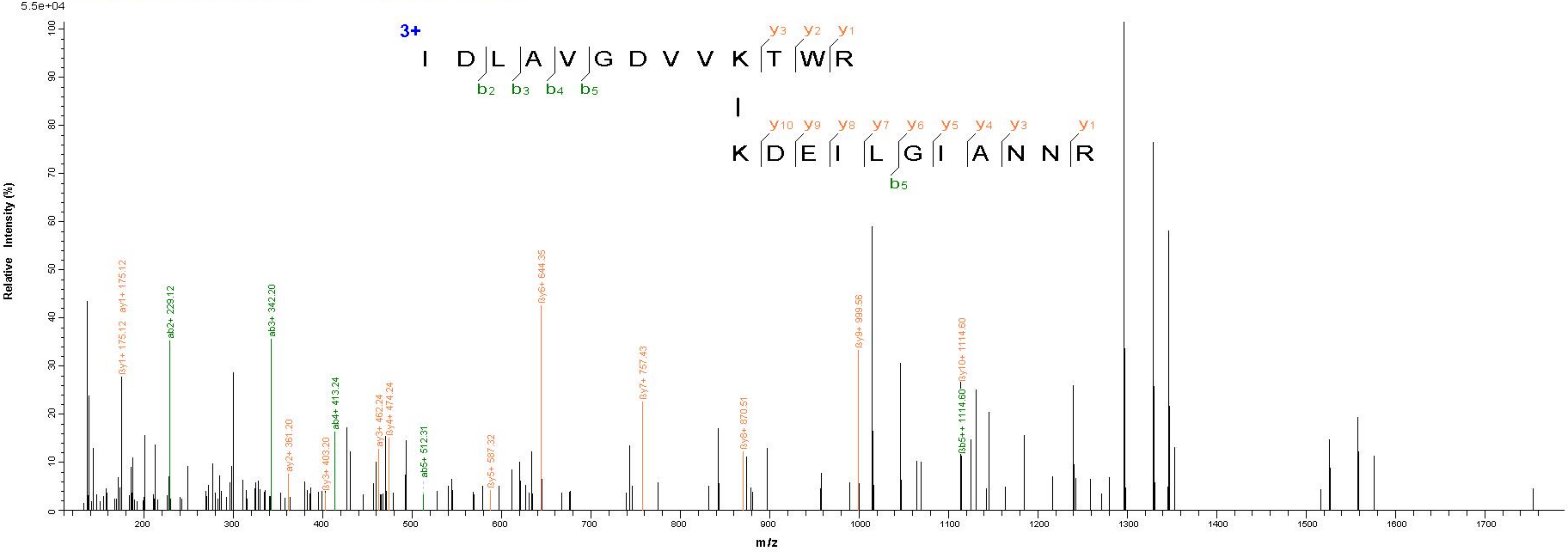

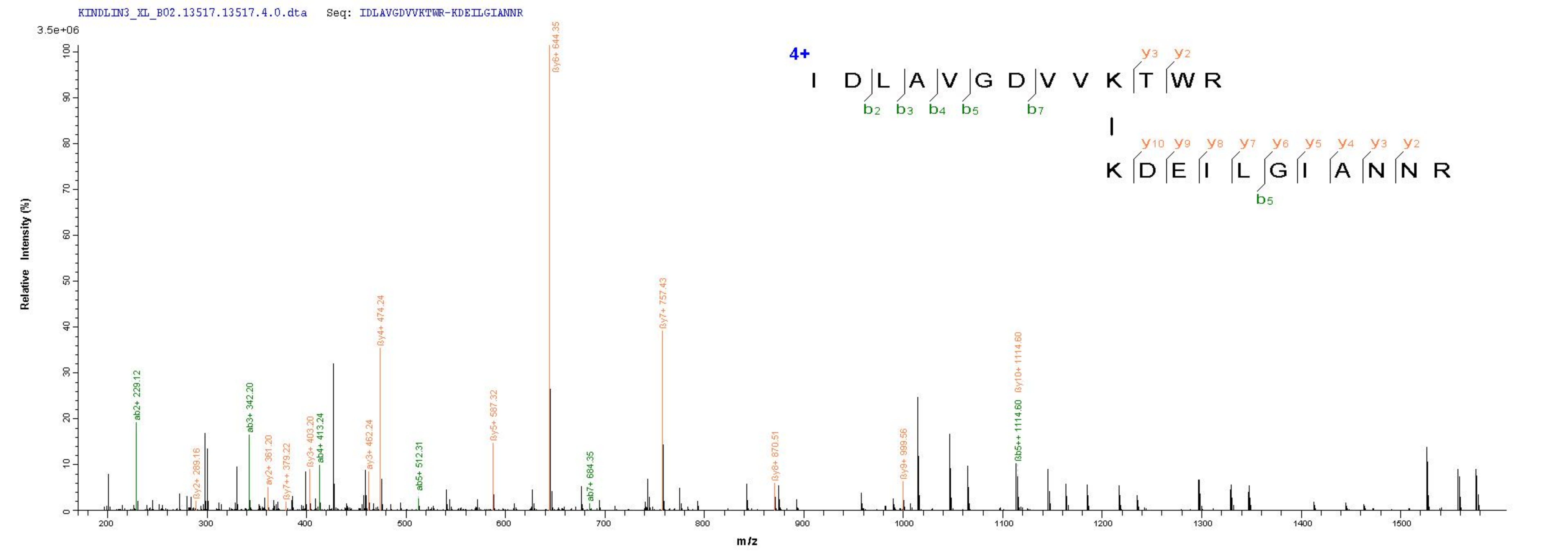

KINDLIN3_XL_B02.13517.13517.4.0.dta Seq: IDLAVGDVVKTWR-KDEILGIANNR
3.5e+06
Relative Intensity (%)
m/z
4+
I D L A V G D V V K T W R
K D E I L G I A N N R
ab2+ 229.12
ßy2+ 289.16
ab3+ 342.20
ay2+ 361.20
ßy7++ 379.22
ßy3+ 403.20
ab4+ 413.24
ay3+ 462.24
ßy4+ 474.24
ab5+ 512.31
ßy5+ 587.32
ßy6+ 644.35
ab7+ 684.35
ßy7+ 757.43
ßy8+ 870.51
ßy9+ 999.56
ßb5++ 1114.60
ßy10+ 1114.60

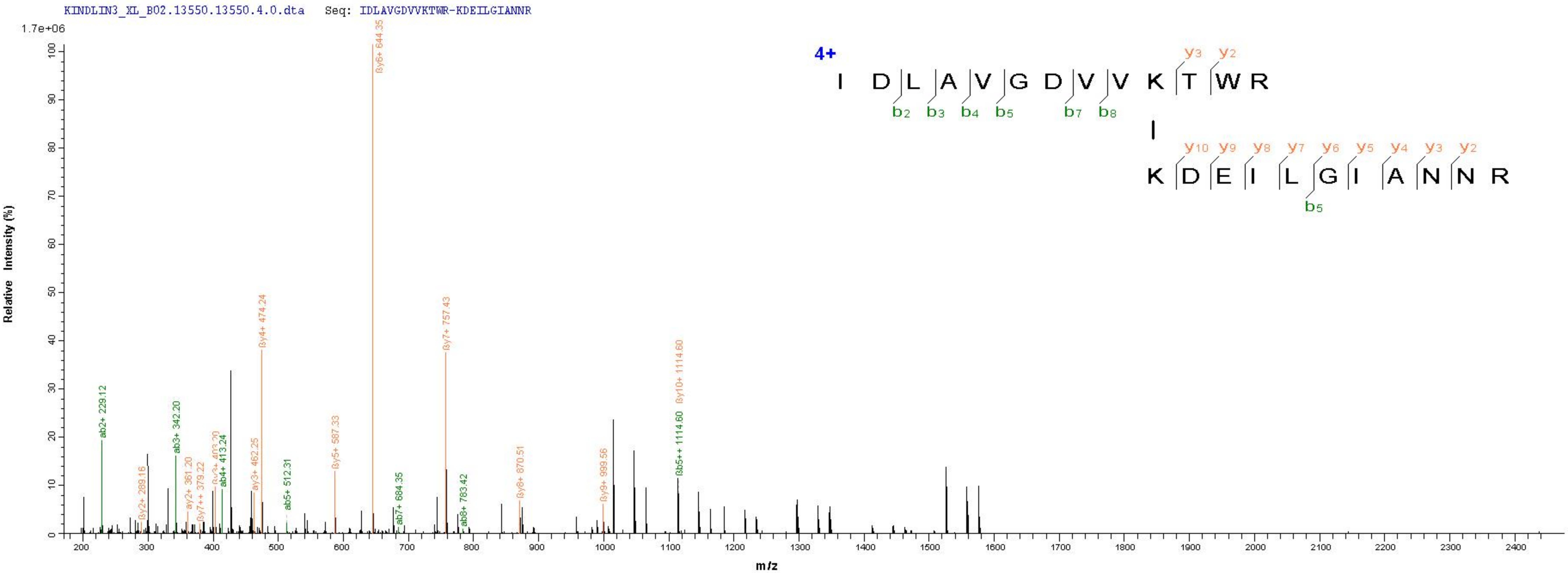
KINDLIN3_XL_B02.13550.13550.4.0.dta Seq: IDLAVGDVVKTWR-KDEILGIANNR
1.7e+06
Relative Intensity (%)
m/z
4+
I D L A V G D V V K T W R
K D E I L G I A N N R
ab2+ 229.12
ab3+ 342.20
ab4+ 413.24
ab5+ 512.31
ab7+ 684.35
ab8+ 783.42
ßy6+ 644.35
ßy4+ 474.24
ßy7+ 757.43
ßy10+ 1114.60
ßb5++ 1114.60
ßy5+ 587.33
ßy8+ 870.51
ßy9+ 999.56
ay3+ 462.25
ay2+ 361.20
ßy2+ 289.16
ßy7++ 379.22

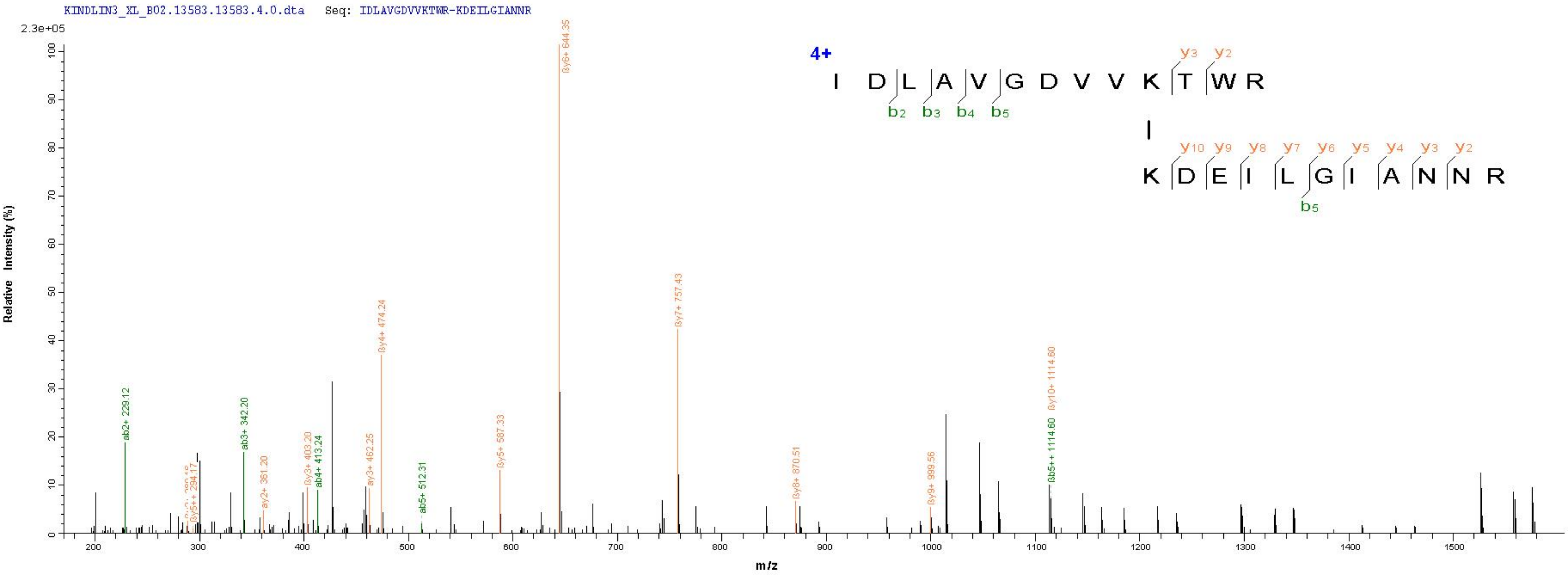

KINDLIN3_XL_B02.13583.13583.4.0.dta Seq: IDLAVGDVVKTWR-KDEILGIANNR
2.3e+05
Relative Intensity (%)
m/z
4+
I D L A V G D V V K T W R
K D E I L G I A N N R
ab2+ 229.12
ßy5++ 294.17
ab3+ 342.20
ay2+ 361.20
ßy3+ 403.20
ab4+ 413.24
ay3+ 462.25
ßy4+ 474.24
ab5+ 512.31
ßy5+ 587.33
ßy6+ 644.35
ßy7+ 757.43
ßy8+ 870.51
ßy9+ 999.56
ßb5++ 1114.60
ßy10+ 1114.60

KINDLIN3_XL_B02.13619.13619.4.0.dta Seq: IDLAVGDVVKTWR-KDEILGIANNR

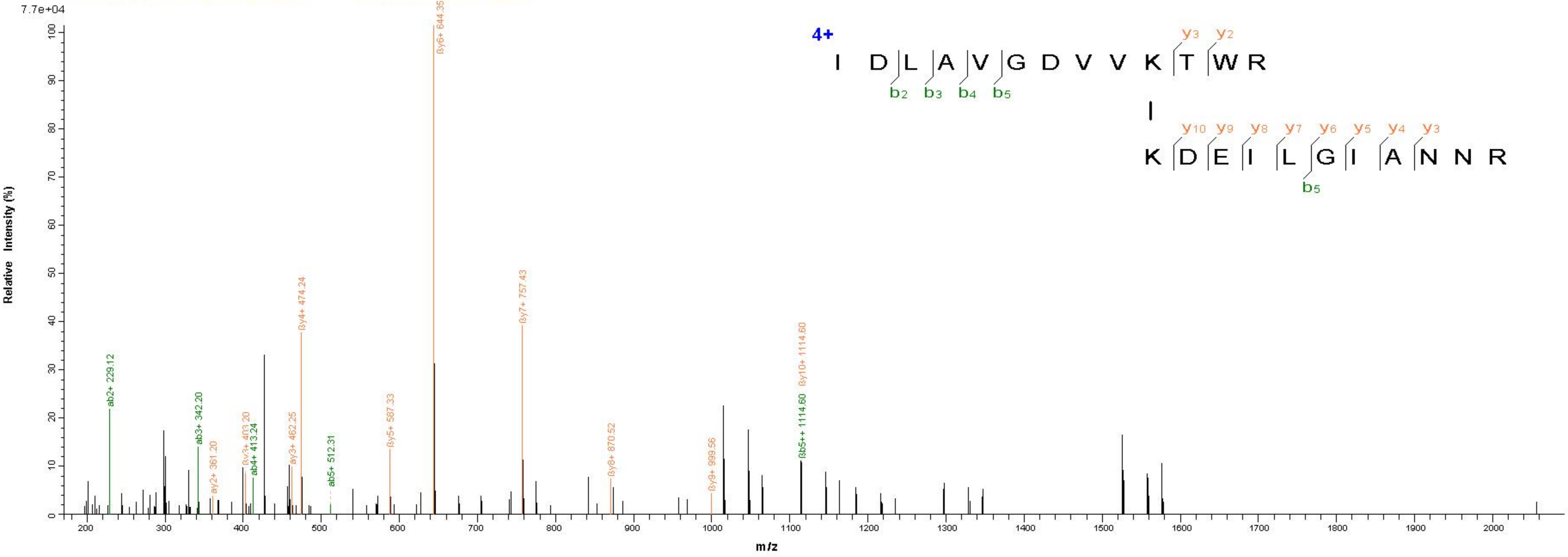

KINDLIN3_XL_B02.13662.13662.4.0.dta   Seq: IDLAVGDVVKTWR-KDEILGIANNR

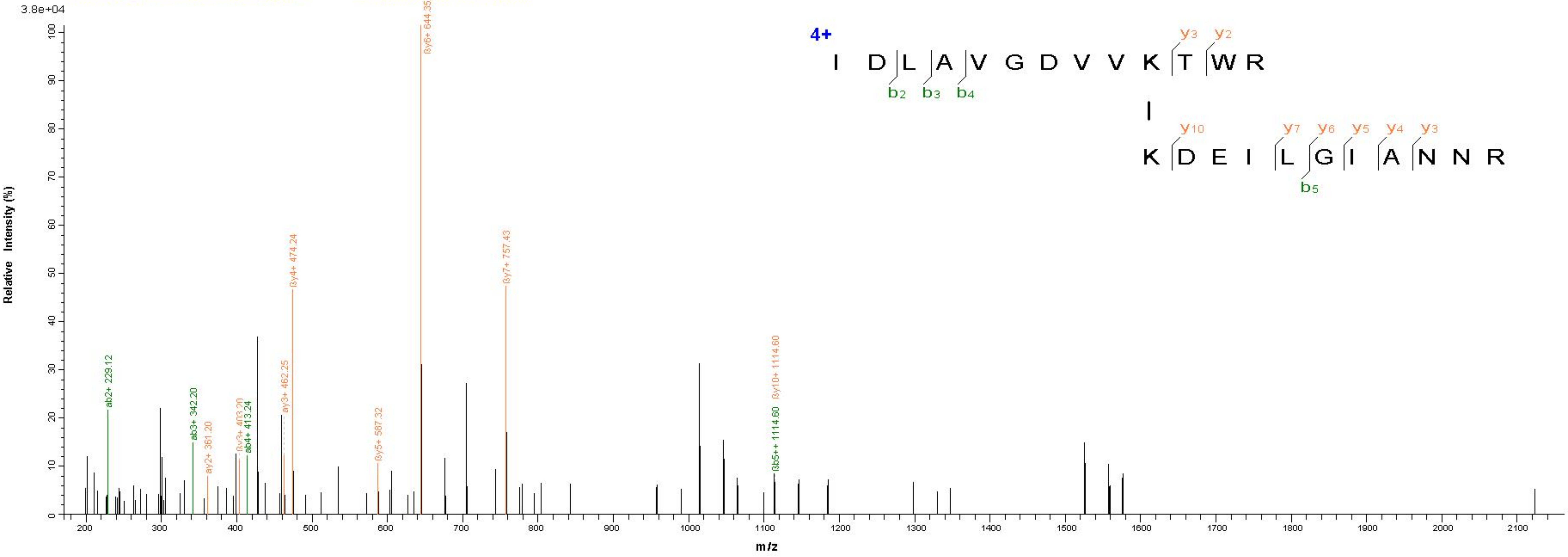

KINDLIN3_XL_B02.13751.13751.4.0.dta Seq: IDLAVGDVVKTWR-KDEILGIANNR

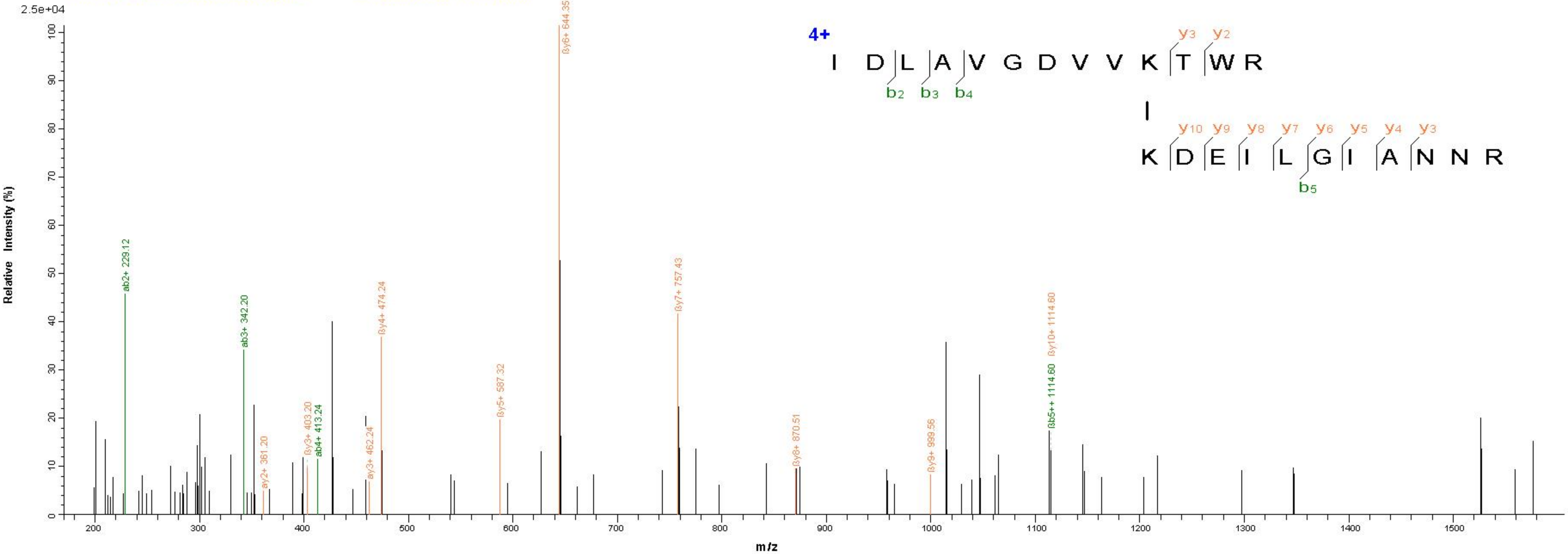

KINDLIN3_XL_B02.13849.13849.4.0.dta Seq: IDLAVGDVVKTWR-KDEILGIANNR Mod: 25,_deamidated[NQ]

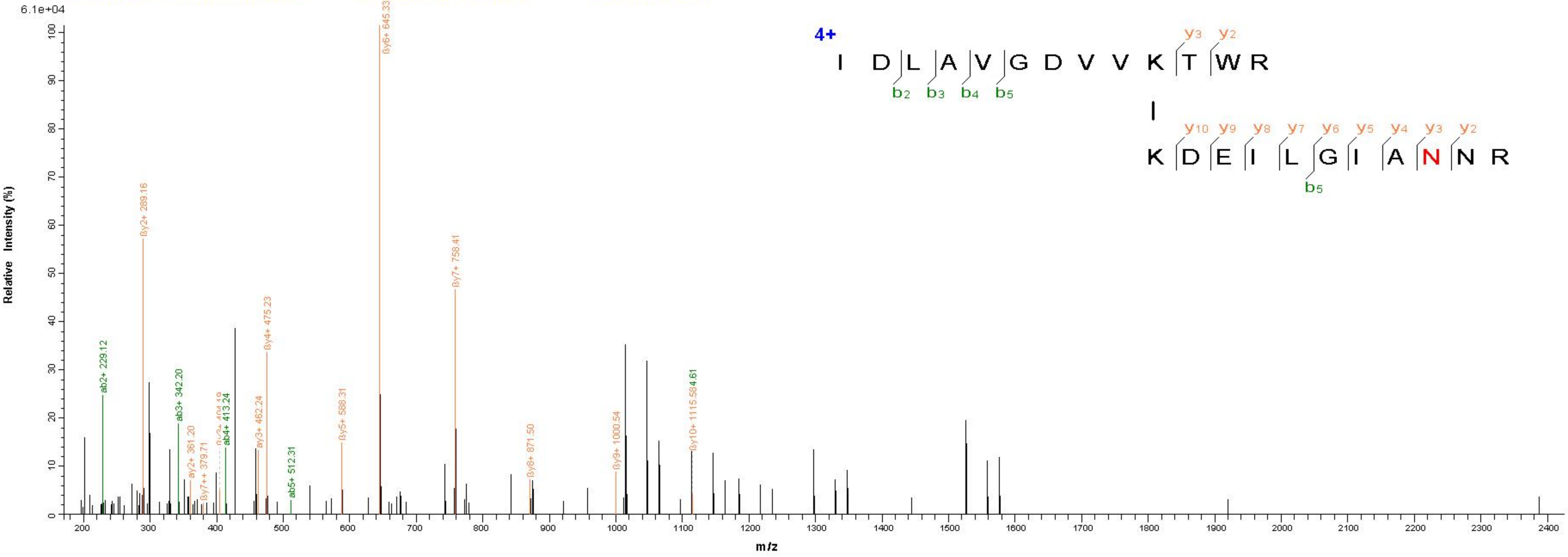

KINDLIN3_XL_B02.13893.13893.4.0.dta Seq: IDLAVGDVVKTWR-KDEILGIANNR Mod: 25,_deamidated[NQ]

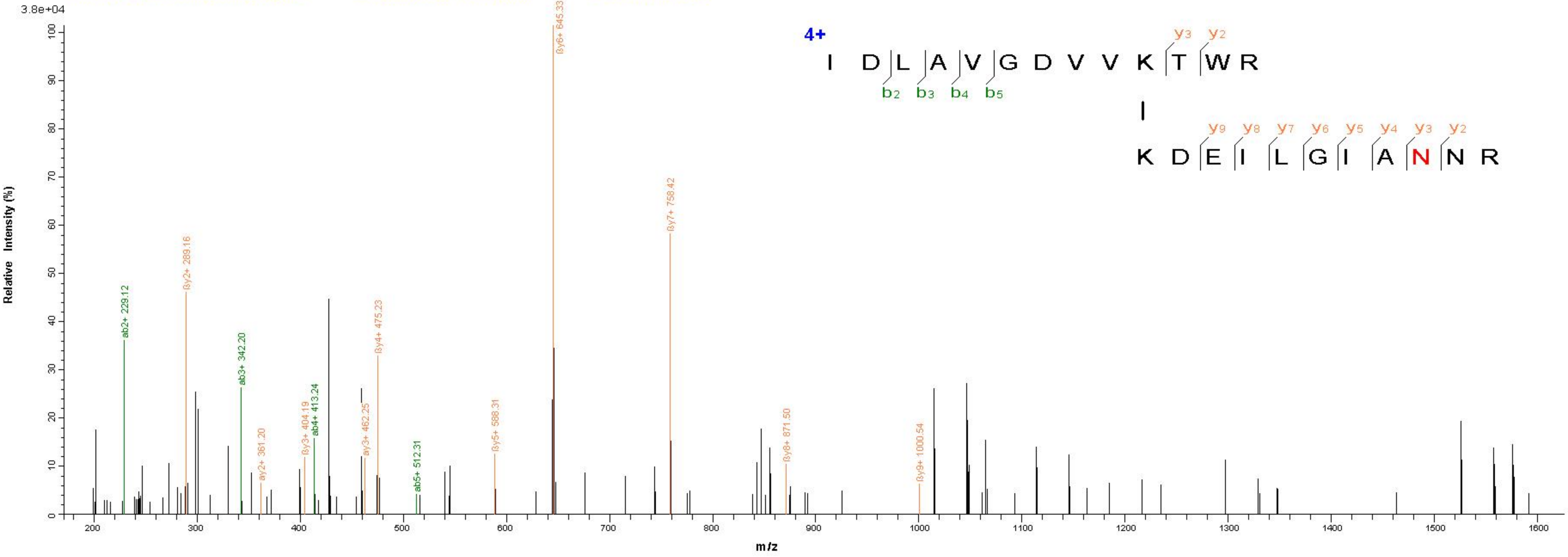

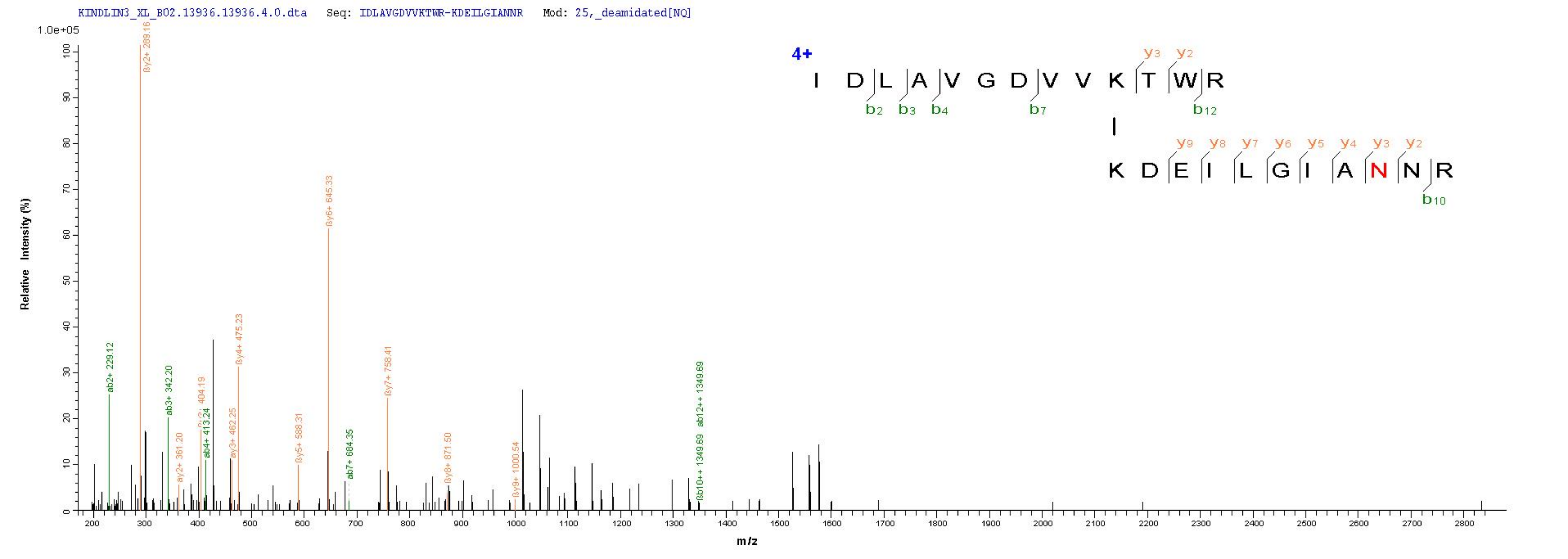

KINDLIN3_XL_B02.13936.13936.4.0.dta Seq: IDLAVGDVVKTWR-KDEILGIANNR Mod: 25,_deamidated[NQ]
1.0e+05
Relative Intensity (%)
m/z
4+
IDLAVGDVVKTWR
KDEILGIANNR
ab2+ 229.12
ßy2+ 289.16
ab3+ 342.20
ay2+ 361.20
404.19
ab4+ 413.24
ay3+ 462.25
ßy4+ 475.23
ßy5+ 588.31
ßy6+ 645.33
ab7+ 684.35
ßy7+ 758.41
ßy8+ 871.50
ßy9+ 1000.54
ßb10++ 1349.69
ab12++ 1349.69

KINDLIN3_XL_B02.13979.13979.4.0.dta Seq: IDLAVGDVVKTWR-KDEILGIANNR Mod: 25,_deamidated[NQ]

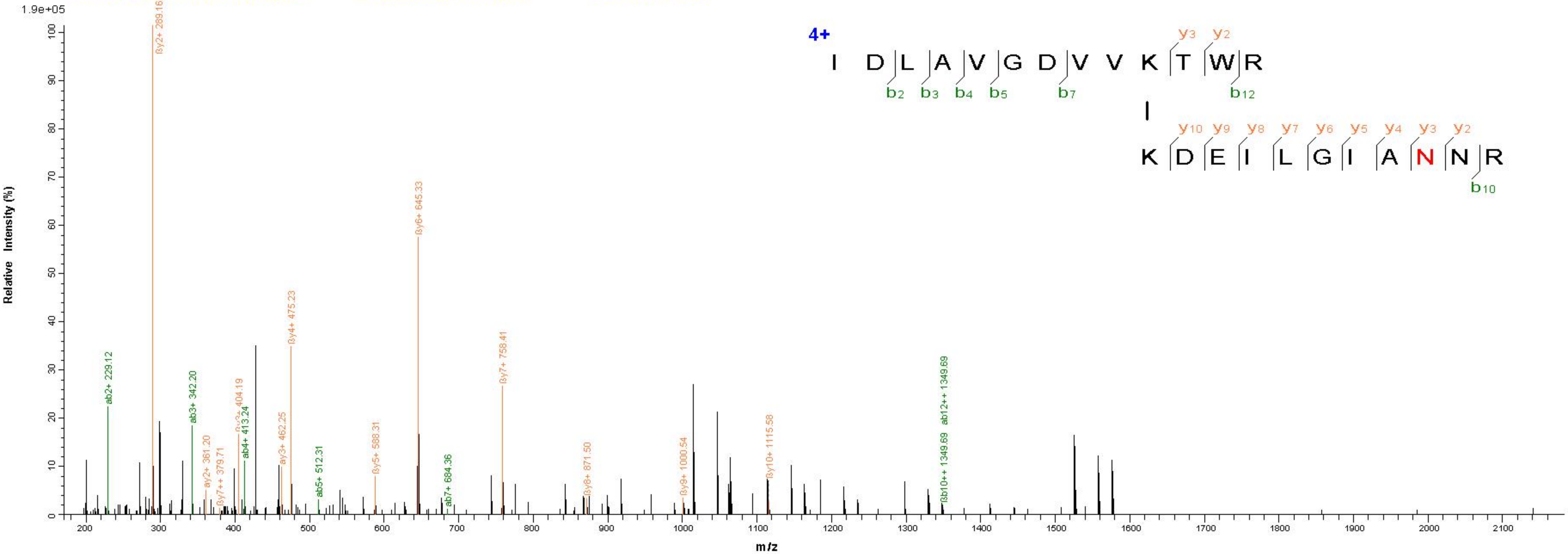

KINDLIN3_XL_B02.14012.14012.4.0.dta Seq: IDLAVGDVVKTWR-KDEILGIANNR Mod: 25,_deamidated[NQ]

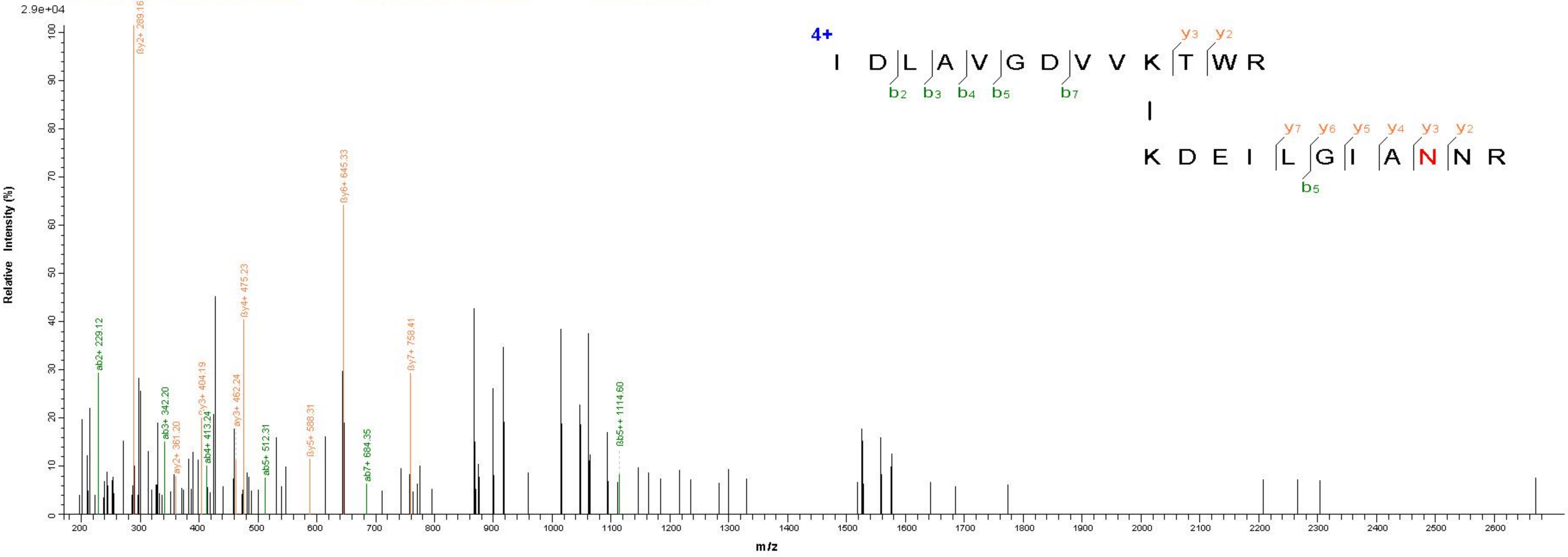

KINDLIN3_XL_B03.13446.13446.4.0.dta   Seq: IDLAVGDVVKTWR-KDEILGIANNR

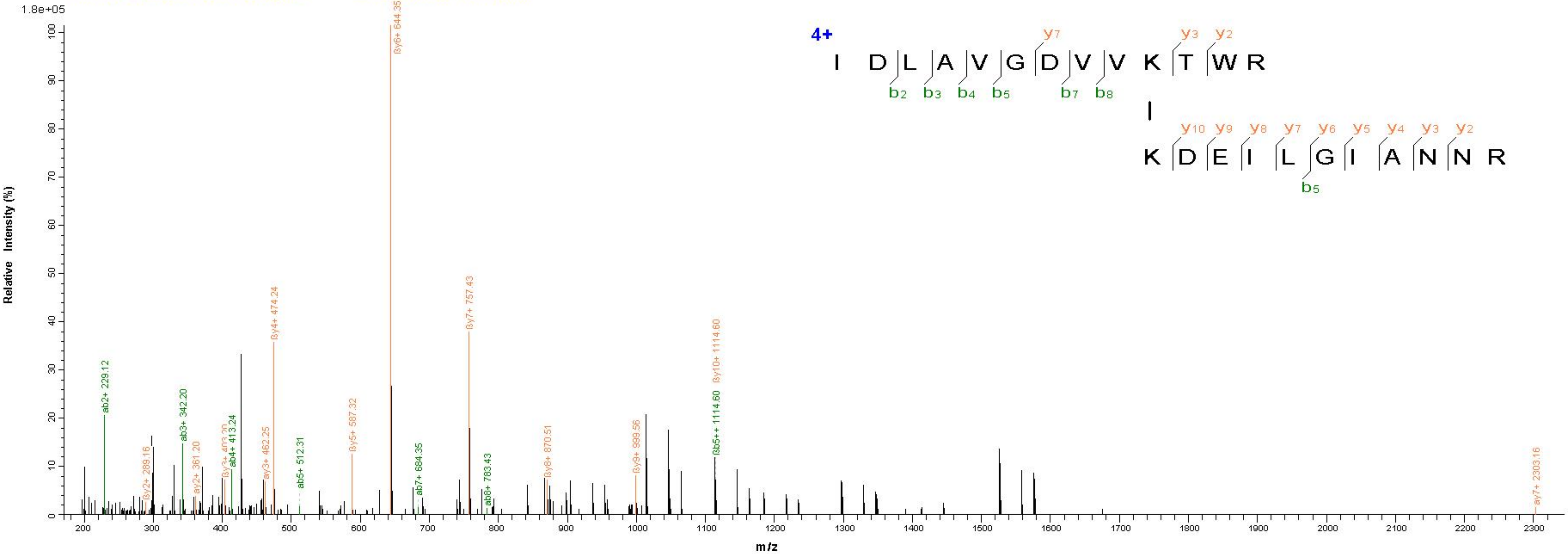

KINDLIN3_XL_B03.13463.13463.3.1.dta   Seq: IDLAVGDVVKTWR-KDEILGIANNR

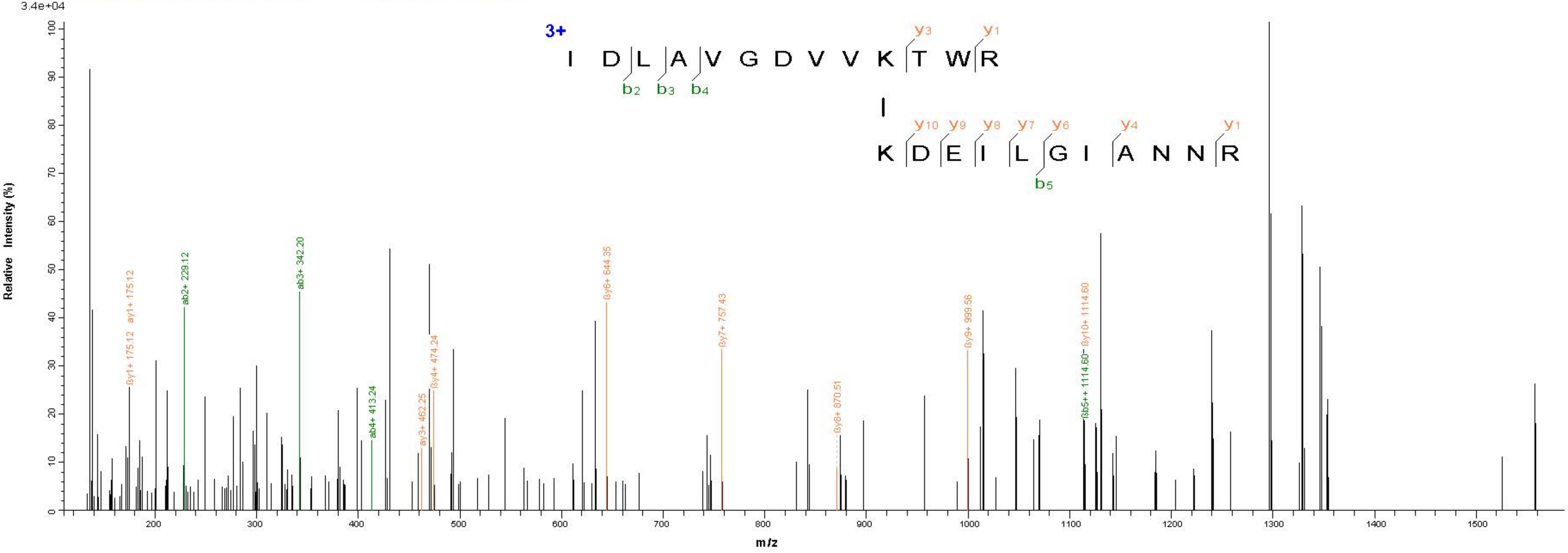

KINDLIN3_XL_B03.13469.13469.3.0.dta Seq: IDLAVGDVVKTWR-KDEILGIANNR

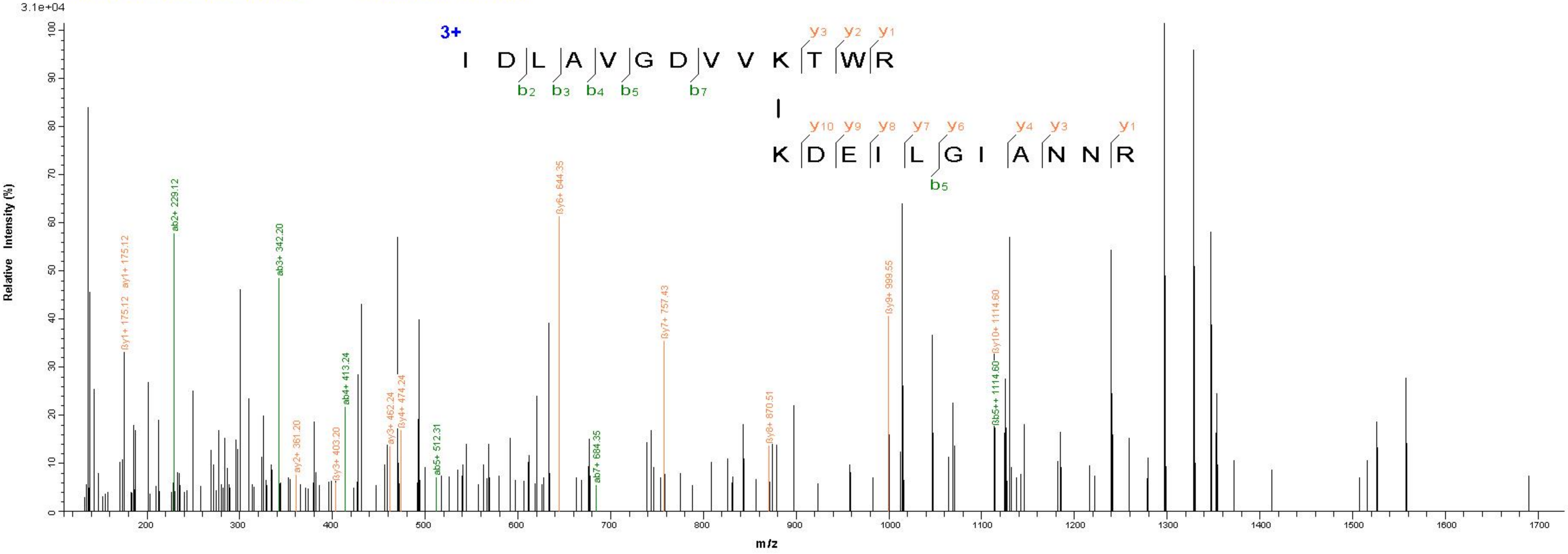

KINDLIN3_XL_B03.13470.13470.3.1.dta Seq: IDLAVGDVVKTWR-KDEILGIANNR

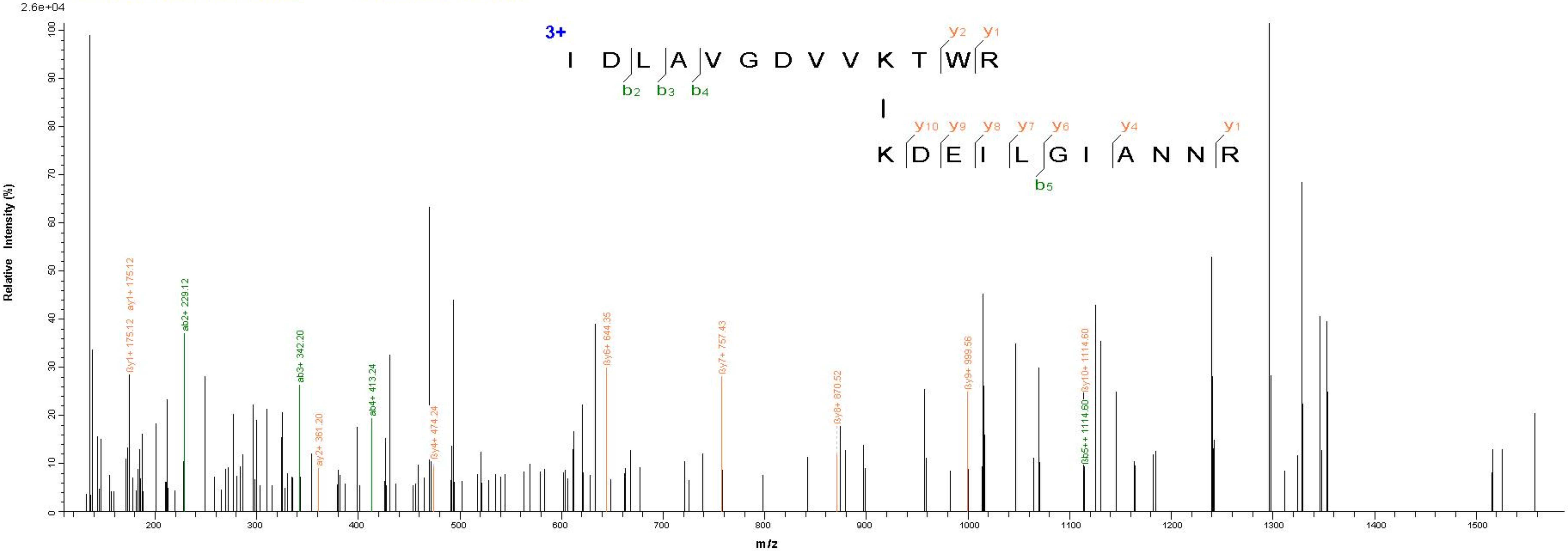

KINDLIN3_XL_B03.13471.13471.3.1.dta Seq: IDLAVGDVVKTWR-KDEILGIANNR

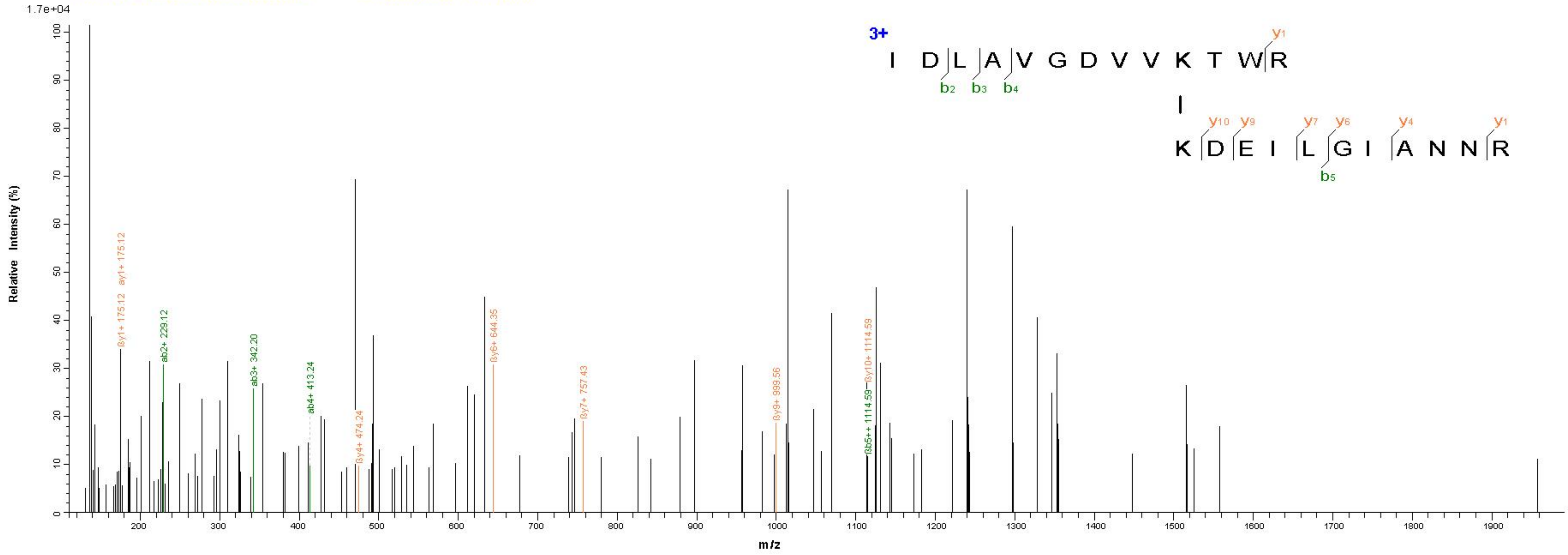

KINDLIN3_XL_B03.13472.13472.3.0.dta Seq: IDLAVGDVVKTWR-KDEILGIANNR

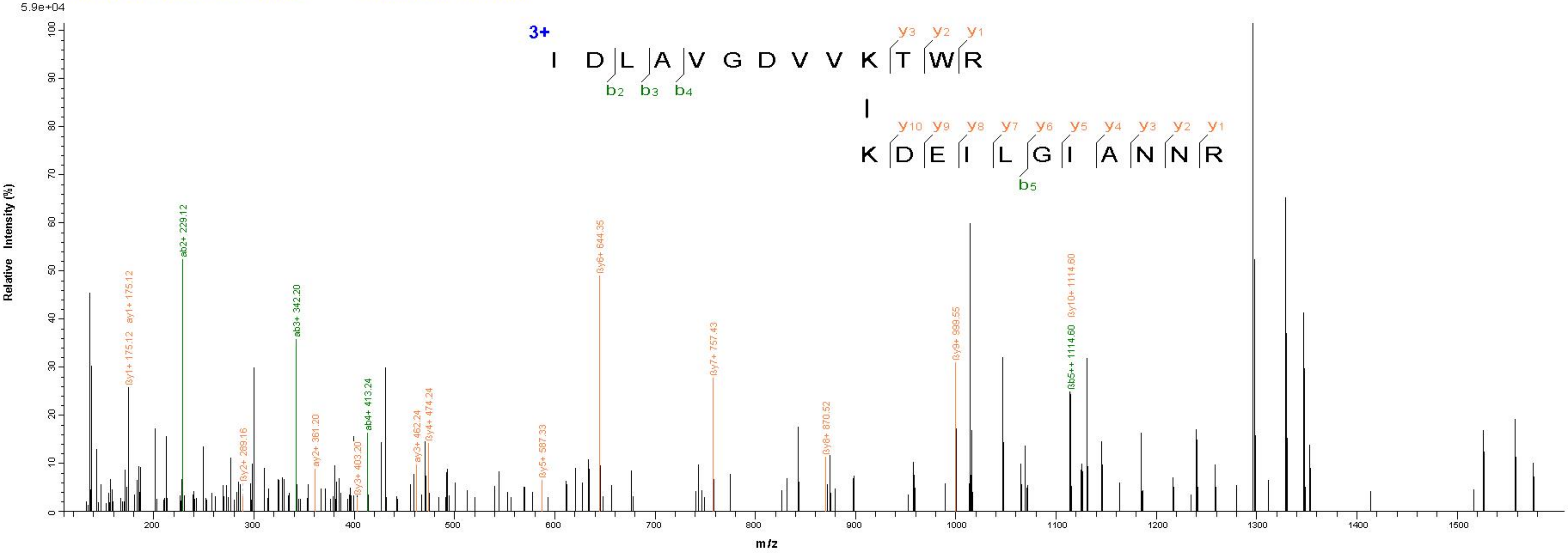

KINDLIN3_XL_B03.13488.13488.4.0.dta   Seq: IDLAVGDVVKTWR-KDEILGIANNR

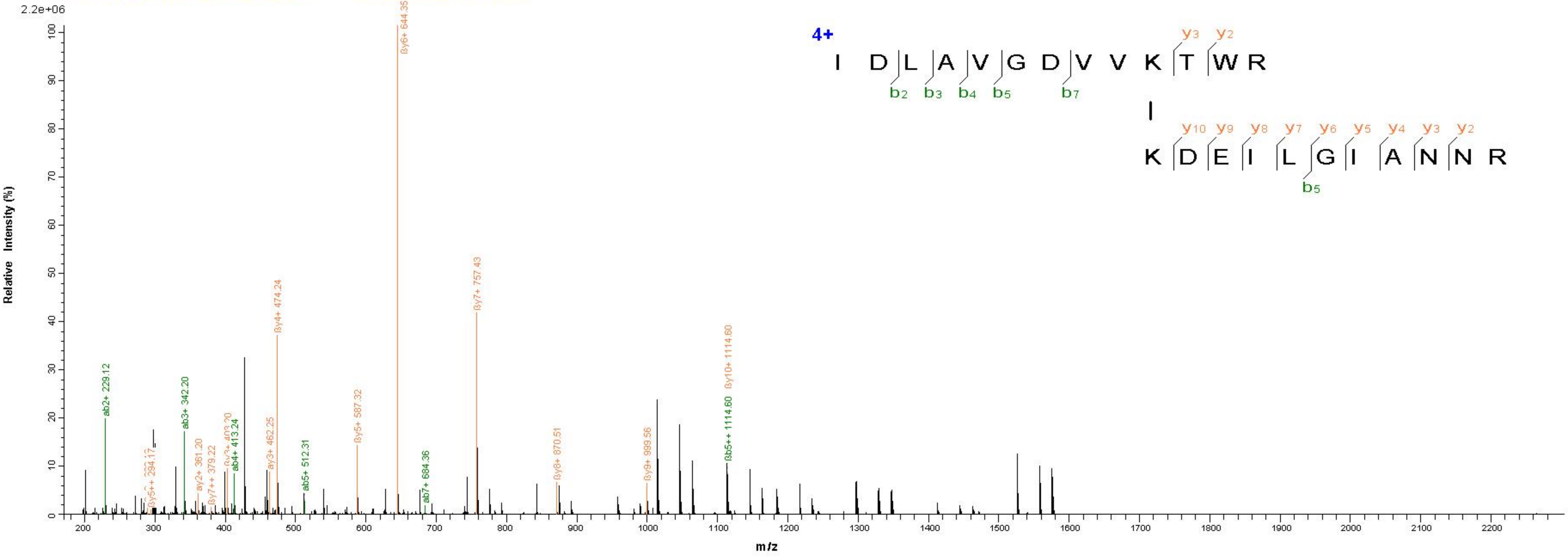

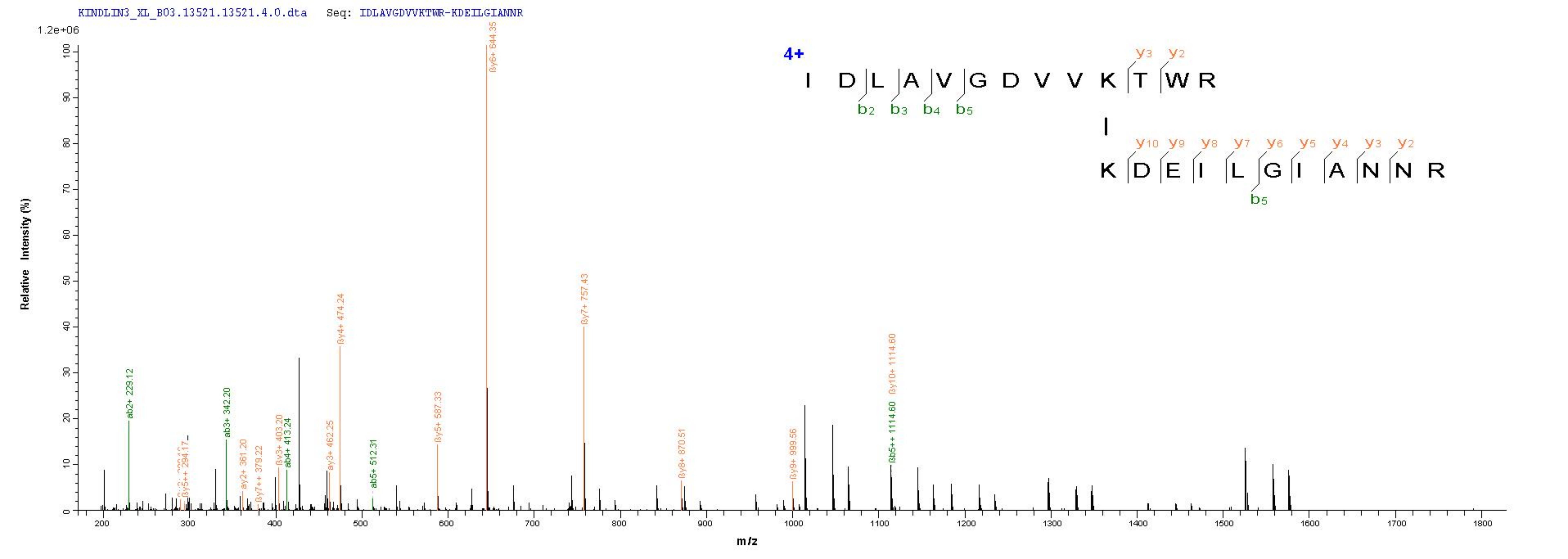

KINDLIN3_XL_B03.13521.13521.4.0.dta Seq: IDLAVGDVVKTWR-KDEILGIANNR
1.2e+06
Relative Intensity (%)
m/z
4+
I D L A V G D V V K T W R
K D E I L G I A N N R
ab2+ 229.12
ßy5++ 294.17
ab3+ 342.20
ay2+ 361.20
ßy7++ 379.22
ßy3+ 403.20
ab4+ 413.24
ay3+ 462.25
ßy4+ 474.24
ab5+ 512.31
ßy5+ 587.33
ßy6+ 644.35
ßy7+ 757.43
ßy8+ 870.51
ßy9+ 999.56
ßb5++ 1114.60
ßy10+ 1114.60

KINDLIN3_XL_B03.13554.13554.4.0.dta Seq: IDLAVGDVVKTWR-KDEILGIANNR

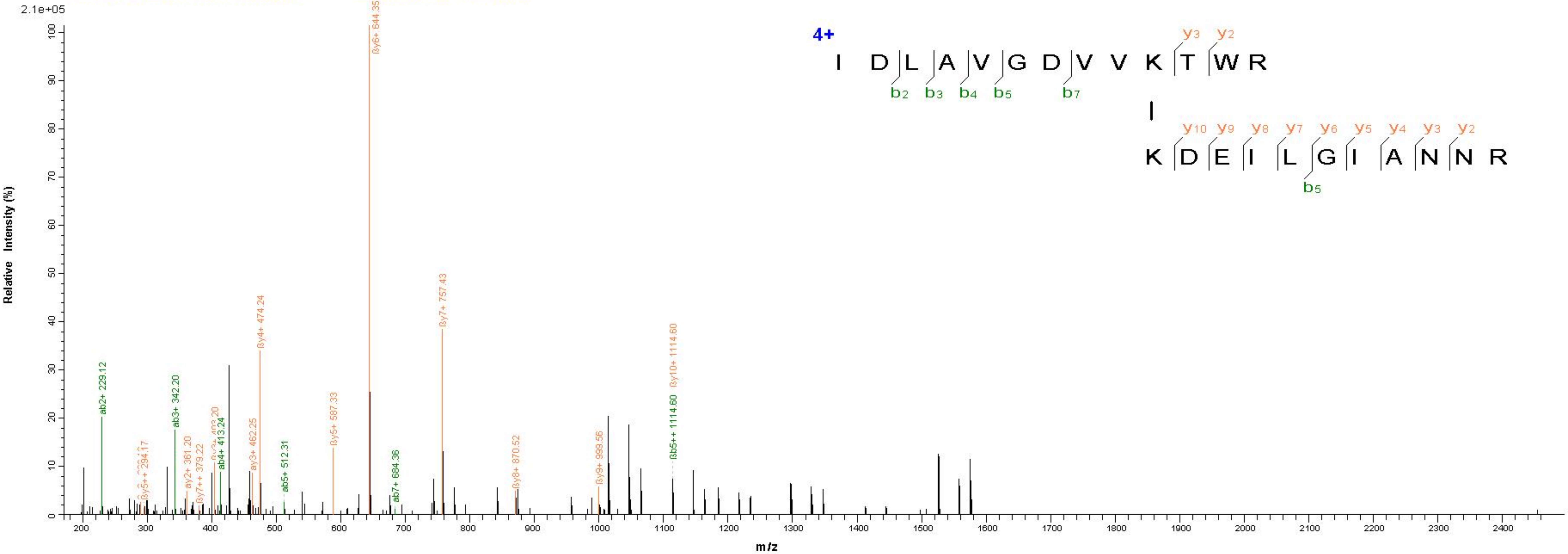

KINDLIN3_XL_B03.13587.13587.4.1.dta Seq: IDLAVGDVVKTWR-KDEILGIANNR

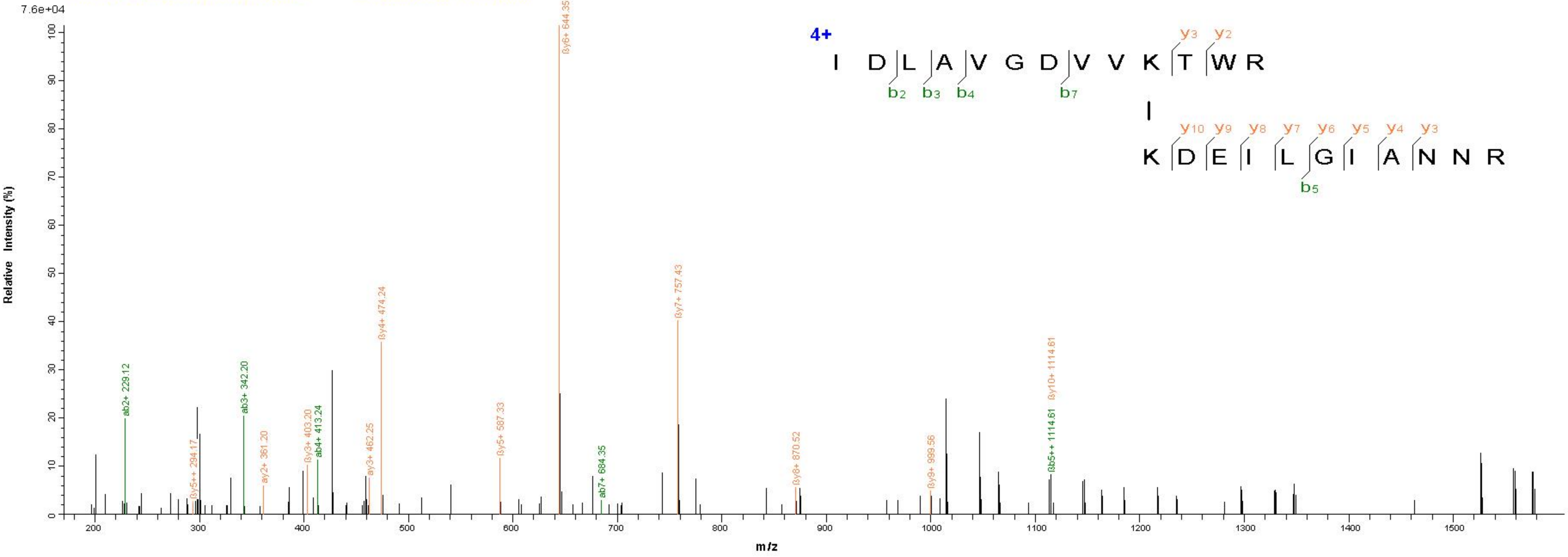

KINDLIN3_XL_B03.13621.13621.4.0.dta Seq: IDLAVGDVVKTWR-KDEILGIANNR

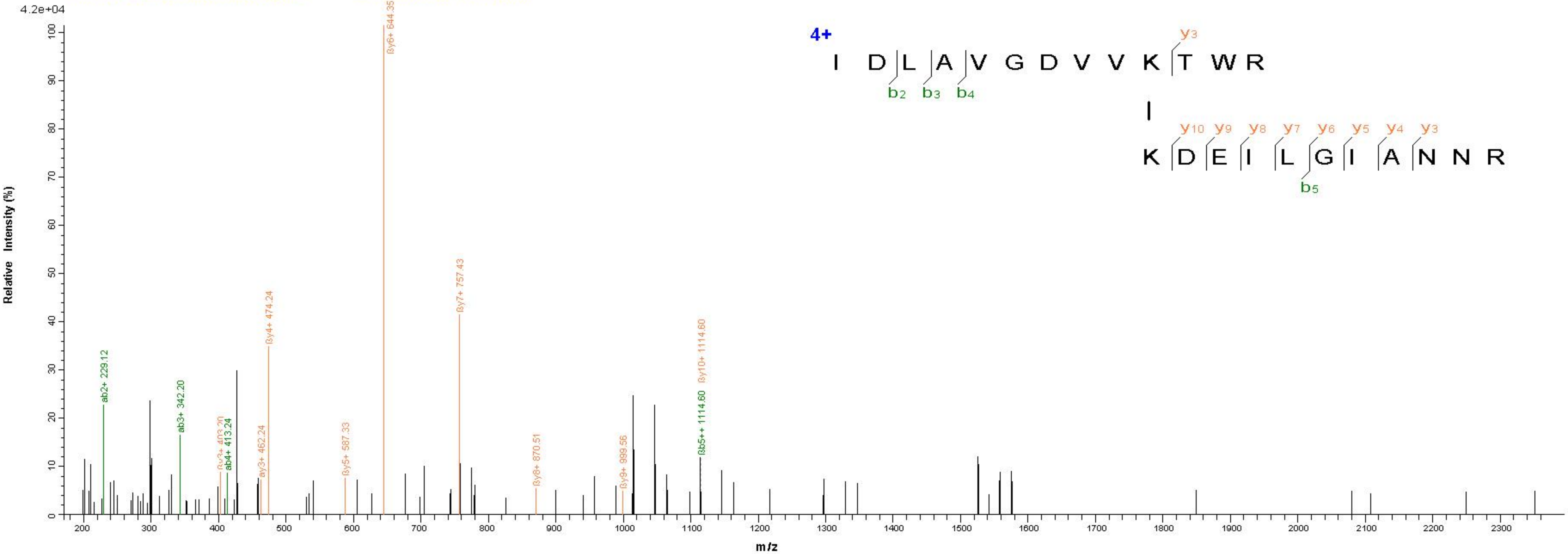

KINDLIN3_XL_B03.13669.13669.4.0.dta Seq: IDLAVGDVVKTWR-KDEILGIANNR

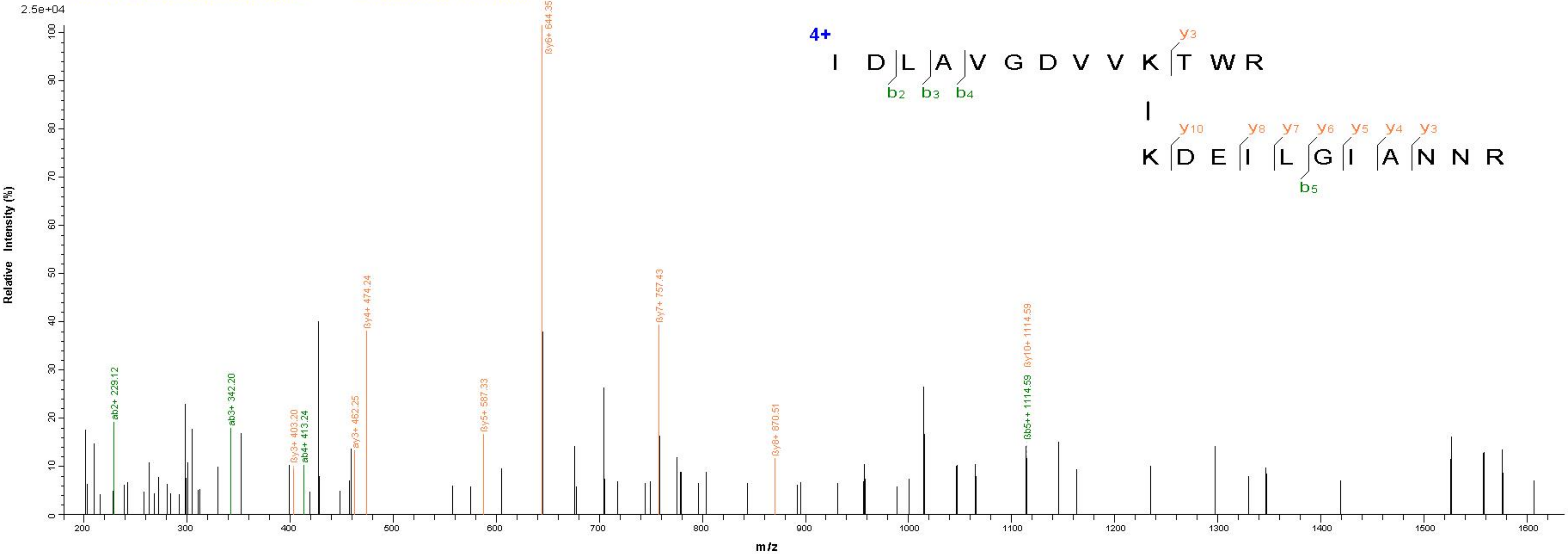

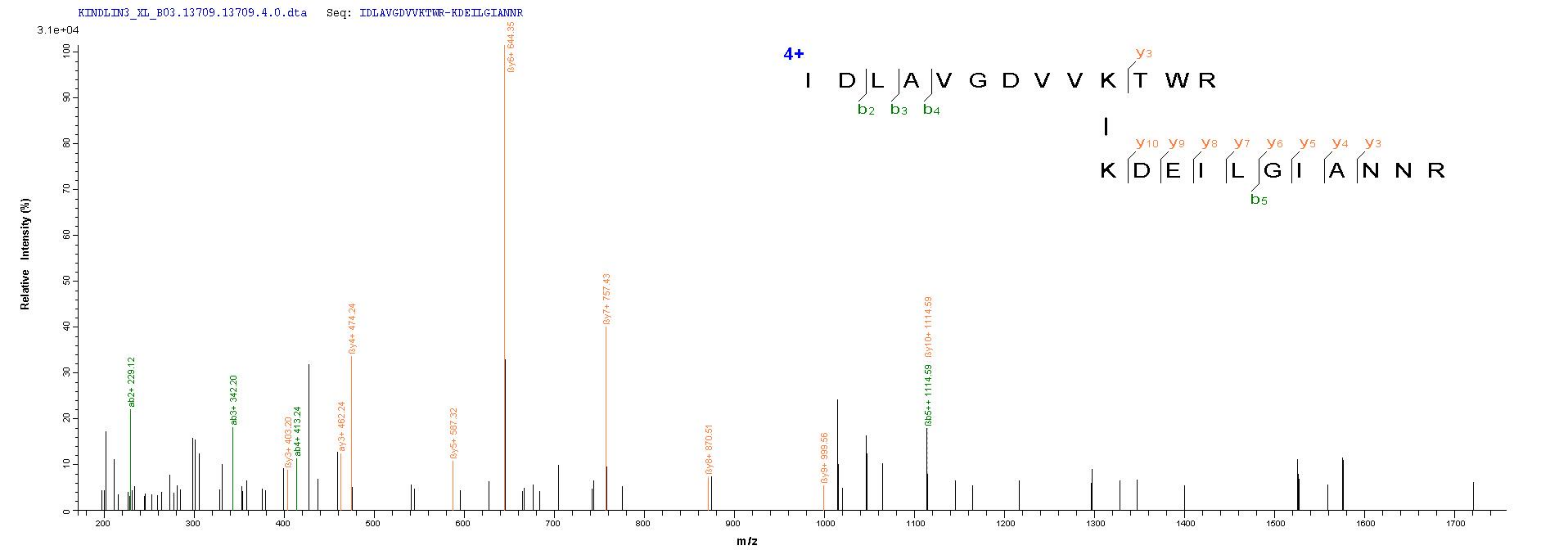

KINDLIN3_XL_B03.13709.13709.4.0.dta Seq: IDLAVGDVVKTWR-KDEILGIANNR
3.1e+04
4+
I D L A V G D V V K T W R
K D E I L G I A N N R
Relative Intensity (%)
m/z
ßy6+ 644.35
ßy7+ 757.43
ßy4+ 474.24
ßy10+ 1114.59
ßb5++ 1114.59
ab2+ 229.12
ab3+ 342.20
ab4+ 413.24
ßy3+ 403.20
ay3+ 462.24
ßy5+ 587.32
ßy8+ 870.51
ßy9+ 999.56

KINDLIN3_XL_B03.13835.13835.4.0.dta   Seq: IDLAVGDVVKTWR-KDEILGIANNR   Mod: 25,_deamidated[NQ]

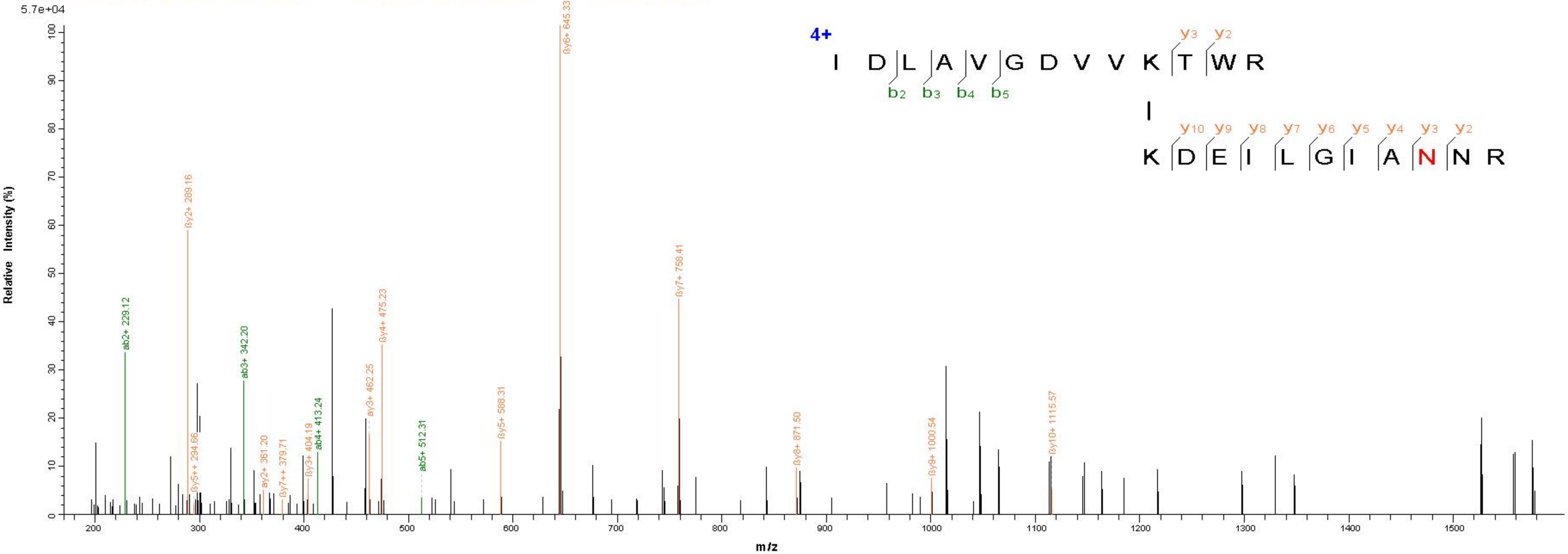

KINDLIN3_XL_B03.13897.13897.4.0.dta Seq: IDLAVGDVVKTWR-KDEILGIANNR Mod: 25,_deamidated[NQ]

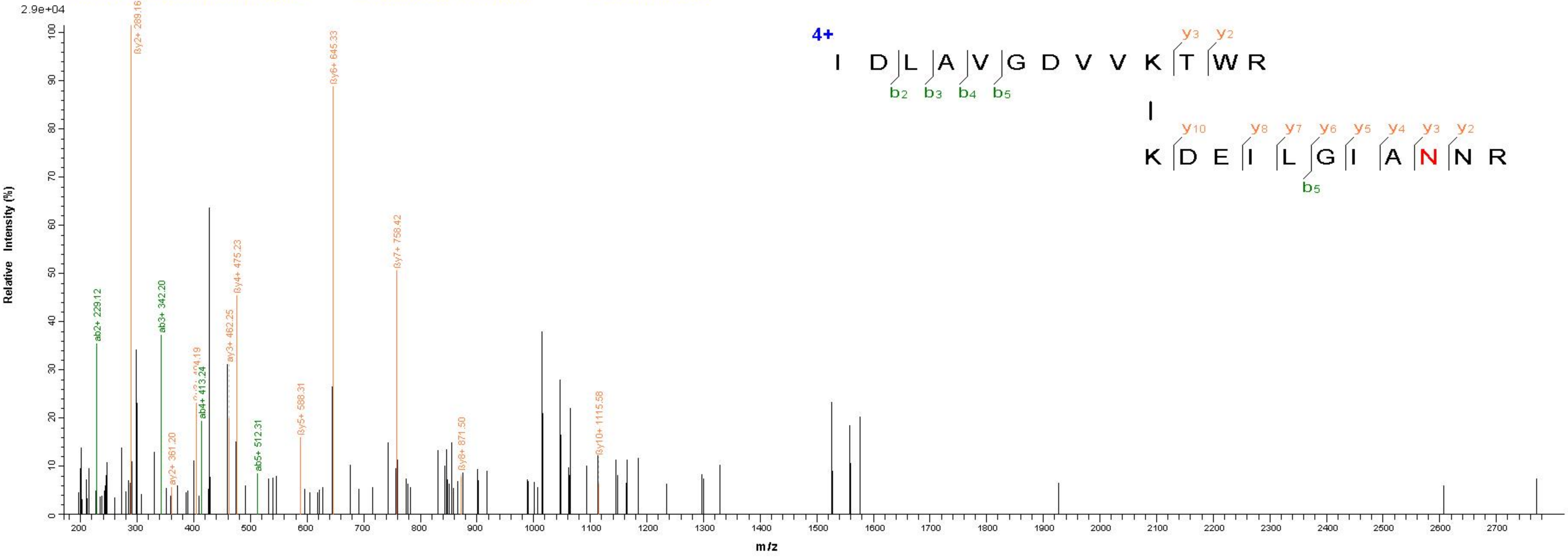

KINDLIN3_XL_B03.13939.13939.4.0.dta Seq: IDLAVGDVVKTWR-KDEILGIANNR Mod: 25,_deamidated[NQ]

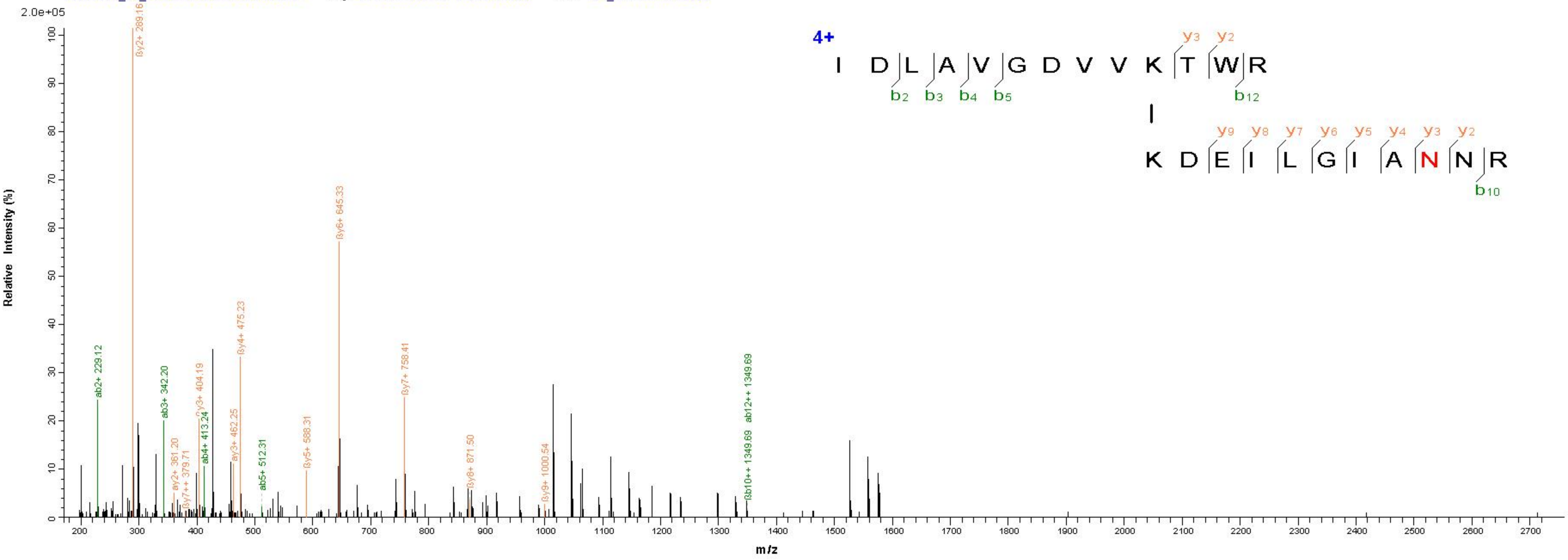

KINDLIN3_XL_B03.13972.13972.4.0.dta Seq: IDLAVGDVVKTWR-KDEILGIANNR Mod: 25,_deamidated[NQ]

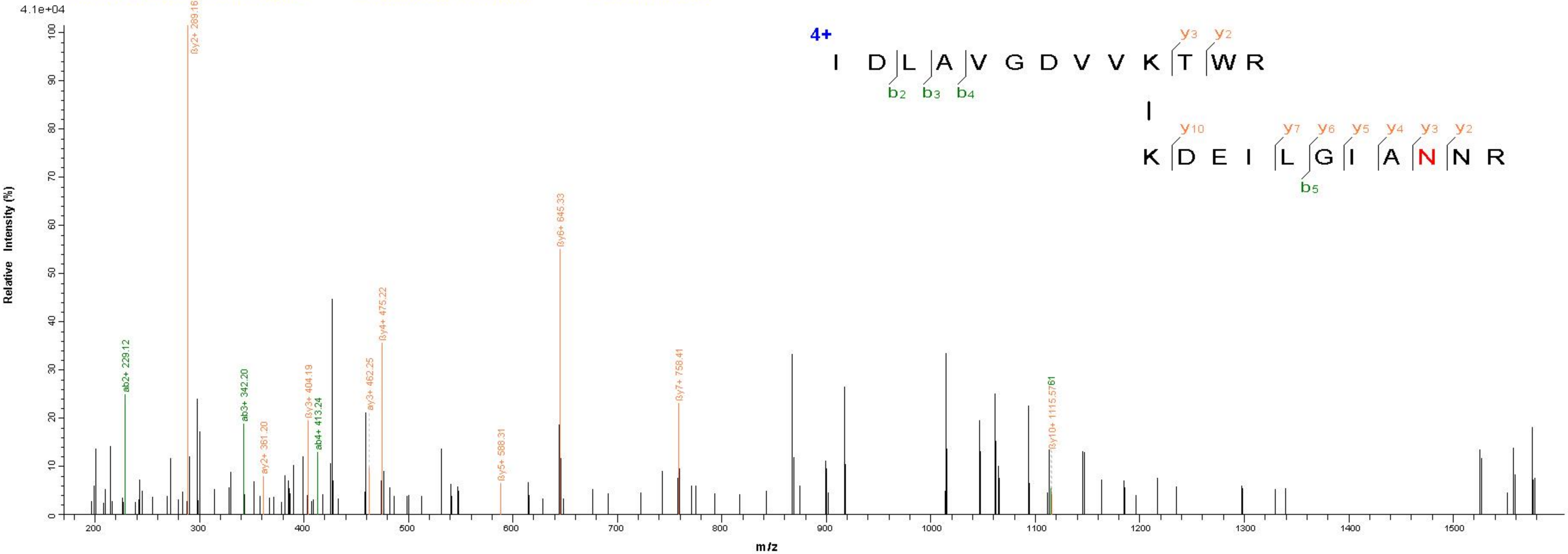

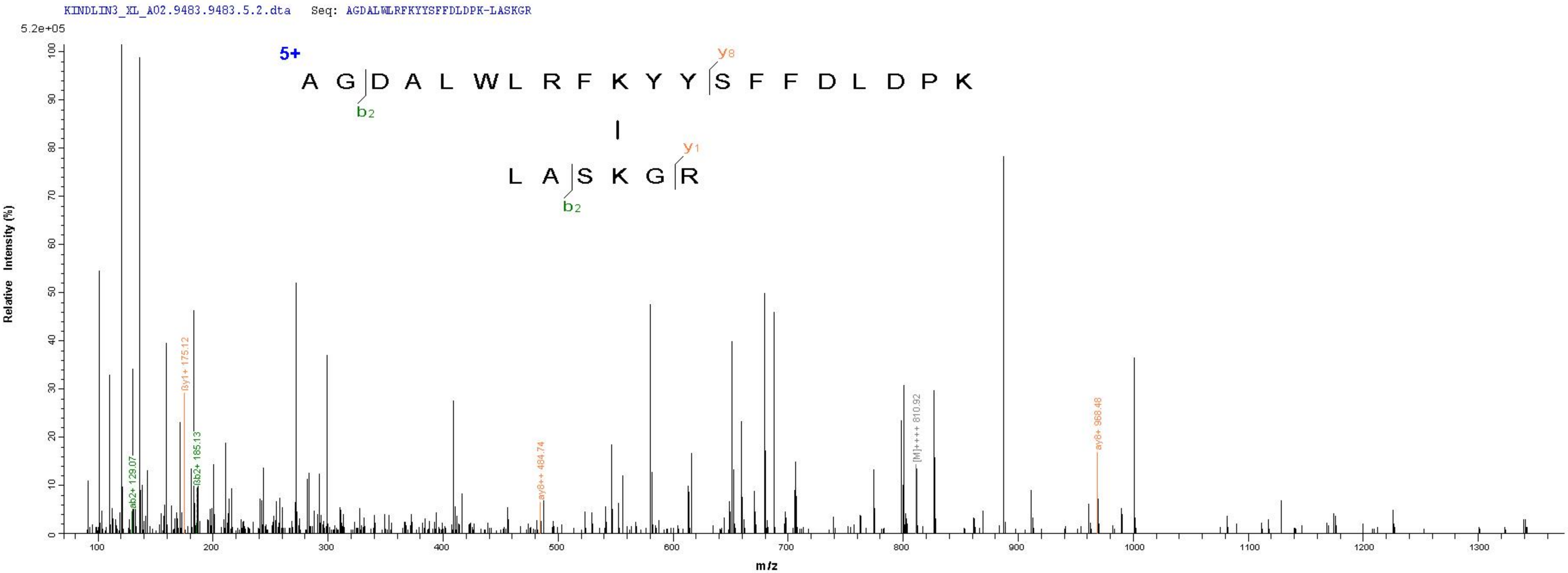

KINDLIN3_XL_A02.9483.9483.5.2.dta Seq: AGDALWLRFKYYSFFDLDPK-LASKGR
5.2e+05
5+
A G D A L W L R F K Y Y S F F D L D P K
L A S K G R
y8
b2
y1
b2
Relative Intensity (%)
m/z
ab2+ 129.07
ßy1+ 175.12
ßb2+ 185.13
ay8++ 484.74
[M]++++ 810.92
ay8+ 968.48

KINDLIN3_XL_A02.9486.9486.5.1.dta Seq: AGDALWLRFKYYSFFDLDPK-LASKGR

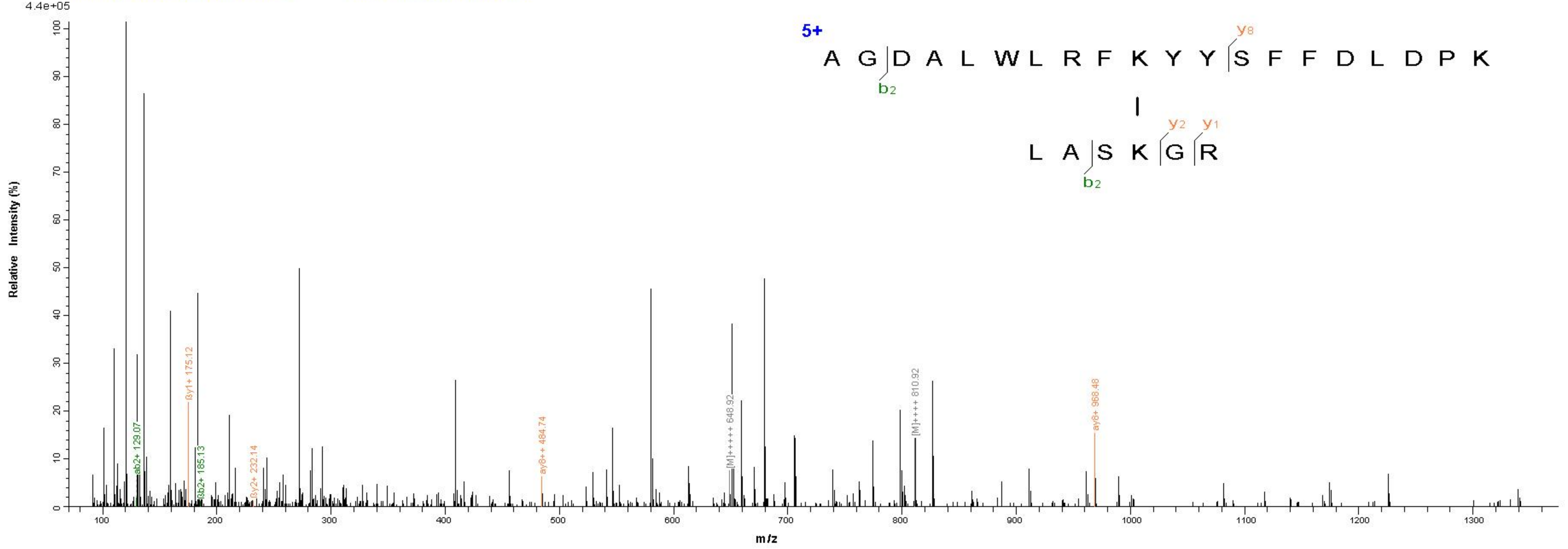

KINDLIN3_XL_A02.9545.9545.6.1.dta   Seq: AGDALWLRFKYYSFFDLDPK-LASKGR

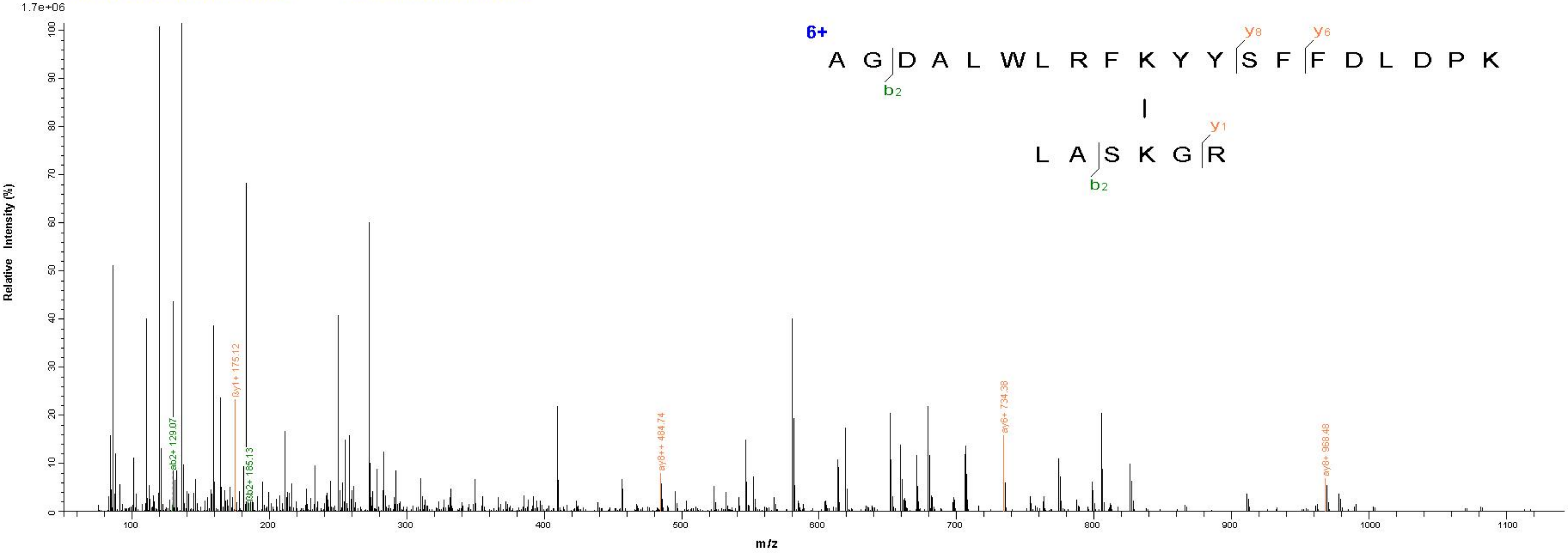

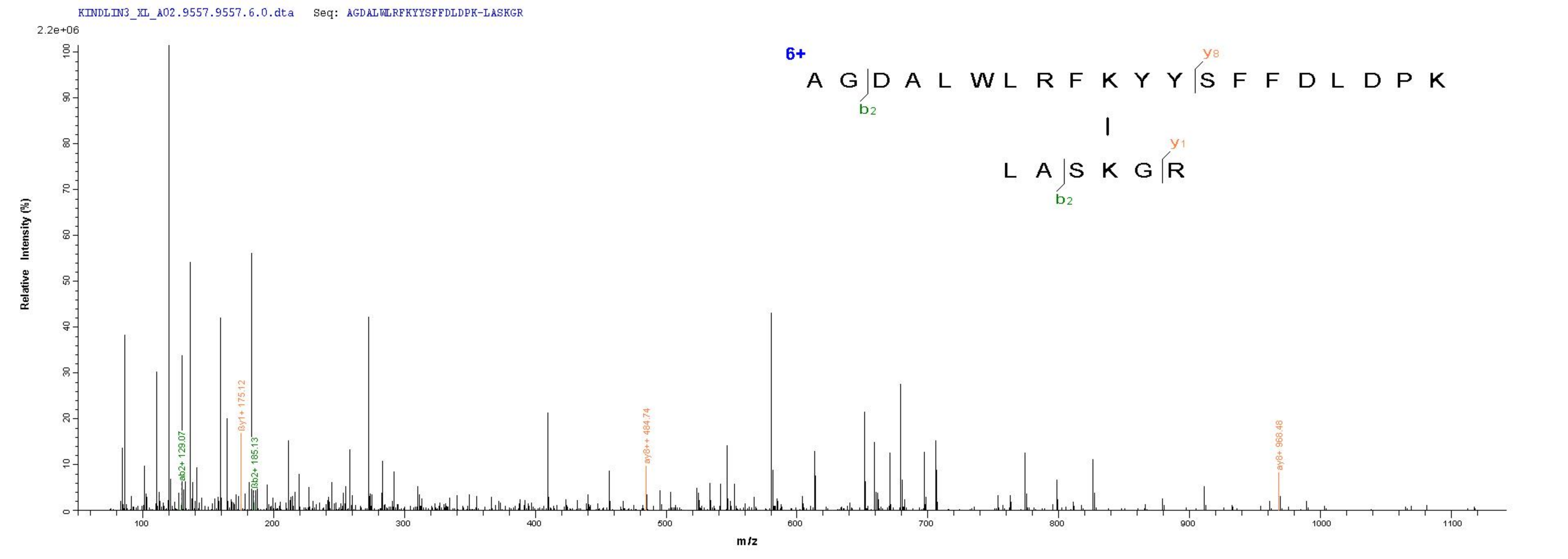
KINDLIN3_XL_A02.9557.9557.6.0.dta Seq: AGDALWLRFKYYSFFDLDPK-LASKGR
2.2e+06
Relative Intensity (%)
m/z
6+
A G D A L W L R F K Y Y S F F D L D P K
L A S K G R
b2
y8
y1
b2
ab2+ 129.07
ßy1+ 175.12
ßb2+ 185.13
ay8++ 484.74
ay8+ 968.48

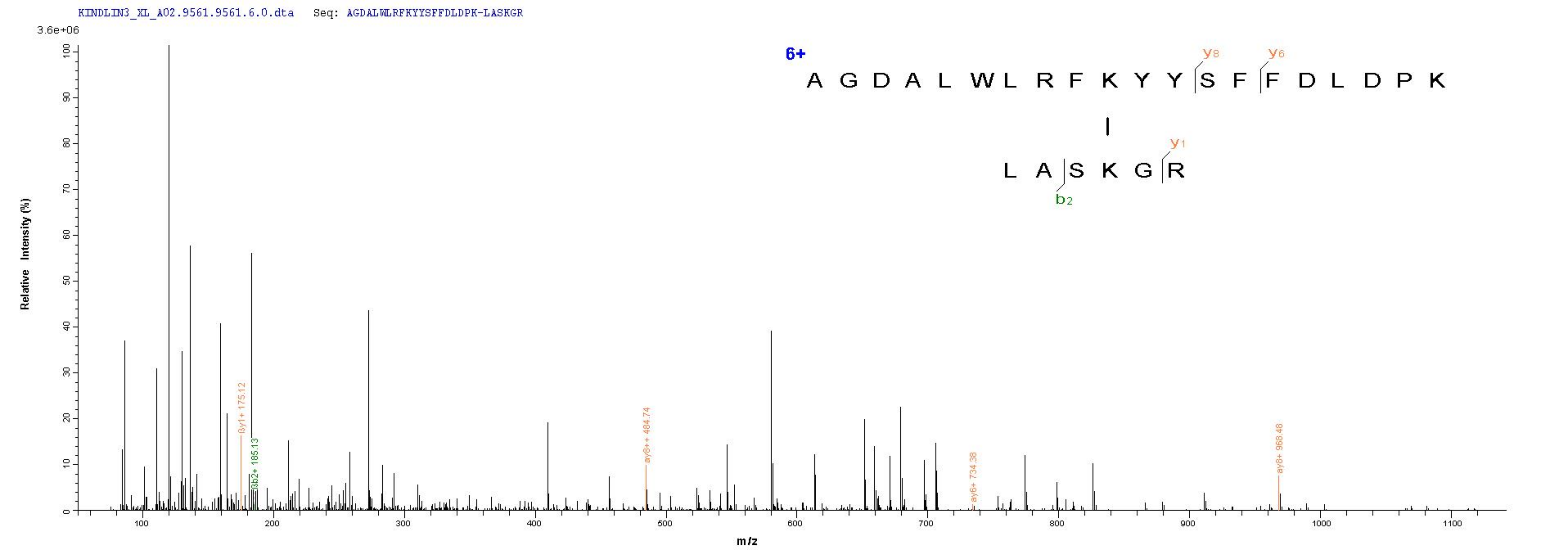
KINDLIN3_XL_A02.9561.9561.6.0.dta Seq: AGDALWLRFKYYSFFDLDPK-LASKGR
3.6e+06
Relative Intensity (%)
m/z
6+
A G D A L W L R F K Y Y S F F D L D P K
L A S K G R
y8
y6
y1
b2
βy1+ 175.12
βb2+ 185.13
αy8++ 484.74
αy6+ 734.38
αy8+ 968.48

KINDLIN3_XL_A02.9574.9574.6.1.dta Seq: AGDALWLRFKYYSFFDLDPK-LASKGR

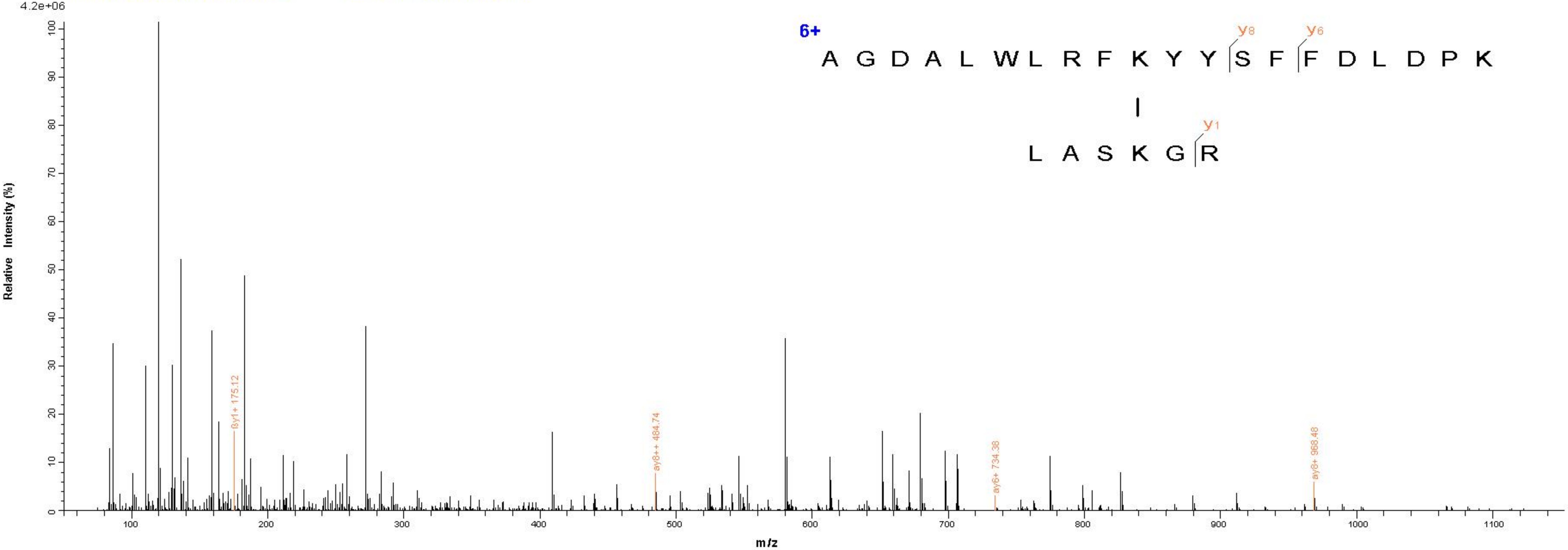

KINDLIN3_XL_B01.9603.9603.6.4.dta Seq: AGDALWLRFKYYSFFDLDPK-LASKGR

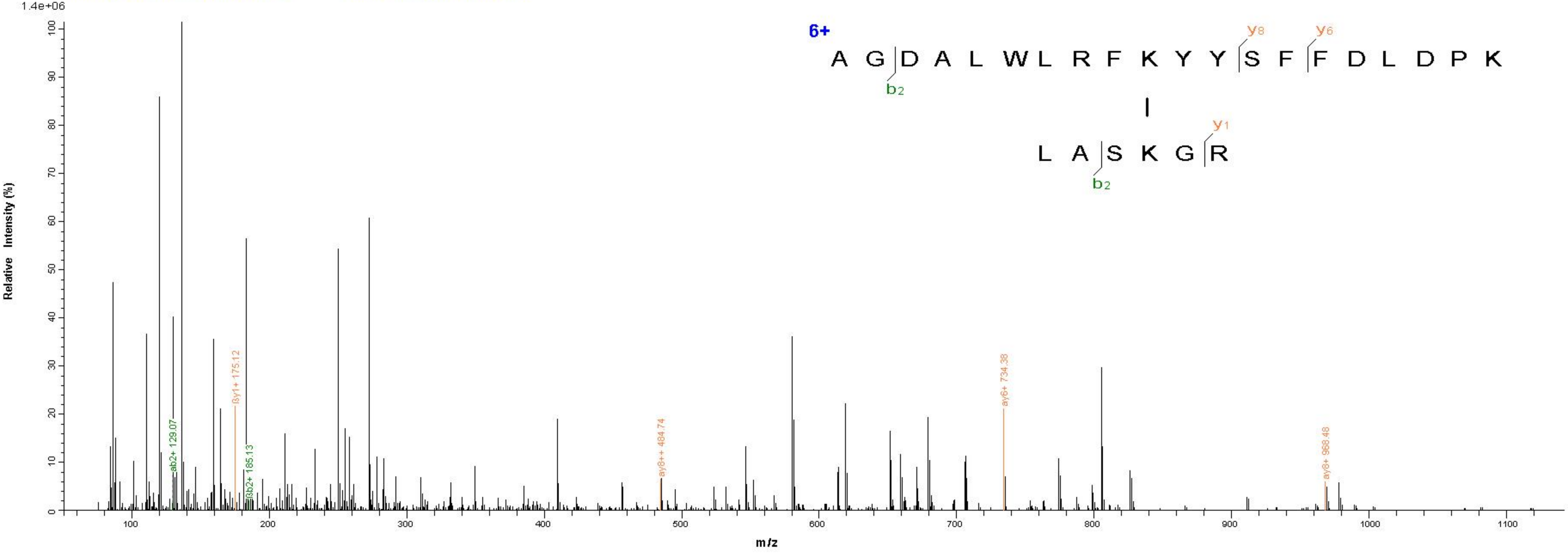

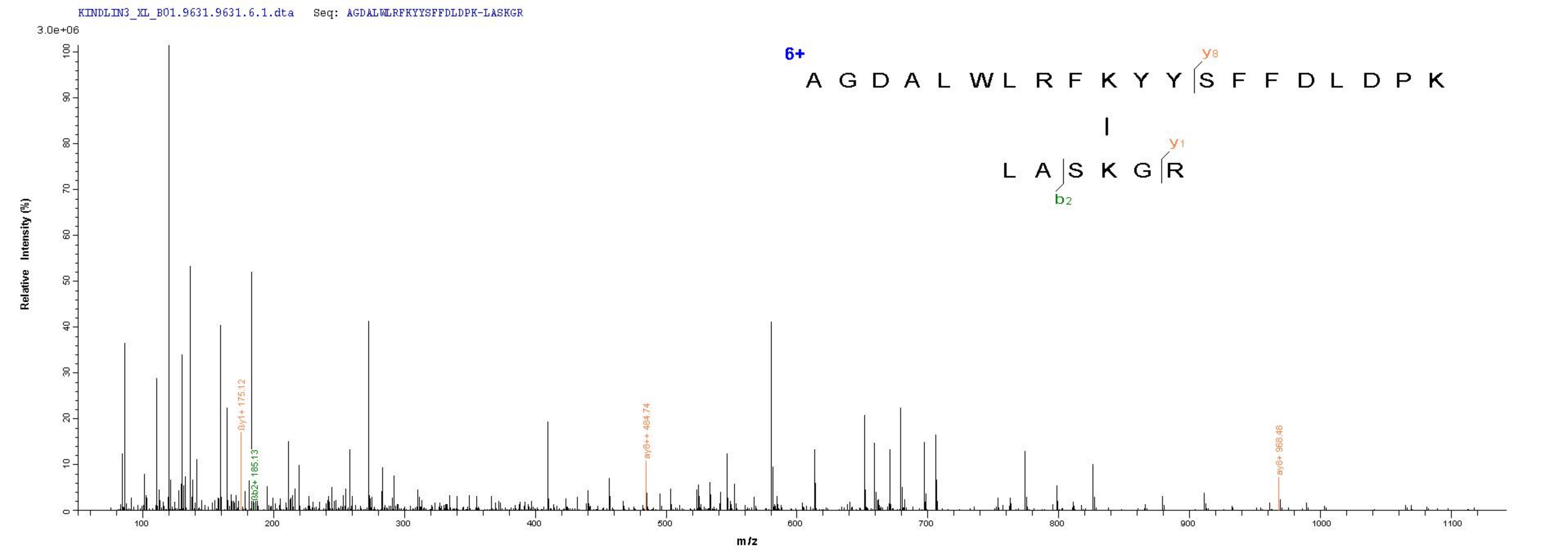

KINDLIN3_XL_B01.9631.9631.6.1.dta Seq: AGDALWLRFKYYSFFDLDPK-LASKGR
3.0e+06
Relative Intensity (%)
m/z
6+
A G D A L W L R F K Y Y S F F D L D P K
L A S K G R
y8
y1
b2
ßy1+ 175.12
ßb2+ 185.13
αy8++ 484.74
αy8+ 968.48

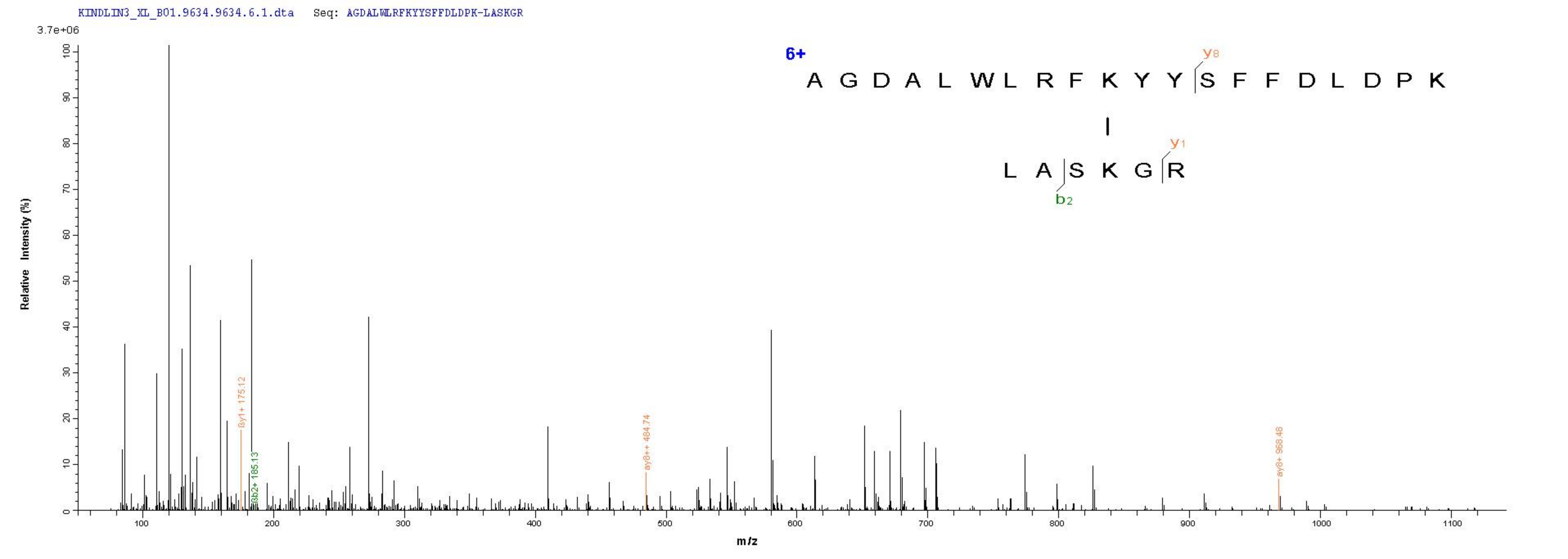

KINDLIN3_XL_B01.9634.9634.6.1.dta Seq: AGDALWLRFKYYSFFDLDPK-LASKGR
3.7e+06
Relative Intensity (%)
m/z
6+
A G D A L W L R F K Y Y S F F D L D P K
L A S K G R
y8
y1
b2
By1+ 175.12
Bb2+ 185.13
ay8++ 484.74
ay8+ 968.48

KINDLIN3_XL_B02.9663.9663.6.4.dta   Seq: AGDALWLRFKYYSFFDLDPK-LASKGR

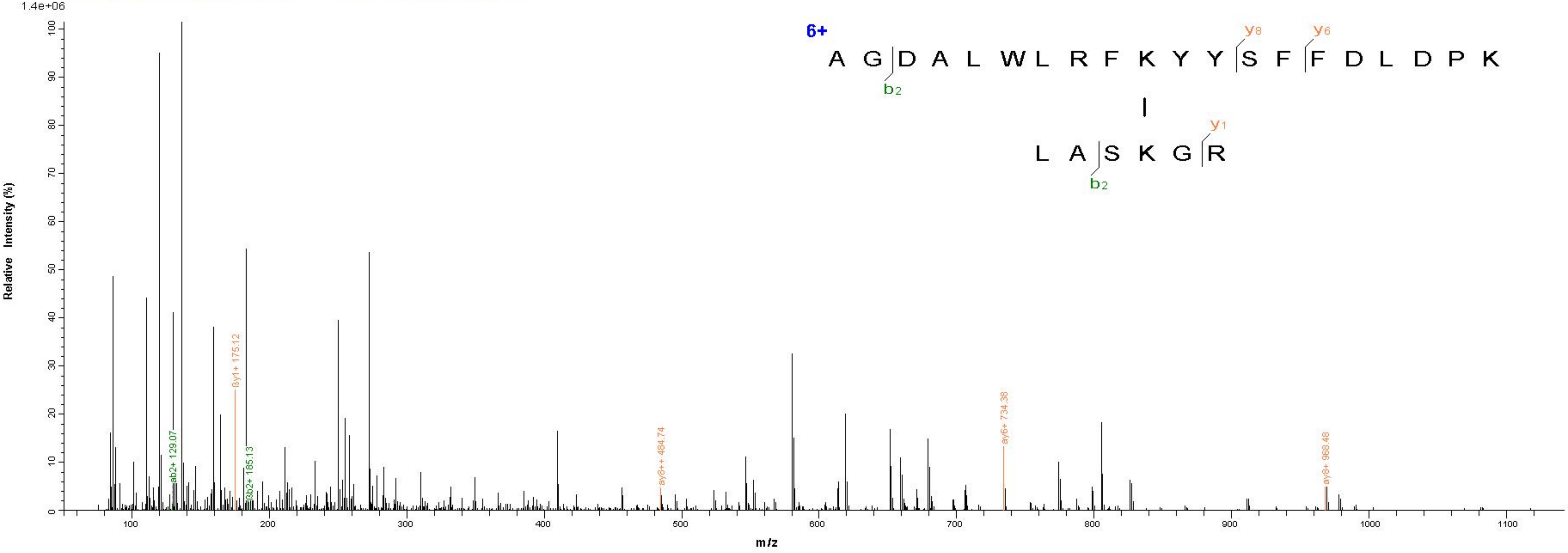

KINDLIN3_XL_B02.9692.9692.6.0.dta Seq: AGDALWLRFKYYSFFDLDPK-LASKGR

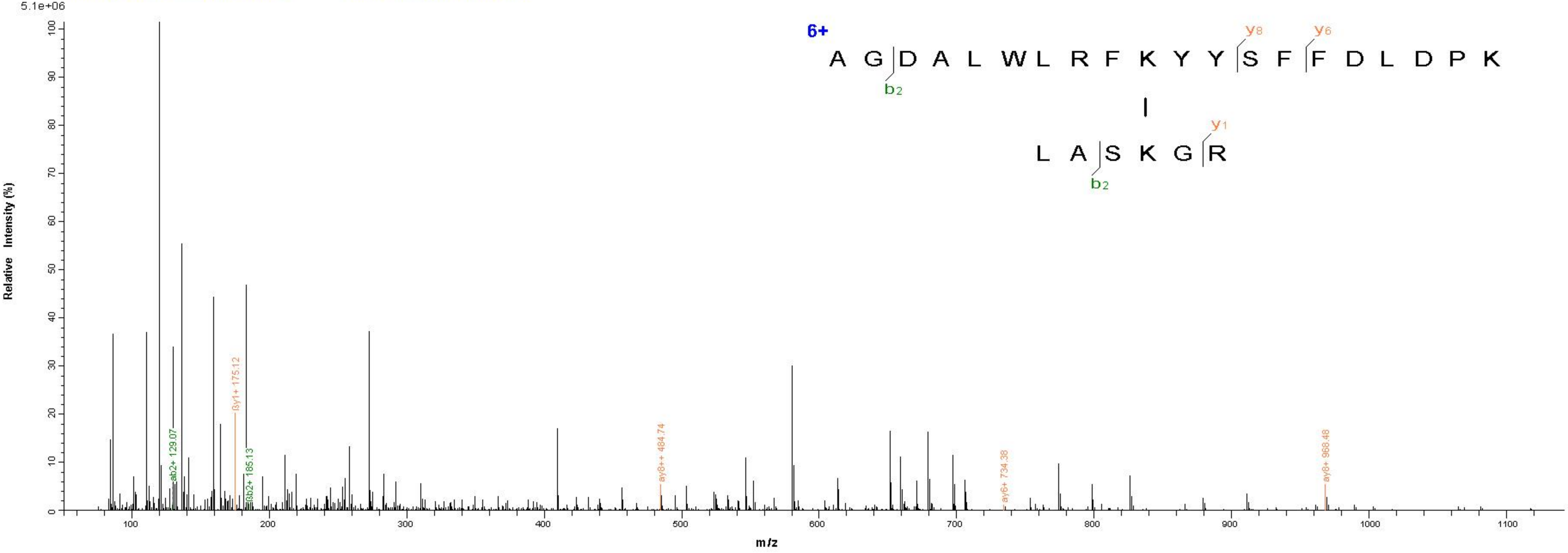

KINDLIN3_XL_B03.9547.9547.5.2.dta Seq: AGDALWLRFKYYSFFDLDPK-LASKGR

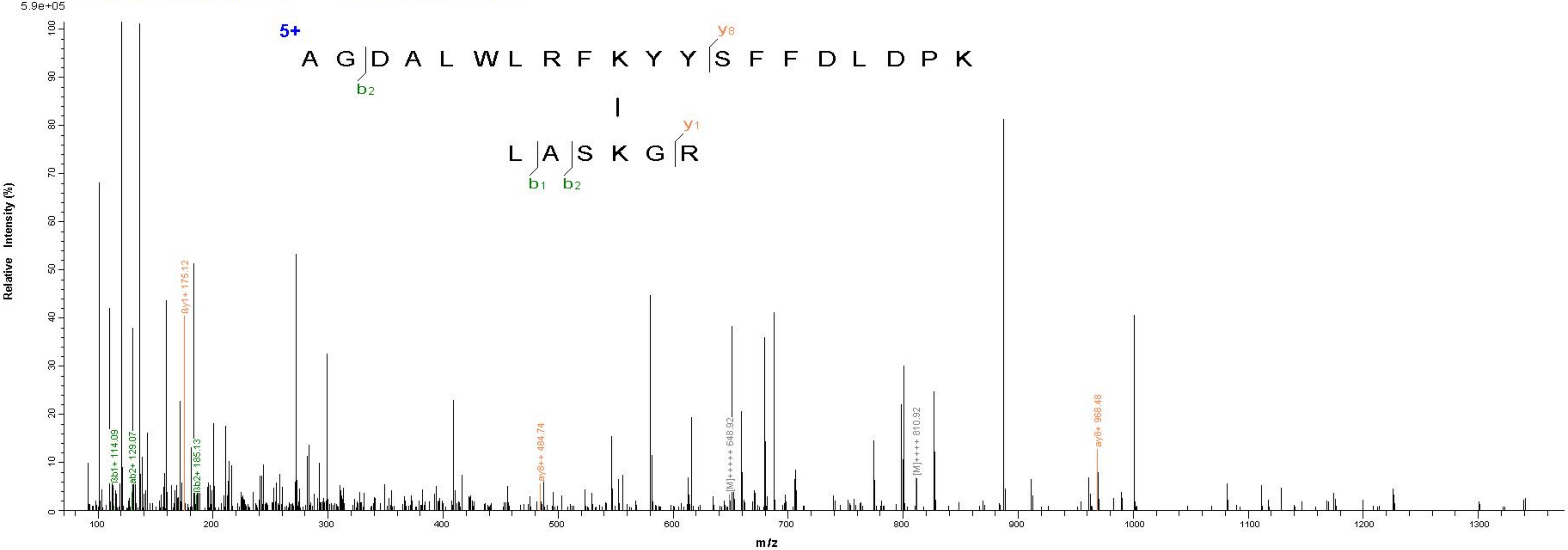

KINDLIN3_XL_B03.9552.9552.5.2.dta Seq: AGDALWLRFKYYSFFDLDPK-LASKGR

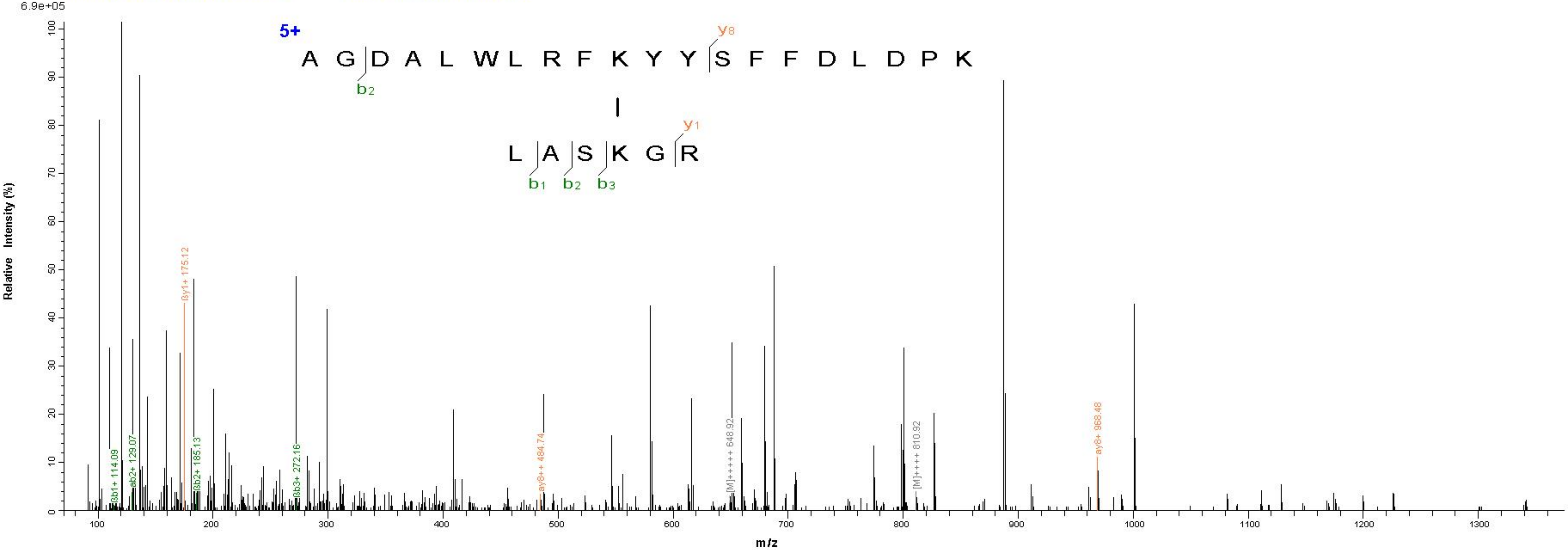

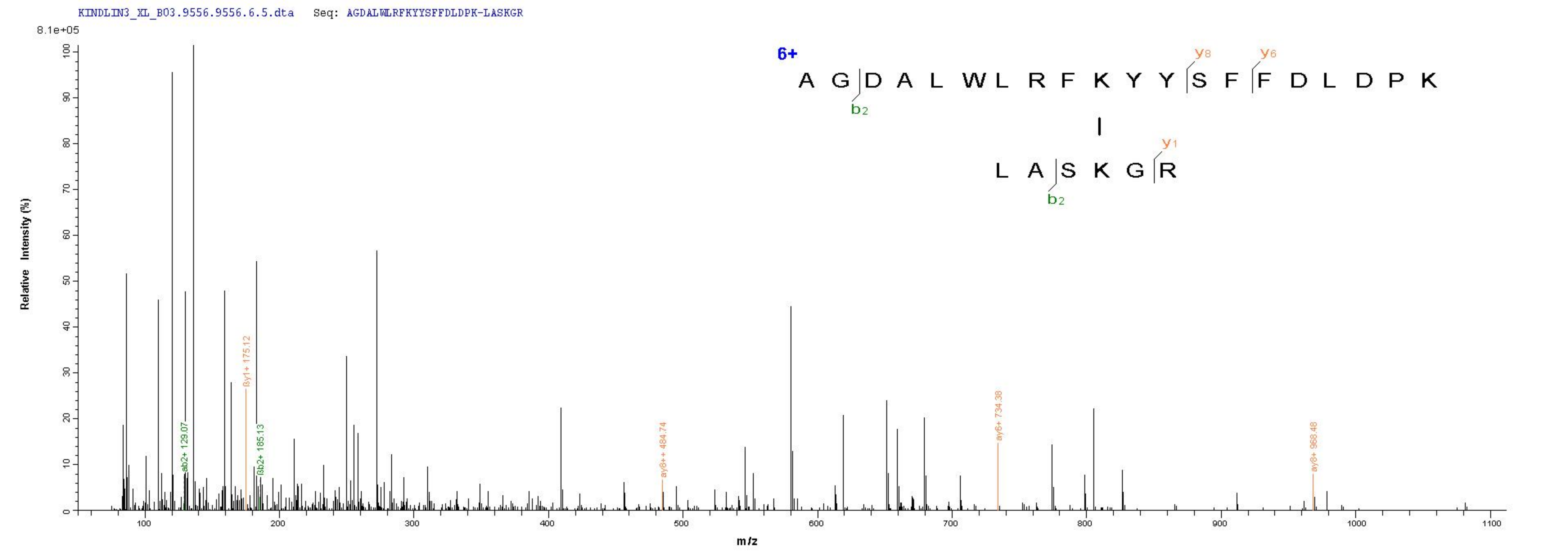
KINDLIN3_XL_B03.9556.9556.6.5.dta Seq: AGDALWLRFKYYSFFDLDPK-LASKGR
8.1e+05
6+
A G D A L W L R F K Y Y S F F D L D P K
L A S K G R
y8
y6
b2
y1
b2
Relative Intensity (%)
m/z
αb2+ 129.07
βy1+ 175.12
βb2+ 185.13
αy8++ 484.74
αy6+ 734.38
αy8+ 968.48

KINDLIN3_XL_B03.9557.9557.6.5.dta Seq: AGDALWLRFKYYSFFDLDPK-LASKGR

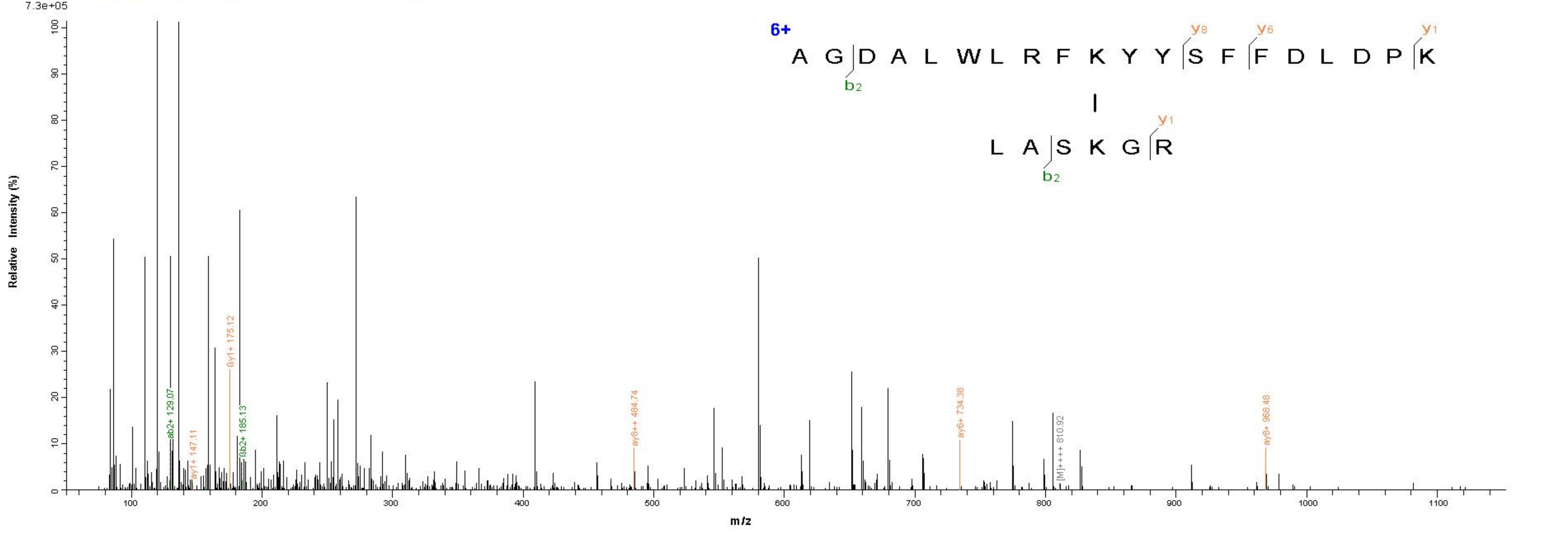

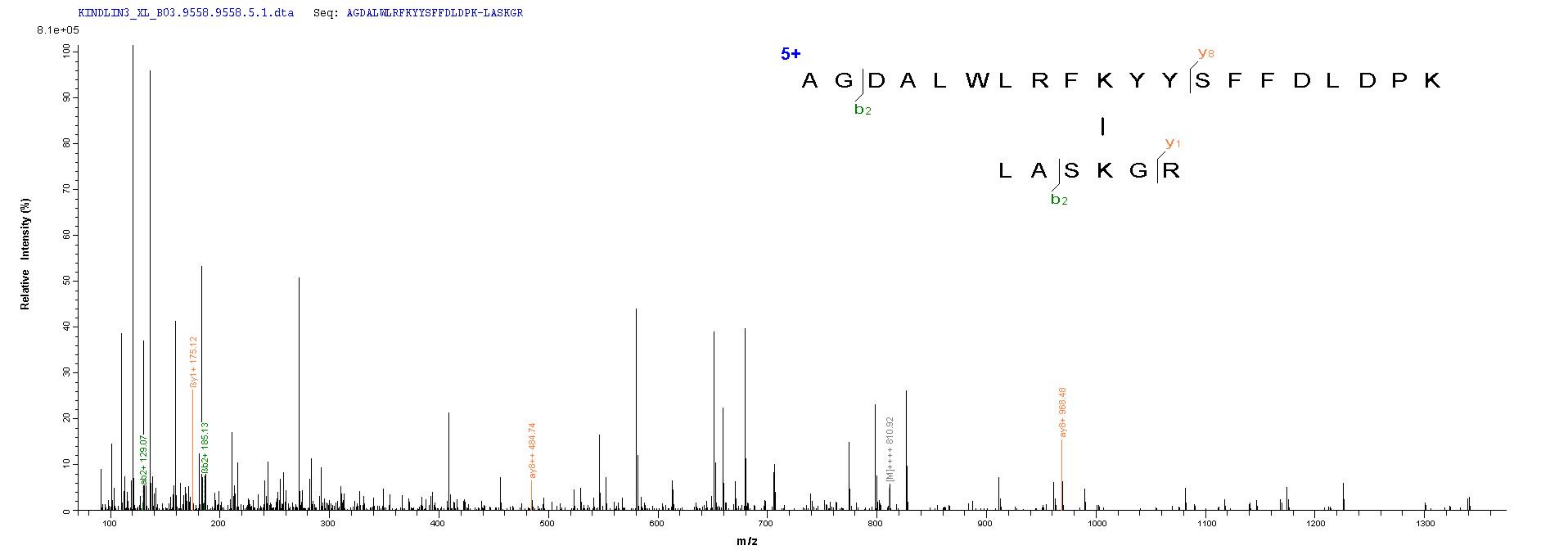

KINDLIN3_XL_B03.9558.9558.5.1.dta Seq: AGDALWLRFKYYSFFDLDPK-LASKGR
8.1e+05
Relative Intensity (%)
m/z
5+
A G D A L W L R F K Y Y S F F D L D P K
L A S K G R
b2
y8
y1
b2
ßy1+ 175.12
ab2+ 129.07
ßb2+ 185.13
ay8++ 484.74
[M]++++ 810.92
ay8+ 968.48

KINDLIN3_XL_B03.9559.9559.5.2.dta Seq: AGDALWLRFKYYSFFDLDPK-LASKGR

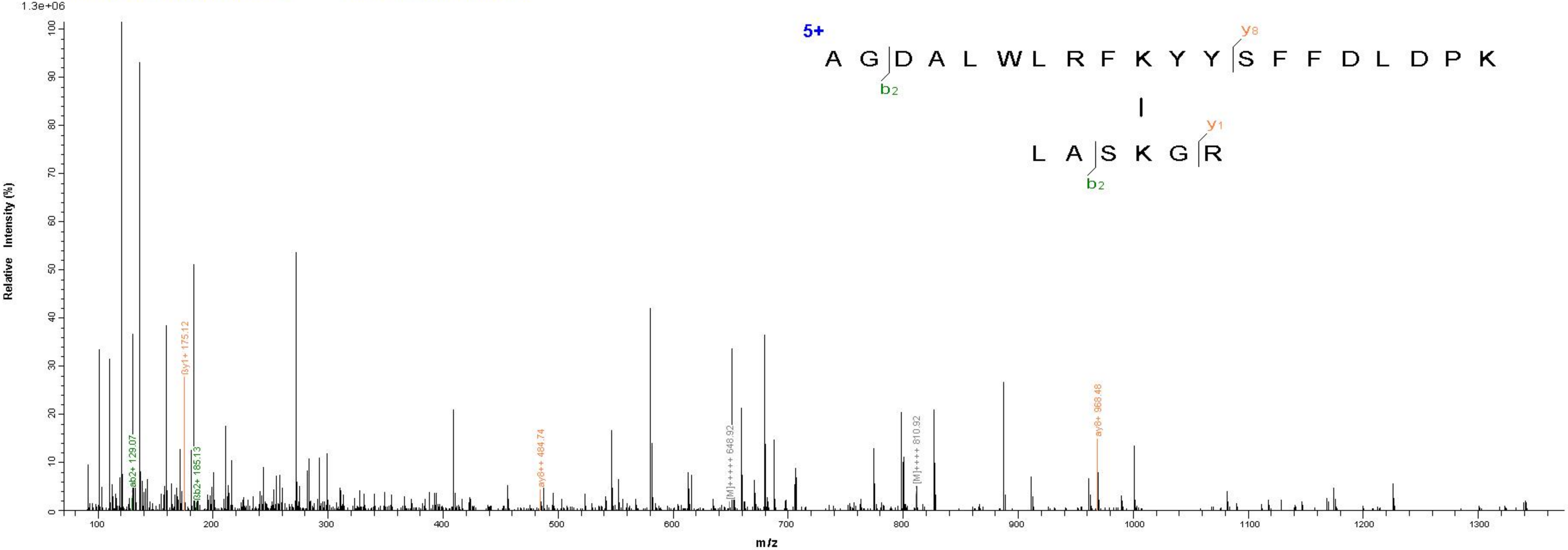

KINDLIN3_XL_B03.9573.9573.6.4.dta   Seq: AGDALWLRFKYYSFFDLDPK-LASKGR

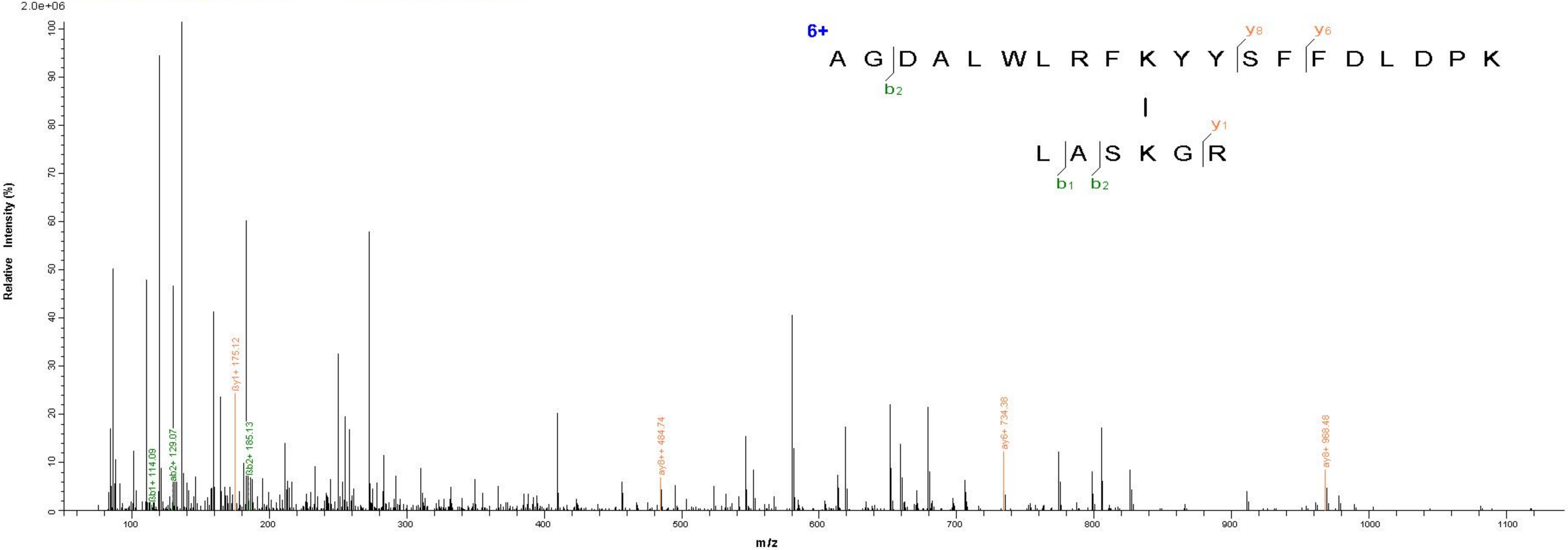

KINDLIN3_XL_B03.9577.9577.6.4.dta Seq: AGDALWLRFKYYSFFDLDPK-LASKGR

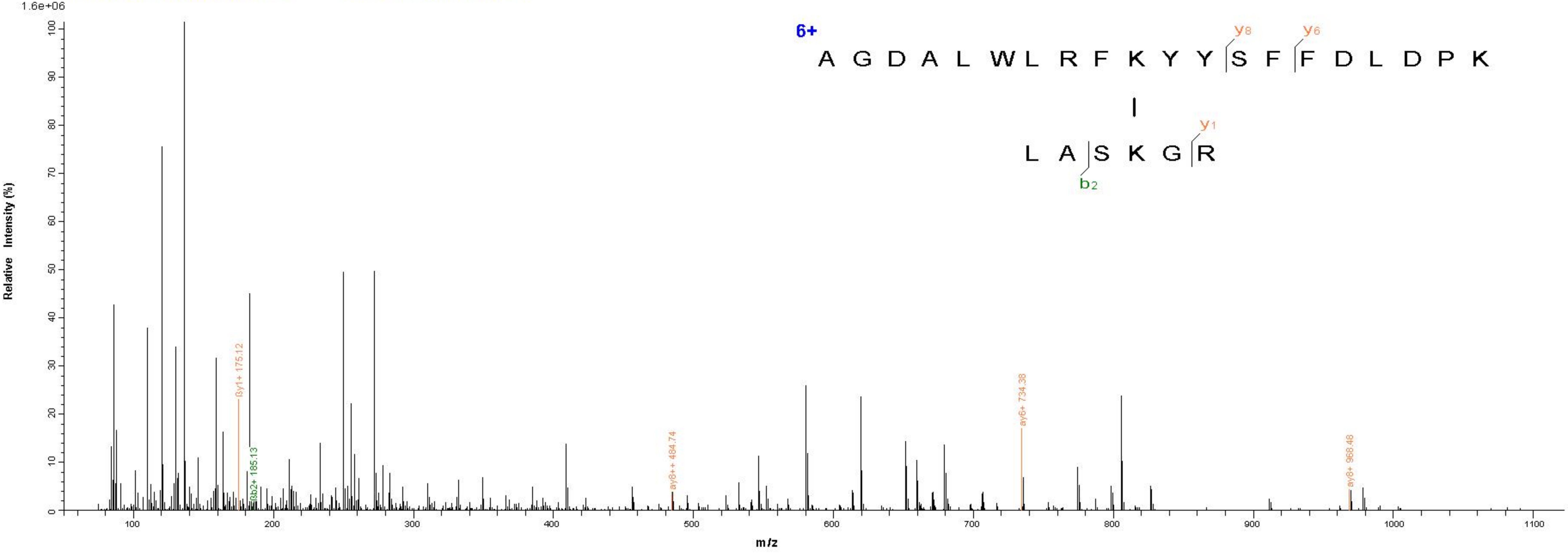

KINDLIN3_XL_B03.9622.9622.6.1.dta   Seq: AGDALWLRFKYYSFFDLDPK-LASKGR

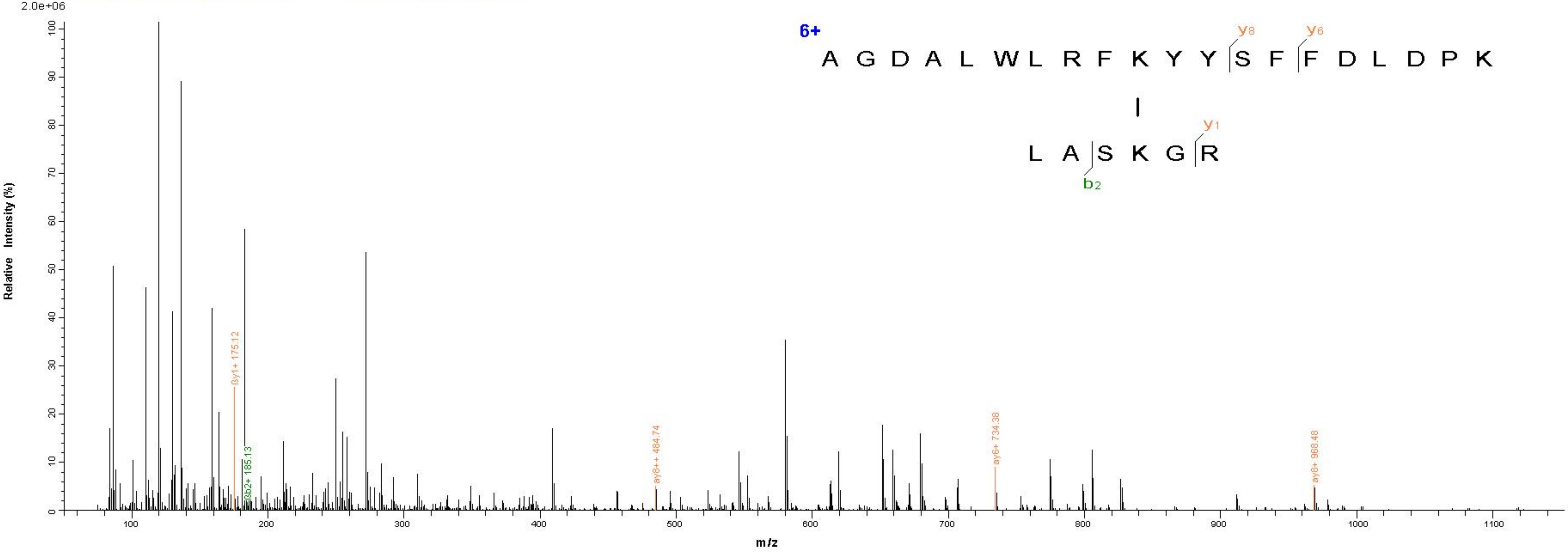

KINDLIN3_XL_B03.9634.9634.6.1.dta Seq: AGDALWLRFKYYSFFDLDPK-LASKGR

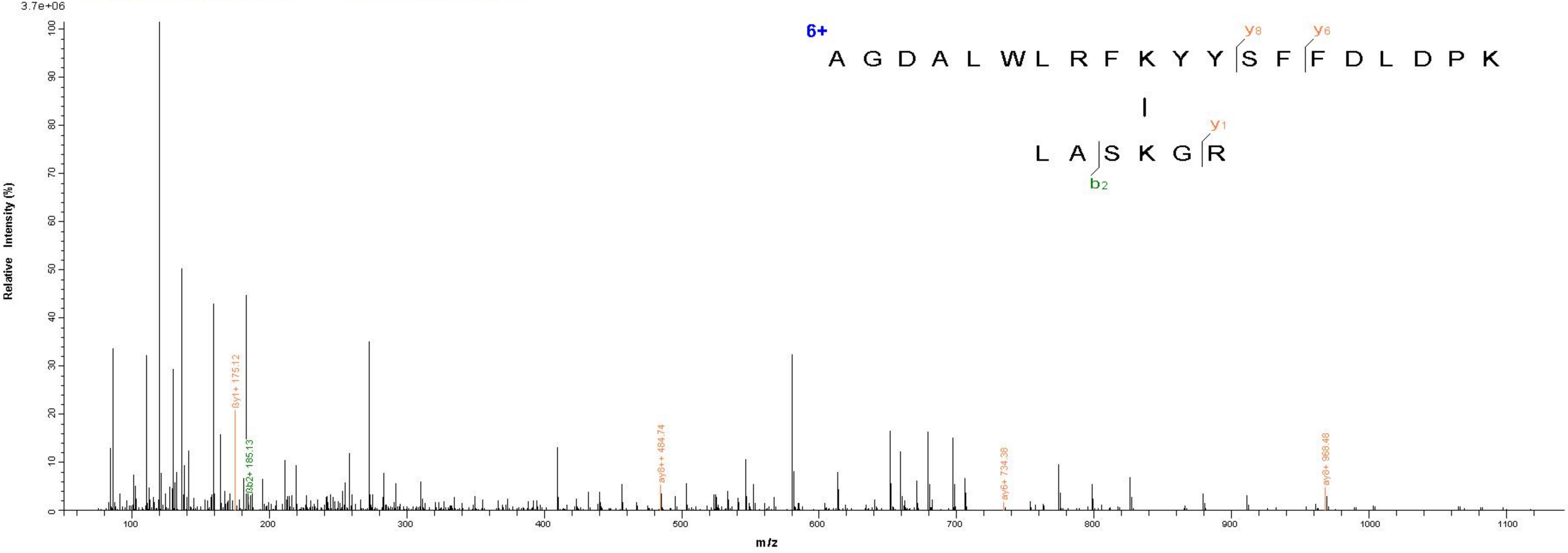

KINDLIN3_XL_B03.9635.9635.6.1.dta Seq: AGDALWLRFKYYSFFDLDPK-LASKGR

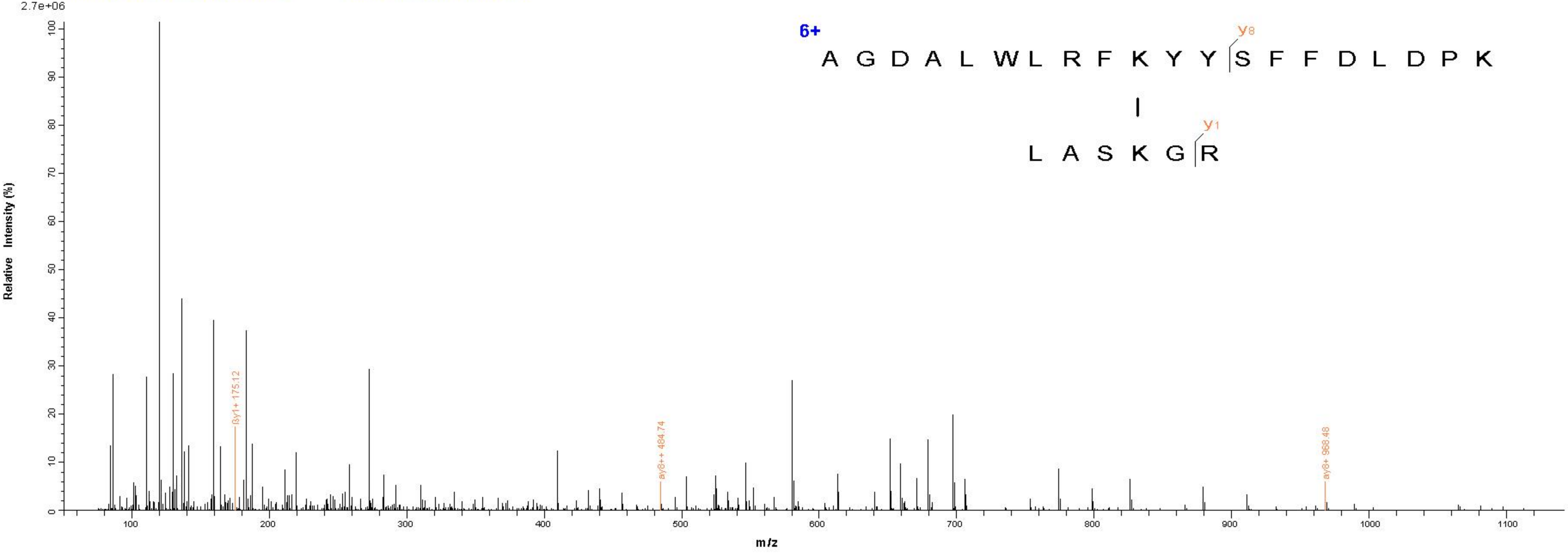

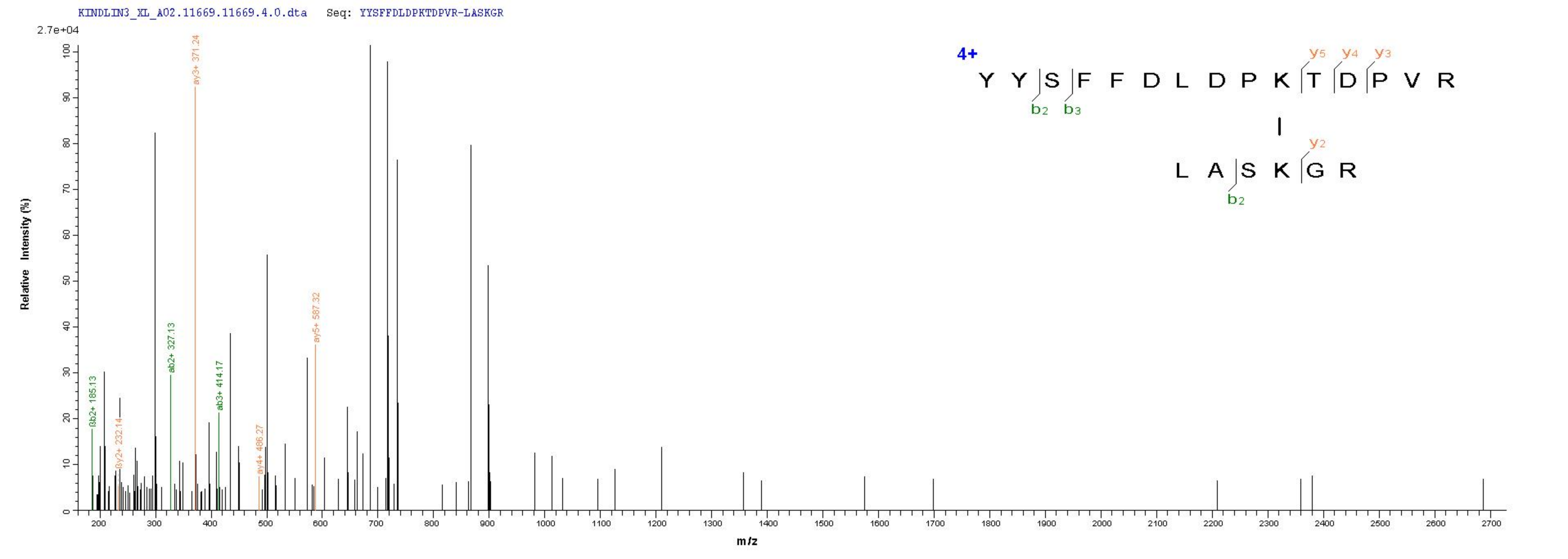
KINDLIN3_XL_A02.11669.11669.4.0.dta Seq: YYSFFDLDPKTDPVR-LASKGR
2.7e+04
Relative Intensity (%)
m/z
ßb2+ 185.13
ßy2+ 232.14
ab2+ 327.13
ay3+ 371.24
ab3+ 414.17
ay4+ 486.27
ay5+ 587.32
4+
y5 y4 y3
Y Y S F F D L D P K T D P V R
b2 b3
y2
L A S K G R
b2

KINDLIN3_XL_A02.11733.11733.4.0.dta Seq: YYSFFDLDPKTDPVR-LASKGR

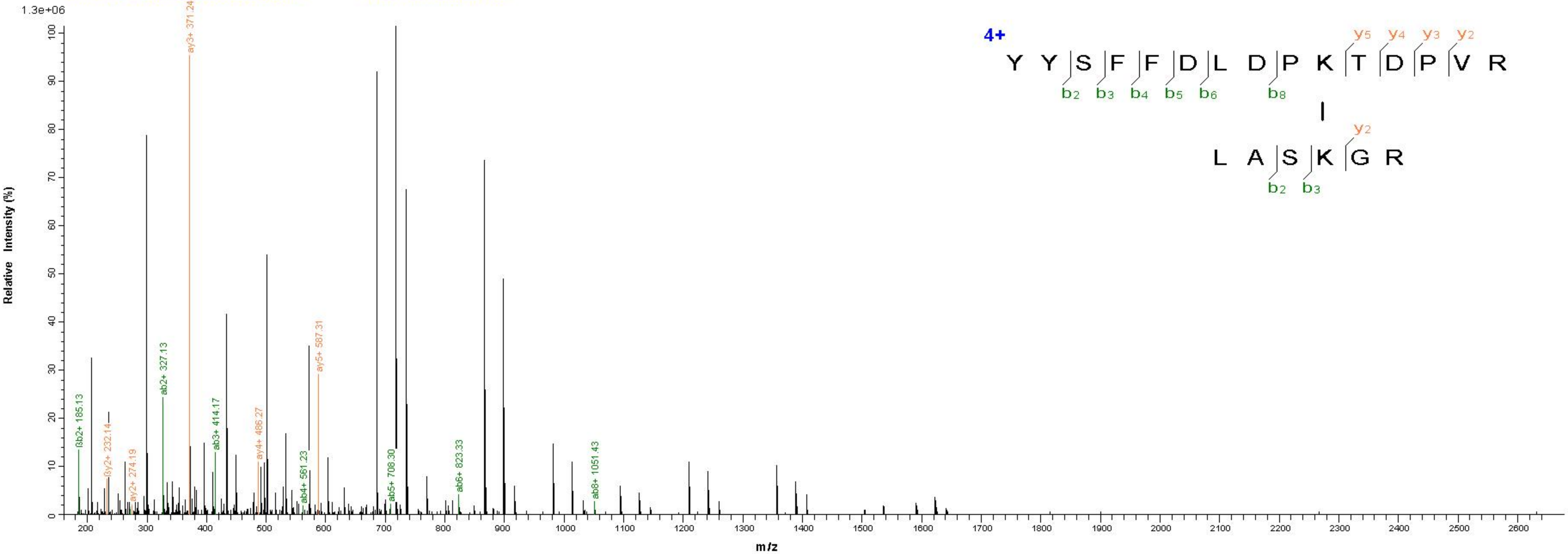

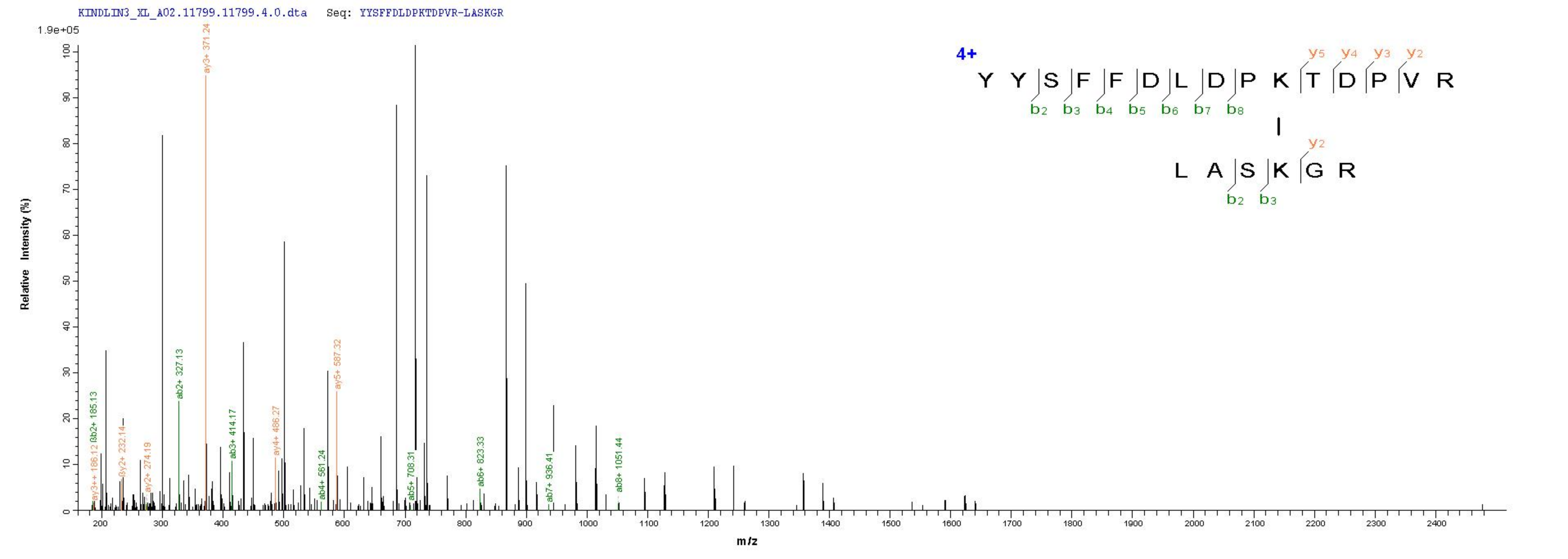

KINDLIN3_XL_A02.11799.11799.4.0.dta Seq: YYSFFDLDPKTDPVR-LASKGR
1.9e+05
Relative Intensity (%)
m/z
4+
Y Y S F F D L D P K T D P V R
y5 y4 y3 y2
b2 b3 b4 b5 b6 b7 b8
L A S K G R
y2
b2 b3
ay3+ 371.24
ay3++ 186.12
bb2+ 185.13
by2+ 232.14
ay2+ 274.19
ab2+ 327.13
ab3+ 414.17
ay4+ 486.27
ab4+ 561.24
ay5+ 587.32
ab5+ 708.31
ab6+ 823.33
ab7+ 936.41
ab8+ 1051.44

KINDLIN3_XL_A02.11818.11818.4.0.dta   Seq: YYSFFDLDPKTDPVR-LASKGR

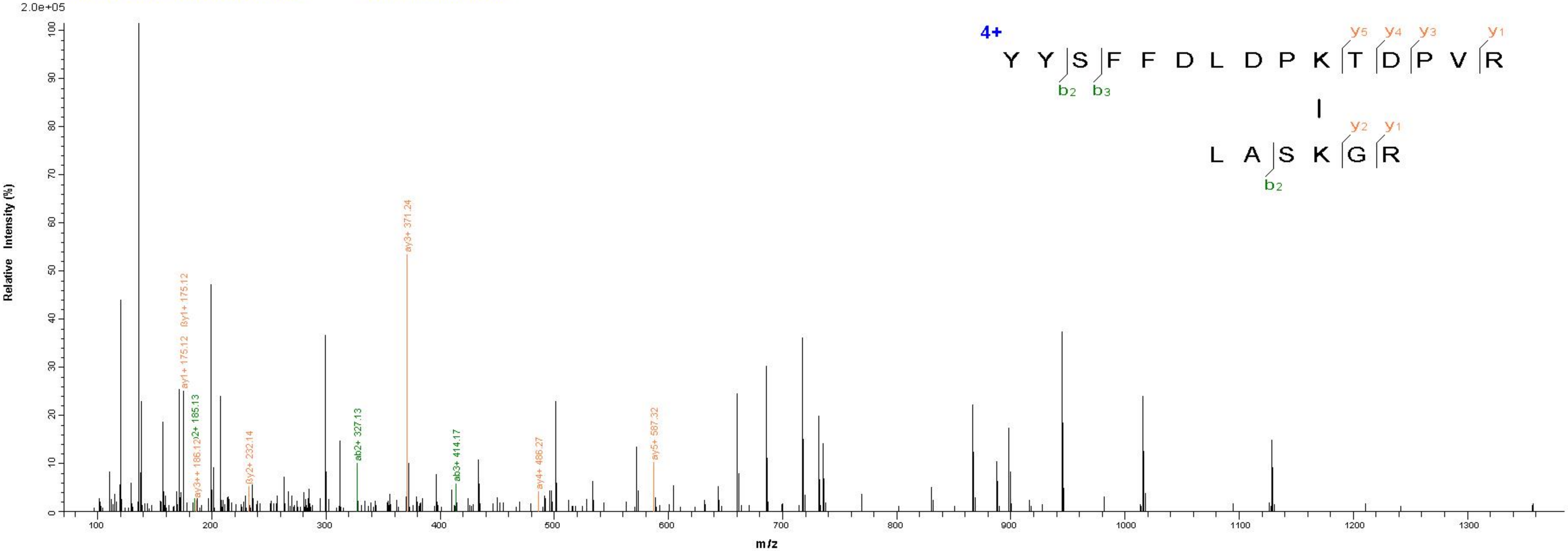

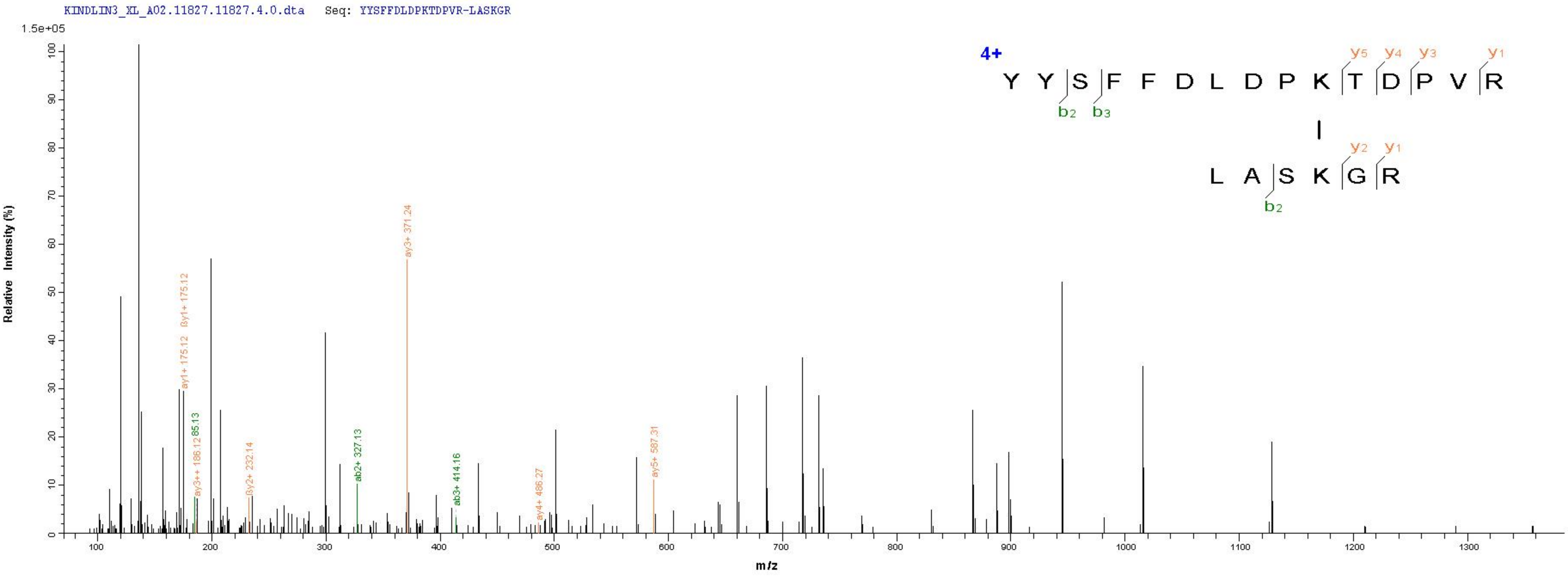
KINDLIN3_XL_A02.11827.11827.4.0.dta Seq: YYSFFDLDPKTDPVR-LASKGR
1.5e+05
Relative Intensity (%)
m/z
4+
Y Y S F F D L D P K T D P V R
y5 y4 y3 y1
b2 b3
L A S K G R
y2 y1
b2
ay1+ 175.12
By1+ 175.12
ay3++ 186.12
85.13
By2+ 232.14
ab2+ 327.13
ay3+ 371.24
ab3+ 414.16
ay4+ 486.27
ay5+ 587.31

KINDLIN3_XL_A03.11706.11706.4.0.dta Seq: YYSFFDLDPKTDPVR-LASKGR

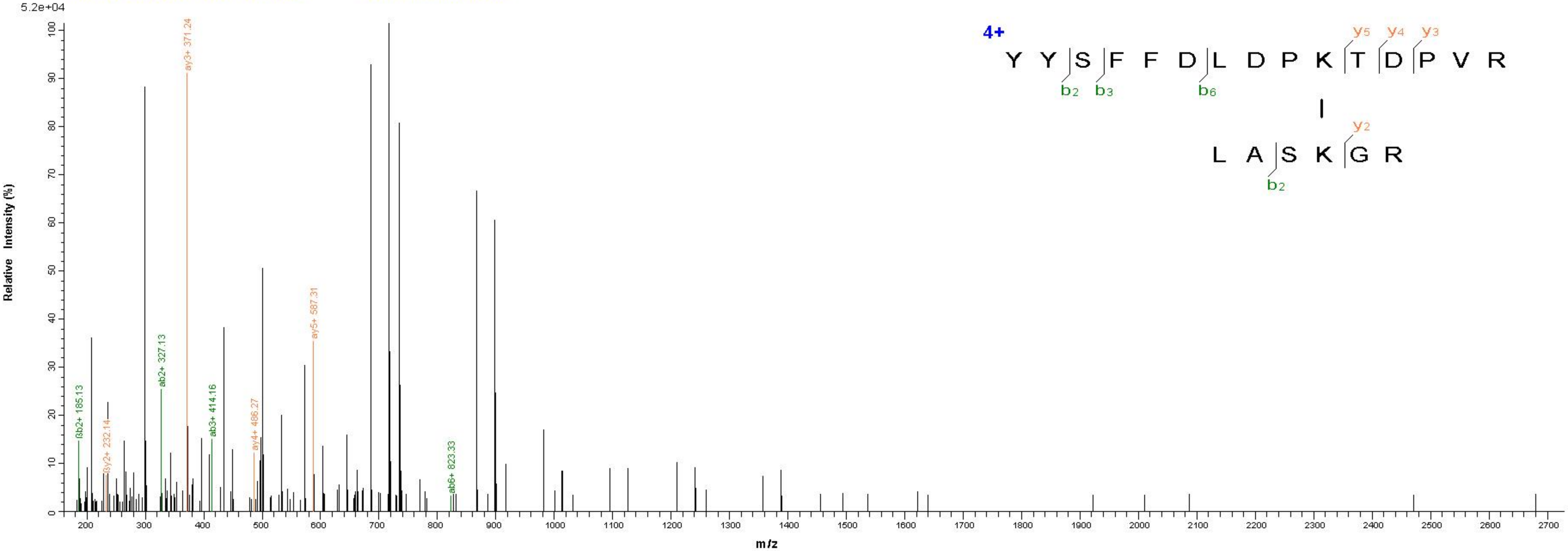

KINDLIN3_XL_A03.11748.11748.4.0.dta Seq: YYSFFDLDPKTDPVR-LASKGR

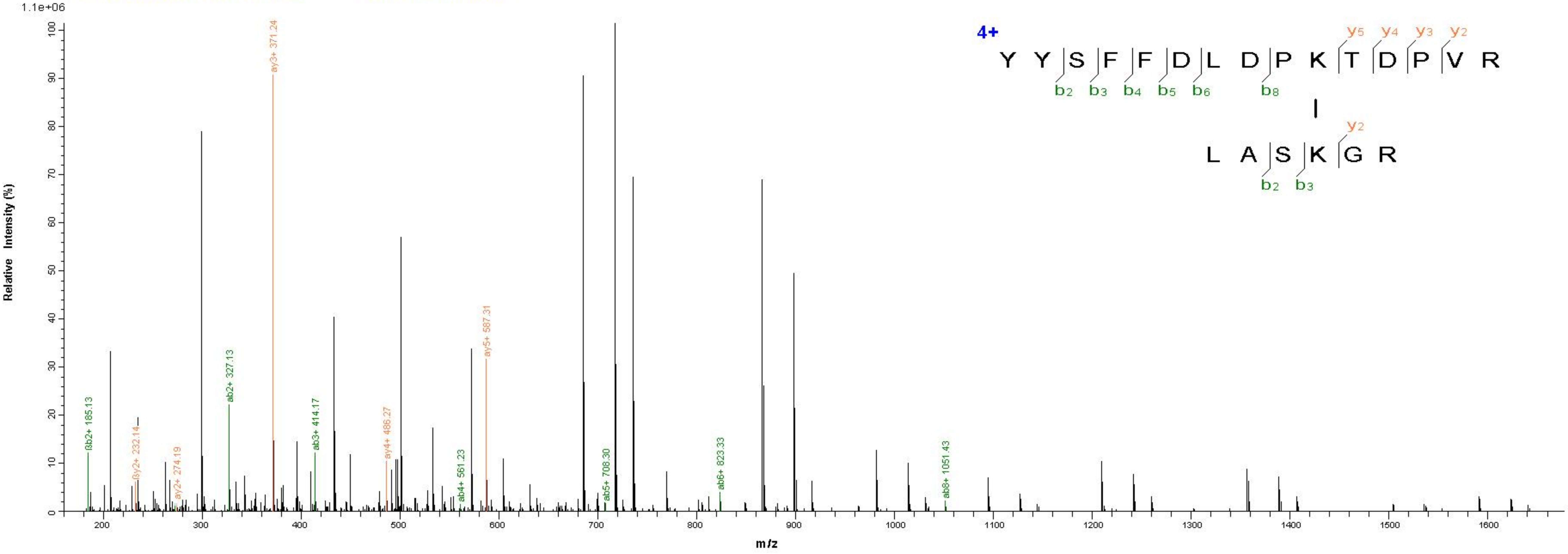

KINDLIN3_XL_A03.11754.11754.3.0.dta   Seq: YYSFFDLDPKTDPVR-LASKGR

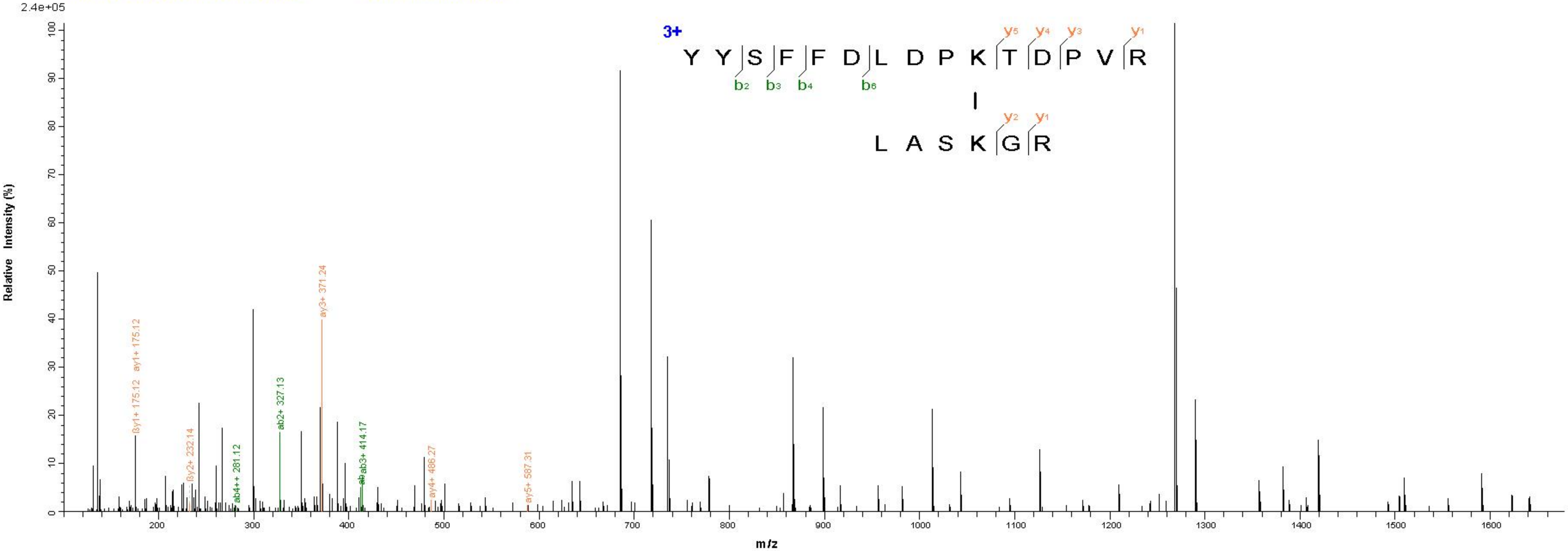

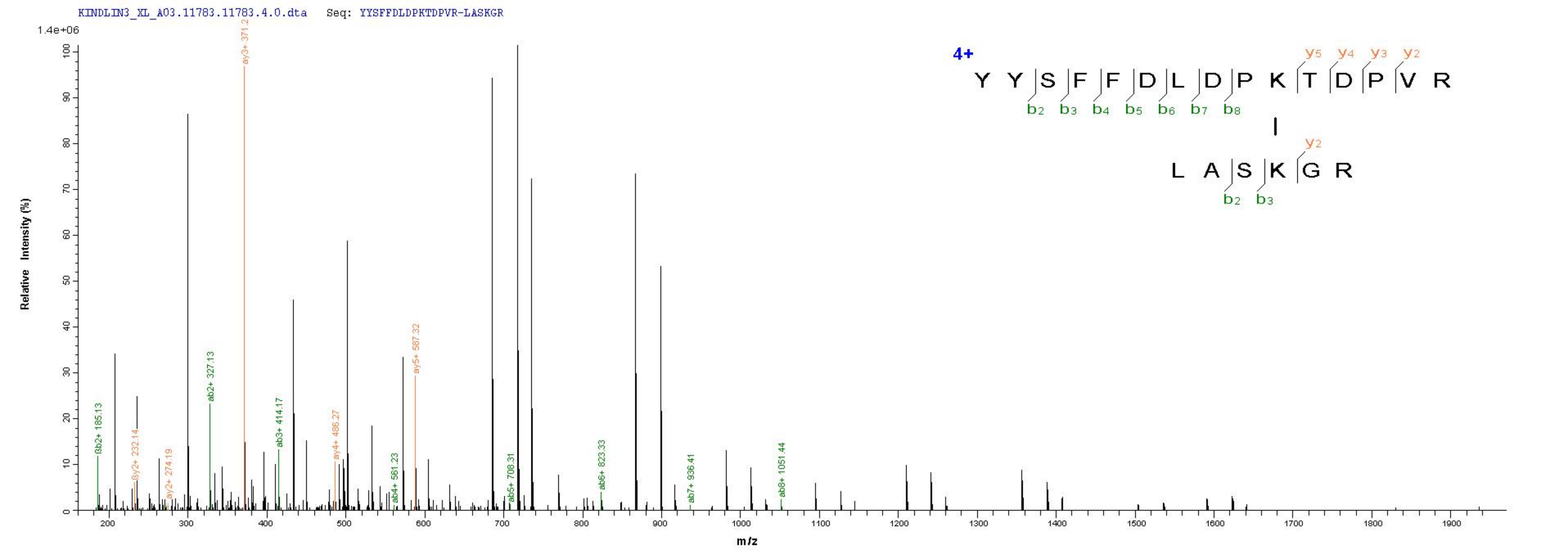
KINDLIN3_XL_A03.11783.11783.4.0.dta Seq: YYSFFDLDPKTDPVR-LASKGR
1.4e+06
Relative Intensity (%)
m/z
4+
Y Y S F F D L D P K T D P V R
L A S K G R
b2 b3 b4 b5 b6 b7 b8
y5 y4 y3 y2
y2
b2 b3
ay3+ 371.2
ay5+ 587.32
ab2+ 327.13
ab3+ 414.17
ay4+ 486.27
ßb2+ 185.13
ßy2+ 232.14
ay2+ 274.19
ab4+ 561.23
ab5+ 708.31
ab6+ 823.33
ab7+ 936.41
ab8+ 1051.44

KINDLIN3_XL_A03.11828.11828.4.0.dta Seq: YYSFFDLDPKTDPVR-LASKGR

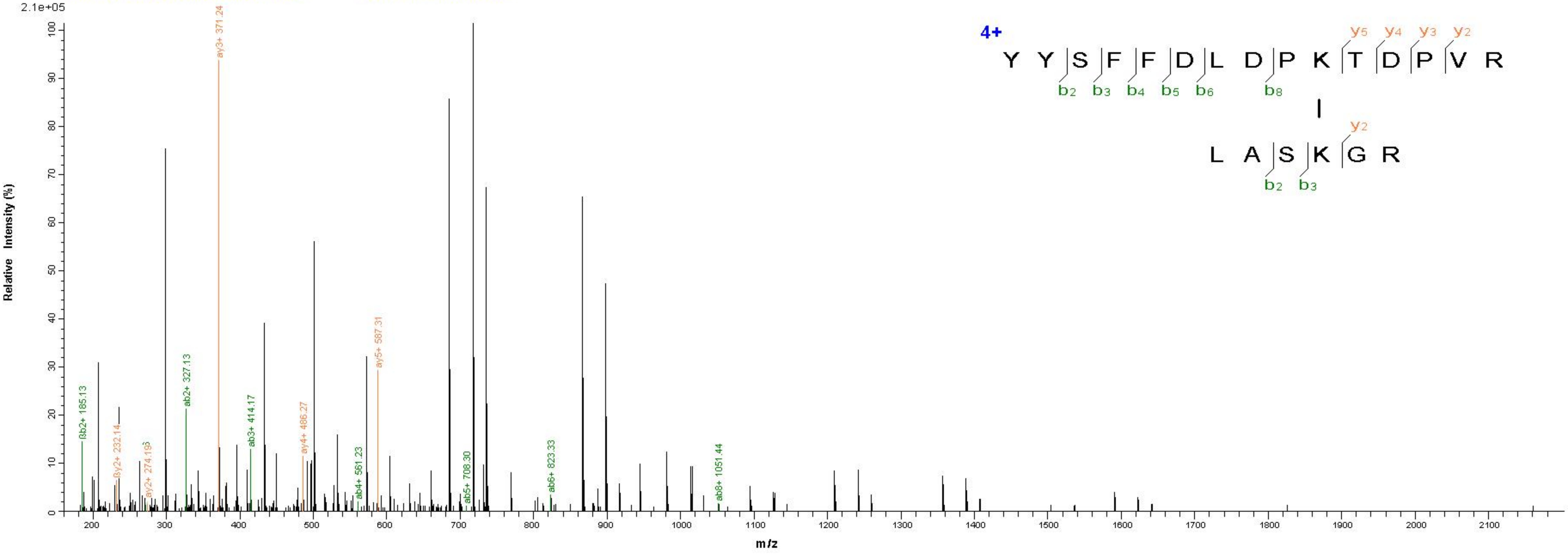

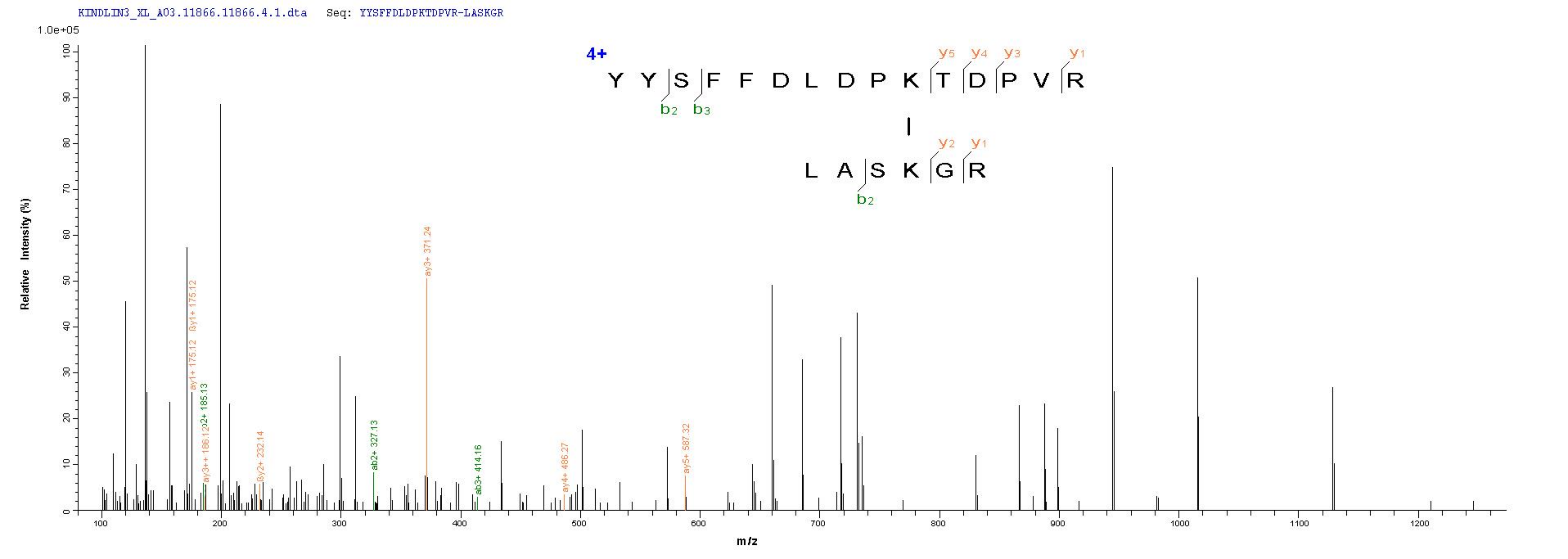
KINDLIN3_XL_A03.11866.11866.4.1.dta Seq: YYSFFDLDPKTDPVR-LASKGR
1.0e+05
4+
Y Y S F F D L D P K T D P V R
L A S K G R
Relative Intensity (%)
m/z
ay3+ 371.24
βy1+ 175.12
ay1+ 175.12
ab2+ 327.13
ab3+ 414.16
ay4+ 486.27
ay5+ 587.32
βy2+ 232.14
ay3++ 186.12
2+ 185.13

KINDLIN3_XL_B01.11700.11700.4.0.dta Seq: YYSFFDLDPKTDPVR-LASKGR

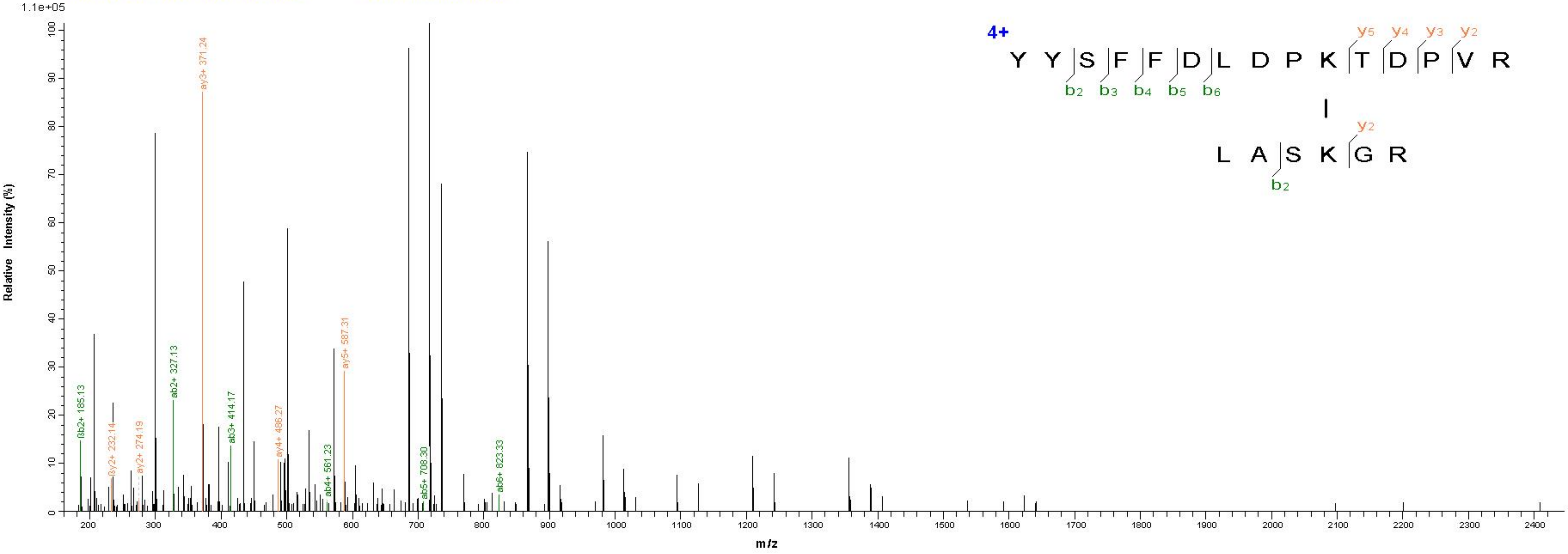

KINDLIN3_XL_B01.11739.11739.4.0.dta   Seq: YYSFFDLDPKTDPVR-LASKGR

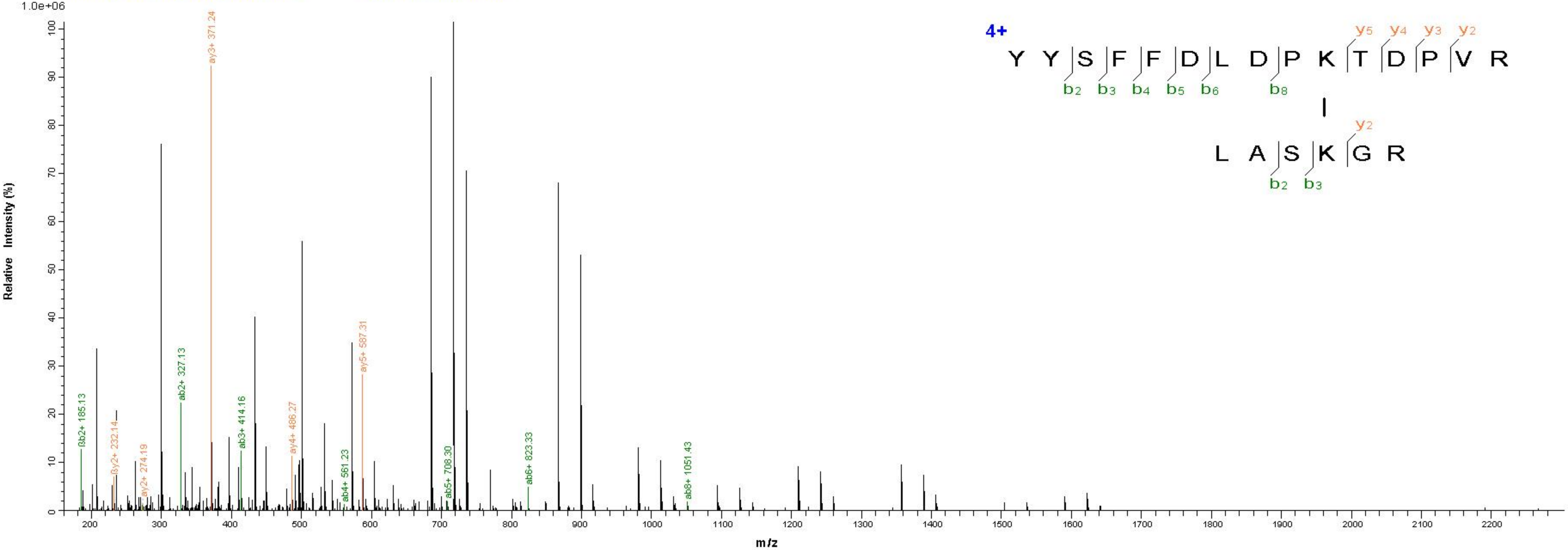

KINDLIN3_XL_B01.11774.11774.4.0.dta Seq: YYSFFDLDPKTDPVR-LASKGR

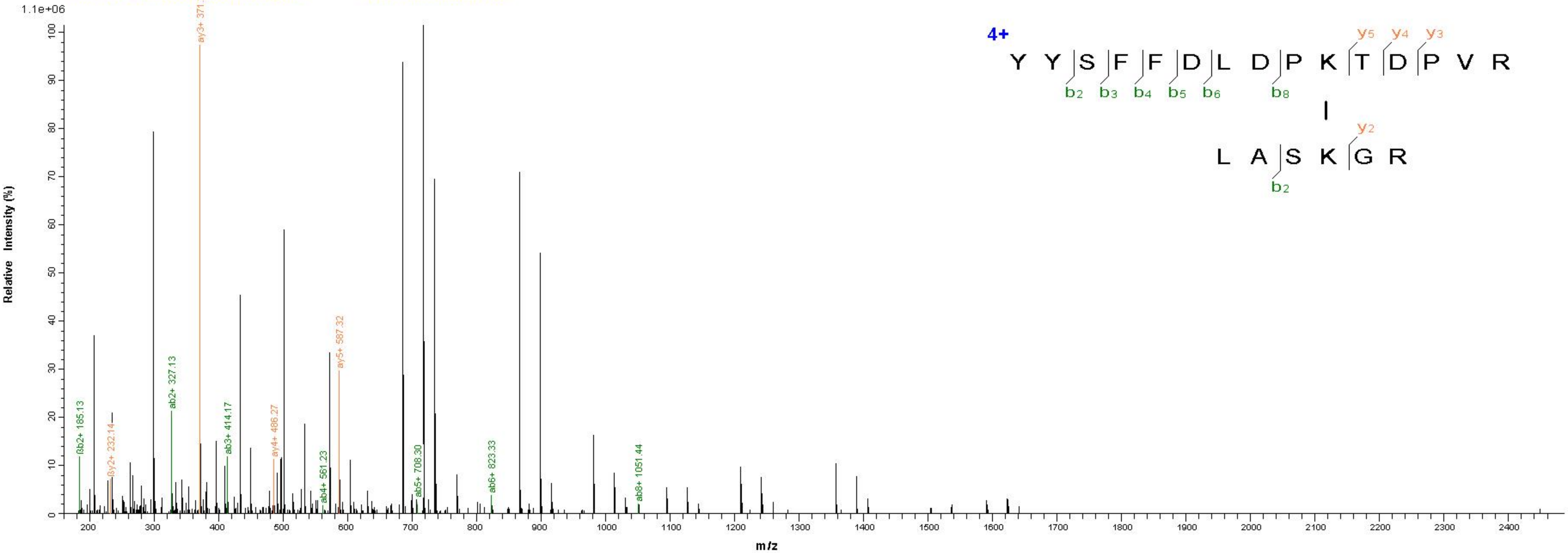

KINDLIN3_XL_B01.11818.11818.4.0.dta Seq: YYSFFDLDPKTDPVR-LASKGR

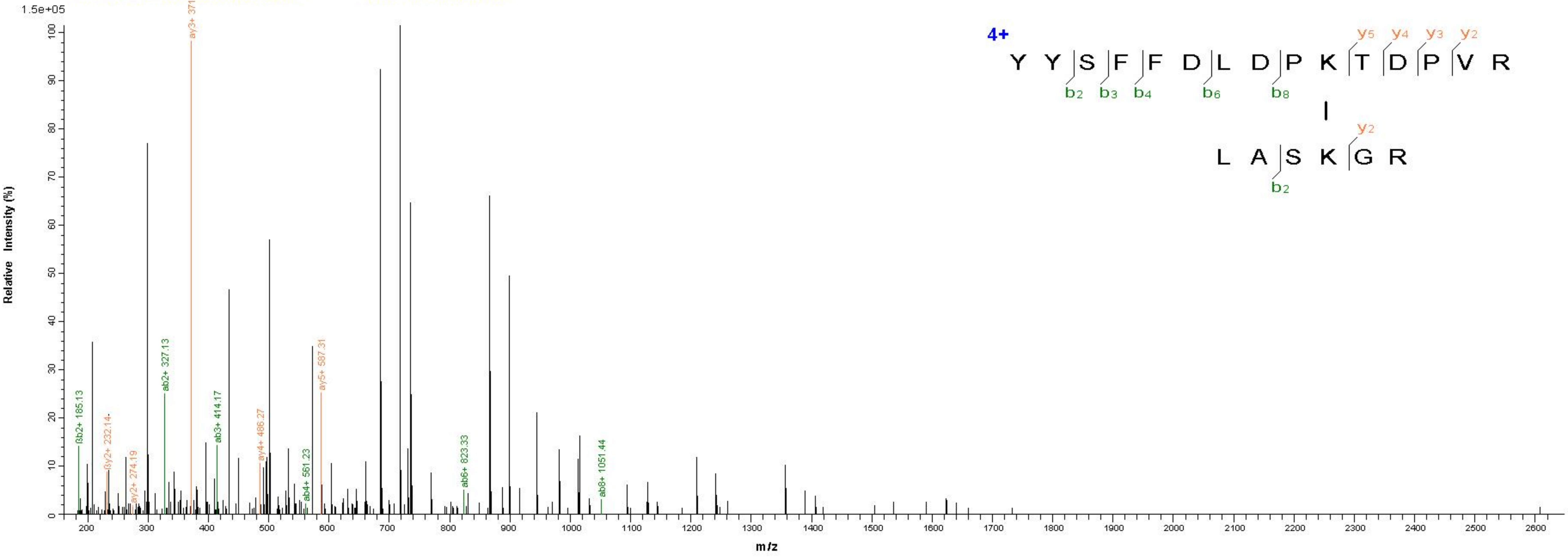

KINDLIN3_XL_B01.11847.11847.4.0.dta Seq: YYSFFDLDPKTDPVR-LASKGR

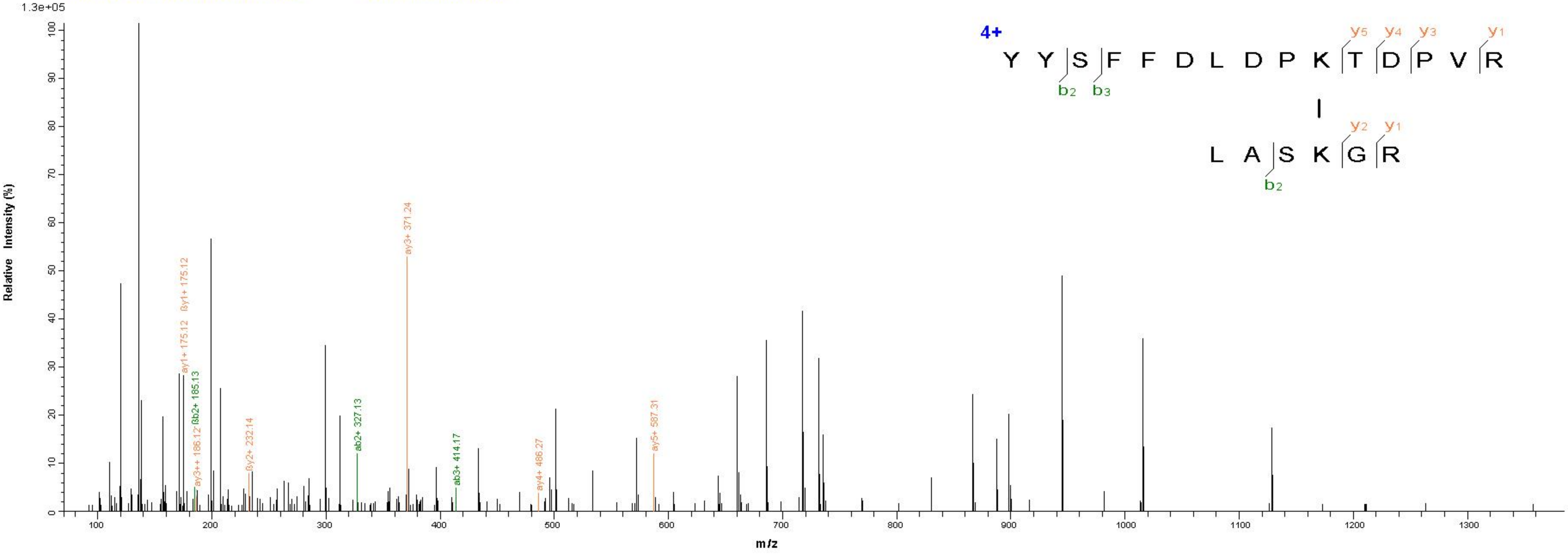

KINDLIN3_XL_B01.11895.11895.4.0.dta   Seq: YYSFFDLDPKTDPVR-LASKGR

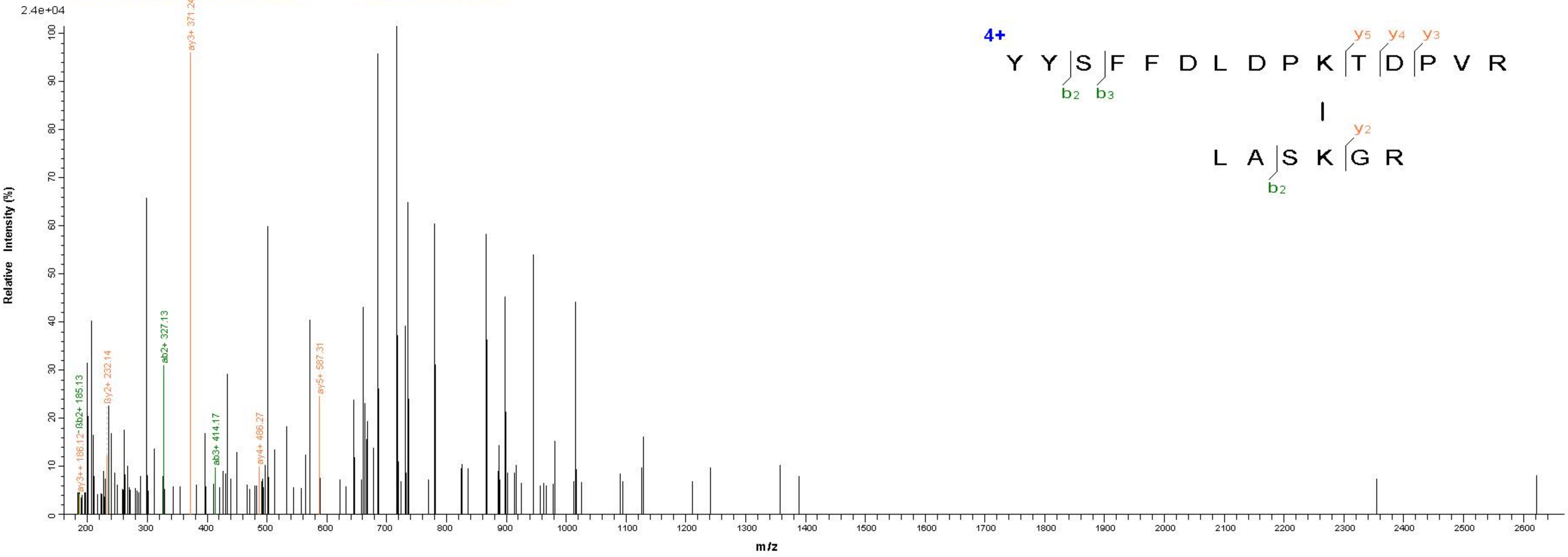

KINDLIN3_XL_B02.11748.11748.4.0.dta Seq: YYSFFDLDPKTDPVR-LASKGR

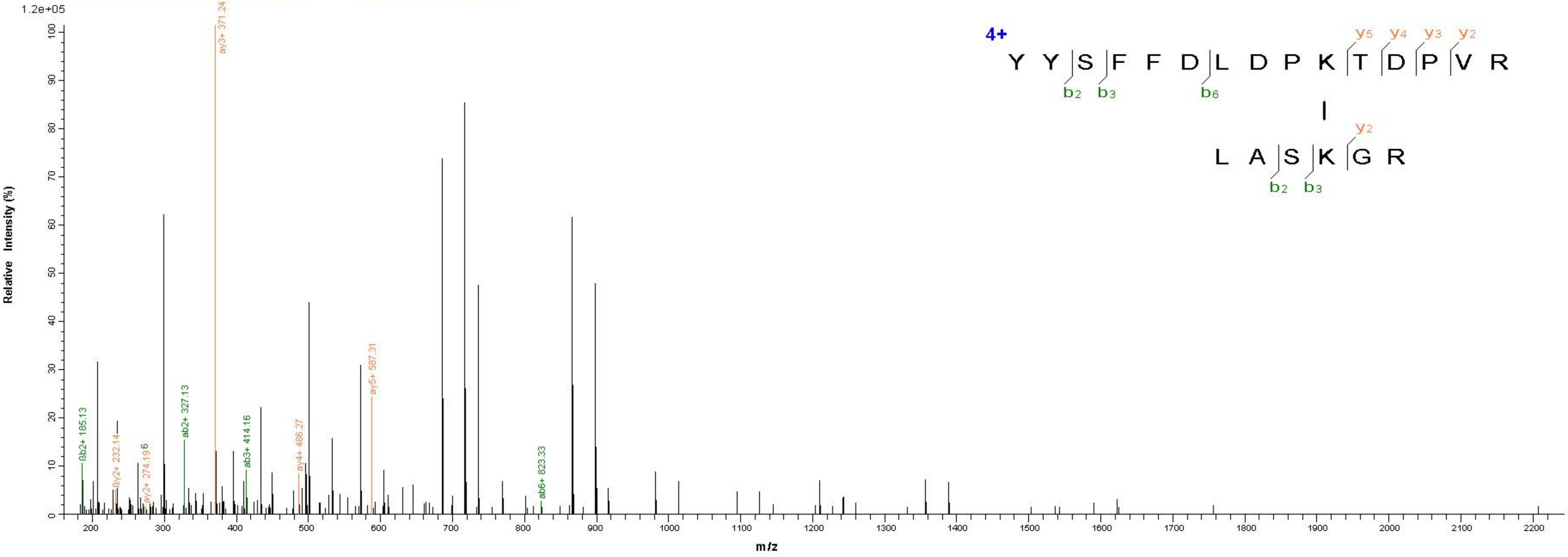

KINDLIN3_XL_B02.11784.11784.3.1.dta Seq: YYSFFDLDPKTDPVR-LASKGR

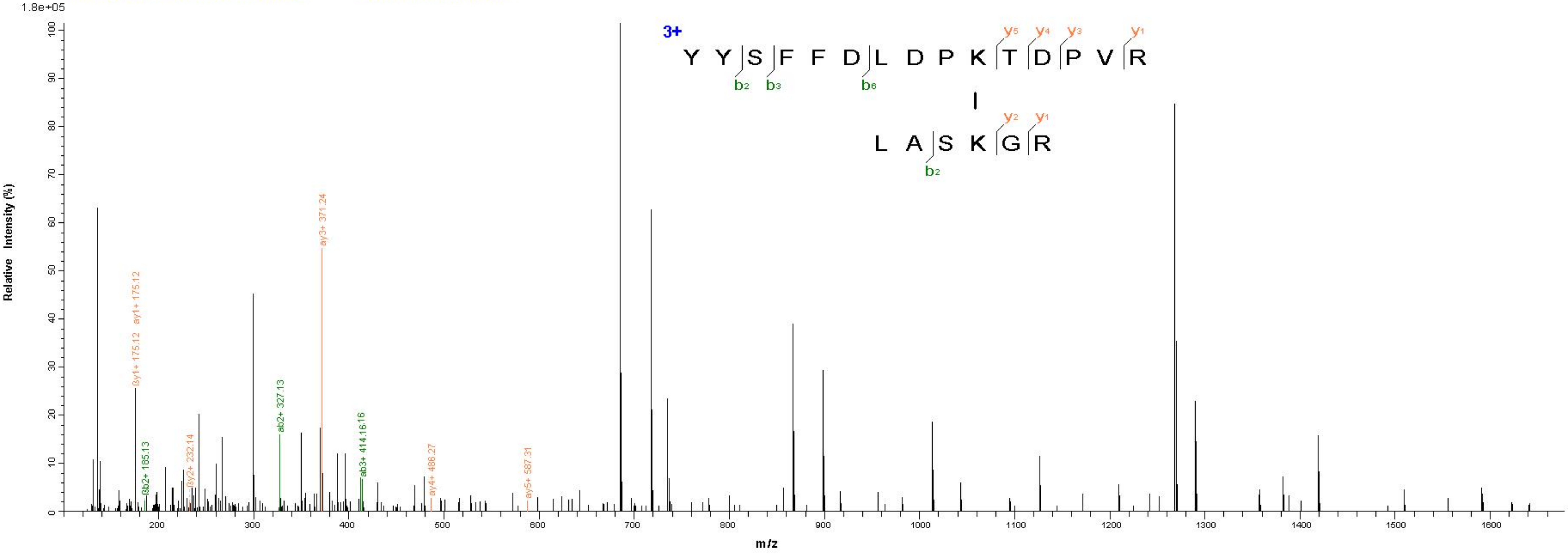

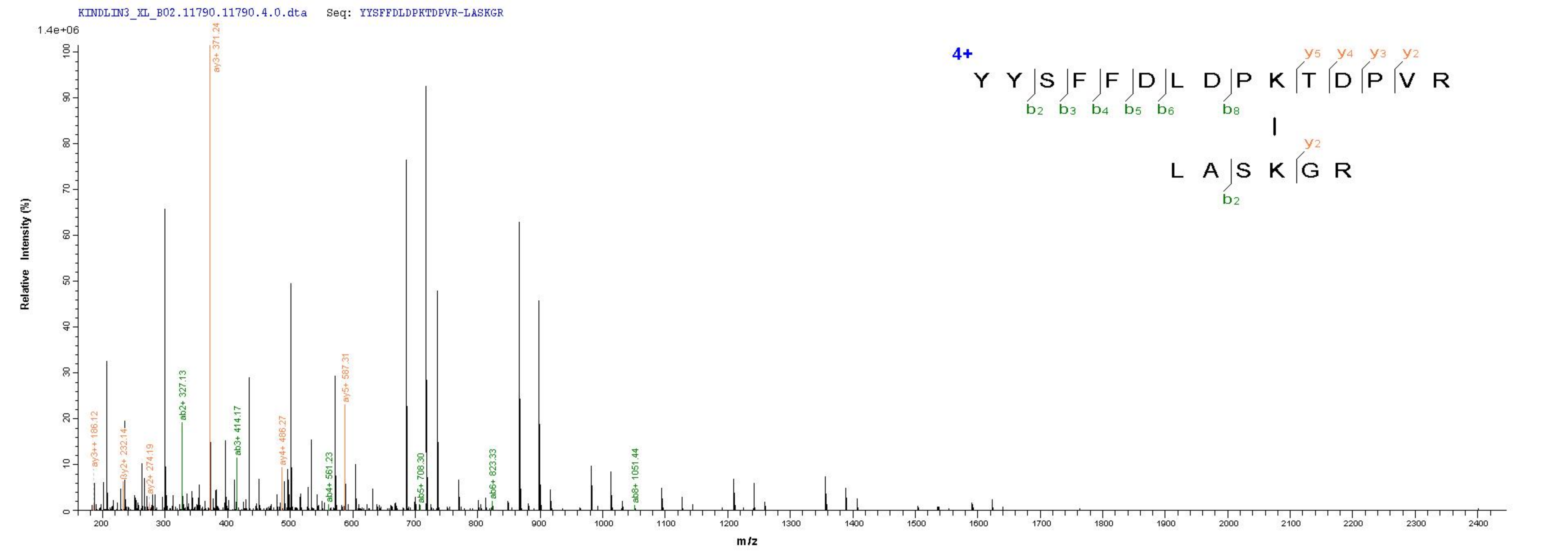
KINDLIN3_XL_B02.11790.11790.4.0.dta Seq: YYSFFDLDPKTDPVR-LASKGR
1.4e+06
Relative Intensity (%)
m/z
4+
Y Y S F F D L D P K T D P V R
L A S K G R
ay3+ 371.24
ay5+ 587.31
ab2+ 327.13
ab3+ 414.17
ay4+ 486.27
ay3++ 186.12
βy2+ 232.14
ay2+ 274.19
ab4+ 561.23
ab5+ 708.30
ab6+ 823.33
ab8+ 1051.44

KINDLIN3_XL_B02.11797.11797.3.2.dta   Seq: YYSFFDLDPKTDPVR-LASKGR

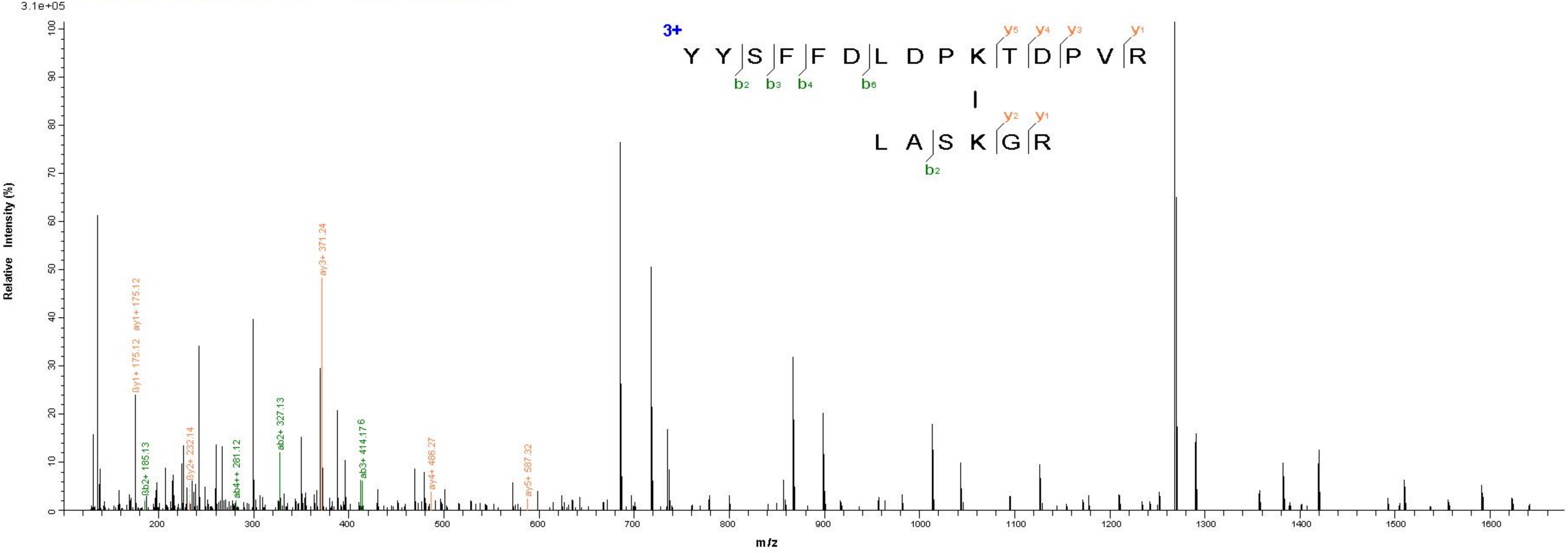

KINDLIN3_XL_B02.11799.11799.3.2.dta Seq: YYSFFDLDPKTDPVR-LASKGR

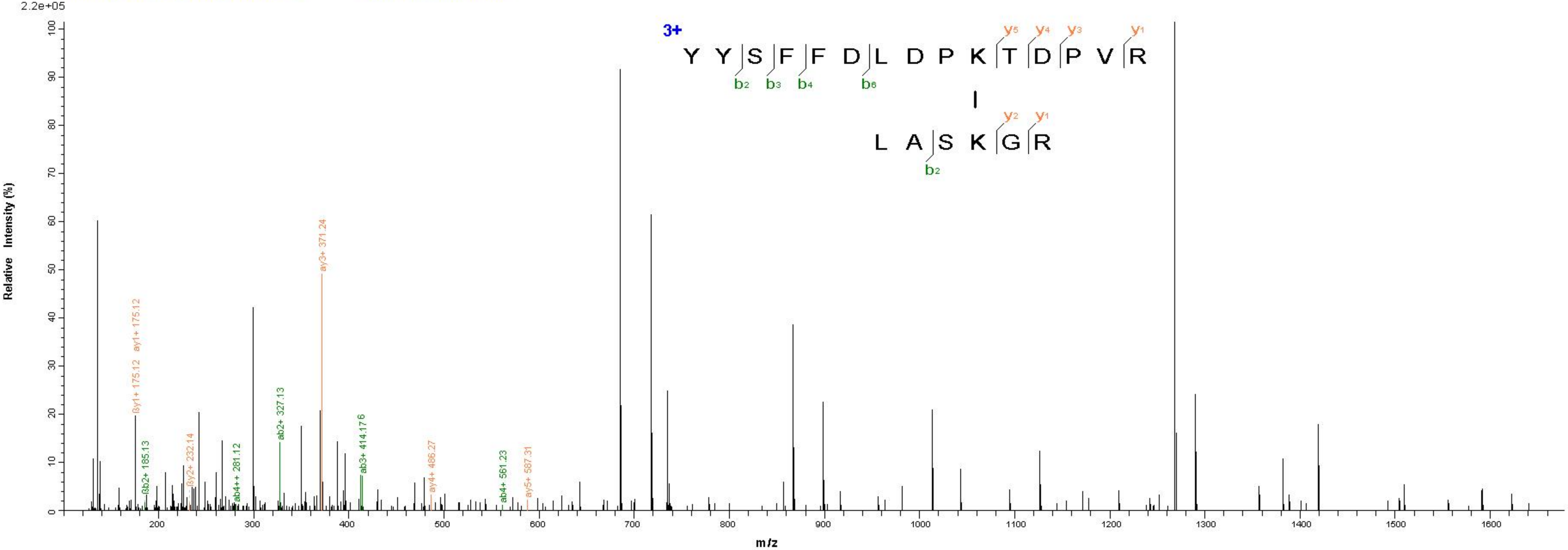

KINDLIN3_XL_B02.11807.11807.3.2.dta Seq: YYSFFDLDPKTDPVR-LASKGR

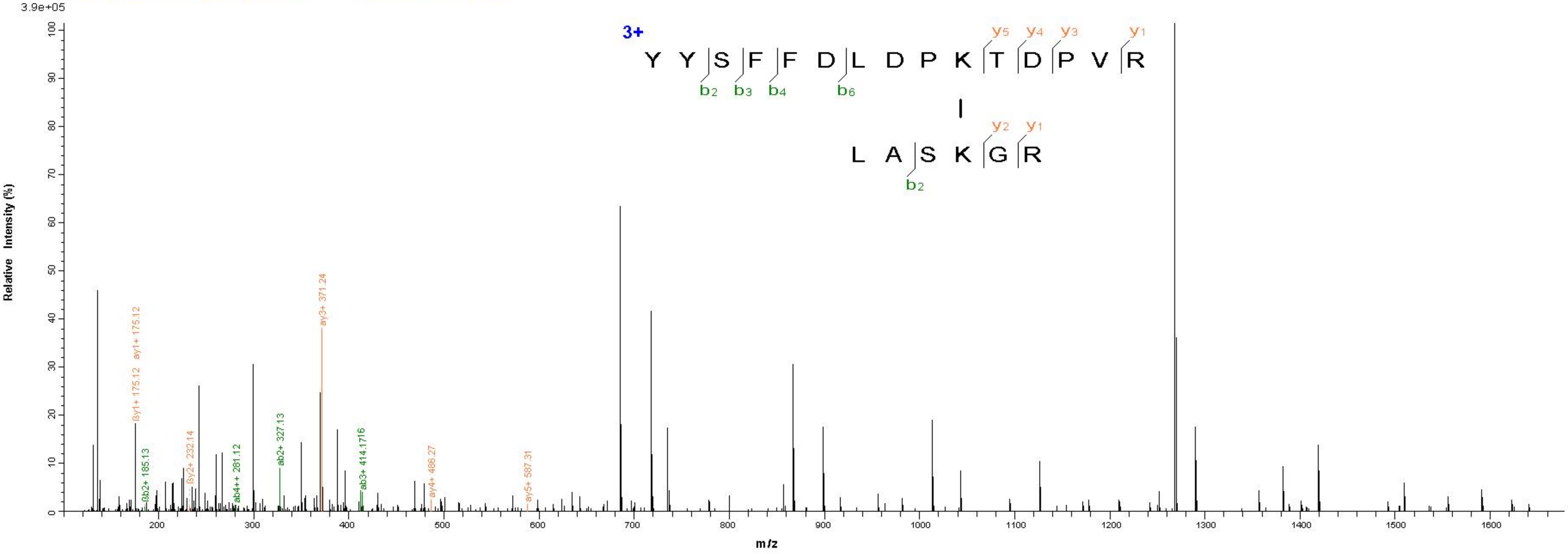

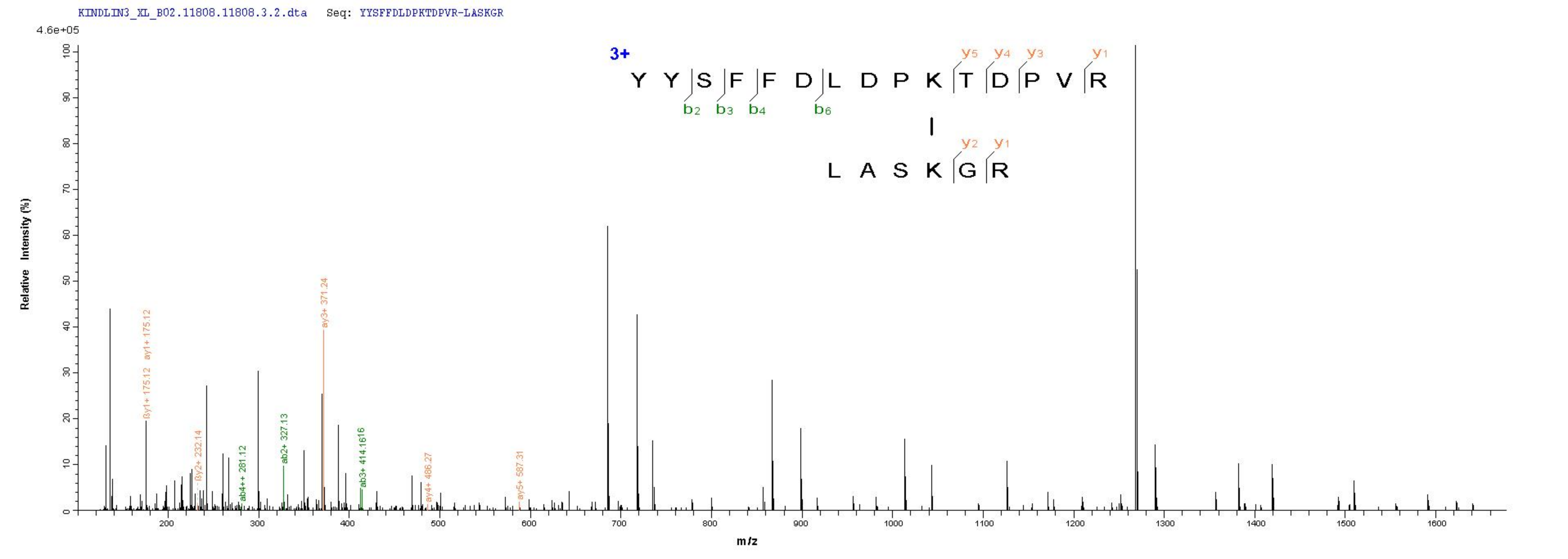
KINDLIN3_XL_B02.11808.11808.3.2.dta Seq: YYSFFDLDPKTDPVR-LASKGR
4.6e+05
3+
Y Y S F F D L D P K T D P V R
L A S K G R
Relative Intensity (%)
m/z
ay1+ 175.12
ßy1+ 175.12
ßy2+ 232.14
ab4++ 281.12
ab2+ 327.13
ay3+ 371.24
ab3+ 414.1616
ay4+ 486.27
ay5+ 587.31

KINDLIN3_XL_B02.11809.11809.3.2.dta Seq: YYSFFDLDPKTDPVR-LASKGR

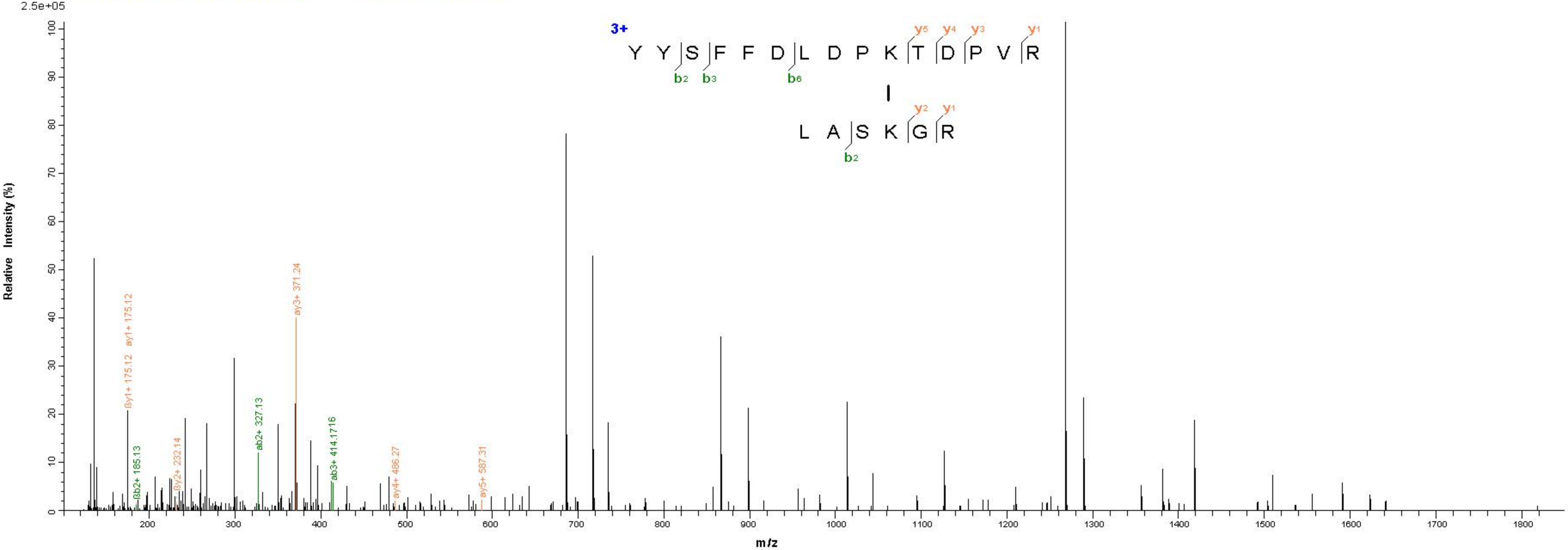

KINDLIN3_XL_B02.11817.11817.3.3.dta   Seq: YYSFFDLDPKTDPVR-LASKGR

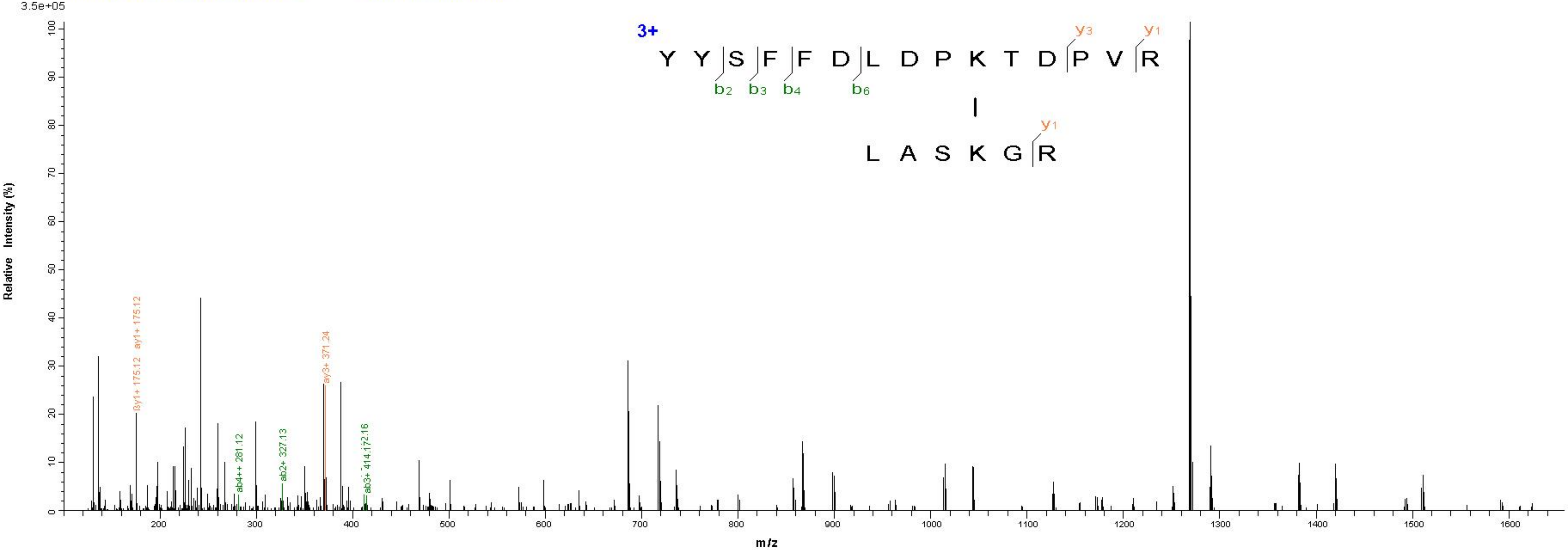

KINDLIN3_XL_B02.11818.11818.3.2.dta   Seq: YYSFFDLDPKTDPVR-LASKGR

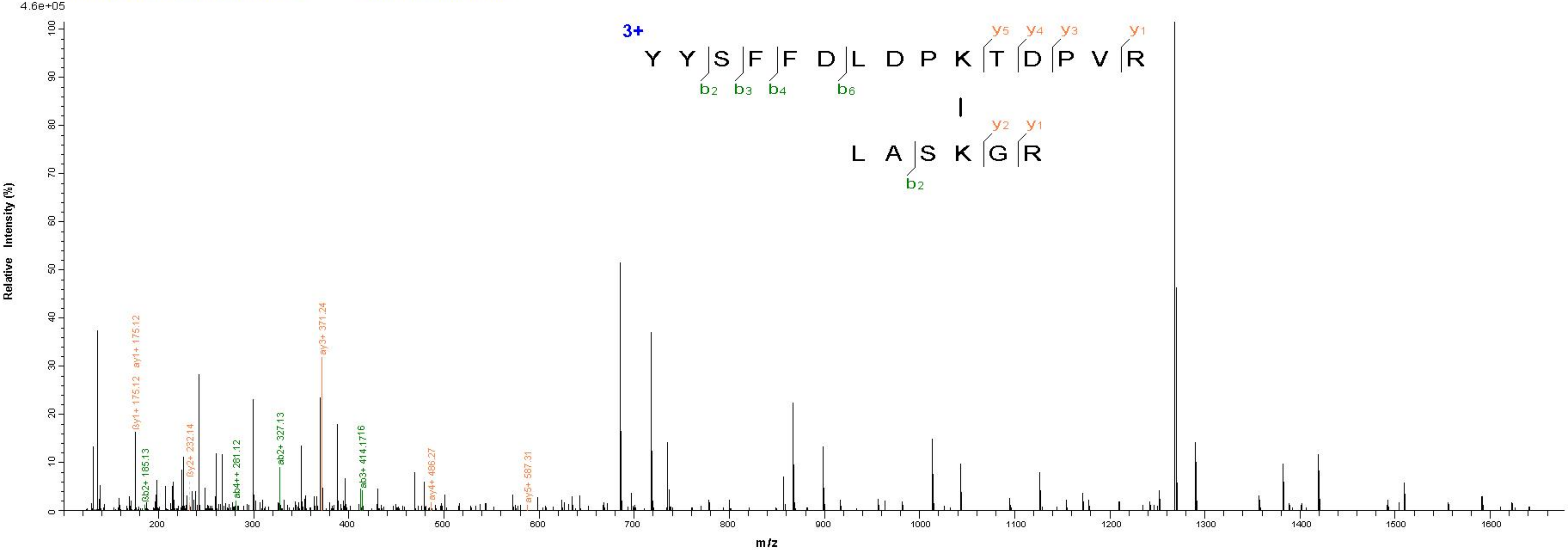

KINDLIN3_XL_B02.11824.11824.4.0.dta   Seq: YYSFFDLDPKTDPVR-LASKGR

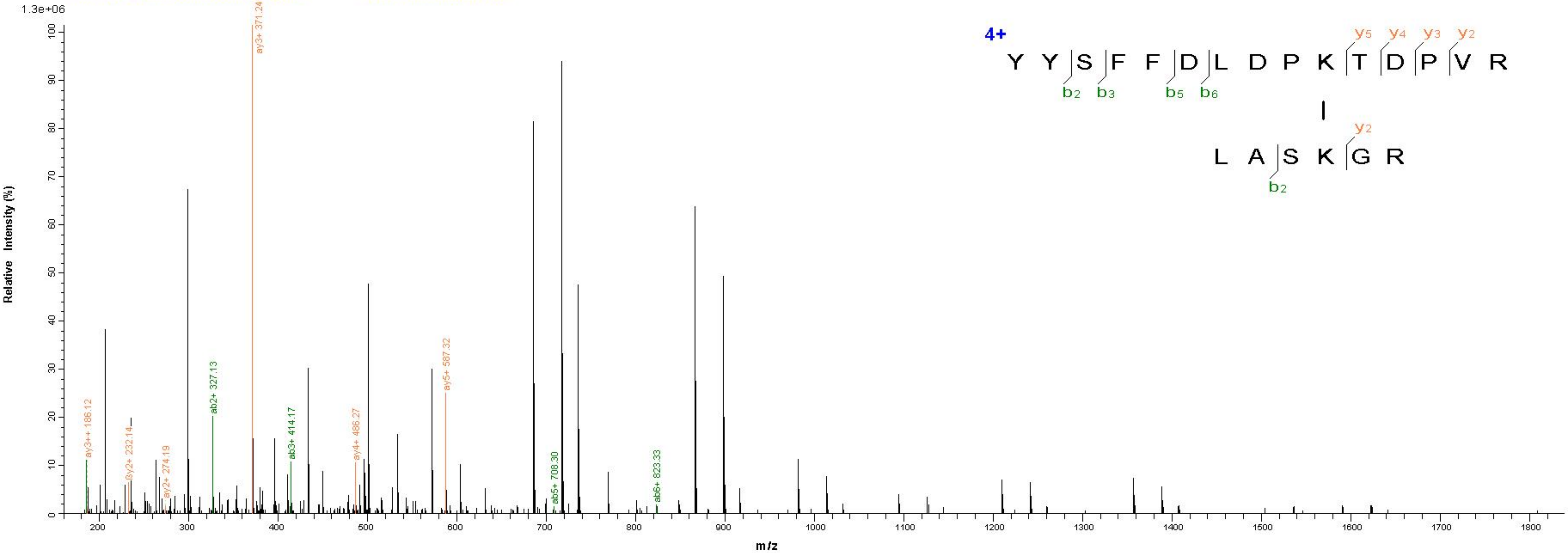

KINDLIN3_XL_B02.11825.11825.3.2.dta Seq: YYSFFDLDPKTDPVR-LASKGR

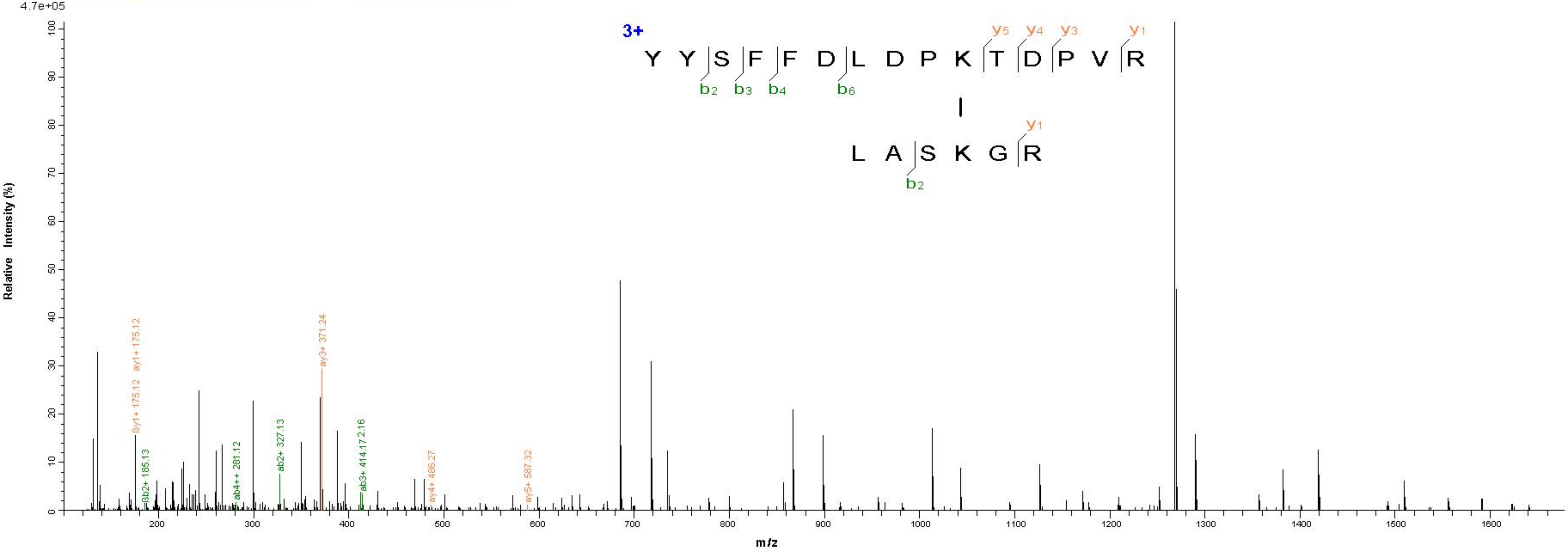

KINDLIN3_XL_B02.11838.11838.3.2.dta   Seq: YYSFFDLDPKTDPVR-LASKGR

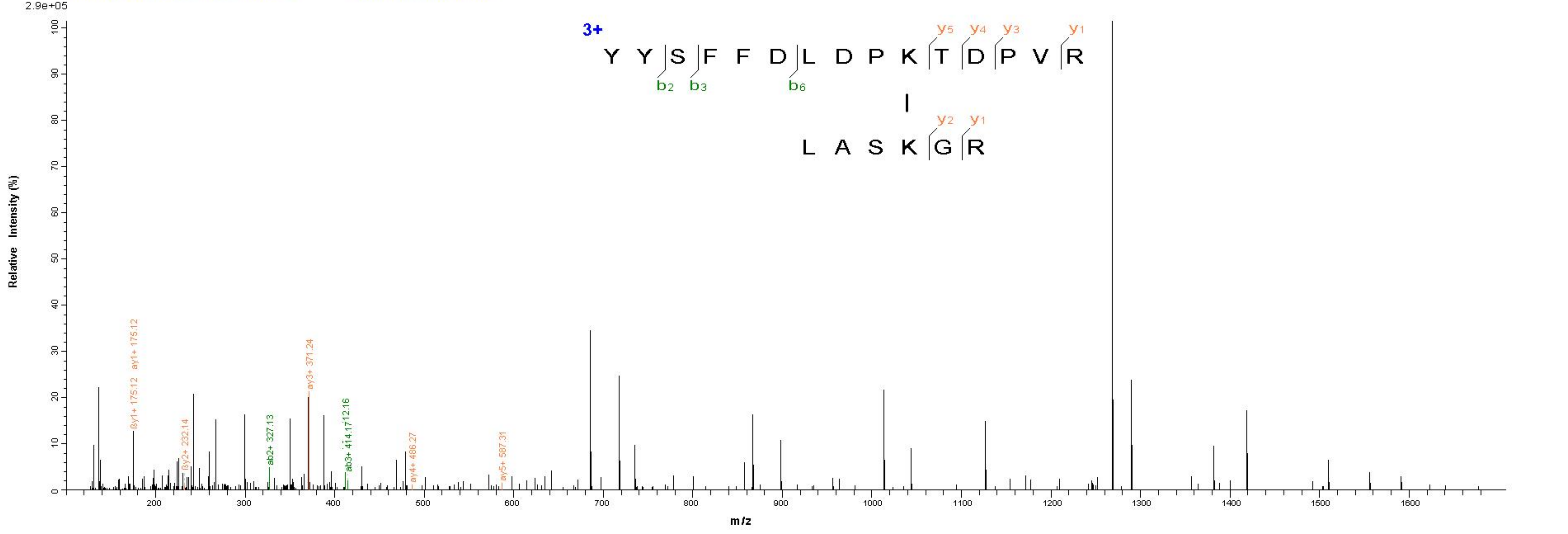

KINDLIN3_XL_B02.11868.11868.4.0.dta Seq: YYSFFDLDPKTDPVR-LASKGR

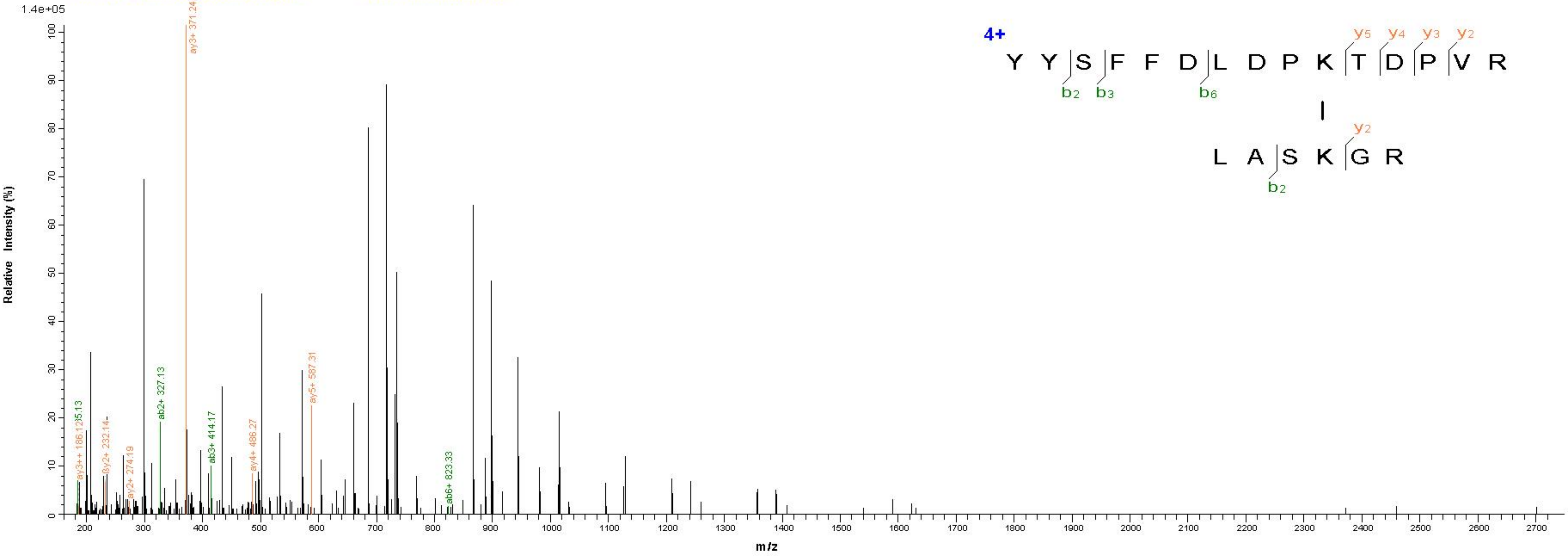

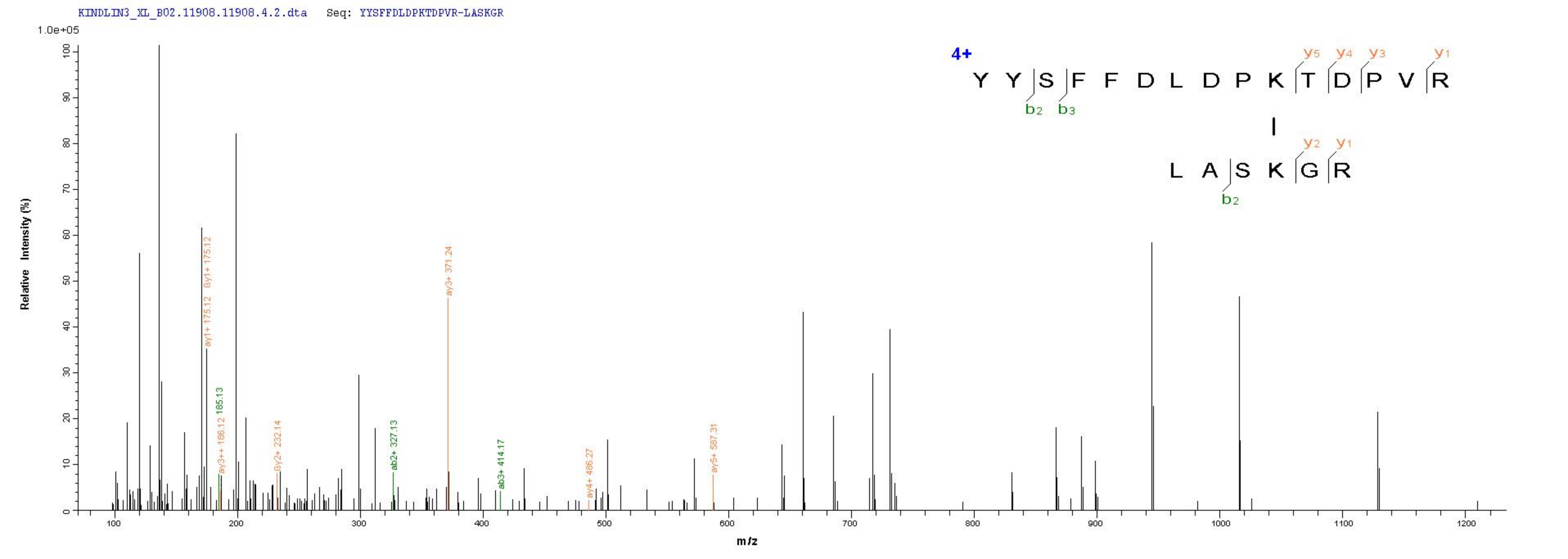
KINDLIN3_XL_B02.11908.11908.4.2.dta Seq: YYSFFDLDPKTDPVR-LASKGR
1.0e+05
Relative Intensity (%)
m/z
4+
Y Y S F F D L D P K T D P V R
L A S K G R
ay1+ 175.12
βy1+ 175.12
ay3++ 186.12 185.13
βy2+ 232.14
ab2+ 327.13
ay3+ 371.24
ab3+ 414.17
ay4+ 486.27
ay5+ 587.31

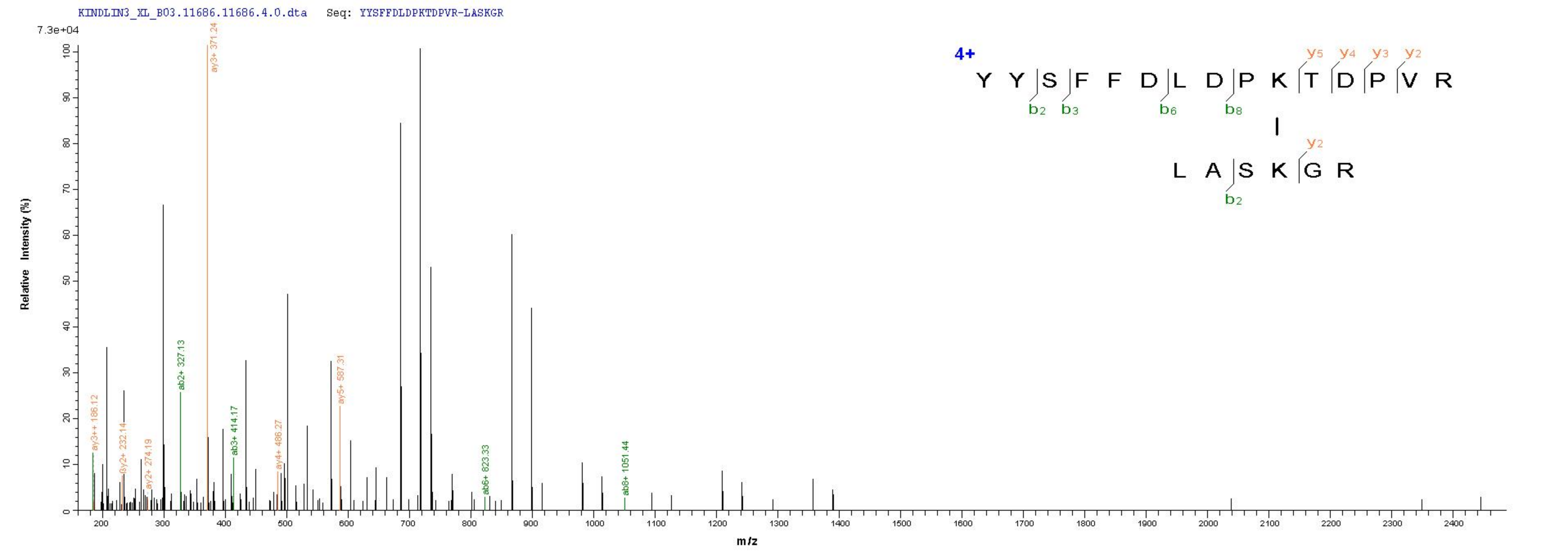

KINDLIN3_XL_B03.11686.11686.4.0.dta Seq: YYSFFDLDPKTDPVR-LASKGR
7.3e+04
Relative Intensity (%)
m/z
4+
Y Y S F F D L D P K T D P V R
L A S K G R
b2 b3 b6 b8
y5 y4 y3 y2
y2
b2
ay3+ 371.24
ay3++ 186.12
ay2+ 232.14
ay2+ 274.19
ab2+ 327.13
ab3+ 414.17
ay4+ 486.27
ay5+ 587.31
ab6+ 823.33
ab8+ 1051.44

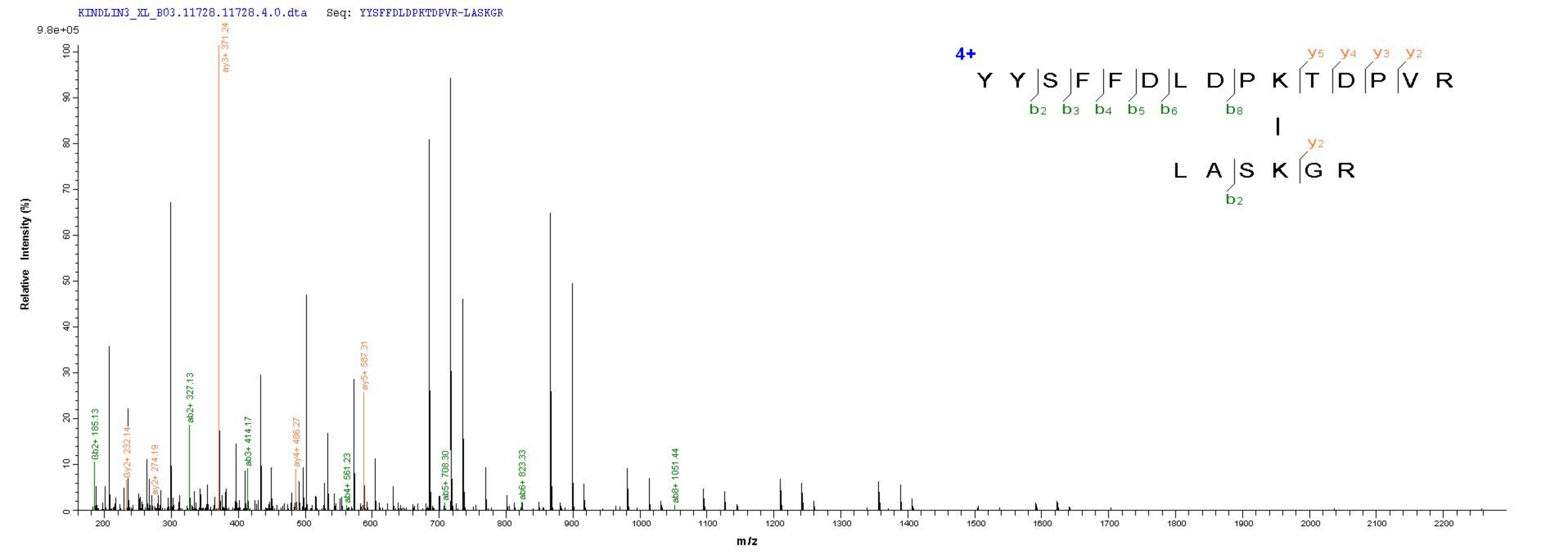

KINDLIN3_XL_B03.11728.11728.4.0.dta Seq: YYSFFDLDPKTDPVR-LASKGR
9.8e+05
Relative Intensity (%)
m/z
4+
Y Y S F F D L D P K T D P V R
L A S K G R
y5 y4 y3 y2
b2 b3 b4 b5 b6 b8
y2
b2
ay3+ 371.24
ay5+ 587.31
ab2+ 327.13
ab3+ 414.17
ay4+ 486.27
βb2+ 185.13
βy2+ 232.14
ay2+ 274.19
ab4+ 561.23
ab5+ 708.30
ab6+ 823.33
ab8+ 1051.44

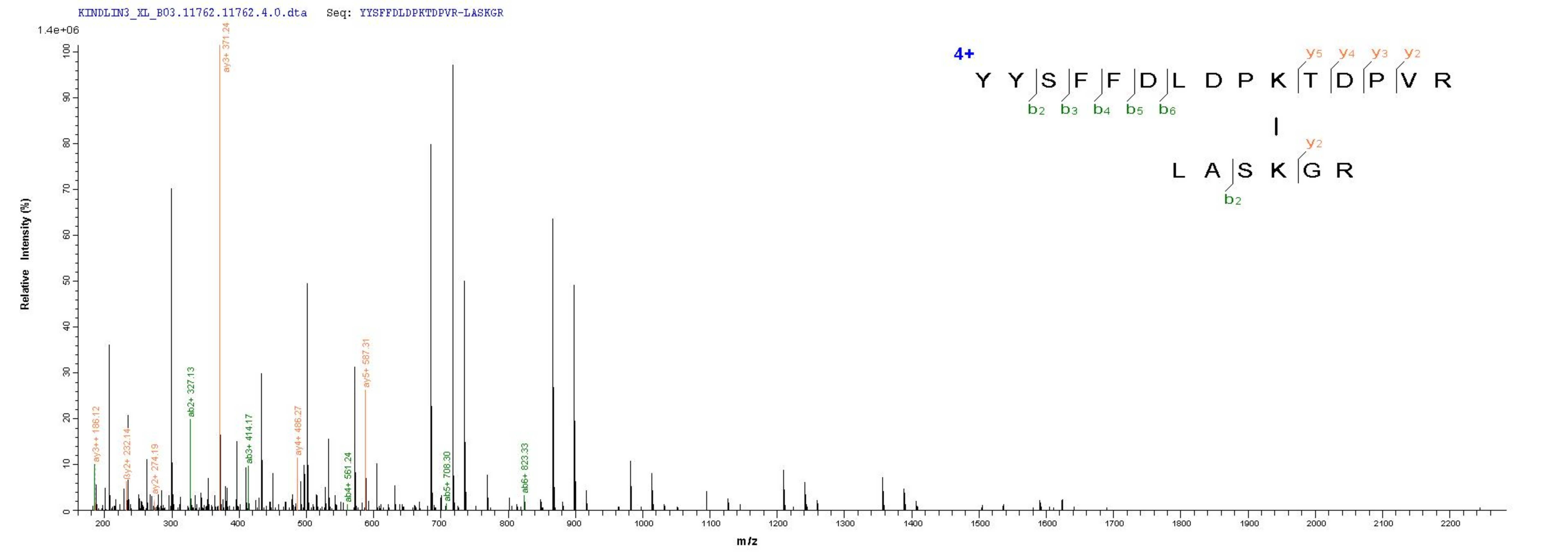

KINDLIN3_XL_B03.11762.11762.4.0.dta Seq: YYSFFDLDPKTDPVR-LASKGR
1.4e+06
Relative Intensity (%)
m/z
ay3+ 371.24
ay3++ 186.12
ay2+ 232.14
ay2+ 274.19
ab2+ 327.13
ab3+ 414.17
ay4+ 486.27
ab4+ 561.24
ay5+ 587.31
ab5+ 708.30
ab6+ 823.33
4+
Y Y S F F D L D P K T D P V R
y5 y4 y3 y2
b2 b3 b4 b5 b6
L A S K G R
y2
b2

KINDLIN3_XL_B03.11810.11810.4.0.dta Seq: YYSFFDLDPKTDPVR-LASKGR

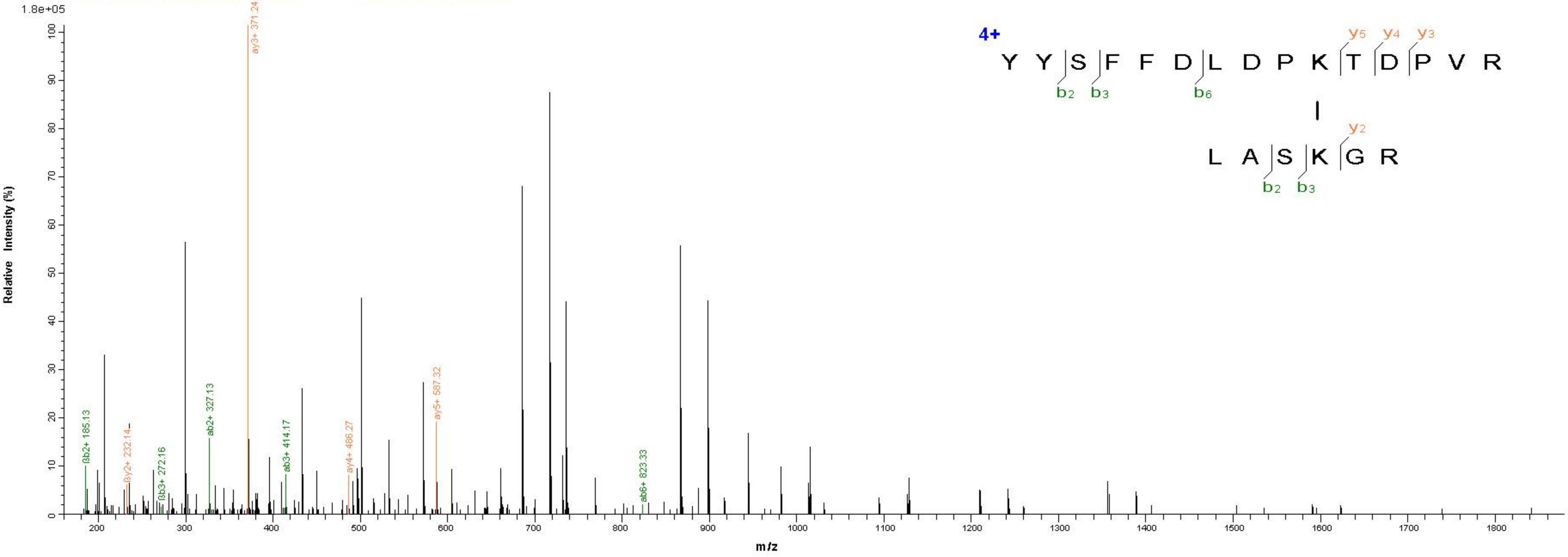

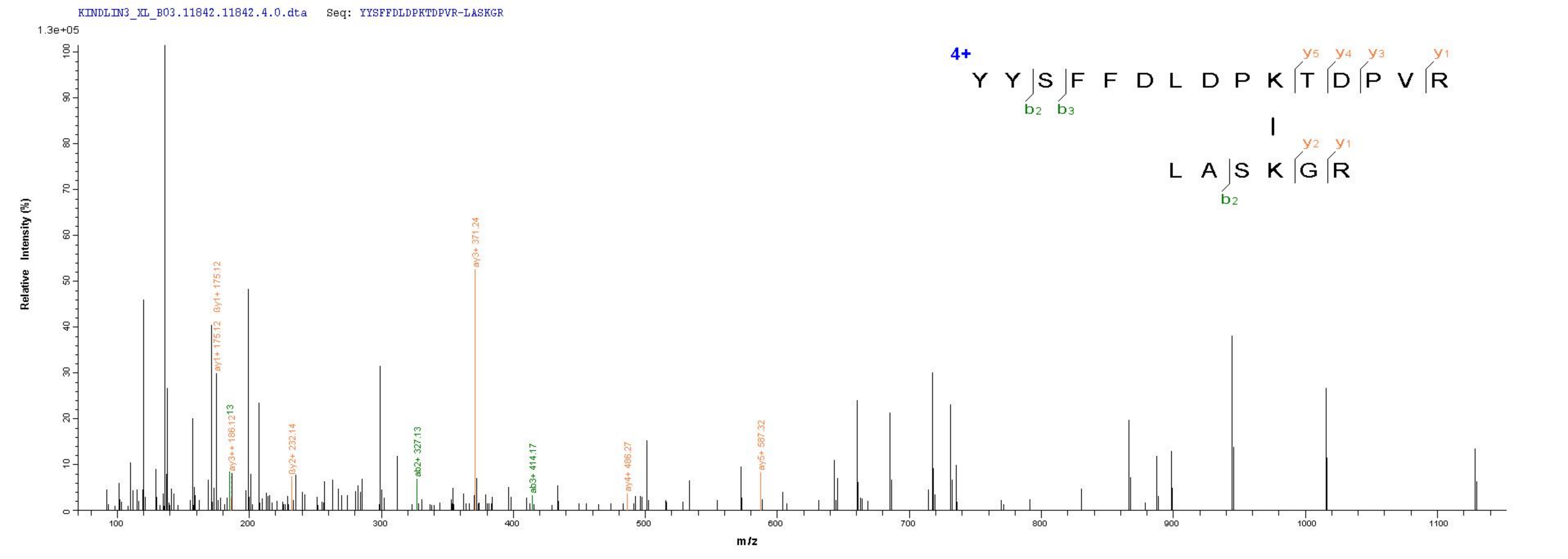

KINDLIN3_XL_B03.11842.11842.4.0.dta Seq: YYSFFDLDPKTDPVR-LASKGR
1.3e+05
Relative Intensity (%)
m/z
4+
Y Y S F F D L D P K T D P V R
L A S K G R
y5 y4 y3 y1
b2 b3
y2 y1
b2
αy1+ 175.12
βy1+ 175.12
αy3++ 186.1213
βy2+ 232.14
αb2+ 327.13
αy3+ 371.24
αb3+ 414.17
αy4+ 486.27
αy5+ 587.32

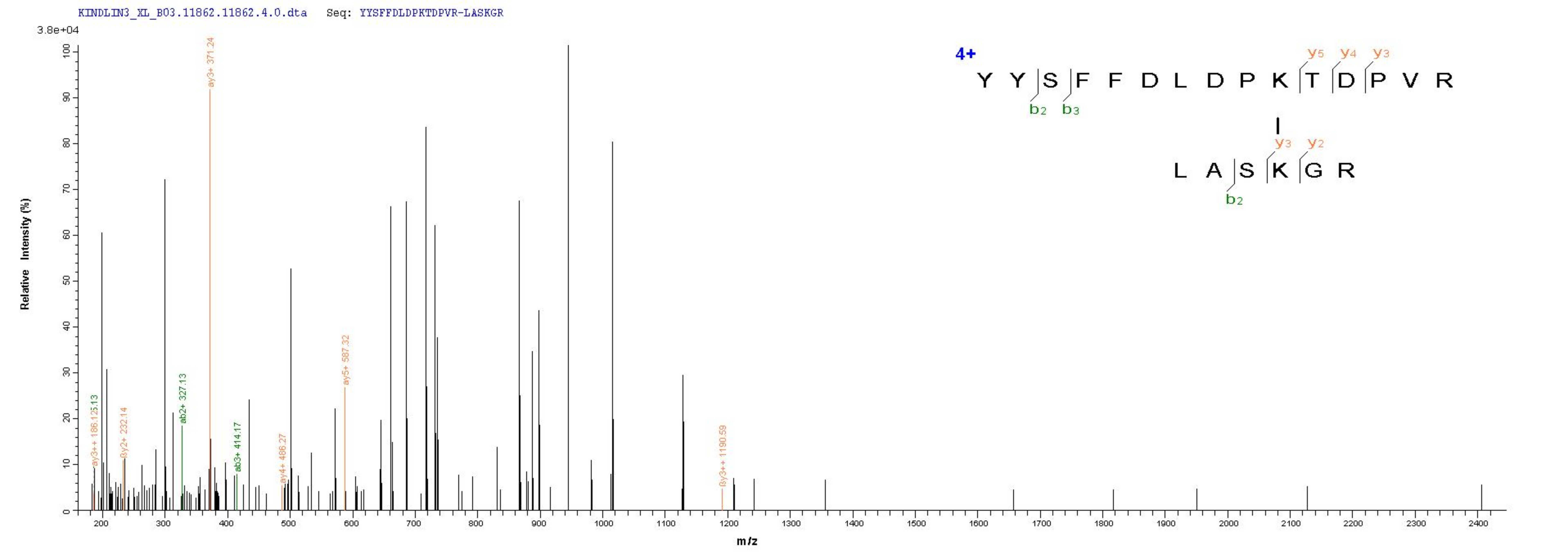

KINDLIN3_XL_B03.11862.11862.4.0.dta Seq: YYSFFDLDPKTDPVR-LASKGR
3.8e+04
Relative Intensity (%)
m/z
ay3++ 186.13
ay3+ 371.24
ßy2+ 232.14
ab2+ 327.13
ab3+ 414.17
ay4+ 486.27
ay5+ 587.32
ßy3++ 1190.59
4+
Y Y S F F D L D P K T D P V R
b2 b3
y5 y4 y3
L A S K G R
b2
y3 y2
