# Supplementary material for: Structural basis of human full-length kindlin-3 homotrimer in an auto-inhibited state
Source: PLoS Biol. 2020 Jul 9;18(7):e3000755. doi: 10.1371/journal.pbio.3000755 (PMC7373317; doi:10.1371/journal.pbio.3000755)
Supplement: S2 Data — (ZIP) [file pbio.3000755.s013.zip › S2_Data.pdf]

Fig. 5C

Left-hand panel shows representative flow cytometry data with expression index (EI) calculated from % gated positive (GP) and geo-mean fluorescence (GM). The plot on the right is derived from three independent experiments with calculated EI as shown in table below. The plot was generated using Graphpad prism. The same software was used for statistical calculations: Two-tailed unpaired Student's t test.

	K3 WT	K3 AFA	K3 F3Δ
Exp1 : EI	28.448	19.488	9.6806
Exp2 : EI	38.808	21.4	16.362
Exp3 : EI	37.323	25.152	18.093

Table 1 eYFP Expression index (EI) of different groups of cells determined by flow cytometry.

Fig. 6B

The data shown in this figure was derived from one flow cytometry experiment in which we examined the cell surface expression level of integrin beta1 on K562 WT cells and the following stable cell lines: K562 K3 KO, K562 K3 KO (K3 WT), and K562 K3 KO (K3 AFA). The values for % gated positive (GP), geo-mean (GM) and Expression Index (EI) (derived from %GP X GM) in each histogram are for that set of data shown.

Fig. 6C.

Supplementary data showing values from shear flow experiments. Note: we only have one shear flow microscope system setup. Hence, only one experiment (Exp) can be conducted at any one time. For example, we conducted K562-WT shear flow without Mn2+ Exp1. After that, we conducted K562-WT shear flow without Mn2+ Exp2 and so on. The plot in main Fig. 6C was therefore derived from at least 3 independent experiments per group. Data were

analyzed and statistics performed using Graphpad prism. The same software was used for statistical calculations: Two-tailed unpaired Student's t test.

Experiment	K562 WT	K562 WT (Mn)	K562 K3 KO	K562 K3 KO (Mn)	K562 K3 KO (K3WT)	K562 K3 KO (K3WT) Mn	K562 K3 KO (K3 AFA)	K562 K3 KO (K3 AFA) Mn
1	18.5	99.25	2.75	52.5	39.75	98.5	123.75	140.5
2	27.5	96.25	4	45.25	70	73	128.25	120.75
3	26	75.75	10.25	58	74.75	95.75	120.25	141
4				86.5	43.5			
5					50.75			

Table 2 Values from shear flow of at least 3 independent experiments per group.
