# Supplementary material for: Structural basis of human full-length kindlin-3 homotrimer in an auto-inhibited state
Source: PLoS Biol. 2020 Jul 9;18(7):e3000755. doi: 10.1371/journal.pbio.3000755 (PMC7373317; doi:10.1371/journal.pbio.3000755)
Supplement: S1 Table — (DOCX) [file pbio.3000755.s014.docx]

**S1 Table.** Data collection and refinement statistics

| Crystals | Kindlin-3  SeMet | Kindlin-3  Native |
| --- | --- | --- |
| PDB code |  | 7C3M |
| Data collection |  |  |
| Space group | C 2 2 2 _1_ | C 2 2 2 _1_ |
| Cell dimensions |  |  |
| a (Å)  b (Å)  c (Å)  α (^o^)  β (^o^)  γ (^o^) | 116.1  204.0  269.8  90.0  90.0  90.0 | 115.8  204.6  270.0  90.0  90.0  90.0 |
| Resolution (Å) | 134.90 - 3.79  (3.89 - 3.79)^a^ | 50.0 - 3.60  (3.69 - 3.60) |
| CC_1/2_ (%) | 99.9 (37.1) | 99.9 (54.7) |
| <I/σ> | 12.4 (0.48) | 14.7 (0.97) |
| Unique reflection | 61367 (4493) | 37506 (2714) |
| Redundancy | 82.9 (76.7) | 22.4 (23.5) |
| Completeness (%) | 99.9 (99.2) | 99.9 (100.0) |
| Refinement |  |  |
| Resolution (Å) |  | 49.2 – 3.60 (3.73-3.60) |
| No. reflections |  | 37452 (3686) |
| Rwork/Rfree (%) |  | 29.9/30.9 |
| r.m.s.d. bonds/angle |  | 0.007/1.63 |
| Number of atoms |  |  |
| Protein |  | 13568 |
| Water |  | 0 |
| B factors |  | 171.8 |
| Ramachandran |  |  |
| Favored (%) |  | 81.2 |
| Allowed (%) |  | 16.4 |
| Outlier (%) |  | 2.4 |

^a^ Values in parentheses are for the highest-resolution shell.
