# Supplementary material for: Structural basis of human full-length kindlin-3 homotrimer in an auto-inhibited state
Source: PLoS Biol. 2020 Jul 9;18(7):e3000755. doi: 10.1371/journal.pbio.3000755 (PMC7373317; doi:10.1371/journal.pbio.3000755)
Supplement: Attachment — Submitted filename: Point-to-point responses_20200416.pdf [file pbio.3000755.s016.pdf]

Point-by-Point Response

Comments are colored red.

For the editor's comments

1. - As you can see, Reviewers 2 and 3 are satisfied with the revision and Reviewer 3 has only one further minor request. Reviewer 1 however still has technical concerns and doesn't feel his/her previous points have been adequately addressed. The concerns continue to relate to the physiological relevance of kindlin-3 trimerization and discrepancy with previous work. The Academic Editor has very carefully evaluated these comments. He/she acknowledges that this is a difficult system to study and that previous work (Yates et al., 2012) characterizing full-length mouse Kindlin-3 reports a monomer by SEC and dimer by SAXS. The finding of a homotrimer state via crystal structure is a provocative result and the key title claim of this manuscript. Therefore, the editorial team feel it is essential to have strong and rigorous evidence for this result. We all agree with Reviewer 1 that this is currently not the case. The Academic Editor suggests that definitive support for the trimer state can be obtained by doing SEC-SAXS on a mixture of trimer and monomer and getting an envelope for each.

>>Thanks for the summarized comments, our response:

1.1) We added the requested result for the Reviewer 3 (**S10d Fig**).

1.2) For the Reviewer 1's comments, we have carefully made our response. In particular, we have recently discussed this issue with some excellent crystallography expert in data collection & processing regarding the resolution (cut-off), and apparently our results are fine (also see below response to review 1's comments).

1.3) I don't think that the previous work (Yates et al., 2012) indicated "full-length mouse Kindlin-3 reports a monomer by SEC and dimer by SAXS". Instead, only monomeric mouse kindlin-3 was reported, and we addressed the reason possibly due to the different purification step in the last rebuttal letter. Perhaps there is some misunderstanding or confusion for this.

1.4) We have employed combinative and complementary approaches (both in vitro and in vivo), all data consistently demonstrated the formation of kindlin-3 trimer, in line with our crystal structure. This finding is for the first time reported, and not contrary to the previous work by SAXA in which it is NOT reported as "a monomer by SEC and dimer by SAXS" (Yates et al. 2012). SEC-MAIL is one of the most reliable techniques for the determination of the absolute molecular mass and rms radius rg in solution (Ogawa & Hirokawa, Biophysical Reviews, 2018, 10: 299-306). As a scattering-based approach, SAXS analysis could provide low resolution information about the shape/mass and dynamics, such as rg, Dmax. Similarly, both SEC-MAIL and SAXS require the sample to be mono-disperse. Given this and our other multiple data (particularly negative-staining of kindlin-3 offering the shape with dimension similar to the trimer structure), our data and findings are technically solid and consistently collected through combinative and complementary approaches.

2. - First point (discrepancy with Yates et al., 2012) - the Academic Editor notes that the Yates paper is on mouse kindlin-3. And while the expression system is a weak argument, he/she notes that the differential heparin step and/or concentration dependence could be factors. The concerns raised in the first point can be addressed via text discussion.

>>Yes, as we pointed out the diverse protocols probably account for the trimer not observed for Yates's paper, such as likely loss of the minor kindlin-3 trimer population prior to gel filtration.

3. - AUC has the same caveats as SEC-MALS and SEC, especially since the trimer has a hole in the middle. As noted above, SEC-SAXS would be the best experiment because an envelope of each peak could be made.

>>As aforementioned (see 1.4), these approaches are complementary to each other, but SEC-MALS is one of the most reliable techniques (Ogawa & Hirokawa, Biophysical Reviews, 2018, 10: 299-306). Given the consistent result for the trimer formation through crystal structure, negative-staining EM, analytical gel filtration, SEC-MALS, crosslinking-MS (consistent with crystal structure arrangement as well as molecular weight of crosslinked monomer in line with that of trimer by both SDS-PAGE and analytical gel filtration analysis), and *in vivo* BiFC, further experiment (even SEC-SAXS) will not be indispensable. If any inconsistence from our diverse approaches, we do believe other approach (SEC-SAXS or AUC) could be employed.

4. - The Academic Editor agrees with Reviewer 1 that the DSSO experiment is not complete and is unconvincing. Crosslinking should be done with monomer to see if there is some interconversion to trimer, especially since you crystallized the monomer and got a trimer. And a MWM ladder that has a higher MWM than 200 kDa should be used (the current gel is inadequate).

>>Crosslinking experiment with kindlin-3 monomer sample was performed. There is a crosslinked trimer observed on SDS-PAGE and analytic gel filtration (**S7a-b Fig**). But this is very dynamic and transient, and only it can be captured by crosslinking (not like a clear band for the cross-linking of trimer). As for the ladder, we want to keep it consistent for monomer kindlin-3 as well. Nevertheless, it is sufficient to show the kindlin-3 was crosslinked (in contrast to the untreated kindlin-3), given that: 1) the crosslinked trimer band in our gel is very clear and is consistently slight above 200 kDa (in contrast to the untreated kindlin-3 band), and we have repeated this experiment several times; 2) there might be a deviation between the theoretical MW and the marker. The marker is a relative reference but could not be precise. For example, the theoretical MW of kindlin-3 in our construct should be 77.1 kDa, but we always have the SDS-PAGE band between markers 75 kDa and 63 kDa (Note that SDS-PAGE is used for approximate measurement of molecular weight, not so precise); 3) we also have the analytic gel filtration data for the crosslinked sample, which exhibited a clear trimer peak compared with the non-treated monomer sample (**S7b Fig**).

5. - The MS data is also weak. It is not clear how many times the crosslink was observed, if this is the only interaction detected, and if so, why. There is also no effort to convince us this is an intermolecular interaction vs intramolecular (by distance arguments), nor whether it is reasonable that 30 Å is OK for a spacer that is 12 Å.

>>We have done duplicates for the experiment, and the crosslink was observed for both duplicates with high confidence. This information was added in this revision. We only showed the crosslink of K457-K567 because we wanted to highlight it and make it easy to understand that crosslink result is supportive to the crystal structure of trimer assembly. Furthermore, the crosslinked kindlin-3 was demonstrated to be a trimer, based on both SDS-PAGE (**S7a and S7c Fig**) and analytical gel filtration calibration (**S7b Fig**).

Following the suggestion, we added crosslinking data for intermolecular interactions. As for the reasonable distance, the constraint is generally in the range of 26-30 Å (double the spacer plus some tolerance, see reference: Merkley et al, Protein Science, 2014, 23: 747-759), but it can be up to 33 Å (Casanal et al, Science, 2017, 358:1056-1059) or even longer 40 Å (Merkley et al, Protein Science, 2014, 23: 747-759) due to protein dynamic and domain flexibility.

There are four pairs of crosslinked residues detected by MS with significant signals (**S8 Fig**). We measured the residue distances both intra and inter molecules according to our trimeric crystal structure, listed as the below table. In particular, the 2nd pair is a convincing intermolecular crosslinking (We only showed this one in our previous **S7 Fig**). As we mentioned in text, the intramolecular K457 in PH domain and K567 in F3 domain are quite far from each other, but they are able to form intermolecular crosslinking, in line with their locations at the trimeric interface in our crystal structure.

	peptides	location	Distance(Å)	
			intra	inter
1	KDEILGIANNR(1)-<br>IDLAVGDVVKTWR(10)	F3 domain 567:589	18.4	84.3 (X)
2	LASKGR(4)-KDEILGIANNR(1)	457(PH):567(F3)	75.2 (X)	30.0
3	AGDALWLRFKYYSFFDLDPK(10)-<br>LASKGR(4)	252(F2L): 457(PH)	52.0 (X)	34.1
4	YYSFFDLDPKTDPVR(10)-LASKGR(4)	262(F2L): 457(PH)	32.0	50.0 (X)

Note: the cross "X" indicating excluding that crosslinking.

6.- Regarding the second point (large buried surface area) - this should be addressed directly.

>>large buried surface demonstrates strong interactions for trimer formation, in line with the fact that we did not observe trimer dissociation. But why the dominant population of kindlin-3 is monomer and the regulatory mechanism still remain elusive, and this is an interesting research field for a follow up topic as we indicated in the manuscript.

7.- Other comments-point 1 (regarding the resolution) - we agree with this concern and it should be addressed and updated, as relevant

>> For the resolution cut-off, we have addressed the issue and cited several papers in our last rebuttal letter. We have run refinements and map calculations at different high-resolution cut-offs, and the 3.6 Å map was the best (**S3 Fig**). Our notion and the data presentation are supported by an expert to whom we have recently discussed.

8.- Other comments-point 2 - please at least indicate how many times the experiment was done and what type of data is presented (e.g. average).

>> We added it. For cross-linking MS, we have done two replicates, with those consistently observed cross-linked peptides in all experiments/replicates shown in **S8 Fig** (and also see the above table). For the native data collection, we collected 3 highly redundant data sets from 3 isomorphous crystals and merged the data together, with the multiplicity of the merged data very high (>20 overall), as indicated in our last rebuttal letter.

Reviewer #1:

First, as I mentioned last time, Yates et al., 2012 only observed kindlin-3 monomer using the similar insect cell expression system. Structurally Yates et al also only observed the monomer. In previous version, the authors ignored this apparent contradiction and simply explained the trimerization they observed in insect cells but not in E. coli was due to the expression condition. In this revised version, the authors argued that the purification protocols are different in the two studies. This is very weak argument. Yates et al purification protocol is standard!

>>We did not have any negative comments on the Yates's purification protocol, but we pointed out the diverse protocols probably account for the trimer not observed for Yates's paper. If only kindlin-3 monomer was pooled for subsequent experiment, of course only kindin-3 monomer would be observed, this scenario is also in line with our observation that "we did not observe concentration-dependent oligomerization or aggregation" as stated previously.

If their conditions could not reveal a trimer, I would strongly suspect whether the authors' experimental conditions generated some non-physiological oligomer in the minor peak position, which the authors claim as a trimer in order to support their crystal structure. Please note that even based on the authors' purification condition, the monomer is predominant from the gel filtration. The so-called "trimer" might be an oligomer or aggregation depending on the injected protein concentration and some unknown reasons such as disulfide-bond induced cross linking. I say this because the size exclusion chromatography largely depends on stokes radius of molecules that gives uncertain molecular weight (MW) information. Thus, the SEC-MALS (Figure 4c) may not always provide conclusive evidence but limited information for estimating apparent MW of proteins. One cannot eliminate the possibility of the observed minor peak as a dimer or tetramer or an oligomer! Indeed, in the newly generated figure of size exclusion chromatography (Fig. 4a, b), the elution positions of MW standards do not seem to match. More quantitative assessments such as analytical ultracentrifugation (AUC) are needed for characterizing this minor peak. Even if the authors can approve this is a trimer in vitro, the physiological relevance of the minor population of such trimer is questionable.

>>We clearly stated that we did not observe concentration-dependent oligomerization or aggregation, so trimer kindlin-3 is not subjected to concentration or disulfide-bond induced cross linking because we added reduced agents in the preparation buffer and no disulfide-bond was observed in our trimer structure. As for the molecular mass by SEC-MALS approach, please see my response to editors' comments (1.4). As for the elution positions of MW standards alter slightly (not exactly the same), because the samples were prepared at different time, and we used different analytical gel filtration columns (although same type), this definitely would have very minor influence on elution volume even for the same sample. However, we ran the standard proteins each time with the target proteins to generate the

accurate calibration curves, this simultaneous calibration curve can make the calibrated MW accurate. We have done experiment to show that disruption of kindlin-3 trimerization exhibits overt integrin activation and increased cell spreading (**Fig 6**). Definitely, more and further experiments to investigate the physiological function of monomer, the trimer, and its regulation are required, that would be also very interesting and attractive.

Second, the human kindlin-3 utilized for crystallization was a monomer as the authors described in the main text. This strongly indicates that the trimer they observed in the crystal lattice is due to the crystal packing. This kind of phenomenon was already previously observed in mouse kindlin-2 that began with monomeric form during crystallization but was crystallized as a dimer (Li et al., PNAS, 2017). Consistent with my view of the crystallization artifact, the large buried surface area (~1,850 A^2) in the trimer interface is huge. Such huge interface would lead to a very tight trimer as a major form. By contrast, the authors observed the monomer predominantly and so-called "trimer" was a very minor peak in the size exclusion experiment. This again indicates that the so-called "trimer" in the crystal structure is physiologically irrelevant, which is clearly a crystallization artifact.

>>We have demonstrated trimer formation both in vitro and in vivo, as well as physiological importance of the trimer. The in vitro evidence includes combinative and complementary approaches, all are in support of trimer formation and consistent with our crystal structure of a trimer. As for large surface buried with monomer/trimer/physiological relevance, see our response to editors' comments (particularly 1.4, 4, 5 & 6). Furthermore, regulation of monomer and trimer formation, as well as protein quality control and degradation, all remains unknown. So we don't think we can simply conclude "a very tight trimer must be existed as a major form during protein expression and preparation"

Other comments:

1). the crystallographic data collection statistics is still poorly characterized. The authors failed to incorporate the Rmerge/R-meas in the data collection statistics table that is essential component for the main table in crystal structure report. Demonstration of all the essential components in the data collection and refinement statistics in the main table is crucial. The R-meas of over 500% in outer shell (500% discrepancy for the measured intensities!) are not acceptable by standard criteria. The resolution limit would be thus around at 4.3-A according to their statistics. The authors need to more carefully reevaluate the resolution limit followed by structure refinement. The authors also need to state the number of native data sets to be merged in the method section.

>>We have addressed this issue and cited several papers with regards to Rmerge/R-meas in our previous rebuttal letter. Again, we have run refinements and map calculations at different high-resolution cutoffs, and the 3.6 Å map was the best (**S3 Fig**). Our notion and the data presentation is supported by an expert to whom we have recently discussed.

2). the ITC measurement for the binding of kindlin-2/-3 to integrin has been criticized by all three reviewers but the authors did not seem to reply to their concerns for distinct binding affinity between two isoforms of kindlin and technical viewpoint. No saturation point was observed for the binding between monomeric kindlin-3 and integrin (Fig. S9a), but was observed for monomeric kindlin-2 (~200 uM vs. 13 uM).

>> The distinct binding affinities between kindlin-2 and kindlin-3 (with lower affinity) has been reported by other groups and we have mentioned it in text and the last response. Due to the relative low binding affinity that almost reach the detectable limitation of ITC, it's hard to get saturation for kindlin-3 monomer for integrin binding assay. If we lower the kindlin-3 concentration (lower than 50 μM), no binding will be detected. If we increase the integrin tail concentration (higher than 800 μM), then precipitation will be observed. Nevertheless, it almost reaches the plateau for the titration curve of monomeric kindlin-3 with integrin.

3). Fig. S10 seems to be the overlay of two structures instead of three as described in legendary.

>>There are clearly three structures overlay in this figure (**S11 Fig** in this new resubmission, three structures colored deep teal, yellow, gray, respectively).

4). Line 540, typo, grid. There are several other typographical errors and uncertain sentences in the revised text.

>>Thank you for this suggestion. We have corrected this typo.

Reviewer #2:

The authors made a nice effort to address my previous concerns. I am fully satisfied with their approach at this point and recommend the paper for publication.

>>Many thanks for the encouraging and rather fair comments.

Reviewer #3:

The authors made large changes to the manuscript in response to the comments. The rather speculative at this stage phosphorylation part of the study is completely removed, making the paper clear and focused. Now the story is straightforward: trimers are observed by crystallography and detected in cells; trimers do no bind integrin; mutants disrupting trimer formation enhance intergrin activation; therefore, trimer formation may be a mechanism of kindlin activity regulation and should be considered in the functional models. In addition, dimers that were previously proposed as important, have not been detected and may have been an artefact of truncation in the previous studies. In my view these are important messages for

the field which may lead to a revised functional model of kindlin activity. All my comments have been addressed, mainly by the removal of more speculative or poor quality data. Writing now is clear an easy to follow.

The only surprising omission is the lack of ITC data for K2 trimer. This should be fairly simple to measure as the authors have both monomers and trimers of K2. I suggest that these data are included in the manuscript.

>>Appreciate for the positive comments, and we added the ITC data for K2 trimer (**S10d Fig**).
