# Supplementary material for: Structural basis of human full-length kindlin-3 homotrimer in an auto-inhibited state
Source: PLoS Biol. 2020 Jul 9;18(7):e3000755. doi: 10.1371/journal.pbio.3000755 (PMC7373317; doi:10.1371/journal.pbio.3000755)
Supplement: Attachment — Submitted filename: Rebuttal_Letter__YGAO_PBIOLOGY-D-19-02291R1.doc [file pbio.3000755.s015.doc]

**Point-by-Point Response**

Comments are colored red.

Reviewer #1: 

This is potentially significant manuscript showing that kindlin-3 might form homotrimer in an autoinhibited state to prevent integrin binding and activation. Previous structural studies on homologous kindlin-2 showed a crystal structure with deletion of PH domain and the protein undergoes monomer/dimer transition although the dimer formation was extremely slow (takes several days). The monomeric structure in this manuscript is convincing but I have major concerns in the trimer study that is the core of the manuscript.

>> Thank you for the positive comments, we have addressed the major concerns below.

**Major points**

1. The authors claimed that they observed pure monomer of kindlin-3 in *E. Coli* system but monomer/trimer equilibrium in insect cell system (sf9). However, the whole manuscript did not describe any experimental conditions about how they observed trimer by gel-filtration. The trimer claim in Fig 2d is not convincing at all since this is superdex 200 with no molecular marker indicated. I can argue that that peak is a dimer or an oligomer or aggregate. Oligomer or aggregate is actually possible since negative staining in Fig. 2f showed a 10 nm particle size that is unrealistically big for a 228 kD trimer. Much more convincing evidence for the trimerization is needed by some definitive techniques such as analytic ultracentrifugation and light scattering.

>> We observed both monomer and trimer peaks with gel filtration chromatography (preparation buffer: 20 mM Tris-HCl pH7.5, 150 mM NaCl, and 5mM β-ME, without incubation) when kindlin-3 was expressed and purified in insect cell system. And the same monomer and trimer forms were also observed in solution with analytical gel filtration chromatography (20 mM Tris-HCl pH7.5, and 150 mM NaCl) and SEC-MALS (20 mM HEPES pH7.5, and 500 mM NaCl). All purification steps, analytical gel filtration chromatography, and SEC-MALS conditions are described in “Materials and Methods” sections 1, 4, and 7, respectively.

Following your kind suggestion, we have added the molecular marker indications in our profiles. These molecular makers are from GE Healthcare gel filtration chromatography calibration Kit HMW as described in Method section 4.

The particle size in negative staining EM is around 10 nm, which is comparable to the size (~110 Å x 110 Å x 90 Å) of kindlin-3 trimer based on our crystal structure. Nevertheless, combinative approaches were employed to confirm the trimer formation. Firstly, SEC-MALS analysis of two populations of both kindlin-3 and kindlin-2 revealed trimerization (**Fig 4c**, revised version). Secondly, crosslinking-MS was also performed to analyze the possible trimer formation. Indeed, the crosslinked kindlin-3 trimer band (above 200 kDa) was detected by SDS-PAGE, and mass spectrometry analysis demonstrated cross-linking between two lysine residues (~30 Å apart based on the structure), located in the PH and F3 domains in two protomers, respectively, which is in line with their positioning in the trimer interface in our structures (**S7 Fig**).

2.The claimed trimer from insect cells contradicts from a previous biophysical study using the same system, which showed only monomer (ref 16). Therefore, the authors’ explanation that only insect cell system produces trimer is not correct. Is it possible that the authors observed some concentration-dependent oligomerization or aggregation? Again, no experimental conditions were provided for how they detected trimer vs monomer.

>> In ref. 16 (in the previous version), the authors reported that kindlin-3 is predominately monomeric. The possible reasons for not observing the kindlin-3 trimer are the following: 1) the majority of kindlin-3 expressed in insect cells is monomer, based on our data; 2) using a different purification procedure to ours. In ref. 16, prior to gel filtration chromatography, additional purification step was carried using HiTrap heparin column, which may result in the loss of the minor kindlin-3 trimer population. We consistently observed two peaks during gel filtration chromatography, representing monomer and timer, respectively. These two kindin-3 states were further characterized by combinative approaches (described in our Methods section and also addressed in comment 1). Notably, we did not observe concentration-dependent oligomerization or aggregation.

3. The ITC studies on the trimer vs monomer binding to integrin are not convincing since ITC is more reliable on strong binding Kd <10 microM). The monomer kindlin-3 binds to integrin at ~200 microM here, which is extremely weak and usually not detectable by ITC. Some alternative method is crucial for confirm the integrin binding studies by ITC. For example, how about GST pull down to show the difference? The authors claim the trimer formation is conserved among kindlins. Since kindlin-1/2 bind much tighter to integrins, which may be easier to be measured, the authors should use one of them (preparation of these kindlins is well established in literatures) to demonstrate this crucial point, i.e., monomer binds better to integrin than trimer.

>> For the ITC studies, we have repeated the experiments several times to verify the data. To demonstrate that the low binding affinity of kindlin-3 for integrin was not caused by non-optimal ITC experimental conditions or data analysis, we also included the binding assay with kindlin-2 monomer expressed and purified from insect cell and integrin β1. The binding affinity of kindlin-2 with integrin (Kd of ~13 μM) was much stronger than that of kindlin-3 with integrin (Kd of ~200 μM) under the same conditions. Furthermore, this significant difference in binding affinity has been reported previously (ref. 32) and can be explained by their variant locations. Unlike kindlin-1/2, which are readily detected at classical focal adhesion, kindlin-3 is not, suggesting weaker and transient interactions with integrin. As for the GST pulldown experiment, it appears to have a high chance of unspecific binding. Also, it is difficult to compare the binding affinities of various samples. Thus, our attempts were not successful.

4.1 From the crystallographic site, the authors reported the crystal SG of C2221, where z equals 8, resulting in several options of monomer numbers per the asymmetric unit. Based on the reported crystal parameters, the authors determine three molecules in the asymmetric unit, resulting in quite outlier values of Matthews probability and solvent content (Vm=3.47 A**3/Da; Solvent=64.58%). Have the authors ever tested to analyze four molecules (as dimer of dimer) in the asymmetric unit, where more feasible values could be accountable (Vm=2.60 A**3/Da; Solvent=52.77%)?

>> We have attempted to search for 4 molecules per ASU using phenix.phaser. The same trimer was observed with an additional fourth molecule (Fig. r1). However, the fourth molecule clashes severely with the trimer and thus seems not feasible. Indeed, the solvent content is rather high, we believe this could be one of the reasons for not being able to improve the resolution despite for the strategy of crystal shrinking employed.

Figure r1: The three molecules forming the previously observed trimer are colored in three different shades of green. The fourth molecule is colored red. The previously reported trimer (colored grey) is superimposed.

4.2 Have the authors encountered potential pseudo-symmetry problem?

>> Through Xtriage analysis of the data (4Å - 50Å), we found the height of the largest Patterson peak (with length >15 Å ) is 2.76% relative to the origin, with p_value of 1.00. This indicates the absence of translational pseudo-symmetry.

In addition, L test returned the below result, indicating the absence of twinning.

|  | Our data | Untwinned | Perfect Twin |
| --- | --- | --- | --- |
| Mean \|L\| | 0.495 | 0.500 | 0.375 |
| Mean L^2^ | 0.325 | 0.333 | 0.200 |

4.3 The current resolution (3.6Å) in the manuscript is low, which may cause ambiguity of the trimer determination. In this regard, the Rmerge was not reported in the statistic table but is required for crystal structure report. The authors need to state the Rmerge or equivalent statistic value during data collection and processing (R-meas if XDS program utilized). If not clear, the entire log file of CORRECT.LP needs to be supplemented. As a rule of thumb, the cut-off of I/sI in the outer shell must be satisfied ≥ 2.0 unless specific reason being provided. CC(1/2) in the outer shell must be also ≥ 70%. If the above criteria can affect the high-resolution cut-off, the authors may need to correct the resolution limit followed by structure refinement.

>> Rmeas of the merged data is 0.208 (overall) and 5.16 (top bin). Detailed merging statistics are as follows:

In the native data collection, we collected 3 highly redundant data sets from 3 isomorphous crystals and merged the data together, in an attempt to obtain higher resolution. As a result, the multiplicity of the merged data is very high (>20 overall).

Rmeas is a redundancy-independent measure of the merging of individual reflections. Therefore, it does not accurately reflect the merging quality of the data when datasets from multiple crystals are used. Indeed, it has been shown that datasets with Rmeas as high as 400% behave well in refinement (Bae et al, 2013). In addition, raising multiplicity by 100-fold (by merging datasets from isomorphous crystals) has been shown to cause a 10-fold increase in Rmeas and Rmerge indifferently (Karplus and Diederichs, 2015).

Regarding the resolution cut-off, while top-bin CC1/2 > 0.7 and I/sig > 2 have been traditionally used, there is abundant evidence suggesting that a more lenient criterion can improve the quality of the final model. For example, Bunker et al (2013) reported that weak data with Rmeas ~200%-600%, CC1/2 ~0.1-0.2, and I/sig ~0.5 significantly improved the refined model. Similarly, Noeske et al (2015) used data with Rmeas ~ 205%, CC1/2 ~ 0.4, and I/sig ~0.3. Lariviere et al (2012) used data with Rmerge ~ 1000%, CC1/2 ~ 0.32, and I/sig ~0.6. A comprehensive review can also be found (Karplus and Diederichs, 2015). Furthermore, the refinement R factors for our model falls within the acceptable range and the map quality is good, as is demonstrated in the response to comment 5. We have made extensive efforts in order to obtain the crystals as well as to improve the resolution. This project has been very challenging, which could be the reason for the lack of structural information on full-length kindlins of any kind. Nevertheless, considering the final model and the map quality (please also see comment 5 below) as well as the reports above, we believe the data used for this structure (a cut-off of 3.6 Å) and the model are convincing.

5. The detailed phasing statistics need to be provided in the method section. Anomalous difference Fourier map for total Se atom positions is also required in the supplementary figure. Representative electron density maps (2Fo-Fc) need to show in the supplementary figure. Crystal packing of C-alpha models with unit cell dimensions need to be presented in the supplementary figure.

>> Following the suggestion, we provided the phasing statistics (see below) in Method section 3,

| Phasing resolution | Space group | Se Sites | Anomalous  Completeness | FOM |
| --- | --- | --- | --- | --- |
| 49.59 – 3.80 | C 2221 | 31 | 99.81% | 0.25 |

Resolution-binned anomalous measurability:

Measurability is defined as the fraction of Bijvoet related intensity differences for which |delta_I|/sigma_delta_I > 3.0, min[I(+)/sigma_I(+), and I(-)/sigma_I(-)] > 3.0 holds.

Values > 0.05 indicate good confidence.

As for Anomalous difference Fourier map for total Se atom positions, representative 2Fo-Fc maps, and crystal pacing of C-alpha models with unit cell dimensions, we provided all the required figures in the supplementary information (**S2-4 Figs**). (Note that: the anomalous difference Fourier map is generated using Global Phasing Buster, the map is displayed with Se sites as green spheres and contoured at 3 sigma)

**Minor points**

1. Line 105-106, please provide the technical details to convince readers about the expression, purification and sample preparation as to how the trimer vs monomer was obtained and crystallized.

>> As mentioned in the response to comment 1 in the major points, the technical details of kindlin expression and purification are described in the section 1 of Methods. For crystallization, we cloned the human full-length kindlin-3 cDNA into pET24a vector with a C-terminal uncleavable 6xHis tag and transformed into BL21(DE3) *E. coli* cell for protein expression. The target protein was sequentially purified by Ni-affinity (HisTrap HP column, GE Healthcare), ion-exchange (HiTrap Q HP column, GE Healthcare), and size-exclusion chromatography (Hiload16/60 Superdex 200 column, GE Healthcare) using buffer: 20 mM Tris-HCl pH 7.5, 150 mM NaCl, and 5 mM β-ME. There was only one population of kindlin-3 observed during gel filtration chromatography. Samples were collected and used for crystallization screening. After optimization, we obtained the good-diffraction crystals under conditions containing 3 M sodium formate pH 7.0, 3% w/v xylitol, and 9 % v/v glycerol at 13 ^o^C by seeding. SeMet-labeled kindlin-3 was expressed and purified in the same way and crystals were obtained under conditions containing 3 M sodium formate pH 7.0, 3% w/v D-sorbitol, and 6% glycerol at 20 ^o^C, or 3 M sodium formate pH 7.0 and 3% w/v D-(+)-trehalose dihydrate at 20 ^o^C by seeding.

In order to study the physiological relevance of kindlin-3, we also expressed and purified kindlin-3 in insect and mammalian cell expression systems (see details in Methods section). Briefly, human full-length kindlin-3 cDNA was inserted into pFB-LIC-Bse vector and transformed into DH10Bac cells. Then, the bacmid containing the target gene was extracted and transfected into sf9 insect cells for further amplification. After three passages, the infected cells were collected for protein purification. As for mammalian cell expression, 6xHis-3xGly(linker)-kindlin-3 cDNA was cloned into pcDNA3.1-zeo(-) vector and transfected into HEK 293 (ATCC) cells. All the purification steps and conditions were the same as that for *E. coli* expressed proteins. Both monomer and trimer forms were observed during size-exclusion chromatography (Hiload16/60 Superdex 200 column, GE Healthcare) using buffer: 20 mM Tris-HCl pH 7.5, 150 mM NaCl, and 5 mM β-ME, and were used for further studies, such as analytical gel filtration, SEC-MALS, and native-staining EM.

2. Line 142-144, please discuss why authors ascertain which K3 molecule the PH domain belongs to after all the limited electron density connecting PH domain to F2 domain.

>> The linkage between the PH domain and the rest of the protein is not observed in the electron density, admittedly raising doubt over its assignment to symmetry-related molecules. Under the currently reported configuration, the straight-line distances of the two gaps before and after the PH domain are ~ 45 Å and 35 Å, respectively. If we connect the PH domain to the next closest symmetry related molecule, the distances would become ~ 60 Å and 45 Å, respectively, which are significantly longer than them in the current configuration. In addition, there is a stretch of clearly resolved peptide (**S5 Fig**) between the N-terminus of the PH domain and the C-terminus of the connecting F2 domain. Combining these observations, we concluded the current PH domain connections to be the most reasonable assignments.

3. Line 239-240, the authors proposed that S484 phosphorylated Kindlin-3 monomer is the ultimate activation state, however S484A K3 monomer mutant only exhibited 1M Kd on ITC. Please discuss how such weak interaction can be biologically significant.

>> There is still a lot of work to be done in order to figure out the relationship between kindlin oligomerization and phosphorylation. Currently, we do not have conclusive data to support this hypothesis. Therefore, we would like to withdraw the phosphorylation related content from this manuscript to make it more structure-oriented and less speculative. Trimer-monomer transition and phosphorylation regulation mechanism is a very interesting avenue to explore for us in the future.

4. Typo on line 240, week ◊ weak.

>> This typo has been revised.

5. Line 244-246, residue S484 didn’t show in Fig 2C to support the sentence.

>> There is no electron density for S484 in our structure since it is located in the loop connecting α2_PH_ and α4_F2_. In the revised version, we did not include S484 phosphorylation result.

6. Line 248-249, it is confusing that S484 phosphorylation does not play the major role in kindlin-3 trimer formation, but somehow affect the kindlin-3 function. Does PH domain play the major role, such as PH domain recruitment to the membrane lipid?

>> Actually, both T482 and S484 phosphorylations were reported previously. PH domain was also demonstrated to be crucial in membrane lipid recruitment and further integrin activation (ref. 17). As mentioned in the response to minor point comment 3, we prefer to remove all the content about S484 phosphorylation.

7. Line 266-269, the statements here are unsubstantiated because i. the K3-AFA mutant interaction with integrin β1 has not been tested; ii. the authors need to reveal the technical details of how they obtained monomer and trimer K3 in sf9 cells and confirm the K3-AFA is a monomer even expressed in the buffer condition for trimer.

>> As for the K3-AFA mutant interaction with integrin β1, since the mutations (Q471, A474, and S478) are located in PH domain, not involving integrin β1 binding pocket which is located in F3 domain, so these mutations should not affect the interaction. Nevertheless, we have tested the binding affinity of K3-AFA for integrin β1, as expected, the result demonstrated similar binding affinity as that of native kindlin-3. As for the technical details of how we obtained the monomer and the trimer K3 from sf9 cells, we described it in Method 1, please also see our responses to major points comment 1 and minor points comment 1. Similarly, K3-AFA purification steps and conditions were same as that for wild type K3.

8. Fig. 2d and S6, a molecular weight marker should be included.

>> Following the suggestion, the molecular weight marker has been added to all of the gel filtration chromatography profiles.

9. Fig. 2e, please box a larger area to include more particles.

>> The protein concentration we used for negative staining EM was low and, consequently, there were just a few particles attached to the gird as seen on the figure below. Nevertheless, the particles support trimer formation.

**Reviewer 2:**

The submitted manuscript discusses the structure of full length kindlin-3. A new mechanism of kindlin-3 regulation is proposed, namely the inactivation of kindlin-3 (inability to bind integrins) via homotrimerization.

I would like to say that as a non-structural biologist I was able to enjoy the manuscript as it was very well written and easy to follow. The structural data appears sound although others are likely much more competent to comment. I found it surprising however that monomeric kindlin-3 from insect cells bound to beta1 integrin tails with a surprisingly high KD of 200 micromolar (and no interaction of E. coli-derived kindlin-3). We have previously found kindlin 1-3 to consistently and strongly bind to integrin cytoplasmic tail peptides in pulldown experiments suggesting a lower KD.

One of the main conclusions from the structural data is that kindlin-3 forms homotrimers in vivo. The manuscript unfortunately is a little bit light on the in vivo corroboration of this idea. In fact, I don't think there is any in vivo data presented that would strongly support the idea of kindlin trimers occurring in vivo. A kindlin-3 mutant induces increased adhesion which is in line with the authors hypothesis however no proof that trimers exist in vivo.

Therefore, I was wondering whether the authors are able to generate some in vivo evidence of trimer formation along the lines of FRET or proximity ligation methods? Also, this may help address the question of when, where and how trimers are formed in vivo.

The data presented is definitely worth publishing and it will be up to the editors to decide how much in vivo corroboration is required for this journal. I do think that a little bit more in vivo data would significantly improve the manuscript. They authors talk about developing cancer drugs based on these new findings in the discussion. Prior to taking this leap I would encourage them to further test their idea of homotrimer formation in vivo and make sure that these indeed exist and are important regulators of integrin activity.

>> Thank you for the positive comments and the suggestion for trimer formation *in vivo*. We appreciated the editor for giving us six months to carry out additional experiments.

As for kindlins binding with integrin, please see our response to Reviewer 1, comment 3. We measured the binding affinity for both kindlin-2 and kindlin-3, the former is stronger than the later. Note that our binding parameters for kindlin-2, and the weak affinity for kindlin-3, are in line with the previously published data.

Following the suggestion, we made further efforts to demonstrate the trimer formation occurring *in vivo*. We designed a bead-based Bimolecular fluorescence complementation (BiFc) approach to detect kindlin-3 trimerization in small scale in transfected cells (see our results of kindlin-3 trimerization in cells) (**Fig 5**). Briefly, HEK 293T cells were transfected with three expression plasmids containing HA-kindlin-3, N-eYFP-12Gly-kindlin-3, and C-eYFP-12Gly-kindlin-3. The population of trimer consisting of one molecule each of HA-kindlin-3, N-eYFP-kindlin-3, and C-eYFP-kindlin-3 was immunoprecipitated and analyzed by flow cytometry (**Fig 5a**). We generated relevant expression plasmids for kindlin-3 WT and two variants in order to demonstrate trimer formation via the binding interface determined from our crystal structure (**Fig 5b**). Interestingly, the highest fluorescence signal was detected with kindlin-3 WT followed by AFA and F3∆ captured nano-beads, and the difference was statistically significant (**Fig 5c**). We inferred that AFA mutations diminished kindlin-3 trimer formation but the disruption was lesser than that caused by F3 domain deletion. We noted that there is still fluorescence signal detected in the F3∆ sample. This could be due to non-specific background binding to the beads and low level of self-association between N-eYFP and C-eYFP (a common observation in BiFc experiments) independent of kindlin-3 trimer formation.

Minor points include referring to Kindler syndrome as a skin atrophy disease. This terminology does not exist in dermatology. I would refer to Kindler syndrome as a disorder within the spectrum of hereditary epidermolytic blistering diseases. Likewise, I would refer to LAD-III as a primary immunodeficiency with platelet dysfunction.

>>Following this suggestion, we revised the manuscript accordingly.

**Reviewer 3:**

Kindlins play important role in the integrin activation and clustering. Currently the main information on the kindlin structure is based on the fragment with the deleted F2 loop and PH domain. The manuscript for the first time reports the structure of a complete kindlin3. While showing similar overall fold to the truncated version, the structure does not have the F2 domain swap dimerization. Instead, it forms a trimer where PH domain interacts with the F3 lobe and partially occlude the integrin binding site. In solution kindlin3 exists as a mixture of stable monomer and trimer that can be separated by gel filtration. The trimeric form does not bind integrin, as expected from the crystal structure, while the monomer has a very weak with integrin. Disruption of the trimeric interface by structure-based mutations promotes integrin activation in cells. The authors propose a model where kindlin activity is modulated through trimerization that is, possibly, disrupted by phosphorylation. Dimerization reported earlier for the truncated form has not been detected in the full-length kindlin3 and kindlin2 by the authors.

The structure of the full-length kindlin is clearly important for the adhesion field, and the proposed model of kindlin regulation is intriguing. However, the presented experimental support for the regulation model and the role of phosphorylation is rather tentative.

>>Thank you for the positive comments, we address the reviewer’s concerns below.

1. The lack of inter-conversion between the monomer and the trimer is puzzling. No structural rearrangement is required, so the equilibrium is expected to be dynamic. The extremely slow inter-conversion suggests that an additional factor may be involved that induces trimer formation. This has an implication for the regulation mechanism that the authors should considered and discuss.

>> We are also very interested in studying the mechanism of monomer-trimer conversion and the relationship between oligomerization and phosphorylation. Despite the phosphorylation, it could be very complicated in human since other kindlin-3 binding partners might also be involved in the monomer-trimer conversion, as you mentioned “factor”. For clarity and to lessen ambiguity, we have decided to focus on the structure and trimerization of kindlin. The monomer to trimer transition would be a major follow-up project as we plan to put more effort in it in the future.

2. The authors concluded that non-phosphorylated form does not bind integrin from the ITC data (Fig S3) and detection of pS484 in sf9, but not in *E.coli*. The ITC titrations of S3a and d look very similar, except for a larger buffer mismatch in S3a. However, the binding curve of S3d is not fitted correctly, because it shows systematic deviations from the experimental data. The values of the dltH axis of S3d do not seem to correspond to the titration data – the heat changes are similar to those in S3a, so the dltH values are also expected to be similar. The theoretical curve should be fitted correctly, and the values checked. From the shape of the curve and the raw data, the binding constant is expected to be similar in S3a and d, which invalidates the conclusion about the effect of phosphorylation on the binding.

>> As aforementioned, we do not yet have conclusive data to determine the relationship between S484 phosphorylation and K3 oligomerization, and have therefore decided to exclude this part. However, we have included additional data demonstrating kindlin trimerization.

3. The ITC experiments should also be conducted on the kindlin2 monomers expressed in e.coli and sf9. The reported integrin interaction is stronger for kindlin2, which will make any potential differences easier to detect.

>> Due to solubility issues, we were not able to purify human full-length kindlin-2 from *E. coli* expression system, therefore, the recombinant protein expressed and purified from insect cells was used for ITC binding assays. Both kindlin-2 and kindlin-3 (with N-terminal 6xHis tags) purified from insect cells, were used for ITC assays (**S9c Fig**) confirming that kindlin-2 has higher binding affinity with integrin than kindlin-3, and the Kd of ~13.4 μM for kindlin-2 is consistent with other reports.

4. The lack of interaction for e.coli kindlin3 monomer is hard to explain by phosphorylation anyway, because S484 distant from the integrin binding site. Perhaps the real cause of the difference is the C-terminal tag of e.coli construct that somehow reduces the binding because of its proximity to the integrin binding site. According to methods, the tag was not removed. The authors should repeat the binding experiment with the tag removed.

>> To determine whether or not the C-terminal tag of the kindlin-3 *E. coli* construct affects the interaction with integrin, we generated human full-length kindlin-3 construct with N-terminal 6xHis tag and purified the protein following the same purification steps and conditions as described in Methods. Despite the very low expression level, no binding was detected with integrin with ITC experiment.

5. MS data do not show how large is the fraction of the phosphorylated protein. This is expected to be too low to have any significant effect on the interactions measured in vitro.

>> Phosphorylation will not be included in the revised manuscript.

6. The clustering part of the model in fig.5 is a pure speculation and is not based on any of the presented data. It should be removed from the figure as potentially misleading.

>> Following the suggestion, we have removed this figure.

7. Description of the preparation of the monomeric and trimeric forms should be included in the methods.

>> We observed both the monomer and the trimer forms of kindlin-3 during gel filtration chromatography. More detailed answer is given in the response to major points comment 1 and the response to minor points comment 1 for Reviewer 1.

8. The manuscript has a number of somewhat awkward and not fully clear sentences. Further editing and refinement of the text is recommended.

>> Following the suggestion, we ask colleagues (native English speaker) to help with English proofreading.

**References:**

Bae B, Davis E, Brown D, Campbell EA, Wigneshweraraj S, Darst SA. Phage T7 Gp2 inhibition of Escherichia coli RNA polymerase involves misappropriation of σ70 domain 1.1. Proc Natl Acad Sci U S A. 2013; 110:19772–19777.

Bunker RD, Bulloch EM, Dickson JM, Loomes KM, Baker EN. Structure and function of human xylulokinase, an enzyme with important roles in carbohydrate metabolism. J Biol Chem. 2013; 288:1643–1652.

Karplus PA, & Diederichs K. Assessing and maximizing data quality in macromolecular crystallography. Current opinion in structural biology. 2015; 34, 60–68.

Lariviere L, Plaschka C, Seizl M, Wenzeck L, Kurth F, Cramer P. Structure of the mediator head module. Nature. 2012; 492:448–451.

Noeske J, Wasserman MR, Terry DS, Altman RB, Blanchard SC, Cate JH. High-resolution structure of the Escherichia coli ribosome. Nat Struct Mol Biol. 2015; 22:336–341.
